# Modelling beyond additive risks: A framework for estimating the cascading burden of environmental exposure

**DOI:** 10.1016/j.eehl.2026.100281

**Published:** 2026-09-01

**Authors:** Jinting Guo, Hengyi Liu, Meng Wang, Mingkun Tong, Jianyu Deng, Ning Kang, Tianjia Guan, Tao Xue, Tong Zhu

**Affiliations:** a Institute of Reproductive and Child Health, National Health Commission Key Laboratory of Reproductive Health and Department of Epidemiology and Biostatistics, Ministry of Education Key Laboratory of Epidemiology of Major Diseases (PKU), School of Public Health, Peking University Health Science Centre, Beijing, 100191, China; b Department of Epidemiology and Environmental Health, School of Public Health and Health Professions, University at Buffalo, Buffalo, NY, 14214, USA; c School of Health Policy and Management, Chinese Academy of Medical Sciences & Peking Union Medical College, Beijing, 100730, China; d SKL-ESPC & SEPKL-AERM, College of Environmental Sciences and Engineering, Institute of Tibetan Plateau, and Center for Environment and Health, Peking University, Beijing, 100871, China; e TUWAS-AEEEH, Xizang University, Lhasa, 850000, China; f National Institute of Health Data Science, nstitute of Medical Technology, Peking University Health Science Centre, Beijing, 100191, China; g State Environmental Protection Key Laboratory of Atmospheric Exposure and Health Risk Management, Center for Environment and Health, Peking University, Beijing, 100871, China

**Keywords:** Cascading effects, Interactive distributed lag model, Air pollution, All-cause mortality

## Abstract

Standard models for assessing the acute health effects of environmental exposures assume that risks from daily exposures are additive, ignoring “cascading effects” where prior events modify an individual’s vulnerability. Here, we introduce and validate the Interactive Distributed Lag Model (iDLM), which quantifies these dynamics by incorporating interaction terms between exposures at different lags. Simulation studies confirm that traditional models are biased when cascading effects are present, whereas the iDLM accurately recovers the true risk structure. We applied this model to a nationwide U.S. dataset to analyze the mortality risks of short-term exposure to PM_2.5_ and O_3_. Consistent with the standard models, the iDLM shows that short-term exposure to PM_2.5_ or O_3_ is significantly associated with increased risk of all-cause mortality. For each 10-μg/m^3^ increment in PM_2.5_ and 10-ppb increase in O_3_ on the day of exposure, the risk of all-cause mortality increased by 0.26% [95% confidence interval (CI): 0.12, 0.40] or 0.18% (95% CI: 0.11, 0.24), respectively. We find that PM_2.5_ exhibits a strong, synergistic amplification effect, while the cascading effects of O_3_ are weaker. Critically, we demonstrate that for both pollutants, a substantial component of the mortality burden appears to be driven by cascading interactions, which are largely overlooked by traditional models. These findings fundamentally reframe the understanding of air pollution’s public health impact, revealing that the history-dependent dynamics of vulnerability, not just independent daily insults, are a crucial mechanism of harm.

## Highlights

• The Interactive Distributed Lag Model (iDLM), a novel extension from the standard DLM, captures the overlooked cascading effects of repeated exposure.
• Simulation studies confirm that traditional models are biased under cascading effects, whereas the iDLM recovers the risk structure.
• The demonstrative study based on the nationwide U.S. dataset reveals that traditional additive models are shown to substantially underestimate air pollution’s mortality burden.
• The application of the iDLM framework could extend beyond air pollution, offering a new paradigm for quantifying health effects of any recurrent stressor.

## Graphical abstract

**Figure:**
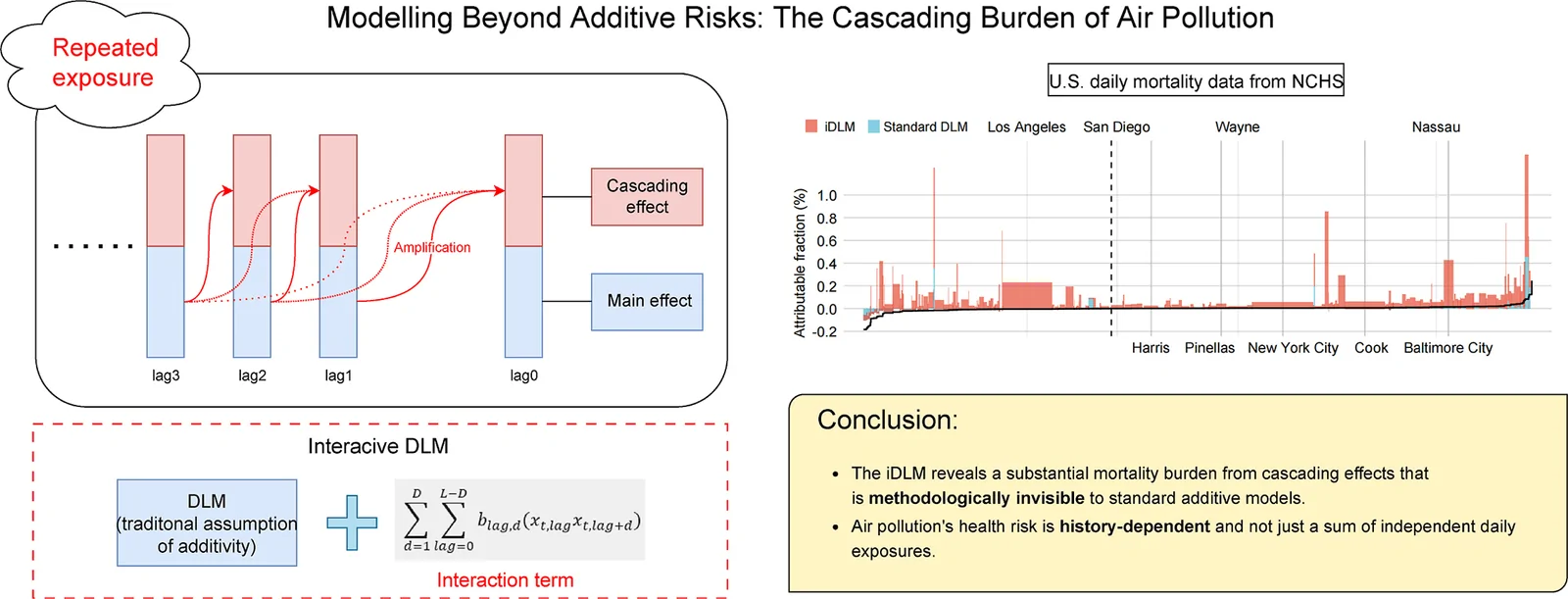

## 1 Introduction

Environmental and climate phenomena often manifest through complex interactions, in which an initial impact can trigger a sequence of consequences, either amplifying or dampening the original effect, which are known as cascading effects [1]. Cascading effects describe a dynamic process in which an initial event triggers a sequence of consequences across interconnected systems, often in a non-linear fashion, which can amplify or dampen subsequent events. It is crucial to distinguish our concept of “cascading effects” from the broader term “compound effects”. While compound effects broadly refer to any non-additive outcome from co-occurring risk factors, cascading effects represent a specific, history-dependent dynamic mechanism in which past exposures actively modify the system’s intrinsic vulnerability to subsequent exposures. Understanding the concept of cascading effects, particularly those involving a primary impact leading directly to a secondary outcome, is vital for effective mitigation and adaptation strategies. For example, prolonged drought increases the risk of wildfires, which in turn can reduce vegetation cover, alter land surface properties, and potentially lead to regional warming and decreased precipitation, thus intensifying drought conditions [2,3]. Similarly, glacier melt, driven by rising temperatures, contributes to sea-level rise, which can further destabilize and accelerate the melting of coastal glaciers and ice sheets through increased erosion and thermal undermining [4]. However, little is known about the cascading pattern in health impacts from global environmental changes.

Based on indirect evidence, the impact of environmental and climate change on health manifests through such cascading effects. For instance, heatstroke compromises the body’s thermoregulatory capacity, making individuals more vulnerable to subsequent heat exposure and potentially leading to more severe outcomes [5,6]. Exposure to air pollution, resulting in respiratory illnesses, can also heighten an individual’s susceptibility to future air pollution episodes, leading to exacerbations and further decline in respiratory function [[7], [8], [9]]. While many cascades involve amplification, some initial health impacts might trigger responses that limit subsequent harm. For instance, low-level exposure to air pollutants might potentially prime the immune system, leading to a more rapid response upon subsequent exposure [10,11]. Furthermore, in response to elevated air pollution levels, individuals may adopt avoidance behaviors such as staying indoors or using air purifiers, thereby reducing their exposure and the potential for further health consequences, which is known as a component of human adaptation [12,13]. Therefore, understanding both the amplifying and shrinking cascade effects is crucial for building resilience and safeguarding both the environment and human well-being in the face of ongoing global change.

Although there is limited direct evidence of cascading health effects, a few clues can be found across different stressors and timescales, suggesting it is a fundamental aspect of environmental physiology. For instance, studies have shown that populations in warmer climates exhibit significantly lower mortality risk from heatwaves compared to those in cooler regions, demonstrating long-term physiological and behavioral adaptations to their local environment [14]. Notably, the duration of heatwaves has been found to modify their health effects on human beings [15,16]. This indicates that progressive exposure to warm temperatures throughout a summer induces a measurable degree of acclimatization, attenuating the health impacts of subsequent heat events. A parallel phenomenon exists for chemical stressors like ozone (O_3_). Controlled human exposure studies reveal that repeated encounters with ozone lead to an attenuated acute response. Subjects initially experiencing significant declines in lung function and airway inflammation following ozone exposure show a markedly weaker reaction upon subsequent exposures in the following days [17,18]. This demonstrates a rapid physiological tolerance, where the initial exposure alters the cellular response pathway. Together, these examples from climatological and toxicological research underscore a unified principle: the history of exposure dynamically recalibrates an organism’s vulnerability. Ignoring this temporal dimension leads to an inaccurate assessment of environmental health risks. Effective public health warning systems and climate change adaptation strategies must therefore evolve to incorporate dynamic, history-dependent models of susceptibility.

The lack of suitable statistical models is the key barrier to quantitatively examining the cascading health effects of environmental or climate exposure. In environmental epidemiology of acute exposure, a commonly used method is the distributed-lag model (DLM), which typically assumes the health effects of exposures at different lag times as additive [19,20]. However, this assumption overlooks the critical phenomenon of cascading effects, wherein prior exposure history can modify the health effects of a subsequent exposure. To deal with this issue, we propose a novel methodological extension that explicitly models these dynamics by incorporating interaction terms between exposures at different lags within the DLM framework. Such an approach holds significant promise for advancing scientific understanding, allowing researchers to directly quantify how past exposures may induce sensitization or adaptation, thereby amplifying or attenuating future health effects. This would revolutionize public health interventions by facilitating dynamic, history-aware risk alerts. However, moving beyond the traditional assumption presents substantial challenges, primarily severe multicollinearity among lagged exposure terms and the consequent demand for immense statistical power. Furthermore, interpreting the resulting high-dimensional interaction surfaces would be exceptionally complex. This study will also illustrate those challenges and point out possible solutions for future research.

The primary aim of this study is to introduce and validate a novel methodological framework, the Interactive DLM (iDLM), designed specifically to quantify history-dependent dynamics. In this paper, we first conduct simulation studies to validate that the iDLM, through structured regularization, can accurately recover the risk structure that traditional models fail to capture. We then provide a demonstrative application of iDLM to a nationwide U.S. dataset to illustrate its potential to generate new epidemiological insights.

## 2 Methods

### 2.1 Statistical model

The DLM is used to quantify the delayed effects of a time-series exposure (e.g., daily temperature) on a concurrent health outcome (e.g., daily mortality counts). Within a Generalized Linear Model (GLM) framework, its fundamental mathematical form can be expressed as:

(1) $$\begin{array}{c} g\left(E\left[y_{t}\right]\right)=\alpha +\sum_{\text{lag}=0}^{L}\beta_{\text{lag}}x_{t,\text{lag}}+Z_{t}\gamma +\theta \left(t\right) \end{array}$$

Where, $E\left[y_{t}\right]$ denotes the expected value of the outcome (e.g., daily deaths) observed at time *t*; α is the intercept term of the model; $x_{t,\text{lag}}$ represents the exposure level at a specific lag time for the $t^{th}$ outcome; $\beta_{\text{lag}}$ is the regression coefficient corresponding to the exposure at the lag time; L denotes the maximum lag time; $Z_{t}$ denotes a set of time-varying confounding variables and γ denotes a vector of their regression coefficients; θ(t) denotes the nonparametric temporal trends, usually including seasonality, long-term trends, and day-of-week effects. Alternatively, θ(t) denotes a fixed effect or stratum by temporal indicator, such as a day-of-week in a certain calendar month. The set of these coefficients $\left(\beta_{0},\beta_{1},\ldots ,\beta_{L}\right)$ forms the distributed-lag effects. The key assumption of this model is additivity: the contributions from exposures at different lags are independent and sum to produce the total effect. The model does not account for interactions between them.

To capture cascading effects—the modulation of health impacts by prior exposures—the standard DLM is extended by introducing interaction terms between exposures at different lags. This forms the iDLM. Its mathematical formula can be written as:

(2) $$\begin{array}{c} g\left(E\left[y_{t}\right]\right)=\alpha +\sum_{\text{lag}=0}^{L}\beta_{\text{lag}}x_{t,\text{lag}}+\sum_{d=1}^{D}\sum_{\text{lag}=0}^{L-D}b_{\text{lag},d}\left(x_{t,\text{lag}}x_{t,\text{lag}+d}\right)+Z_{t}\gamma +\theta \left(t\right) \end{array}$$

Compared to the standard DLM, this model adds a crucial component: $x_{t,\text{lag}}x_{t,\text{lag}+d}$ is the interaction term between two exposure terms separated by a distance of d units. $b_{\text{lag},d}$ is the coefficient for this interaction term. This coefficient directly quantifies the cascading effect. If $b_{\text{lag},d}>0$, it indicates a positive interaction or amplification effect, suggesting that exposure at one point in time sensitizes an individual to the harm from exposure at another time. If $b_{\text{lag},d}<0$, it indicates a negative interaction or adaptation effect, suggesting that prior exposure induces tolerance, thereby dampening the harm from subsequent exposure. If $b_{\text{lag},d}=0$, there is no interaction between exposures at these two lags, and the model simplifies to the standard DLM for this pair.

By estimating the matrix of b coefficients, the iDLM moves beyond the simple assumption of linear additivity, allowing for the simultaneous assessment of the main effects of exposure (captured by the β coefficients) and their self-modulating effects over time (captured by the b coefficients) within a single, unified framework.

### 2.2 Structured splines

In Eq. 2, there is severe multicollinearity in estimating numerous individual lag and interaction coefficients. The iDLM addresses this issue by using splines for structured dimensionality reduction, just like the well-developed DLM. Instead of estimating a separate coefficient for each term in the full model (2), we impose smoothness constraints by re-parameterizing the coefficients as linear combinations of a small number of basis functions. For the main effects, the lag-specific coefficients ($\beta_{1},\ldots ,\beta_{L}$) are assumed to lie on a smooth curve defined by a one-dimensional spline basis, $s_{m}\left(\cdot \right)$.

(3) $$\begin{array}{c} \beta \left(\text{lag}\right)=\sum_{m=1}^{M}\eta_{m}s_{m}\left(\text{lag}\right) \end{array}$$

Here, the model estimates the M basis coefficients (η) instead of the L+1 highly correlated $\beta_{\text{lag}}$ coefficients, where M<L+1. Similarly, for the interaction effects, the coefficients $b_{\text{lag},d}$ are modeled as a smooth two-dimensional surface. This is parameterized as a function of the primary lag and the lag interval d, using a tensor product spline basis or a two-dimensional spline:

(4) $$\begin{array}{c} b\left(\text{lag},d\right)=\sum_{p=1}^{P}\sum_{q=1}^{Q}\theta_{pq}\pi_{p}\left(\text{lag}\right)\psi_{q}\left(d\right)\; \end{array}$$

Where, $\pi_{p}\left(\cdot \right)$ and $\psi_{q}\left(d\right)$ are two sets of basis functions. This reduces the vast number of $b_{\text{lag},d}$ parameters to a much smaller, more stable set of P×Q basis coefficients (θ). By substituting these spline representations back into the iDLM, the model’s complexity is drastically reduced. It estimates the stable basis coefficients (θ,η)—which are fewer in number—rather than the numerous, collinear original parameters, thus effectively controlling for multicollinearity through structured regularization.

### 2.3 Simulation analyses

To validate the iDLM method, we implement a Monte Carlo simulation: First, we generate synthetic time-series data where the health outcome at time t is drawn from a Poisson distribution. The mean is constructed from a known ground-truth model incorporating both main effects and a pre-specified cascading interaction structure. The main effect (β) and the interaction (b) are defined by deterministic functions. The lag-response function is expected to decay and thus can be specified as follows:

(5) $$\begin{array}{c} \beta \left(\text{lag}\right)=c_{0}\cdot \;\exp\left(-c_{1}\text{lag}\right) \end{array}$$

where $c_{0}$ and $c_{1}$ are positive constants to control the curvature. For the cascading effect, the interaction can be specified as follows:

(6) $$\begin{array}{c} b\left(\text{lag},d\right)=c_{0}\cdot \;\exp\left(-c_{1}\text{lag}-c_{2}d\right) \end{array}$$

where $c_{1}$ and $c_{2}$ are positive constants to control corresponding decaying rates with lag between exposure and outcome and interval between the two lags (d), respectively. If $c_{0}$ is a positive constant, Eq. 6 represents a synergistic effect, showing how lagged exposures increase susceptibility; if negative, Eq. 6 represents a negative feedback loop potentially reflecting processes such as human adaptation. Subsequently, we fit two models to this data: one for the positive cascading effect, and the other for the negative. The proposed iDLM attempts to recover the full generative structure by estimating parameters pre-determined by Eqs. 5 and 6. As a comparison, a traditional DLM is fitted, but it is mis-specified as it omits the interaction terms.By iterating this process 1000 times, the simulation evaluates the performance of the iDLM by comparing its estimated parameters (β,b) to the true parameters generated by Eqs. 5 and 6), thereby assessing its ability to accurately recover the underlying cascading effects and examining the bias in the traditional DLM estimates when such interactions are ignored. The detailed settings of data simulation and statistical analysis are illustrated in Supplementary Methods.

### 2.4 Demonstrative data and analysis

To illustrate the usage of the iDLM method, we propose a nationwide analysis to directly quantify the cascading health effects of major air pollutants by applying it to a uniquely detailed historical dataset of mortality and environmental exposures in the U.S. Conventional models, such as DLM, linking air pollution to mortality have treated daily exposures as independent insults, assuming their effects are simply additive over time. This study introduces and examines the following additional assumption: prior exposures can induce adaptation or sensitization, thereby altering vulnerability to subsequent events.

Our study leverages a comprehensive daily time-series dataset constructed for all U.S. counties from 1972 to 1988. This historical period was specifically chosen because public-use mortality records from the National Center for Health Statistics (NCHS; available at cdc.gov/nchs/nvss) provide the exact date of death at the county level, a level of spatio-temporal resolution unavailable in more recent public data. This allows for a precise alignment of health outcomes with daily environmental exposures.

To construct daily pollution time series for this historical period, we integrated several data streams. For PM_2.5_, we used concentrations from the historical reconstruction by Hao et al. in the Northern Hemisphere from 1959 to 2022 [21]. This dataset was generated using a machine learning approach that estimates daily PM_2.5_ from near-surface visibility and meteorological variables; it demonstrated high accuracy, with a cross-validation R^2^ of 0.80 and a root-mean-square error (RMSE) of 13.5 μg/m^3^. For ground-level O_3_, we used the daily maximum 8-h moving average from the U.S. Environmental Protection Agency’s Air Quality System (AQS; epa.gov/aqs). To control confounding by weather, daily mean temperature and temperature variability were obtained from the high-resolution ERA5-Land reanalysis dataset (from the Copernicus Climate Change Service). All datasets were aggregated at the daily-county level. Due to different spatiotemporal coverages, we prepared two datasets: one containing 581 geographic units from 1980 to 1988 for O_3_, and the other containing 274 geographic units from 1972 to 1988. Geographic units were specified by the NCHS code at the county level. For New York City, the NCHS assigned a uniform code for all the counties. For each county, the temporal coverage was differentially determined by the availability of PM_2.5_/O_3_ data, as documented in Table S1. For all analyses, we used a complete-case approach. The PM_2.5_ exposure dataset, being a model-based reconstruction, was spatially and temporally complete for our study period. For the O_3_ analysis, any day in a given county with missing data for mortality, O_3_, or meteorological variables was excluded from that county’s time series. We applied the iDLM to this dataset to assess the effects of PM_2.5_ or O_3_ on all-cause mortality by geographic unit and pooled the unit-specific estimates together in a second-stage meta-analysis using the R package mixmeta [22]. For the first-stage analysis, we fitted a conditional Poisson regression model, equivalent to a time-stratified case-crossover design, which controls for all time-invariant unobserved confounders. For the first time, the model focusing on the cascading effect of air pollution directly tested our core hypothesis: a positive b would provide evidence of synergistic amplification, whereas a negative coefficient would signify an adaptation. The specific details of all model parameters, including the degrees of freedom and the number of knots for the iDLM cross-basis, are provided in the Supplementary Methods.

## 3 Results

### 3.1 Statistical simulation

**Fig. 1:**
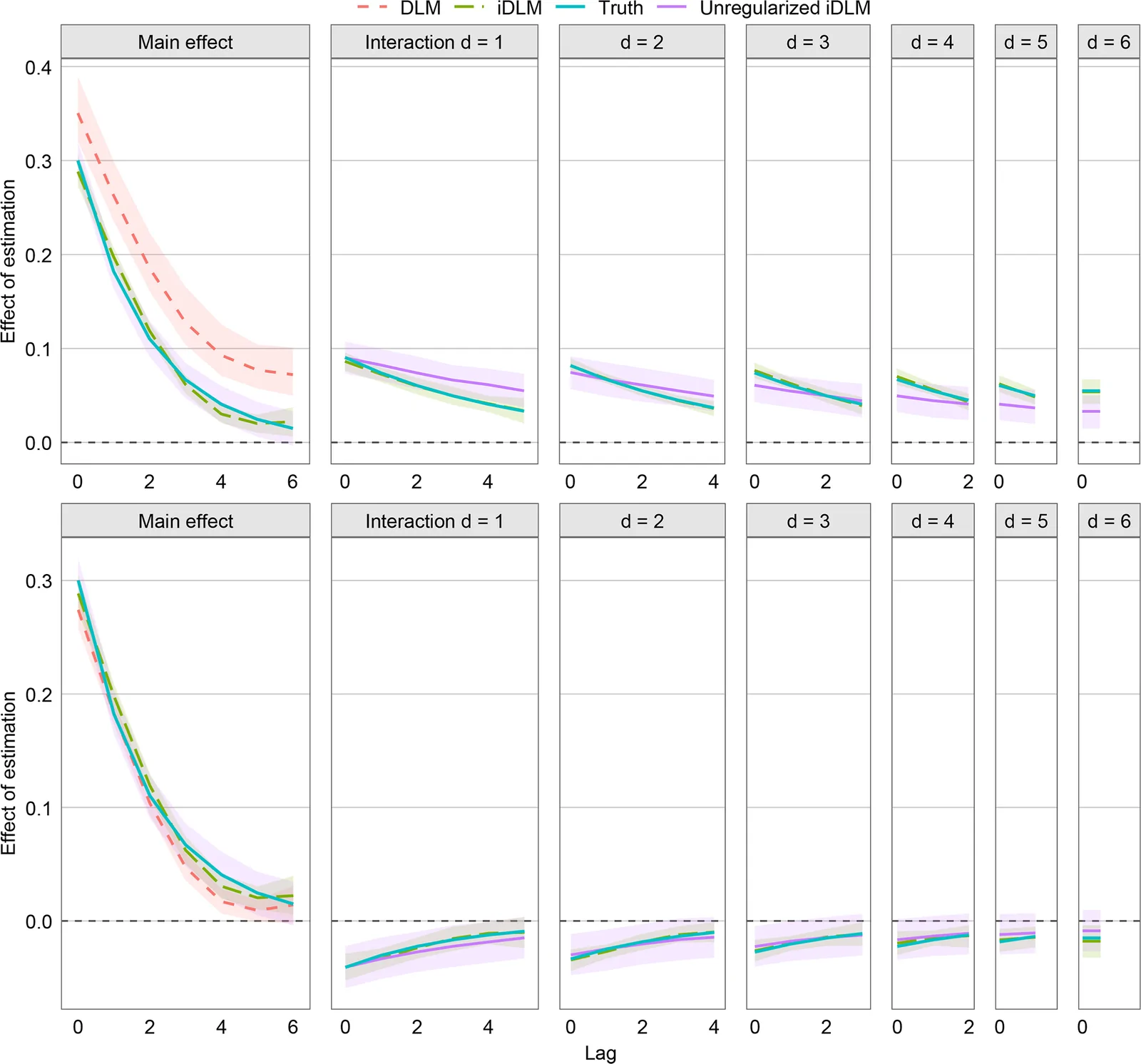
Simulated performance of different statistical models in estimating cascading effects. The upper panel shows a positive cascading effect, and the lower shows a negative one.

Our simulation results compellingly demonstrate the methodological limitations of standard DLM and highlight the advantages of the proposed iDLM framework when cascading effects are present (Fig. 1). In scenarios with underlying synergistic amplification (upper panel), the standard DLM (red line), which is mis-specified by its assumption of additivity, gets the wrong answer. It exhibits substantial upward bias, severely overestimating the lag-distributed main effects by misattributing the additional risk from positive interactions to them. Conversely, under adaptation scenarios (lower panel), its estimates of the main effect exhibit a downward bias. While an unregularized iDLM (purple line) can, on average, produce unbiased estimates due to its correct model specification, it suffers from high variance, yielding unstable estimates with wide confidence intervals. This instability becomes particularly problematic when interaction effects are weak or data are sparse. In contrast, our proposed regularized iDLM (green line) resolves this trade-off. By employing penalized splines to impose a smoothness constraint, it achieves low bias by correctly modeling the interaction structure while maintaining low variance. This allows it to produce precise and stable estimates that closely track the ground truth (blue line) for both the main and cascading effects across both amplification and adaptation scenarios. This simulation confirms that without a structured, regularized approach to modeling interactions, traditional methods provide a biased and incomplete picture of the true risk structure.

### 3.2 Demonstrative analyses

**Fig. 2:**
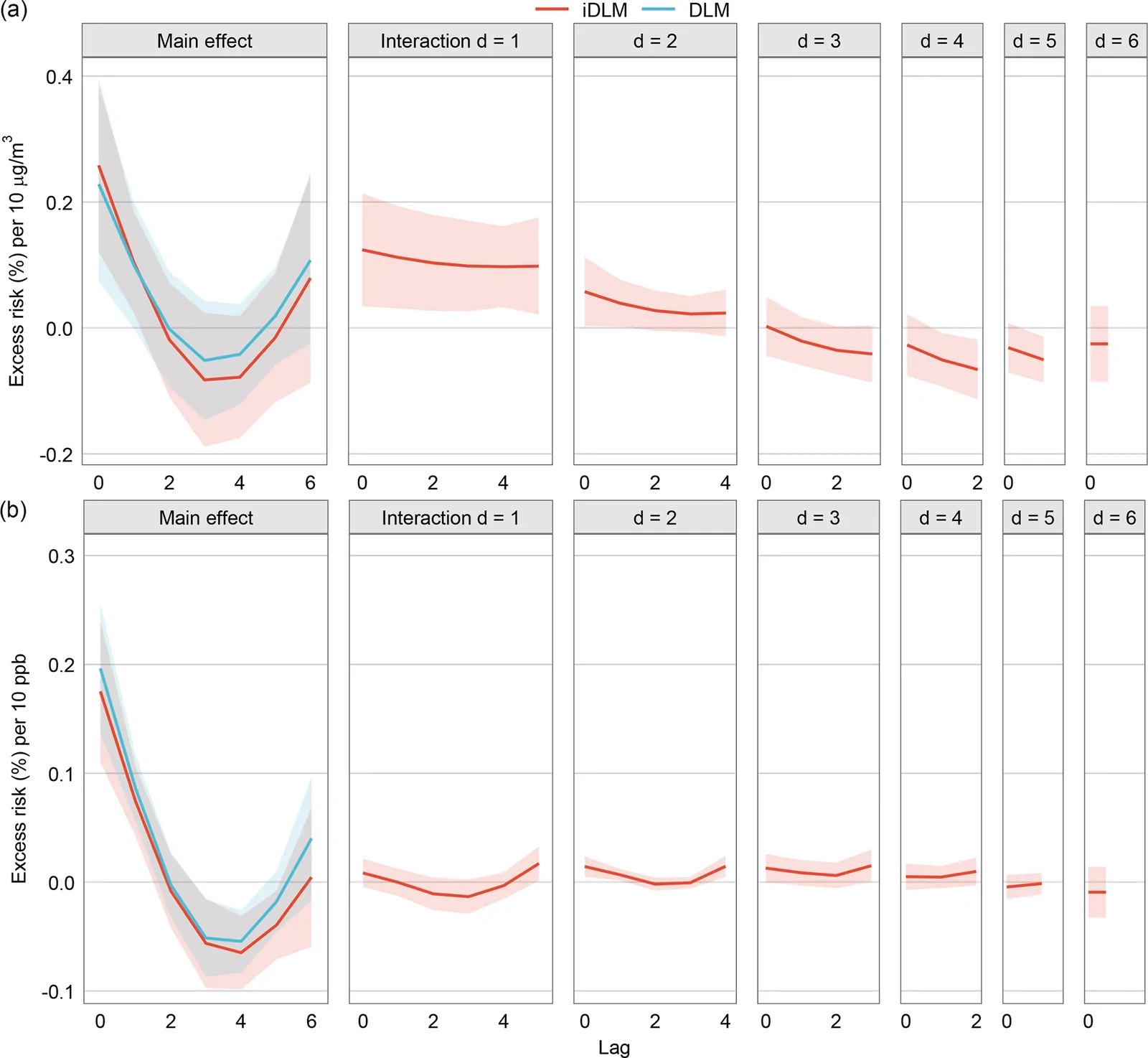
Lag-distributed main and cascading main effects of PM_2.5_ (a) or O_3_ (b) on all-cause mortality in the U.S. The left panel for each pollutant shows the main lag-response effect estimated by a traditional distributed-lag model (DLM, blue) and the interactive DLM (iDLM, red). The subsequent panels show the cascading effect estimated by the interaction terms of the iDLM. The cascading effects are plotted against the primary lag for different intervals (day) between exposures. Shaded areas represent 95% confidence intervals. For each county, an iDLM or DLM is applied to the corresponding time series separately. The county-specific estimates are pooled together, as presented here.

Application of the iDLM to the nationwide U.S. dataset reveals distinct cascading effect patterns for PM_2.5_ and O_3_, providing novel insights into the dynamic nature of air pollution’s health burden. While the main effects appear similar across pollutants, their interactive effects—which are invisible to standard models—diverge significantly, suggesting different underlying physiological pathways of harm. A detailed description of the analyzed datasets is documented in Table S1. For both PM_2.5_ (Fig. 2a) and O_3_ (Fig. 2b), the main lagged effects on all-cause mortality, as estimated by both the iDLM and the traditional DLM, show a consistent pattern. The estimated effect is highest on the day of exposure (lag 0) and decreases over the subsequent days. For each 10-μg/m^3^ increment in PM_2.5_ and 10-ppb increase in O_3_ on the day of exposure, the risk of all-cause mortality increased by 0.26% (95% confidence interval [CI]: 0.12, 0.40) or 0.18% (95% CI: 0.11, 0.24), respectively. Both pollutants exhibited a slight harvesting effect, indicated by a negative effect around lags 3–4, which was particularly pronounced for O_3_. The similarity in the main effect curves estimated by both models suggests that the primary, immediate risk component is robustly captured by either method.

However, the iDLM uncovers critical differences in their cascading effects. While the main effects appear similar between the models, the right panels of Fig. 2a and b reveal the hidden story of cascading effects, which can be interpreted as follows. For PM_2.5_ (Fig. 2a), the consistently positive red bands in the “d = 1” and “d = 2” panels represent a synergistic amplification. This means that the harm from today’s PM_2.5_ exposure is significantly increased if there was also high exposure yesterday (d = 1) or the day before (d = 2). This pattern suggests that initial exposure “primes” the body for greater injury from subsequent exposures. This amplification diminishes as the lag (time since event) and the interval between exposures increase. Conversely, at longer lag and interval times (e.g., d = 4 or 5), the interaction terms trend towards zero or slightly negative, hinting at a potential long-term adaptive response or the depletion of susceptible individuals. In contrast, for O_3_ (Fig. 2b), these interaction bands are much flatter and closer to zero. This indicates a substantially weaker or non-existent amplification effect. While some positive interaction is present, particularly for exposures two days apart (d = 2), the overall magnitude is smaller than for PM_2.5_, and the effects do not show a clear decay pattern with increasing lag or interval. These divergent patterns, which were confirmed by a formal multivariate likelihood ratio test (*p* < 0.01), underscore the importance of modeling such interactions to avoid a biased understanding of risk and to accurately characterize the distinct hazard profiles of different pollutants. Furthermore, a formal model comparison using a pooled likelihood ratio test confirmed the superior fit of the iDLM, indicating that the inclusion of cascading interaction terms is statistically justified for both PM_2.5_ (*p* = 0.018) and O_3_ (*p* < 0.001).

### 3.3 Public health implications

**Fig. 3:**
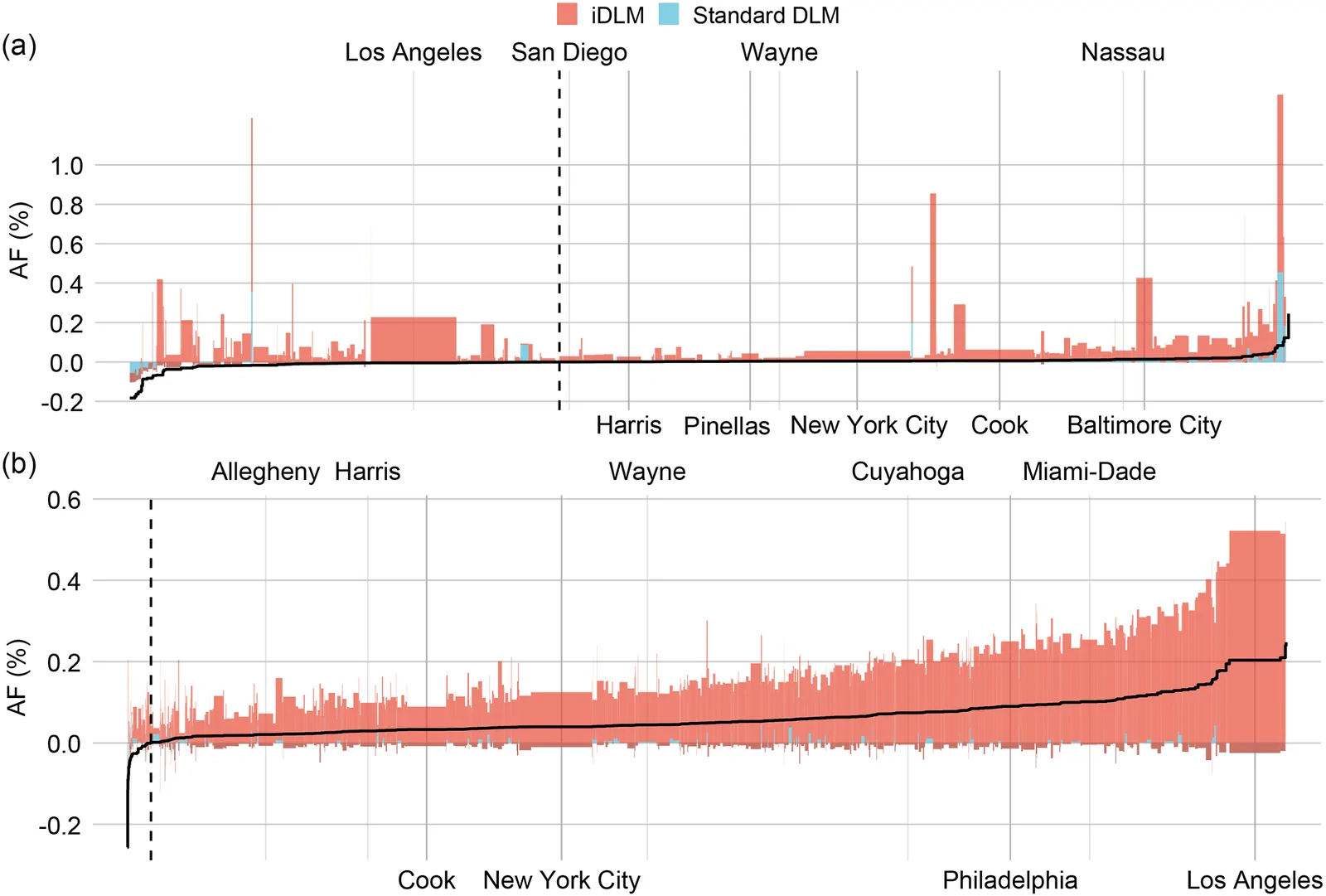
Comparison of mortality burden estimated by the iDLM and a standard DLM. County-specific attributable fraction (AF, %) of all-cause mortality for PM_2_._5_ (a), and O_3_ (b). Each vertical bar represents a county. For each pollutant, the red bar represents the total AF estimated by the iDLM. For comparison, the blue bar shows the AF estimated by a standard DLM for the same county. The solid black line indicates the lower 95% confidence limit of the total AF; counties to the right of the vertical dashed line have a statistically significant total AF > 0.

Our analysis (Fig. 3 and Table S2) reveals that the mortality burden attributable to short-term exposure to PM_2.5_ or O_3_ differs due to the settings of the model. Critically, the total attributable fraction (AF) estimated by the iDLM is, in most counties, significantly larger than the AF estimated by a traditional DLM that only considers additive effects. This gap highlights the crucial contribution of dynamic, history-dependent cascading interactions in iDLM, a component of risk structurally invisible to conventional models. Fig. 3 displays the county-specific AF of mortality estimated by both the iDLM (red) and a standard DLM (blue). Specifically, for PM_2.5_ (Fig. 3a), a striking pattern emerges: the synergistic amplification of risk from repeated exposures is the primary driver of PM_2.5_-related mortality. For major metropolitan areas such as Los Angeles County (LA), Baltimore City, New York City (NY); and Harris County (center of Houston metropolitan area), the overall attributable fractions estimated by iDLM were 0.227% (95% CI: −0.004, 0.466), 0.427% (95% CI: 0.014, 0.856), 0.056% (95% CI: 0.005, 0.109), 0.027% (95% CI: 0.001, 0.055), respectively. In stark contrast, the AFs estimated by the standard DLM for these same areas are substantially smaller and often not statistically significant. Quantitatively, relying on the traditional additive model would mean overlooking approximately 98.4%, 94.6%, 94.8%, and 94.6% of the total mortality burden identified by the iDLM in these respective counties.

A similar, even less heterogeneous, pattern is observed for O_3_ (Fig. 3b). Again, the mortality burden attributable to cascading effects surpasses that of the standard DLM in most counties. For the aforementioned counties, results from iDLM show that short-term O_3_ exposure contributes to 0.522% (0.204, 0.846), 0.312% (0.126, 0.499), 0.125% (0.040, 0.210), and 0.099% (0.029, 0.169) of all-cause mortality, respectively. However, a standard DLM typically captured only a negligible fraction of the total mortality burden or sometimes yielded non-significant effect estimates. While the overall attribution for O_3_ is generally lower than for PM_2.5_, the conclusion remains the same: ignoring the interactive effects leads to a gross underestimation of the true public health impact. These findings fundamentally challenge the current paradigm of risk assessment, demonstrating that the dynamic, modulatory effects of exposure history are not a minor nuance but rather appear to be a major contributor to the mortality burden of ambient air pollution.

## 4 Discussions

In this study, we demonstrate that the mortality risk from short-term air pollution exposure is not a simple summation of independent daily insults but is dynamically shaped by the history of prior exposures through cascading effects. By developing and applying a novel iDLM, we provide the first quantitative, nationwide evidence that repeated exposures to PM_2.5_ will lead to a strong, synergistic amplification of mortality risk, a pattern that is substantially weaker and less structured for O_3_. Most strikingly, our analysis reveals that these history-dependent cascading effects constitute a substantial portion of the total mortality burden, one that dwarfs the contribution captured by standard additive models alone.

Our main additive effect estimates align with landmark time-series studies like the National Morbidity, Mortality, and Air Pollution Study (NMMAPS), which analyzed data from a similar historical period. It is estimated that a 10 μg/m^3^ increase in PM_2.5_ was associated with an increase of 0.25%–0.72% in total mortality risk [23]. Our study finds a comparable immediate effect for PM_2.5_ of 0.26% at lag 0. For O_3_, NMMAPS reported a 0.25% increase in mortality per 10 ppb increase, which aligns with the immediate risk profile we identified. However, our primary contribution lies beyond these additive effects. The NMMAPS analysis, like virtually all time-series studies of its era, was based on a standard DLM that, by design, assumes the health effects of daily exposures are additive and independent. It is also important to interpret the uncertainty associated with these estimated effects. As shown in Fig. 2, the confidence intervals for the PM_2.5_ interaction terms are narrowest for short lags and intervals (e.g., d = 1, 2), where the synergistic effect is strongest and most statistically significant. As the lag and interval increase, the confidence intervals tend to widen, reflecting greater uncertainty in regions where the data may be sparser or the true effect is closer to null. Nevertheless, the positive and significant amplification effect in the immediate short term remains a robust finding even after accounting for this uncertainty, underscoring the stability of our central conclusion. By explicitly modeling the interactions between lagged exposures, we find that the total mortality burden estimated by iDLM is substantially larger than that captured by the additive main effects alone. This suggests that a significant component of risk was methodologically invisible to previous models. However, we need to clarify that our application of the iDLM in the demonstrative analysis should be seen not as a definitive epidemiological re-analysis, but as a powerful illustration of how this novel framework can uncover potentially crucial, history-dependent risk dynamics that traditional approaches cannot.

The cascading mortality patterns observed for air pollutants are biologically plausible due to their toxicological mechanisms and the body’s subsequent physiological responses. The strong, synergistic amplification seen with PM_2.5_ can be explained by a multi-systemic, self-reinforcing cycle of injury. Initial exposure to fine particulate matter triggers pronounced pulmonary oxidative stress and inflammation [24,25]. PM_2.5_ components can then translocate into the bloodstream, directly impairing vascular endothelial function and promoting a pro-thrombotic state [[26], [27], [28]]. This initial insult compromises cardiovascular homeostasis and depletes the body’s antioxidant capacity. A subsequent exposure on the following day encounters a system already primed for injury, leading to a heightened risk of acute cardiovascular events like myocardial infarction or stroke [[29], [30], [31]]. The weaker and less structured cascading effect of O_3_ may relate to its primary mode of action as a highly reactive gaseous oxidant. The effects of O_3_ are most potent at the epithelial surfaces of the respiratory tract, where it causes immediate cellular damage and inflammation [18,32]. However, repeated O_3_ exposure is known to induce a tolerance or adaptation phenomenon, where subsequent exposures elicit a diminished inflammatory response in the lungs [17,33]. This adaptive mechanism could counteract a potential amplification effect, resulting in a weaker, more ambiguous cascading pattern. Nevertheless, these connections are based on synthesizing evidence from different lines of research. Future dedicated toxicological and clinical studies, designed specifically to test the “repeated exposure” hypothesis, are needed to definitively confirm and elucidate the cellular and systemic mechanisms behind the cascading effects observed at the population level. The utility of the iDLM framework extends far beyond air pollution, offering a new paradigm for quantifying the health effects of any environmental stressor where risk is dependent on the temporal pattern of exposure. For instance, the concept of a heatwave’s duration effect can be mechanistically modeled as a cascading process. Instead of treating duration as a simple effect modifier, the iDLM can directly test the hypothesis that the health risk on the third day of a heatwave is amplified by the accumulated physiological stress from the preceding two days, a phenomenon quantifiable through positive interaction coefficients [34]. Similarly, the iDLM can elucidate the risks associated with temperature volatility. Stable high temperatures may allow for physiological adaptation (a negative, protective cascade), but this process can be disrupted by volatile temperature swings [6,35]. Furthermore, this framework may help resolve long-standing paradoxes in environmental epidemiology, such as the persistent discrepancy between the risk estimates for short-term and long-term exposure. Cohort studies consistently find that the mortality risk from long-term PM_2.5_ exposure is substantially larger than the acute risk estimated by time-series studies [[36], [37], [38], [39]]. The iDLM provides a mechanistic bridge to address this gap: the cumulative risk from long-term exposure is the accumulation of daily risks plus the compounding, synergistic amplification from the cascading effects of repeated exposures. In essence, any field where repeated exposures are common and outcomes are history-dependent—from the health impacts of intermittent wildfire smoke to the efficacy of repeated therapeutic drug doses in pharmacoepidemiology—stands to benefit from this modeling approach [[40], [41], [42]].

To clarify the novelty of the iDLM, it is essential to position it within the landscape of existing methods. While standard Distributed Lag (Non-linear) Models (DLM/DLNMs) flexibly capture the direct exposure-response relationship over time, they are built on a foundational assumption of additivity. The iDLM fundamentally relaxes this assumption by modeling exposure-exposure interactions and quantifying how the risk landscape itself is dynamically shaped by exposure history. Simpler non-additive approaches, such as using a moving average of past exposures as an effect modifier, impose a rigid, equal-weighting structure that can obscure the temporal dynamics of vulnerability. In contrast, the iDLM’s flexible surface allows the data to reveal these patterns without strong a priori constraints. While prior studies have occasionally included specific interaction terms, they have typically done so in an unregularized framework prone to instability. The core innovation of the iDLM is its use of structured, penalized splines to regularize the entire high-dimensional interaction surface at once, enabling a robust and comprehensive estimation of history-dependent risk dynamics.

While this study introduces a new framework to model the health effects of short-term environmental exposure, several limitations highlight avenues for future research. First, our current iDLM assumes linear interactions and relies on pre-specified spline functions to model lag and interaction structures; we acknowledge that the true biological responses may be more complex and non-linear. For example, the cascading effect of prior exposure might only occur after a certain threshold of exposure is crossed. Second, a deeper exploration of how iDLM represents the “vulnerability state” of the population is warranted. The current study does not explicitly assess the divergence of health impacts from different exposure patterns, such as a sharp, high-exposure spike versus a period of moderate exposure. Future work using targeted simulations or diverse real-world data (e.g., intermittent wildfire smoke vs. chronic traffic pollution) is needed to formally explore the full spectrum of history-dependent vulnerability. Third, by including all pairwise lagged interactions, the model faces a high-dimensional challenge that demands significant statistical power and computational resources. However, owing to the structured interaction terms and regularization configuration, the computational demand remains manageable. Fourth, like all observational studies, our demonstrative analysis is susceptible to potential residual confounding, which presents a core identification challenge. Our interpretation of the cascading effects relies on the conditional independence assumption: after controlling for time trend and measured covariates, daily pollution variation is as-good-as-random with respect to unobserved factors affecting mortality. While our use of a conditional Poisson model inherently controls for time-invariant confounders, the model might not fully capture the influence of non-stationary confounders, e.g., influenza outbreaks. Fifth, our model does not explicitly disentangle physiological adaptation from behavioral adaptation. The “adaptive cascades” captured by negative interaction terms represent the net effect of all processes that reduce risk following an initial exposure. This could be a true physiological tolerance (as seen with ozone) or a behavioral feedback loop, where individuals respond to a high-pollution event by reducing their exposure in subsequent days. Finally, the findings of our nationwide analysis are intended as a methodological demonstration, and their generalizability is subject to the limitations of historical data. We chose the 1972–1988 period specifically because of the unique availability of daily, county-level mortality records, which were essential for illustrating the iDLM’s capabilities. However, it is crucial to recognize that both environmental conditions and population characteristics of that era differ from those of today. Therefore, while the patterns are illustrative, the specific risk estimates may not be directly transportable to contemporary settings. Future methodological development should focus on overcoming these limitations. The immediate next step is to extend the iDLM to a non-linear framework capable of modeling non-linear exposure-response relationships for both main and interactive effects. To address the high-dimensionality problem, future work should integrate sophisticated interaction-screening techniques. Instead of a priori including all interaction terms, methods inspired by hierarchical regularization, such as the *glinternet* algorithm, could be adopted to penalize and select the most relevant interaction terms [43,44]. To bolster causal claims, researchers could apply the iDLM framework within quasi-experimental settings in the future to quantify how strong an unmeasured confounder would need to be to negate the observed effects. Furthermore, applying the iDLM framework to modern, high-quality datasets from recent decades—with more accurate exposure—would be essential to test whether the specific patterns of cascading effects observed here are persistent. Such analyses, aiming for definitive epidemiological conclusions, would necessitate a level of methodological rigor beyond the scope of our current demonstrative study. This includes: first, formal justification and sensitivity analysis for the choice of spline configurations, the maximum lag, and the interval windows. Second, a rigorous statistical treatment of daily mortality data is essential, including fitting a quasi-Poisson model to address overdispersion and performing formal diagnostics to ensure the absence of residual autocorrelation. Only through such comprehensive applications can iDLM be transformed from a validated methodological concept to a robust tool for modern environmental risk assessment, providing more actionable insights for public health policy. These advancements would enhance the model’s statistical robustness, computational efficiency, and interpretability.

Our findings lay the groundwork for a new generation of “history-aware” public health warning systems. Current systems based on static concentration thresholds are largely amnesic, meaning they evaluate the risk of a given day in isolation, without considering the recently accumulated vulnerability. An iDLM-based system, in contrast, could issue dynamic, risk-based alerts that account for the population’s exposure history. To offer a concrete example, consider a day with a moderate predicted PM_2.5_ level. If this day follows a period of clean air, a standard alert focused on sensitive groups might be sufficient. However, if this identical prediction follows a sustained pollution episode, the iDLM would recognize the heightened population vulnerability and could trigger an upgraded, broader public health warning, advising all individuals to reduce outdoor exertion and schools to consider canceling outdoor activities. This shift from a concentration-based to a vulnerability-based paradigm would allow for more targeted and efficient public health interventions, mitigating risks before they escalate and reducing the potential for ineffective alerts on days when the history-adjusted risk is low.

## 5 Conclusion

In this study, we developed and validated the iDLM, a new framework to quantify the cascading health effects of environmental exposures. Through a nationwide analysis of air pollution and mortality in the U.S., we demonstrate that the health risks from PM_2.5_ and O_3_ appear to be dynamically modulated by exposure history. We establish two central findings: first, PM_2.5_ exposure leads to a strong, synergistic amplification of mortality risk, a pattern distinct from the weaker interactions of O_3_. Second, most importantly, these cascading effects are not a minor component of risk but constitute a substantial proportion of the total mortality burden, far outweighing the contribution from the direct additive effects captured by conventional models. Our work challenges the foundational assumption of additivity in environmental risk assessment and shows that a dynamic, history-dependent view is essential for accurately understanding risks caused by environmental change. By revealing these hidden risk dynamics, our approach provides a new tool for developing precise public health strategies, such as building a history-aware warning system.

## CRediT authorship contribution statement

**Jinting Guo:** Writing – original draft, Formal analysis, Conceptualization. **Hengyi Liu:** Writing – review & editing, Writing – original draft, Formal analysis. **Meng Wang:** Validation, Supervision. **Mingkun Tong:** Resources, Data curation. **Jianyu Deng:** Data curation. **Ning Kang:** Data curation. **Tianjia Guan:** Supervision. **Tao Xue:** Writing – original draft, Methodology, Funding acquisition, Conceptualization. **Tong Zhu:** Supervision.

## Data availability statement

The R code is available at https://github.com/JTGuo-i/iDLM.

## Declaration of competing interest

All authors declare that they have no conflict of interest:
