# Supplementary material for: Modelling beyond additive risks: A framework for estimating the cascading burden of environmental exposure
Source: Eco Environ Health. 2026 Sep 1;5(4):100281. doi: 10.1016/j.eehl.2026.100281 (PMC13587907; doi:10.1016/j.eehl.2026.100281)
Supplement: Supplementary file 1 [file mmc1.docx]

**Supplementary Methods**

**S1. Model Validation via Monte Carlo Simulation**

To validate the proposed Interactive Distributed Lag Model (iDLM) and assess its performance relative to a standard Distributed Lag Model (DLM), we conducted a comprehensive Monte Carlo simulation study. The simulation was designed to generate synthetic time-series data with a known ground-truth risk structure, including both main and cascading effects. We then evaluated the ability of both the iDLM and a mis-specified standard DLM to recover these true underlying parameters. The process was iterated 1,000 times for each scenario to ensure robust evaluation.

**Data Generation Process**

For each of the 1,000 iterations, a synthetic dataset was generated following a pre-specified process:

1. Time series: A time-series of 10,000 observations was generated to ensure sufficient statistical power.
2. Exposure simulation: The exposure variable **x** was simulated as a vector of independent and identically distributed random variables from a standard normal distribution. Lagged exposure terms up to a maximum lag of 6 were then created from this series.
3. Outcome generation: The daily outcome **y** for each observation was generate from a Poisson distribution with a mean **λ** defined by the following model:

$$\log\left( \lambda\right)=0.1+\text{Σ}\left[ x_{t}-lag*\beta\left( lag \right) \right]+\text{ΣΣ}\left[ x_{t}-lag*x_{t}-\left( lag+d \right)*\gamma\left( lag,d \right) \right]$$

where **β(lag)** is the true main effect function, and **γ(lag, d)** is the true cascading interaction effect function.

4. Specification of true effect: The true lag-response for the main effect **β(lag)** was defined as an exponential decay function:

$$\beta\left( lag \right)=0.3*\exp\left( -0.5*lag \right)$$

This function specifies a strong immediate effect that diminishes over subsequent lags.

We simulated two distinct scenarios to represent different types of cascading effects **γ(lag, d)**:

$$\gamma\left( lag,d \right)=0.1*\exp\left( -0.2*lag-0.1*d \right)$$

$$\gamma\left( lag,d \right)=-0.05*\exp\left( -0.3*lag-0.2*d \right)$$

The first positive, decaying function of **lag** and **d** represents a scenario where prior exposure sensitizes an individual, but this sensitization effect weakens as the exposures become more distant in time. And the second function simulates a tolerance-inducing effect that diminishes over time.

**Model Fitting and Comparsion**

In each simulation iteration, the following three models were fitted to the generated data:

**Proposed iDLM**: Our primary model, designed to capture both main and interaction effects using penalized spline for regularization. It was specified with the following settings:

1. The model was fitted using a standard Poisson regression without any covariates in this simulation context. This setup was chosen to isolate the model's ability to estimate the exposure-response relationship itself.
2. The parameters were chosen as follows: Maximum lag was set to 6. Maximum interval was set to 6. The lag dimension for the main effects was modeled using a spline basis with k = 3 basis functions. The two-dimensional interaction surface **(lag, d)** was modeled using a penalized spline basis with k = 6 basis functions, generated via the mgcv package.

**Standard DLM**: A traditional DLM that incorrectly assumes additivity by omitting the interaction terms. The spline basis for the main effects with k = 3 basis functions, consistent with iDLM and the interaction terms were removed.

**Unregularized GLM:** A saturated generalized linear model with poisson family was fitted, including each main lag term and each valid pairwise interaction term (e.g., x_lag0_*x_lag1_, x_lag0_*x_lag2_, etc.) as independent linear predictors without any smoothing or regularization.

The estimated coefficients for the main and interaction effects from all three models were stored for each iteration. The performance was assessed by comparing the mean of the estimated coefficients across the 1,000 iterations to the known ground-truth values. This process allowed us to quantify the bias in the standard DLM and evaluate the ability of the iDLM to accurately and precisely recover the true, complex risk structure in a controlled setting.

**S2. Demonstrative Analysis**

To illustrate the application and utility of the iDLM framework, we conducted a nationwide analysis of the short-term effects of PM2.5 and O3 on all-cause mortality in the United States. The analysis followed a two-stage approach: in the first stage, we fitted county-specific time-series models; in the second stage, we pooled the county-specific estimates using a random-effects meta-analysis.

**First-Stage: County-Specific Time-Series Model**

For each county, we fitted a conditional Poisson regression model using the *gnm* function. This approach is equivalent to a time-stratified case-crossover design, which provides robust control for unobserved confounding factors that are stable over short time periods. To control seasonality, long-term trends, and day-of-week effects, we defined strata by creating a factor variable for each unique combination of year, month, and day of the week (e.g., "1980-January-Monday"). The *gnm* model was fitted conditioning the likelihood on the total number of deaths within each stratum. This design inherently controls any confounders that are constant within these short strata.

We further controlled for time-varying weather effects by including cross-basis terms for daily temperature, created using the *dlnm* package. Daily mean temperature was modeled with a cross-basis using a natural cubic spline with 3 degrees of freedom for the exposure-response dimension and a natural cubic spline with 3 degrees of freedom for the lag dimension, covering lags up to 6 days. Standard deviation of daily temperature was modeled with a cross-basis using a linear function for the exposure-response and a natural cubic spline with 3 degrees of freedom for the lag dimension (up to 6 days), with logarithmically spaced knots.

Within the time-stratified model for each county, we specified the pollutant effects by two settings for model. The iDLM included both the main effects **β(lag)** and cascading effects **b(lag, d**).

$$\beta\left( lag \right)=\sum_{m=1}^{M} \eta_{m}s_{m}\left( lag \right).$$

$$b\left( lag,d \right)=\sum_{p=1}^{P} \sum_{q=1}^{Q} \theta_{pq}\pi_{p}\left( lag \right)\psi_{q}\left( d \right)$$

The lag dimension for the main effects was modeled using a natural cubic spline with **M = 3** basis functions. The interactions of cascading effects was modeled using a tensor product of two penalized splines, with a total of **P×Q = 6** basis functions to ensure flexibility while preventing overfitting.

For comparison, a standard DLM was also fitted while the main effects were specified identically to the iDLM and the interactions terms were removed.

**Second-Stage: Meta-Analysis**

The county-specific coefficients and their variance-covariance matrices for the spline parameters were pooled in a second stage using a random-effects meta-analysis, implemented with the *mixmeta* R package. This was performed separately for the iDLM (both main and interaction terms) and the standard DLM to obtain the nationwide average exposure-response curves.

**Attributable Fraction**

To quantify the total public health burden attributable to air pollution, we calculated the county-specific Attributable Fraction (AF). The AF represents the fraction of total mortality that would have been averted under a counterfactual scenario of zero exposure to the pollutant. Our calculation followed a Monte Carlo simulation approach to robustly propagate the uncertainty from the pooled model coefficients into the final AF estimates.

First, we calculated the total AF for each county based on the pooled coefficients from the meta-analysis for both the iDLM and the standard DLM. The AF was calculated by predicting mortality under the observed daily pollution time-series and comparing it to a counterfactual scenario where all pollution main effects (and interaction terms for the iDLM) were set to zero.

The specific computational procedure was as follows:

1. Obtain Pooled Coefficients: From the second-stage meta-analysis, we obtained the vector of pooled basis coefficients ($\hat{\beta}$) and its corresponding variance-covariance matrix ($V\left( \hat{\beta} \right)$) for both the iDLM and the standard DLM.
2. Simulate Coefficients: We drew 1,000 random samples of the coefficients from a multivariate normal distribution with mean $\hat{\beta}$ and variance $V\left( \hat{\beta} \right)$. This step generates a distribution of 1,000 plausible exposure-response curves, fully accounting for the estimation uncertainty of the pooled model.
3. Calculate County-Specific AF: For each of the 1,000 simulated coefficient sets, and for each county, we calculated the daily attributable risk ($AR_{t}$) using the formula: $AR_{t}=1-exp\left( -X_{t}*\beta_{sim} \right)$, where $X_{t}$ is the vector of exposure basis variables on day *t* for that county and $\beta_{sim}$ is a simulated coefficient vector from Step 2. The total AF for that county and simulation run ($AF_{sim}$) was then calculated by weighting the daily $AR_{t}$ by the number of deaths on each day $y_{t}$, and summing over the entire time series:

$$AF_{sim}=\Sigma\left( AR_{t}*y_{t} \right)/\Sigma\left( y_{t} \right)$$

4. Summarize Results: The final point estimate for the county-specific AF was calculated as the mean of the 1,000 $AF_{sim}$ values. The 95% confidence interval was derived from the 2.5th and 97.5th percentiles of this empirical distribution. This process was performed separately for $AF_{iDLM}$ (using the full set of main and interaction basis variables) and $AF_{DLM}$ (using only the main effect basis variables).

Furthermore, to quantify the magnitude of risk missed by traditional approaches, we calculated the percentage of the total burden identified by the iDLM that is overlooked by the standard DLM. This metric was defined for each county as:

$$\left( AF_{iDLM}-AF_{DLM} \right)/AF_{iDLM}*100$$

**S3. Re-parameterization of Interaction Effects via Penalized Splines**

A central challenge in modeling all pairwise interactions is the high dimensionality and severe multicollinearity of the resulting predictor variables. A "saturated" model, including a separate coefficient $b_{lag,d}$ for each lag,d interaction term, would be statistically unstable and prone to overfitting. The iDLM framework overcomes this by re-parameterizing the high-dimensional interaction matrix using a low-dimensional set of two-dimensional spline basis functions. This process involves two key steps: dimensionality reduction before model fitting, and reconstruction of interpretable effects after model fitting.

**Dimensionality Reduction**

The core assumption is that the individual coefficients $b_{\mathrm{lag},d}$ do not vary randomly but lie on a smooth two-dimensional surface defined by lag and d. This smooth surface can be approximated by a linear combination of a small number of 2D basis functions.

First, we conceptually define the full space of interactions. For a max lag L = 6 and max interval D = 6, we need to create 21 $\mathrm{interactio}n_{lag,d}$ variables ($\frac{L\left( L+1 \right)}{2}$), where each represents the product of two lagged exposures. Second, we use the *gam* function from the *mgcv* package to generate a basis matrix for a penalized 2D spline. This is done by fitting a dummy model $\mathrm{gam}\left( y \sim s\left( \mathrm{lag}, d, k = k \right) \right)$, where **k** specifies the total number of basis functions. Through the design matrix of the dummy model **T**, the high-dimensional set of raw interaction terms is then transformed into a new, low-dimensional set of **k** predictor variables. Then the Poisson regression model is then fitted using these new predictor variables, thereby estimating only **k** coefficients (let's call them $\theta_{1}$ to $\theta_{k}$) for the entire interaction surface. This re-parameterization not only provides statistical stability by breaking the multicollinearity but also confers a significant computational advantage. As demonstrated in Figure S1, the computational time of the iDLM scales much more favorably with increasing sample size compared to an unregularized model that includes all individual interaction terms.

**Reconstruction of Interpretable Effects**

The **θ** coefficients estimated by the model for the basis functions are not directly interpretable. To understand the cascading effect at a specific **lag** and **d**, we must reconstruct the $b_{lag,d}$ coefficient from the estimated **θ** values. The relationship is the reverse of the dimension-reduction step. The estimated coefficient for any specific (lag, d) pair, $\hat{b_{lag,d}}$, is a linear combination of the estimated basis coefficients $\hat{\theta}$. The weights for this combination are also given by the design matrix **T** of the dummy model. The variance-covariance matrix of the interpretable coefficients, $V\left( \hat{b} \right)$, is calculated from the variance-covariance matrix of the basis coefficients, $V\left( \hat{\theta} \right)$, as follows:

$$V\left( \hat{b} \right) =T$$

This process allows us to generate the interpretable 2D surfaces and their confidence intervals, as presented in Figure 2 of the main text.

**S4. A Toy Example Illustrating Cascading vs. Additive Effects**

To build a clear, intuitive understanding of the fundamental difference between the iDLM framework and traditional additive models, we present a simplified "toy example." This example is designed to illustrate how the total health burden can be critically dependent on the temporal pattern of exposure when cascading effects are present, even when the total cumulative exposure is held constant.

**Scenario Setup**

We consider a simplified 4-day time series and define two distinct exposure scenarios, each with a total cumulative exposure of 2 units:

- **Scenario A (Isolated Spikes):** Two single-day exposure events separated by a day of no exposure. The exposure sequence is [1, 0, 1, 0].
- **Scenario B (Repeated Exposure):** A two-day period of consecutive exposure, followed by two days of no exposure. The exposure sequence is [1, 1, 0, 0].

**Model Assumptions**

For this illustrative example, we assume a simple health risk model where risk on a given day is influenced by both the current day's exposure (lag=0) and the previous day's exposure (lag=1). The main effect coefficient for lag 0 is $\beta_{0}$ = 0.1 and for lag 1 is $\beta_{1}$ = 0.05. For the iDLM, we introduce a single positive interaction term $b_{0,1}$ = 0.08, representing a synergistic (amplifying) effect when exposure occurs on two consecutive days.

**Comparison of Cumulative Risk**

We then calculate the total cumulative risk over the 4-day period for each model under both scenarios. The results are visualized in Figure S2.

Standard DLM calculates risk as a simple sum of the independent daily effects ($Risk=\beta_{0}*x_{t}+\beta_{1}*x_{t-1}$). Because this model is blind to the temporal pattern, it predicts the exact same total cumulative risk (0.30 units) for both Scenario A and Scenario B, as the total exposure dose is identical. However, iDLM includes the additional cascading term ($Risk=\beta_{0}*x_{t}+\beta_{1}*x_{t}-1+b_{0,1}*x_{t}*x_{t-1}$). In Scenario A, the interaction term is never activated because $x_{t}*x_{t-1}$ is always zero. Thus, the total risk is the same as the additive model (0.30 units). However, in Scenario B, the interaction term is activated on Day 2 (1*1), adding extra risk. Consequently, the iDLM predicts a substantially higher total cumulative risk (0.38 units) for the repeated exposure scenario.

**Figure S1. Computational Scalability and Efficiency of the iDLM.** The y-axis represents the Computational Speedup Factor, calculated as the ratio of the computation time of the unregularized model to that of the iDLM. The x-axis shows the number of observations (sample size) in the simulated dataset.

**Figure S2. A Toy Example Illustrating the Difference Between Cascading and Additive Risk Accumulation.**
