# Supplementary material for: Modelling beyond additive risks: A framework for estimating the cascading burden of environmental exposure
Source: Eco Environ Health. 2026 Sep 1;5(4):100281. doi: 10.1016/j.eehl.2026.100281 (PMC13587907; doi:10.1016/j.eehl.2026.100281)
Supplement: Supplementary file 2 [file mmc2.docx]

**Table S1.** Study-period coverage and descriptive statistics by geographic unit for the PM_2.5_ and O_3_ analyses

| **Pollutant** | **Geographic unit** | **Total deaths** | **Observation days** | **Study period** | | **Exposure concentration (PM_2.5_: µg/m³; O_3_: ppb)** | | | **Mean temperature (°C)** | | | **Temperature variability (°C)** | | |
| --- | --- | --- | --- | --- | --- | --- | --- | --- | --- | --- | --- | --- | --- | --- |
|  |  |  |  | **Start** | **End** | **Mean** | **2.5th** | **97.5th** | **Mean** | **2.5th** | **97.5th** | **Mean** | **2.5th** | **97.5th** |
| PM2.5 | 00036 | 413,347 | 2,273 | 01/07/73 | 01/01/81 | 14.45 | 9.02 | 28.80 | 11.41 | -6.91 | 26.20 | 2.75 | 0.93 | 5.06 |
| PM2.5 | 02090 | 87 | 208 | 02/19/73 | 12/20/75 | 14.76 | 4.57 | 41.31 | -2.53 | -29.38 | 17.70 | 2.45 | 0.55 | 4.93 |
| PM2.5 | 06001 | 66,150 | 2,674 | 01/07/73 | 08/01/81 | 12.91 | 6.54 | 32.14 | 14.12 | 5.43 | 24.83 | 3.93 | 1.46 | 6.54 |
| PM2.5 | 06067 | 97,491 | 5,681 | 01/07/73 | 12/31/88 | 9.84 | 3.76 | 33.45 | 15.81 | 4.99 | 27.33 | 4.37 | 1.25 | 7.08 |
| PM2.5 | 06073 | 91,919 | 2,696 | 01/07/73 | 08/01/81 | 13.48 | 8.11 | 22.52 | 16.71 | 6.82 | 27.76 | 4.01 | 1.34 | 5.77 |
| PM2.5 | 06075 | 30,727 | 1,349 | 10/04/83 | 12/31/88 | 17.52 | 4.00 | 35.39 | 13.91 | 7.64 | 19.29 | 2.70 | 0.97 | 4.97 |
| PM2.5 | 06083 | 18,512 | 3,032 | 01/11/73 | 08/01/81 | 8.87 | 5.51 | 15.13 | 14.78 | 6.15 | 24.29 | 3.50 | 1.24 | 5.22 |
| PM2.5 | 09005 | 8,327 | 2,267 | 01/07/75 | 08/01/81 | 10.91 | 7.20 | 16.60 | 8.59 | -12.13 | 24.22 | 3.06 | 0.98 | 5.54 |
| PM2.5 | 12099 | 27,487 | 1,522 | 04/07/77 | 08/01/81 | 14.68 | 3.65 | 20.33 | 23.38 | 12.94 | 28.33 | 2.35 | 1.03 | 4.15 |
| PM2.5 | 16005 | 2,815 | 2,470 | 01/07/73 | 01/01/81 | 7.66 | 3.35 | 18.75 | 6.18 | -12.47 | 23.75 | 3.84 | 1.18 | 6.85 |
| PM2.5 | 16077 | 311 | 2,470 | 01/07/73 | 01/01/81 | 50.77 | 6.05 | 75.63 | 7.31 | -12.05 | 25.23 | 3.81 | 1.25 | 6.71 |
| PM2.5 | 17031 | 504,484 | 3,814 | 01/29/77 | 12/31/88 | 14.48 | 6.99 | 25.72 | 9.50 | -14.29 | 26.82 | 2.60 | 0.74 | 5.33 |
| PM2.5 | 17201 | 7,379 | 1,259 | 08/08/77 | 08/01/81 | 11.08 | 4.06 | 22.86 | 8.51 | -15.06 | 25.36 | 3.12 | 0.92 | 5.72 |
| PM2.5 | 18089 | 44,632 | 3,814 | 01/29/77 | 12/31/88 | 18.98 | 6.51 | 33.88 | 9.74 | -14.19 | 26.82 | 2.88 | 0.80 | 5.74 |
| PM2.5 | 22071 | 45,002 | 2,786 | 01/07/73 | 01/01/81 | 16.18 | 4.12 | 23.39 | 20.47 | 5.25 | 29.11 | 2.09 | 0.78 | 3.84 |
| PM2.5 | 24033 | 8,572 | 992 | 06/07/73 | 08/01/81 | 12.89 | 4.47 | 36.33 | 13.18 | -4.23 | 27.73 | 2.81 | 0.98 | 4.94 |
| PM2.5 | 24510 | 67,914 | 2,142 | 05/18/75 | 12/31/88 | 17.55 | 2.81 | 55.11 | 13.02 | -5.63 | 27.87 | 2.88 | 0.93 | 5.26 |
| PM2.5 | 26163 | 248,379 | 3,991 | 02/05/77 | 12/31/88 | 13.15 | 6.83 | 30.92 | 9.62 | -12.72 | 26.10 | 2.65 | 0.84 | 5.11 |
| PM2.5 | 34001 | 18,938 | 2,653 | 01/07/73 | 12/28/80 | 10.95 | 2.89 | 26.93 | 13.04 | -4.90 | 26.85 | 2.50 | 0.86 | 4.43 |
| PM2.5 | 34013 | 31,266 | 1,287 | 01/07/77 | 01/01/81 | 8.18 | 2.60 | 19.50 | 10.03 | -8.96 | 25.30 | 2.96 | 1.05 | 5.43 |
| PM2.5 | 36055 | 61,116 | 3,565 | 01/07/73 | 12/31/88 | 9.39 | 2.43 | 30.24 | 8.82 | -11.49 | 24.88 | 2.53 | 0.69 | 5.32 |
| PM2.5 | 37021 | 13,705 | 2,825 | 01/07/73 | 08/01/81 | 16.46 | 4.24 | 32.78 | 11.80 | -5.82 | 23.33 | 3.39 | 1.16 | 6.02 |
| PM2.5 | 39061 | 1,055 | 41 | 12/31/76 | 07/27/78 | 13.88 | 8.55 | 24.26 | -1.13 | -15.14 | 24.58 | 2.94 | 1.14 | 6.32 |
| PM2.5 | 40109 | 41,916 | 2,998 | 01/07/73 | 08/01/81 | 10.16 | 6.36 | 24.18 | 16.32 | -3.72 | 31.64 | 3.46 | 1.12 | 6.01 |
| PM2.5 | 42129 | 301 | 27 | 06/26/76 | 02/03/78 | 10.44 | 5.14 | 16.55 | -1.04 | -12.55 | 19.41 | 2.63 | 1.31 | 6.91 |
| PM2.5 | 45019 | 5,232 | 1,049 | 01/07/75 | 01/01/78 | 12.70 | 4.74 | 25.79 | 19.19 | 3.70 | 28.43 | 2.31 | 0.92 | 4.31 |
| PM2.5 | 47065 | 856 | 101 | 06/11/78 | 07/07/81 | 10.52 | 3.93 | 19.53 | 9.33 | -4.15 | 23.64 | 3.34 | 1.28 | 6.21 |
| PM2.5 | 47093 | 13,156 | 1,697 | 01/07/75 | 01/01/80 | 14.34 | 5.43 | 26.70 | 13.82 | -4.46 | 26.35 | 3.40 | 1.20 | 5.80 |
| PM2.5 | 48201 | 256,965 | 5,970 | 01/07/72 | 12/31/88 | 12.43 | 6.76 | 16.96 | 20.22 | 4.95 | 29.64 | 3.11 | 1.11 | 5.51 |
| PM2.5 | 48439 | 109,121 | 6,021 | 01/07/72 | 12/31/88 | 11.87 | 4.41 | 17.70 | 18.74 | 0.46 | 32.13 | 3.39 | 1.04 | 5.97 |
| PM2.5 | 48485 | 17,335 | 5,562 | 01/14/73 | 12/31/88 | 10.75 | 1.51 | 22.72 | 18.18 | -1.30 | 32.52 | 3.68 | 1.16 | 6.54 |
| PM2.5 | 49035 | 115 | 12 | 01/24/77 | 01/21/78 | 29.16 | 7.49 | 39.35 | -4.80 | -7.10 | -1.99 | 3.38 | 0.77 | 6.06 |
| PM2.5 | 06037 | 863,896 | 5,156 | 01/07/73 | 12/31/88 | 20.23 | 7.09 | 36.08 | 16.11 | 4.70 | 27.57 | 4.32 | 1.52 | 6.25 |
| PM2.5 | 06047 | 10,641 | 4,552 | 01/07/73 | 12/31/88 | 11.78 | 5.35 | 17.73 | 17.34 | 5.44 | 29.46 | 4.50 | 1.56 | 6.85 |
| PM2.5 | 06059 | 89,071 | 3,037 | 01/07/73 | 08/01/81 | 18.08 | 5.09 | 44.92 | 16.42 | 9.05 | 25.02 | 3.50 | 1.18 | 6.27 |
| PM2.5 | 06111 | 22,326 | 2,575 | 12/11/79 | 12/31/88 | 12.50 | 7.16 | 20.96 | 15.57 | 4.85 | 26.15 | 4.03 | 1.50 | 6.08 |
| PM2.5 | 18003 | 1,999 | 356 | 03/10/73 | 08/25/79 | 14.89 | 4.80 | 26.07 | 17.32 | 3.64 | 25.66 | 3.24 | 1.17 | 5.45 |
| PM2.5 | 22063 | 243 | 489 | 09/18/86 | 12/31/88 | 9.94 | 2.37 | 24.60 | 19.64 | 5.59 | 28.58 | 3.02 | 0.96 | 5.25 |
| PM2.5 | 22087 | 1,527 | 1,170 | 08/08/85 | 12/31/88 | 11.75 | 3.89 | 22.84 | 21.08 | 7.62 | 28.74 | 1.36 | 0.48 | 2.79 |
| PM2.5 | 25003 | 23,762 | 5,586 | 01/07/73 | 12/31/88 | 9.57 | 4.99 | 15.04 | 7.50 | -13.36 | 23.50 | 3.03 | 0.92 | 5.58 |
| PM2.5 | 36029 | 169,177 | 5,907 | 01/07/72 | 12/31/88 | 12.41 | 5.38 | 32.77 | 8.40 | -12.31 | 23.85 | 2.53 | 0.70 | 5.33 |
| PM2.5 | 36119 | 35,084 | 1,567 | 01/07/73 | 12/28/80 | 12.41 | 3.93 | 24.08 | 10.33 | -8.58 | 25.24 | 2.72 | 0.96 | 5.07 |
| PM2.5 | 42081 | 4,796 | 1,409 | 01/07/73 | 07/24/78 | 8.40 | 1.22 | 21.33 | 8.54 | -10.17 | 23.24 | 3.04 | 1.01 | 5.92 |
| PM2.5 | 42125 | 169 | 27 | 06/26/76 | 02/03/78 | 9.10 | 5.17 | 15.20 | -0.67 | -12.97 | 19.63 | 2.85 | 1.33 | 6.74 |
| PM2.5 | 47037 | 360 | 20 | 01/18/78 | 12/24/88 | 13.44 | 8.68 | 20.47 | 8.94 | -4.22 | 20.50 | 2.52 | 0.93 | 4.02 |
| PM2.5 | 47157 | 120,642 | 5,498 | 01/07/73 | 12/31/88 | 16.81 | 5.70 | 25.13 | 16.52 | -2.81 | 29.71 | 3.15 | 1.01 | 5.46 |
| PM2.5 | 49049 | 22 | 12 | 01/24/77 | 01/21/78 | 20.86 | 0.83 | 34.15 | -5.21 | -7.58 | -3.22 | 3.90 | 1.20 | 6.61 |
| PM2.5 | 53005 | 3,116 | 2,546 | 01/07/73 | 12/03/85 | 8.60 | 2.98 | 22.66 | 12.43 | -5.54 | 29.16 | 3.90 | 1.32 | 6.44 |
| PM2.5 | 09001 | 37,557 | 2,089 | 01/07/75 | 11/22/83 | 11.38 | 5.28 | 26.28 | 9.43 | -9.96 | 24.74 | 2.75 | 0.97 | 5.03 |
| PM2.5 | 09003 | 43,230 | 2,267 | 01/07/75 | 08/01/81 | 11.70 | 4.82 | 20.78 | 9.37 | -11.00 | 25.01 | 3.18 | 1.01 | 5.72 |
| PM2.5 | 11001 | 128,150 | 5,615 | 01/07/73 | 12/31/88 | 13.67 | 4.91 | 40.08 | 13.27 | -5.06 | 28.09 | 3.28 | 1.00 | 5.93 |
| PM2.5 | 12011 | 45,724 | 1,537 | 08/18/76 | 08/01/81 | 7.56 | 4.84 | 14.74 | 23.46 | 13.18 | 28.24 | 2.67 | 1.27 | 4.76 |
| PM2.5 | 12033 | 13,386 | 2,804 | 01/14/73 | 04/28/84 | 20.21 | 6.15 | 47.05 | 19.11 | 3.15 | 28.42 | 3.09 | 1.14 | 5.23 |
| PM2.5 | 12103 | 179,124 | 5,539 | 01/07/73 | 12/31/88 | 13.55 | 7.69 | 24.59 | 22.10 | 10.98 | 28.33 | 1.67 | 0.63 | 3.24 |
| PM2.5 | 15003 | 65,100 | 6,165 | 01/07/72 | 12/31/88 | 8.05 | 3.50 | 10.79 | 23.02 | 20.00 | 25.46 | 1.29 | 0.66 | 1.88 |
| PM2.5 | 26099 | 42,126 | 3,977 | 07/07/77 | 12/31/88 | 12.81 | 4.90 | 17.88 | 9.38 | -12.68 | 25.92 | 2.59 | 0.81 | 5.09 |
| PM2.5 | 36063 | 9,287 | 1,608 | 12/28/83 | 12/31/88 | 13.52 | 6.79 | 25.77 | 9.03 | -11.06 | 25.11 | 2.25 | 0.67 | 4.69 |
| PM2.5 | 36087 | 7,339 | 1,567 | 01/07/73 | 12/28/80 | 6.97 | 1.21 | 17.28 | 10.28 | -8.57 | 25.13 | 2.83 | 0.97 | 5.22 |
| PM2.5 | 41039 | 10,924 | 2,675 | 01/07/73 | 08/01/81 | 10.73 | 4.16 | 24.27 | 8.44 | -2.53 | 20.15 | 3.40 | 0.95 | 6.58 |
| PM2.5 | 42015 | 2,805 | 1,710 | 01/07/73 | 08/01/78 | 10.45 | 6.43 | 15.54 | 7.92 | -12.11 | 23.06 | 3.08 | 1.03 | 6.14 |
| PM2.5 | 48141 | 291 | 92 | 09/07/72 | 01/01/73 | 10.58 | 5.15 | 18.46 | 14.86 | 1.75 | 24.22 | 4.01 | 2.13 | 6.01 |
| PM2.5 | 48323 | 1,931 | 5,531 | 01/07/73 | 12/31/88 | 12.25 | 4.16 | 26.26 | 21.99 | 6.72 | 32.32 | 3.72 | 1.20 | 6.36 |
| PM2.5 | 48355 | 13,576 | 2,682 | 01/14/73 | 01/01/81 | 10.86 | 4.59 | 16.93 | 22.33 | 8.40 | 29.72 | 2.78 | 1.09 | 4.93 |
| PM2.5 | 53061 | 28,215 | 5,201 | 01/07/73 | 12/31/88 | 17.46 | 4.43 | 24.17 | 6.47 | -6.05 | 18.96 | 2.75 | 0.65 | 6.05 |
| PM2.5 | 53063 | 15,410 | 2,269 | 01/07/73 | 12/08/80 | 10.91 | 5.15 | 22.10 | 9.23 | -9.60 | 25.47 | 3.67 | 1.05 | 6.52 |
| PM2.5 | 53067 | 4,832 | 2,534 | 01/07/73 | 08/01/81 | 10.42 | 2.61 | 41.17 | 9.84 | -0.02 | 20.21 | 2.90 | 0.78 | 5.76 |
| PM2.5 | 01073 | 108,803 | 5,482 | 01/07/73 | 12/31/88 | 21.24 | 9.51 | 41.75 | 16.99 | -0.30 | 28.99 | 3.33 | 1.05 | 5.57 |
| PM2.5 | 08041 | 13,585 | 2,840 | 01/07/75 | 12/31/88 | 7.20 | 2.18 | 13.16 | 7.89 | -9.80 | 22.66 | 4.68 | 1.60 | 7.11 |
| PM2.5 | 24005 | 13,252 | 992 | 06/07/73 | 08/01/81 | 8.31 | 4.14 | 15.54 | 11.88 | -6.38 | 26.53 | 2.85 | 1.11 | 5.01 |
| PM2.5 | 34017 | 20,355 | 1,287 | 01/07/77 | 01/01/81 | 14.04 | 5.56 | 36.33 | 10.18 | -9.14 | 25.50 | 2.92 | 1.01 | 5.36 |
| PM2.5 | 34039 | 16,874 | 1,287 | 01/07/77 | 01/01/81 | 15.34 | 7.38 | 34.80 | 10.36 | -8.50 | 25.59 | 2.92 | 1.03 | 5.37 |
| PM2.5 | 36067 | 30,720 | 2,715 | 01/07/73 | 01/01/81 | 7.14 | 1.78 | 46.08 | 8.03 | -12.52 | 23.83 | 2.75 | 0.81 | 5.60 |
| PM2.5 | 39049 | 19,353 | 1,309 | 08/02/76 | 08/01/81 | 11.60 | 5.70 | 24.32 | 9.64 | -13.78 | 25.24 | 3.15 | 0.96 | 6.02 |
| PM2.5 | 39151 | 9,627 | 1,306 | 11/27/76 | 08/01/81 | 15.19 | 3.99 | 28.61 | 8.90 | -12.54 | 24.37 | 3.09 | 0.94 | 6.01 |
| PM2.5 | 41043 | 3,625 | 2,439 | 01/07/73 | 01/01/81 | 12.96 | 4.32 | 35.93 | 8.43 | -3.36 | 20.84 | 3.41 | 0.89 | 6.70 |
| PM2.5 | 44007 | 22,324 | 1,344 | 01/07/75 | 12/31/78 | 10.36 | 5.26 | 22.37 | 9.54 | -9.70 | 24.68 | 3.11 | 0.89 | 5.66 |
| PM2.5 | 48303 | 11,642 | 2,962 | 01/07/73 | 08/01/81 | 6.60 | 3.10 | 14.21 | 15.99 | -2.14 | 29.28 | 4.30 | 1.52 | 6.98 |
| PM2.5 | 48375 | 8,726 | 2,803 | 06/07/73 | 05/31/81 | 6.21 | 2.76 | 12.69 | 14.93 | -4.13 | 29.46 | 4.37 | 1.43 | 7.08 |
| PM2.5 | 49057 | 31 | 12 | 01/24/77 | 01/21/78 | 23.05 | 6.07 | 37.52 | -6.31 | -9.60 | -2.21 | 3.70 | 1.36 | 6.30 |
| PM2.5 | 53033 | 26,444 | 905 | 01/07/73 | 12/31/88 | 9.38 | 5.01 | 25.79 | 8.33 | -3.83 | 20.09 | 2.89 | 0.77 | 6.34 |
| PM2.5 | 53053 | 54,809 | 5,676 | 01/07/73 | 12/31/88 | 17.49 | 9.24 | 26.39 | 7.26 | -4.21 | 19.56 | 2.81 | 0.74 | 5.90 |
| PM2.5 | 06019 | 14,284 | 1,463 | 09/07/72 | 01/01/77 | 17.28 | 7.88 | 47.07 | 12.85 | 0.00 | 25.56 | 4.18 | 1.66 | 6.06 |
| PM2.5 | 06061 | 15,602 | 5,710 | 01/07/73 | 12/31/88 | 16.39 | 13.76 | 23.61 | 10.99 | -1.34 | 24.55 | 3.96 | 0.90 | 6.59 |
| PM2.5 | 20209 | 44 | 12 | 06/09/73 | 06/20/73 | 28.41 | 17.81 | 35.73 | 24.00 | 20.33 | 26.33 | 3.12 | 1.95 | 4.11 |
| PM2.5 | 26081 | 13,169 | 1,255 | 11/26/76 | 08/01/81 | 9.13 | 4.13 | 19.62 | 7.60 | -14.49 | 24.48 | 2.95 | 0.89 | 5.57 |
| PM2.5 | 34003 | 30,329 | 1,567 | 01/07/73 | 12/28/80 | 15.91 | 6.13 | 29.40 | 10.73 | -7.85 | 25.51 | 2.85 | 0.97 | 5.19 |
| PM2.5 | 41059 | 2,741 | 2,546 | 01/07/73 | 12/03/85 | 9.64 | 2.38 | 22.68 | 9.08 | -7.03 | 24.68 | 3.76 | 1.26 | 6.88 |
| PM2.5 | 42007 | 185 | 27 | 06/26/76 | 02/03/78 | 10.04 | 2.75 | 20.26 | -1.46 | -13.57 | 19.36 | 2.83 | 1.16 | 6.98 |
| PM2.5 | 51760 | 9,492 | 1,199 | 01/07/73 | 01/01/77 | 14.69 | 9.09 | 31.10 | 14.51 | -1.49 | 27.01 | 3.33 | 1.19 | 5.70 |
| PM2.5 | 53077 | 7,736 | 2,219 | 01/07/73 | 08/01/81 | 8.33 | 2.87 | 23.36 | 7.73 | -7.81 | 23.03 | 3.56 | 1.08 | 6.29 |
| PM2.5 | 06099 | 29,586 | 4,552 | 01/07/73 | 12/31/88 | 16.73 | 5.04 | 35.81 | 16.75 | 5.04 | 28.77 | 4.45 | 1.43 | 6.80 |
| PM2.5 | 32003 | 8,737 | 1,415 | 01/07/73 | 01/02/77 | 8.08 | 5.91 | 10.80 | 17.18 | 1.25 | 32.80 | 3.85 | 1.81 | 5.32 |
| PM2.5 | 33001 | 2,733 | 2,499 | 01/07/73 | 10/15/84 | 5.48 | 2.45 | 14.15 | 6.85 | -14.34 | 23.36 | 2.96 | 0.89 | 5.88 |
| PM2.5 | 36001 | 52,630 | 5,564 | 01/07/73 | 12/31/88 | 12.35 | 4.89 | 34.17 | 8.07 | -12.93 | 23.92 | 3.15 | 0.95 | 6.11 |
| PM2.5 | 36103 | 53,612 | 2,089 | 01/07/75 | 11/22/83 | 8.58 | 5.43 | 24.38 | 10.58 | -7.32 | 24.82 | 1.94 | 0.65 | 3.85 |
| PM2.5 | 40031 | 10,599 | 5,562 | 01/14/73 | 12/31/88 | 17.07 | 8.86 | 24.65 | 16.89 | -2.33 | 31.44 | 3.52 | 1.11 | 6.16 |
| PM2.5 | 41051 | 39,758 | 2,222 | 10/21/81 | 12/31/88 | 8.18 | 3.31 | 33.44 | 10.67 | -1.34 | 23.21 | 3.31 | 0.98 | 6.79 |
| PM2.5 | 46013 | 3,055 | 3,314 | 01/07/73 | 10/01/82 | 6.89 | 4.72 | 18.10 | 7.16 | -20.66 | 27.01 | 3.50 | 1.01 | 6.55 |
| PM2.5 | 48029 | 54,045 | 2,835 | 01/07/73 | 01/01/81 | 21.08 | 12.94 | 26.02 | 19.76 | 3.94 | 30.09 | 3.53 | 1.19 | 5.88 |
| PM2.5 | 51161 | 1,876 | 1,542 | 01/07/73 | 10/15/88 | 7.91 | 2.79 | 13.53 | 12.27 | -4.41 | 24.41 | 3.47 | 1.09 | 6.38 |
| PM2.5 | 09009 | 52,750 | 2,653 | 01/07/75 | 11/22/83 | 11.71 | 3.52 | 22.32 | 10.08 | -9.20 | 25.07 | 2.70 | 0.93 | 4.89 |
| PM2.5 | 25005 | 42,148 | 3,507 | 01/07/73 | 12/31/88 | 14.94 | 5.61 | 40.89 | 10.04 | -8.48 | 24.89 | 2.60 | 0.84 | 4.79 |
| PM2.5 | 26091 | 2,205 | 1,216 | 12/26/76 | 07/10/88 | 11.22 | 4.09 | 23.52 | 8.86 | -14.27 | 25.11 | 3.09 | 0.96 | 5.67 |
| PM2.5 | 39153 | 12,568 | 1,306 | 11/27/76 | 08/01/81 | 18.07 | 4.52 | 34.83 | 8.72 | -13.08 | 24.38 | 3.02 | 0.87 | 5.99 |
| PM2.5 | 42085 | 3,205 | 1,265 | 01/07/78 | 08/01/81 | 20.11 | 8.76 | 23.70 | 8.25 | -13.12 | 23.17 | 3.07 | 0.87 | 5.88 |
| PM2.5 | 45045 | 17,503 | 2,825 | 01/07/73 | 08/01/81 | 16.46 | 7.63 | 33.56 | 15.35 | -1.40 | 27.30 | 3.44 | 1.02 | 5.92 |
| PM2.5 | 48245 | 22,985 | 3,433 | 01/18/73 | 12/19/88 | 13.64 | 5.51 | 25.76 | 20.77 | 5.70 | 29.13 | 2.36 | 0.92 | 4.34 |
| PM2.5 | 48453 | 40,994 | 5,595 | 01/14/73 | 12/31/88 | 10.37 | 5.35 | 16.98 | 19.73 | 3.47 | 30.70 | 3.45 | 1.08 | 5.77 |
| PM2.5 | 06085 | 61,252 | 3,058 | 01/07/73 | 04/28/84 | 14.12 | 5.82 | 59.23 | 13.99 | 5.38 | 24.71 | 3.91 | 1.42 | 6.31 |
| PM2.5 | 06107 | 17,431 | 3,508 | 01/07/73 | 11/01/88 | 20.71 | 2.93 | 43.31 | 12.02 | -0.42 | 25.01 | 4.07 | 1.49 | 5.89 |
| PM2.5 | 13089 | 15,783 | 1,748 | 08/11/81 | 12/31/88 | 12.26 | 3.32 | 21.70 | 16.32 | 0.14 | 28.83 | 3.47 | 0.97 | 6.06 |
| PM2.5 | 18141 | 28,667 | 4,778 | 01/07/73 | 12/31/88 | 18.07 | 5.39 | 41.10 | 10.33 | -12.84 | 25.99 | 3.08 | 0.85 | 6.00 |
| PM2.5 | 27137 | 6,699 | 1,037 | 01/07/78 | 01/01/81 | 6.15 | 3.99 | 11.38 | 3.22 | -23.18 | 22.25 | 2.81 | 0.87 | 5.64 |
| PM2.5 | 36059 | 49,216 | 1,567 | 01/07/73 | 12/28/80 | 13.61 | 5.42 | 25.78 | 11.34 | -5.75 | 25.68 | 2.19 | 0.79 | 4.14 |
| PM2.5 | 39095 | 12,653 | 1,216 | 12/26/76 | 07/10/88 | 12.80 | 4.31 | 26.49 | 9.44 | -13.84 | 25.66 | 2.99 | 0.95 | 5.65 |
| PM2.5 | 49011 | 17 | 12 | 01/24/77 | 01/21/78 | 14.17 | 3.69 | 25.84 | -4.29 | -7.02 | -0.22 | 2.34 | 0.56 | 4.62 |
| PM2.5 | 12009 | 32,320 | 4,954 | 11/19/74 | 12/31/88 | 10.96 | 4.24 | 16.95 | 22.36 | 11.35 | 28.07 | 2.06 | 0.85 | 3.85 |
| PM2.5 | 24025 | 2,140 | 992 | 06/07/73 | 08/01/81 | 9.30 | 3.15 | 21.59 | 12.00 | -5.89 | 26.52 | 2.68 | 0.96 | 4.64 |
| PM2.5 | 35025 | 5,028 | 5,309 | 01/17/73 | 12/31/88 | 11.29 | 4.38 | 12.80 | 16.57 | 0.03 | 28.71 | 4.45 | 1.72 | 6.85 |
| PM2.5 | 40143 | 11,206 | 910 | 01/07/79 | 08/01/81 | 10.80 | 2.44 | 22.84 | 16.05 | -5.14 | 32.75 | 3.30 | 0.96 | 5.92 |
| PM2.5 | 41019 | 3,695 | 2,302 | 01/07/73 | 01/01/81 | 9.11 | 3.24 | 19.18 | 9.34 | -1.65 | 20.79 | 3.72 | 1.02 | 6.93 |
| PM2.5 | 42077 | 9,830 | 1,407 | 01/07/73 | 05/19/78 | 11.41 | 7.47 | 20.09 | 10.75 | -7.23 | 24.97 | 3.23 | 1.02 | 6.00 |
| PM2.5 | 22033 | 36,443 | 5,330 | 01/07/73 | 12/31/88 | 12.14 | 3.08 | 23.59 | 19.68 | 3.63 | 28.67 | 3.18 | 1.18 | 5.38 |
| PM2.5 | 25013 | 27,512 | 2,267 | 01/07/75 | 08/01/81 | 7.81 | 3.56 | 17.00 | 8.51 | -12.44 | 24.35 | 3.18 | 1.01 | 5.84 |
| PM2.5 | 28075 | 2,717 | 1,069 | 01/07/73 | 01/01/81 | 18.32 | 8.28 | 33.49 | 17.99 | 0.76 | 29.73 | 3.35 | 0.92 | 5.51 |
| PM2.5 | 34023 | 14,082 | 1,287 | 01/07/77 | 01/01/81 | 10.82 | 4.46 | 26.66 | 10.77 | -7.92 | 25.77 | 2.96 | 0.99 | 5.36 |
| PM2.5 | 42051 | 136 | 27 | 06/26/76 | 02/03/78 | 5.76 | 2.96 | 11.85 | -0.74 | -11.89 | 19.20 | 2.71 | 1.35 | 6.56 |
| PM2.5 | 06007 | 22,677 | 5,536 | 01/07/73 | 12/31/88 | 13.98 | 4.29 | 57.83 | 14.86 | 2.07 | 28.82 | 4.30 | 0.90 | 7.14 |
| PM2.5 | 06021 | 1,496 | 3,006 | 01/07/73 | 12/31/88 | 23.05 | 4.31 | 43.64 | 16.44 | 3.70 | 30.45 | 4.34 | 1.13 | 6.80 |
| PM2.5 | 26005 | 1,685 | 1,252 | 12/02/76 | 08/01/81 | 6.33 | 3.57 | 21.01 | 8.12 | -14.12 | 24.62 | 2.46 | 0.65 | 4.89 |
| PM2.5 | 30049 | 2,884 | 3,098 | 01/07/73 | 11/08/88 | 8.21 | 2.52 | 17.17 | 3.28 | -17.92 | 19.77 | 3.69 | 1.15 | 6.56 |
| PM2.5 | 35049 | 431 | 234 | 04/27/88 | 12/31/88 | 4.71 | 2.32 | 7.78 | 13.11 | -3.53 | 22.40 | 4.23 | 1.91 | 6.17 |
| PM2.5 | 39099 | 235 | 27 | 06/26/76 | 02/03/78 | 10.12 | 4.20 | 16.44 | -2.10 | -13.49 | 19.07 | 2.91 | 1.15 | 6.89 |
| PM2.5 | 42095 | 8,319 | 1,407 | 01/07/73 | 05/19/78 | 15.36 | 3.96 | 32.92 | 10.48 | -7.82 | 24.82 | 3.19 | 1.04 | 5.92 |
| PM2.5 | 06029 | 44,309 | 5,114 | 01/07/73 | 12/31/88 | 21.24 | 4.75 | 43.77 | 16.41 | 3.45 | 29.45 | 4.21 | 1.72 | 5.96 |
| PM2.5 | 13051 | 26,467 | 4,439 | 01/07/73 | 12/31/88 | 17.61 | 8.88 | 33.46 | 19.42 | 3.98 | 28.96 | 2.71 | 0.96 | 4.92 |
| PM2.5 | 23005 | 9,713 | 1,749 | 01/07/73 | 01/01/79 | 9.14 | 3.07 | 21.74 | 7.39 | -12.74 | 23.58 | 2.67 | 0.87 | 5.13 |
| PM2.5 | 39003 | 2,772 | 1,216 | 12/26/76 | 07/10/88 | 20.56 | 7.45 | 33.98 | 9.55 | -14.58 | 25.50 | 3.13 | 0.99 | 5.79 |
| PM2.5 | 40135 | 3,172 | 5,423 | 01/07/73 | 12/31/88 | 9.52 | 3.76 | 21.57 | 16.14 | -2.73 | 30.24 | 3.21 | 0.93 | 5.54 |
| PM2.5 | 41047 | 8,635 | 2,302 | 01/07/73 | 01/01/81 | 13.65 | 3.85 | 18.26 | 9.62 | -2.03 | 21.44 | 3.36 | 0.92 | 6.54 |
| PM2.5 | 04019 | 30,365 | 2,903 | 01/07/73 | 01/01/81 | 8.82 | 6.36 | 10.62 | 20.14 | 6.43 | 33.23 | 4.52 | 1.81 | 6.55 |
| PM2.5 | 35013 | 1,125 | 738 | 03/17/73 | 12/31/88 | 11.93 | 5.38 | 19.92 | 17.24 | 1.86 | 29.81 | 4.33 | 1.99 | 6.13 |
| PM2.5 | 42049 | 5,179 | 1,010 | 12/03/76 | 01/01/81 | 10.80 | 3.11 | 25.45 | 8.19 | -12.50 | 23.25 | 2.36 | 0.66 | 4.92 |
| PM2.5 | 42069 | 11,588 | 1,407 | 01/07/73 | 05/19/78 | 11.70 | 1.86 | 28.94 | 8.57 | -10.67 | 22.95 | 2.97 | 0.96 | 5.83 |
| PM2.5 | 51770 | 4,715 | 1,386 | 01/07/73 | 01/01/77 | 9.18 | 4.25 | 24.46 | 12.62 | -3.62 | 24.89 | 3.58 | 1.05 | 6.65 |
| PM2.5 | 51059 | 31,577 | 5,615 | 01/07/73 | 12/31/88 | 9.18 | 3.48 | 23.48 | 13.20 | -5.08 | 27.88 | 3.07 | 0.98 | 5.48 |
| PM2.5 | 53011 | 8,003 | 2,222 | 10/21/81 | 12/31/88 | 6.81 | 3.41 | 19.56 | 10.66 | -1.29 | 23.06 | 3.21 | 0.85 | 6.76 |
| PM2.5 | 54039 | 18,652 | 2,746 | 01/07/73 | 08/01/81 | 8.97 | 4.81 | 18.50 | 12.66 | -8.18 | 25.20 | 3.19 | 0.98 | 6.22 |
| PM2.5 | 06031 | 1,972 | 1,463 | 09/07/72 | 01/01/77 | 18.85 | 8.18 | 52.24 | 18.75 | 5.69 | 32.01 | 4.58 | 1.95 | 6.45 |
| PM2.5 | 42011 | 11,970 | 1,407 | 01/07/73 | 05/19/78 | 10.70 | 1.91 | 26.50 | 11.10 | -6.33 | 25.24 | 3.11 | 1.05 | 5.71 |
| PM2.5 | 06081 | 28,142 | 2,499 | 01/07/73 | 04/28/84 | 21.45 | 6.41 | 107.76 | 13.01 | 7.05 | 19.79 | 2.56 | 0.90 | 4.92 |
| PM2.5 | 39155 | 5,581 | 1,306 | 11/27/76 | 08/01/81 | 13.49 | 5.13 | 28.41 | 8.41 | -12.90 | 23.82 | 3.04 | 0.87 | 5.97 |
| PM2.5 | 41011 | 1,767 | 1,549 | 03/03/77 | 08/01/81 | 4.11 | 2.15 | 8.94 | 10.57 | 2.21 | 18.88 | 2.89 | 0.83 | 5.44 |
| PM2.5 | 42117 | 1,510 | 1,409 | 01/07/73 | 07/24/78 | 9.90 | 4.79 | 17.67 | 7.65 | -11.53 | 22.76 | 3.01 | 0.97 | 5.81 |
| PM2.5 | 51087 | 4,036 | 1,199 | 01/07/73 | 01/01/77 | 12.71 | 3.13 | 29.14 | 14.46 | -1.45 | 26.93 | 3.31 | 1.19 | 5.73 |
| PM2.5 | 12057 | 36,570 | 1,818 | 08/05/76 | 12/31/88 | 13.69 | 6.01 | 31.68 | 21.82 | 9.79 | 28.09 | 2.85 | 1.17 | 5.07 |
| PM2.5 | 19163 | 12,596 | 3,807 | 01/07/73 | 12/30/88 | 12.07 | 3.89 | 28.67 | 9.58 | -16.32 | 27.30 | 3.19 | 0.94 | 6.00 |
| PM2.5 | 42005 | 57 | 27 | 06/26/76 | 02/03/78 | 7.97 | 3.23 | 17.01 | -1.56 | -13.04 | 19.16 | 2.57 | 1.23 | 7.05 |
| PM2.5 | 53065 | 1,774 | 3,865 | 07/07/77 | 12/31/88 | 15.29 | 3.73 | 40.91 | 7.42 | -13.60 | 24.39 | 3.46 | 1.13 | 6.16 |
| PM2.5 | 01103 | 2,902 | 1,567 | 01/07/75 | 08/01/81 | 14.40 | 5.99 | 24.79 | 15.98 | -2.53 | 28.40 | 3.25 | 1.13 | 5.50 |
| PM2.5 | 06113 | 12,245 | 5,614 | 01/07/73 | 12/31/88 | 16.41 | 7.71 | 50.25 | 16.63 | 5.36 | 29.25 | 4.46 | 1.25 | 7.20 |
| PM2.5 | 15007 | 1,867 | 3,066 | 01/07/73 | 08/01/81 | 8.37 | 6.79 | 13.14 | 21.78 | 18.71 | 24.31 | 1.36 | 0.69 | 1.88 |
| PM2.5 | 53021 | 1,207 | 2,546 | 01/07/73 | 12/03/85 | 7.66 | 1.78 | 15.53 | 12.66 | -5.80 | 29.35 | 3.79 | 1.32 | 6.37 |
| PM2.5 | 23017 | 2,250 | 1,749 | 01/07/73 | 01/01/79 | 9.78 | 2.89 | 22.47 | 4.68 | -16.77 | 22.23 | 3.05 | 0.95 | 5.84 |
| PM2.5 | 40147 | 1,094 | 910 | 01/07/79 | 08/01/81 | 12.34 | 4.09 | 26.99 | 15.33 | -6.57 | 32.52 | 3.41 | 1.06 | 6.24 |
| PM2.5 | 56025 | 2,663 | 2,674 | 01/07/73 | 08/01/81 | 3.00 | 1.77 | 7.09 | 5.46 | -16.83 | 24.00 | 4.11 | 1.34 | 6.87 |
| PM2.5 | 28047 | 18,221 | 4,555 | 01/14/73 | 12/31/88 | 15.81 | 4.74 | 30.74 | 20.16 | 4.52 | 28.75 | 2.62 | 0.97 | 4.48 |
| PM2.5 | 42115 | 1,746 | 1,710 | 01/07/73 | 08/01/78 | 6.96 | 1.80 | 22.80 | 7.75 | -12.32 | 22.87 | 3.05 | 1.02 | 6.11 |
| PM2.5 | 42131 | 1,063 | 1,710 | 01/07/73 | 08/01/78 | 7.86 | 2.88 | 10.43 | 8.21 | -11.86 | 23.13 | 3.03 | 1.03 | 6.03 |
| PM2.5 | 22103 | 2,796 | 1,170 | 08/08/85 | 12/31/88 | 12.45 | 3.72 | 34.96 | 20.14 | 5.52 | 28.55 | 2.52 | 0.84 | 4.34 |
| PM2.5 | 02020 | 261 | 177 | 03/15/73 | 07/26/78 | 7.30 | 3.34 | 15.72 | -4.22 | -19.62 | 9.45 | 1.54 | 0.59 | 2.92 |
| PM2.5 | 16069 | 2,812 | 3,274 | 01/07/73 | 08/10/86 | 6.82 | 3.93 | 11.47 | 8.85 | -8.26 | 25.64 | 3.55 | 1.09 | 6.63 |
| PM2.5 | 01089 | 5,293 | 1,567 | 01/07/75 | 08/01/81 | 11.48 | 2.97 | 18.58 | 15.58 | -2.83 | 28.10 | 3.26 | 1.10 | 5.58 |
| PM2.5 | 15009 | 5,018 | 4,296 | 01/07/73 | 12/31/88 | 14.79 | 11.65 | 15.07 | 21.36 | 18.32 | 23.90 | 1.41 | 0.77 | 1.83 |
| PM2.5 | 48203 | 7,217 | 5,509 | 01/07/73 | 12/31/88 | 18.76 | 4.92 | 28.00 | 18.73 | 1.24 | 30.47 | 3.23 | 0.95 | 5.41 |
| PM2.5 | 06027 | 1,899 | 3,486 | 01/07/73 | 06/21/86 | 9.64 | 4.29 | 16.63 | 15.65 | -0.15 | 30.88 | 3.55 | 1.48 | 4.90 |
| PM2.5 | 53027 | 3,168 | 2,069 | 01/07/73 | 08/01/81 | 4.48 | 1.42 | 9.75 | 9.90 | 0.48 | 18.55 | 2.58 | 0.73 | 5.20 |
| PM2.5 | 46103 | 3,840 | 3,031 | 01/07/73 | 08/01/81 | 7.32 | 2.96 | 14.85 | 8.11 | -15.11 | 25.98 | 3.84 | 1.21 | 6.76 |
| PM2.5 | 01033 | 2,039 | 1,567 | 01/07/75 | 08/01/81 | 11.34 | 6.09 | 20.29 | 16.06 | -2.68 | 28.60 | 3.25 | 1.16 | 5.38 |
| PM2.5 | 13297 | 1,718 | 2,739 | 01/07/73 | 08/01/81 | 14.20 | 3.92 | 39.45 | 16.71 | -0.22 | 28.39 | 3.37 | 1.08 | 5.64 |
| PM2.5 | 41003 | 1,789 | 2,302 | 01/07/73 | 01/01/81 | 6.59 | 1.34 | 19.02 | 10.42 | -0.10 | 20.65 | 3.35 | 0.93 | 6.52 |
| PM2.5 | 28059 | 2,587 | 1,414 | 01/07/76 | 01/01/80 | 15.24 | 6.03 | 29.03 | 19.24 | 2.63 | 28.81 | 2.78 | 0.96 | 4.60 |
| PM2.5 | 12111 | 13,377 | 4,954 | 11/19/74 | 12/31/88 | 12.22 | 5.68 | 23.71 | 22.81 | 12.20 | 28.19 | 2.43 | 1.09 | 4.39 |
| PM2.5 | 19045 | 5,797 | 3,807 | 01/07/73 | 12/30/88 | 11.84 | 4.21 | 27.90 | 9.22 | -16.87 | 27.04 | 3.22 | 0.94 | 5.97 |
| PM2.5 | 13151 | 2,523 | 3,618 | 01/07/73 | 12/30/88 | 17.63 | 5.32 | 45.86 | 16.61 | 0.25 | 28.38 | 3.34 | 0.97 | 5.78 |
| PM2.5 | 20091 | 50 | 12 | 06/09/73 | 06/20/73 | 12.42 | 4.20 | 16.19 | 23.97 | 20.41 | 26.29 | 3.05 | 1.81 | 4.04 |
| PM2.5 | 25011 | 3,600 | 2,267 | 01/07/75 | 08/01/81 | 9.07 | 2.63 | 25.42 | 7.57 | -13.53 | 23.72 | 3.26 | 1.00 | 6.09 |
| PM2.5 | 32031 | 8,893 | 2,832 | 01/07/73 | 01/01/81 | 8.92 | 2.50 | 22.95 | 8.85 | -8.68 | 25.42 | 3.44 | 1.30 | 5.31 |
| PM2.5 | 39103 | 1,700 | 1,306 | 11/27/76 | 08/01/81 | 15.83 | 6.01 | 29.76 | 8.78 | -13.26 | 24.51 | 2.99 | 0.89 | 6.02 |
| PM2.5 | 06039 | 1,834 | 1,463 | 09/07/72 | 01/01/77 | 13.33 | 7.14 | 39.94 | 12.47 | -0.21 | 25.28 | 4.43 | 1.50 | 6.59 |
| PM2.5 | 44003 | 4,708 | 1,344 | 01/07/75 | 12/31/78 | 6.88 | 2.56 | 17.47 | 9.84 | -9.00 | 24.68 | 2.93 | 0.86 | 5.30 |
| PM2.5 | 53007 | 2,432 | 2,219 | 01/07/73 | 08/01/81 | 8.61 | 3.59 | 18.82 | 4.31 | -9.40 | 18.69 | 2.96 | 0.81 | 5.94 |
| PM2.5 | 30063 | 8,545 | 5,694 | 01/07/73 | 12/31/88 | 19.64 | 12.22 | 26.12 | 3.73 | -15.66 | 20.54 | 3.54 | 1.20 | 6.48 |
| PM2.5 | 26017 | 3,062 | 1,266 | 11/29/76 | 08/01/81 | 9.04 | 3.79 | 19.49 | 7.03 | -15.77 | 24.57 | 2.86 | 0.86 | 5.59 |
| PM2.5 | 30083 | 486 | 2,004 | 01/07/76 | 06/01/84 | 6.51 | 3.79 | 13.35 | 6.58 | -21.84 | 25.50 | 3.73 | 1.16 | 6.86 |
| PM2.5 | 12085 | 3,307 | 1,522 | 04/07/77 | 08/01/81 | 7.90 | 3.10 | 11.46 | 23.49 | 12.70 | 28.79 | 2.02 | 0.86 | 3.45 |
| PM2.5 | 34041 | 3,108 | 1,407 | 01/07/73 | 05/19/78 | 9.34 | 3.20 | 24.02 | 10.51 | -7.94 | 24.87 | 3.09 | 1.04 | 5.75 |
| PM2.5 | 36065 | 19,213 | 2,715 | 01/07/73 | 01/01/81 | 13.41 | 3.37 | 33.39 | 7.10 | -13.96 | 23.02 | 2.91 | 0.89 | 5.89 |
| PM2.5 | 41067 | 6,992 | 2,391 | 01/07/73 | 01/01/81 | 9.44 | 3.18 | 23.60 | 10.19 | -0.55 | 21.58 | 3.07 | 0.85 | 6.08 |
| PM2.5 | 13077 | 3,754 | 3,618 | 01/07/73 | 12/30/88 | 19.56 | 4.36 | 46.96 | 16.50 | 0.26 | 28.08 | 3.34 | 1.01 | 5.72 |
| PM2.5 | 26065 | 5,831 | 1,134 | 01/12/77 | 08/01/81 | 9.93 | 4.94 | 20.20 | 8.69 | -14.01 | 24.76 | 3.07 | 0.93 | 5.74 |
| PM2.5 | 06087 | 10,753 | 2,499 | 01/07/73 | 04/28/84 | 9.88 | 2.62 | 69.52 | 12.96 | 6.63 | 19.85 | 2.58 | 0.93 | 4.58 |
| PM2.5 | 16055 | 6,989 | 5,684 | 01/07/73 | 12/31/88 | 12.98 | 6.75 | 23.07 | 7.43 | -10.54 | 23.90 | 3.45 | 0.93 | 6.72 |
| PM2.5 | 16019 | 2,518 | 2,470 | 01/07/73 | 01/01/81 | 7.35 | 3.15 | 16.68 | 4.62 | -13.71 | 21.39 | 3.97 | 1.19 | 7.02 |
| PM2.5 | 30111 | 6,570 | 3,045 | 01/07/73 | 08/01/81 | 7.94 | 3.54 | 16.23 | 7.78 | -17.03 | 25.80 | 4.02 | 1.22 | 7.01 |
| PM2.5 | 47145 | 1,647 | 1,697 | 01/07/75 | 01/01/80 | 12.38 | 7.55 | 21.82 | 14.37 | -3.90 | 26.91 | 3.19 | 1.04 | 5.60 |
| PM2.5 | 08029 | 825 | 1,415 | 01/07/73 | 03/06/78 | 4.92 | 3.47 | 6.58 | 5.17 | -12.82 | 21.07 | 5.01 | 1.87 | 7.61 |
| PM2.5 | 06103 | 2,763 | 3,006 | 01/07/73 | 12/31/88 | 9.89 | 5.05 | 18.71 | 14.55 | 1.53 | 29.08 | 4.24 | 0.90 | 6.88 |
| PM2.5 | 23003 | 5,004 | 2,440 | 01/07/73 | 12/31/88 | 9.42 | 4.19 | 16.89 | 4.54 | -19.27 | 22.00 | 2.95 | 0.89 | 5.74 |
| PM2.5 | 13115 | 336 | 101 | 06/11/78 | 07/07/81 | 12.92 | 5.85 | 25.01 | 10.61 | -2.63 | 24.42 | 3.35 | 1.24 | 6.21 |
| PM2.5 | 13223 | 1,518 | 3,618 | 01/07/73 | 12/30/88 | 17.13 | 2.62 | 36.84 | 15.91 | -0.47 | 27.61 | 3.19 | 1.00 | 5.40 |
| PM2.5 | 48361 | 792 | 755 | 10/09/83 | 12/19/88 | 8.05 | 3.45 | 16.89 | 21.56 | 6.62 | 29.37 | 2.73 | 1.07 | 4.63 |
| PM2.5 | 41061 | 1,088 | 2,546 | 01/07/73 | 12/03/85 | 8.48 | 2.81 | 24.47 | 5.77 | -9.95 | 21.66 | 3.56 | 1.04 | 6.68 |
| PM2.5 | 56021 | 3,190 | 2,696 | 01/07/73 | 08/01/81 | 5.33 | 1.58 | 9.36 | 7.37 | -12.43 | 23.52 | 4.01 | 1.13 | 6.71 |
| PM2.5 | 16057 | 1,581 | 3,274 | 01/07/73 | 08/10/86 | 8.06 | 4.25 | 10.49 | 7.84 | -9.21 | 24.38 | 3.47 | 0.98 | 6.56 |
| PM2.5 | 26033 | 361 | 557 | 05/04/86 | 12/26/87 | 7.53 | 2.37 | 12.71 | 8.51 | -10.65 | 22.48 | 1.99 | 0.65 | 3.93 |
| PM2.5 | 38105 | 972 | 2,004 | 01/07/76 | 06/01/84 | 9.75 | 6.16 | 18.38 | 5.08 | -23.77 | 24.01 | 3.56 | 0.99 | 6.76 |
| PM2.5 | 47009 | 2,797 | 1,697 | 01/07/75 | 01/01/80 | 8.61 | 3.92 | 14.48 | 13.25 | -5.28 | 25.20 | 3.41 | 1.20 | 6.11 |
| PM2.5 | 06079 | 9,806 | 3,032 | 01/11/73 | 08/01/81 | 11.82 | 4.78 | 27.39 | 15.39 | 5.74 | 26.67 | 3.94 | 1.47 | 5.67 |
| PM2.5 | 16083 | 7,248 | 5,563 | 01/07/73 | 12/31/88 | 18.11 | 8.30 | 22.87 | 8.29 | -10.56 | 25.67 | 4.02 | 1.36 | 6.96 |
| PM2.5 | 53075 | 1,702 | 3,305 | 01/07/73 | 08/10/86 | 7.21 | 3.49 | 12.23 | 9.85 | -7.00 | 26.29 | 3.51 | 1.09 | 6.36 |
| PM2.5 | 23001 | 4,447 | 1,749 | 01/07/73 | 01/01/79 | 12.51 | 3.53 | 23.65 | 6.79 | -14.18 | 23.86 | 3.01 | 0.96 | 5.83 |
| PM2.5 | 53001 | 487 | 2,269 | 01/07/73 | 12/08/80 | 6.74 | 2.29 | 14.95 | 11.84 | -6.56 | 28.44 | 3.62 | 1.27 | 6.06 |
| PM2.5 | 13135 | 3,905 | 1,748 | 08/11/81 | 12/31/88 | 13.66 | 3.19 | 39.36 | 16.03 | 0.20 | 28.38 | 3.35 | 0.91 | 5.73 |
| PM2.5 | 31079 | 1,690 | 1,230 | 01/07/73 | 08/01/81 | 5.73 | 2.83 | 18.17 | 11.27 | -11.25 | 29.46 | 3.62 | 1.01 | 6.48 |
| PM2.5 | 53073 | 9,749 | 4,634 | 01/07/73 | 12/31/88 | 7.87 | 3.75 | 14.95 | 4.89 | -8.69 | 18.57 | 2.65 | 0.69 | 5.87 |
| PM2.5 | 22015 | 6,318 | 5,509 | 01/07/73 | 12/31/88 | 10.36 | 3.70 | 19.13 | 18.60 | 1.14 | 30.36 | 3.23 | 1.00 | 5.37 |
| PM2.5 | 06009 | 2,231 | 4,552 | 01/07/73 | 12/31/88 | 9.16 | 4.90 | 14.89 | 13.42 | 1.29 | 25.92 | 4.38 | 1.09 | 6.77 |
| PM2.5 | 06101 | 5,707 | 5,606 | 01/07/73 | 12/31/88 | 15.75 | 5.46 | 31.48 | 17.14 | 5.25 | 30.12 | 4.39 | 1.16 | 7.06 |
| PM2.5 | 53041 | 3,171 | 2,534 | 01/07/73 | 08/01/81 | 6.02 | 2.17 | 12.37 | 7.46 | -3.78 | 19.23 | 2.98 | 0.76 | 6.20 |
| PM2.5 | 55087 | 3,934 | 1,609 | 06/07/73 | 01/01/78 | 10.64 | 3.85 | 21.13 | 7.94 | -16.52 | 25.40 | 3.05 | 0.94 | 5.82 |
| PM2.5 | 53025 | 4,270 | 5,347 | 01/07/73 | 12/31/88 | 8.67 | 2.25 | 16.06 | 11.00 | -9.41 | 28.42 | 3.66 | 1.28 | 6.14 |
| PM2.5 | 55043 | 4,303 | 3,663 | 01/07/73 | 12/30/88 | 10.53 | 3.07 | 25.79 | 8.69 | -16.56 | 26.38 | 3.01 | 0.89 | 5.55 |
| PM2.5 | 53035 | 909 | 354 | 01/07/73 | 01/24/77 | 6.57 | 2.46 | 14.75 | 10.20 | 1.56 | 21.63 | 2.40 | 0.59 | 5.03 |
| PM2.5 | 08069 | 5,746 | 2,696 | 01/07/73 | 08/01/81 | 13.06 | 2.42 | 17.54 | 3.66 | -13.32 | 18.38 | 4.45 | 1.55 | 7.04 |
| PM2.5 | 37173 | 492 | 1,697 | 01/07/75 | 01/01/80 | 14.45 | 4.93 | 30.56 | 10.89 | -7.85 | 22.29 | 3.37 | 1.26 | 6.05 |
| PM2.5 | 48013 | 1,537 | 2,835 | 01/07/73 | 01/01/81 | 14.01 | 5.18 | 64.96 | 21.02 | 5.25 | 30.94 | 3.64 | 1.16 | 6.13 |
| PM2.5 | 30093 | 7,515 | 5,694 | 01/07/73 | 12/31/88 | 8.85 | 3.18 | 27.13 | 2.04 | -17.28 | 18.47 | 4.17 | 1.49 | 6.95 |
| PM2.5 | 47105 | 1,226 | 1,697 | 01/07/75 | 01/01/80 | 7.70 | 3.37 | 16.77 | 14.30 | -3.76 | 26.63 | 3.24 | 1.12 | 5.65 |
| PM2.5 | 41023 | 455 | 2,667 | 01/07/73 | 12/17/80 | 6.92 | 2.48 | 19.92 | 6.11 | -10.00 | 22.10 | 3.82 | 1.27 | 6.76 |
| PM2.5 | 53047 | 3,887 | 5,566 | 01/07/73 | 12/31/88 | 11.47 | 6.40 | 18.73 | 4.56 | -12.96 | 21.06 | 3.35 | 1.10 | 5.99 |
| PM2.5 | 08045 | 545 | 1,415 | 01/07/73 | 03/06/78 | 4.05 | 1.81 | 7.01 | 3.62 | -14.03 | 19.84 | 4.38 | 1.71 | 6.74 |
| PM2.5 | 53009 | 2,222 | 2,075 | 01/07/73 | 08/01/81 | 3.81 | 2.52 | 8.21 | 8.06 | -1.19 | 17.34 | 2.14 | 0.75 | 4.01 |
| PM2.5 | 53071 | 2,908 | 2,546 | 01/07/73 | 12/03/85 | 10.03 | 4.44 | 22.30 | 11.77 | -5.44 | 27.83 | 3.77 | 1.26 | 6.79 |
| PM2.5 | 40081 | 1,983 | 2,998 | 01/07/73 | 08/01/81 | 7.40 | 0.10 | 14.90 | 16.41 | -3.51 | 31.60 | 3.38 | 1.03 | 5.91 |
| PM2.5 | 53013 | 405 | 3,274 | 01/07/73 | 08/10/86 | 13.94 | 2.52 | 20.05 | 9.22 | -7.12 | 25.35 | 3.49 | 1.07 | 6.40 |
| PM2.5 | 25015 | 5,746 | 2,267 | 01/07/75 | 08/01/81 | 18.12 | 2.19 | 44.10 | 8.17 | -12.87 | 24.15 | 3.22 | 1.03 | 5.92 |
| PM2.5 | 06105 | 726 | 3,006 | 01/07/73 | 12/31/88 | 24.00 | 5.08 | 59.09 | 10.57 | -2.16 | 25.16 | 4.26 | 0.82 | 7.03 |
| PM2.5 | 06063 | 2,098 | 5,502 | 01/07/73 | 12/31/88 | 14.30 | 6.70 | 32.02 | 8.22 | -6.54 | 23.54 | 4.17 | 1.02 | 6.66 |
| PM2.5 | 48273 | 1,451 | 2,682 | 01/14/73 | 01/01/81 | 11.47 | 4.79 | 25.82 | 22.65 | 9.02 | 29.99 | 2.83 | 1.04 | 5.04 |
| PM2.5 | 24027 | 995 | 992 | 06/07/73 | 08/01/81 | 13.42 | 7.40 | 21.96 | 12.21 | -5.91 | 26.90 | 2.94 | 1.03 | 5.20 |
| PM2.5 | 53051 | 395 | 2,269 | 01/07/73 | 12/08/80 | 2.27 | 0.74 | 5.77 | 6.37 | -12.80 | 23.14 | 3.31 | 1.01 | 6.25 |
| PM2.5 | 53003 | 1,307 | 3,274 | 01/07/73 | 08/10/86 | 10.33 | 3.10 | 29.03 | 8.52 | -8.03 | 25.13 | 3.60 | 1.12 | 6.55 |
| PM2.5 | 31157 | 1,256 | 1,265 | 01/07/73 | 08/01/81 | 4.01 | 1.95 | 8.20 | 9.77 | -10.27 | 26.93 | 4.29 | 1.13 | 7.19 |
| PM2.5 | 15001 | 4,508 | 3,003 | 01/07/73 | 08/01/81 | 8.88 | 5.82 | 11.43 | 16.84 | 14.03 | 19.14 | 2.13 | 1.23 | 2.84 |
| PM2.5 | 51069 | 794 | 1,291 | 01/07/73 | 11/11/77 | 11.36 | 3.64 | 24.62 | 12.25 | -4.53 | 25.50 | 3.32 | 0.93 | 6.19 |
| PM2.5 | 42089 | 2,207 | 1,407 | 01/07/73 | 05/19/78 | 9.24 | 2.50 | 25.18 | 9.23 | -9.14 | 23.47 | 2.95 | 0.98 | 5.52 |
| PM2.5 | 02110 | 584 | 2,844 | 01/07/73 | 01/31/86 | 11.16 | 2.59 | 32.89 | -1.34 | -20.89 | 10.52 | 1.25 | 0.30 | 3.13 |
| PM2.5 | 48243 | 64 | 2,760 | 09/02/77 | 12/29/88 | 6.92 | 2.55 | 9.12 | 16.06 | 1.43 | 27.40 | 4.18 | 1.71 | 6.49 |
| PM2.5 | 41037 | 218 | 1,381 | 11/18/83 | 12/31/88 | 13.70 | 13.61 | 13.61 | 8.38 | -8.65 | 24.21 | 3.93 | 1.22 | 6.22 |
| PM2.5 | 27017 | 729 | 1,037 | 01/07/78 | 01/01/81 | 7.04 | 2.97 | 18.21 | 4.29 | -21.96 | 22.73 | 3.05 | 0.85 | 6.13 |
| PM2.5 | 53045 | 1,272 | 2,534 | 01/07/73 | 08/01/81 | 6.10 | 1.37 | 13.93 | 9.09 | -0.98 | 20.21 | 2.62 | 0.71 | 5.42 |
| PM2.5 | 38053 | 277 | 2,004 | 01/07/76 | 06/01/84 | 8.70 | 2.90 | 19.66 | 5.90 | -22.64 | 24.82 | 3.60 | 1.08 | 6.80 |
| PM2.5 | 46127 | 271 | 1,275 | 01/07/73 | 08/01/81 | 9.16 | 2.33 | 27.01 | 9.87 | -12.84 | 27.84 | 3.45 | 0.95 | 6.32 |
| PM2.5 | 01119 | 328 | 1,069 | 01/07/73 | 01/01/81 | 9.88 | 5.38 | 19.12 | 18.11 | 1.01 | 29.90 | 3.32 | 0.95 | 5.51 |
| PM2.5 | 41063 | 547 | 3,274 | 01/07/73 | 08/10/86 | 11.39 | 3.49 | 21.60 | 5.38 | -11.02 | 21.98 | 3.55 | 1.06 | 6.45 |
| PM2.5 | 41025 | 441 | 2,667 | 01/07/73 | 12/17/80 | 11.56 | 6.14 | 23.08 | 8.03 | -10.23 | 25.04 | 3.76 | 1.33 | 6.02 |
| PM2.5 | 06043 | 372 | 1,463 | 09/07/72 | 01/01/77 | 14.88 | 5.12 | 32.80 | 11.77 | -0.82 | 24.60 | 4.41 | 1.38 | 6.65 |
| PM2.5 | 30081 | 1,403 | 2,816 | 01/07/73 | 08/01/81 | 11.10 | 3.20 | 23.54 | 3.26 | -14.59 | 19.02 | 3.89 | 1.35 | 6.71 |
| PM2.5 | 46033 | 445 | 3,031 | 01/07/73 | 08/01/81 | 7.42 | 3.33 | 12.02 | 7.66 | -14.87 | 25.30 | 3.97 | 1.22 | 6.74 |
| PM2.5 | 30087 | 523 | 3,022 | 01/07/73 | 08/01/81 | 5.49 | 2.37 | 14.85 | 8.12 | -16.76 | 26.73 | 3.92 | 1.25 | 6.87 |
| PM2.5 | 27031 | 318 | 3,133 | 07/07/77 | 12/31/88 | 5.38 | 3.11 | 9.71 | 4.13 | -21.87 | 21.36 | 2.30 | 0.68 | 4.65 |
| PM2.5 | 16009 | 584 | 3,274 | 01/07/73 | 08/10/86 | 8.33 | 3.20 | 24.80 | 7.23 | -9.81 | 23.75 | 3.46 | 0.90 | 6.69 |
| PM2.5 | 08103 | 172 | 1,415 | 01/07/73 | 03/06/78 | 8.79 | 6.41 | 14.67 | 4.33 | -15.03 | 21.88 | 4.33 | 1.61 | 6.89 |
| O3 | 00036 | 661,159 | 3,219 | 01/07/80 | 12/31/88 | 33.59 | 10.36 | 72.00 | 11.59 | -7.44 | 26.85 | 2.78 | 0.87 | 5.10 |
| O3 | 04005 | 1,056 | 850 | 05/07/80 | 12/31/88 | 39.51 | 7.00 | 66.00 | 18.00 | 0.14 | 26.52 | 4.36 | 2.16 | 6.00 |
| O3 | 04013 | 129,142 | 3,282 | 01/07/80 | 12/31/88 | 40.48 | 15.75 | 67.57 | 21.47 | 6.91 | 35.07 | 4.51 | 1.84 | 6.65 |
| O3 | 06001 | 85,807 | 3,219 | 01/07/80 | 12/31/88 | 31.86 | 12.75 | 56.03 | 14.24 | 5.21 | 24.79 | 3.86 | 1.31 | 6.51 |
| O3 | 06067 | 61,291 | 3,219 | 01/07/80 | 12/31/88 | 47.84 | 13.50 | 97.91 | 15.82 | 4.93 | 27.36 | 4.31 | 1.17 | 7.04 |
| O3 | 06073 | 134,528 | 3,219 | 01/07/80 | 12/31/88 | 51.03 | 23.70 | 89.09 | 16.88 | 6.52 | 27.61 | 3.89 | 1.35 | 5.68 |
| O3 | 06075 | 17,900 | 798 | 01/07/80 | 08/05/85 | 57.84 | 22.93 | 110.00 | 12.79 | 6.91 | 18.84 | 2.59 | 0.92 | 4.83 |
| O3 | 06083 | 22,213 | 3,219 | 01/07/80 | 12/31/88 | 39.94 | 19.18 | 66.86 | 14.91 | 5.94 | 24.68 | 3.41 | 1.19 | 5.24 |
| O3 | 08001 | 8,342 | 2,631 | 01/07/80 | 12/31/88 | 52.96 | 16.00 | 106.00 | 9.50 | -11.32 | 25.44 | 4.55 | 1.45 | 7.28 |
| O3 | 08013 | 7,837 | 3,134 | 01/07/80 | 12/31/88 | 42.83 | 16.50 | 74.34 | 4.21 | -12.66 | 18.51 | 4.50 | 1.42 | 7.17 |
| O3 | 09005 | 5,350 | 1,621 | 04/08/80 | 11/21/88 | 62.00 | 21.00 | 139.50 | 16.15 | 4.14 | 25.40 | 3.19 | 1.03 | 5.29 |
| O3 | 12031 | 46,306 | 3,016 | 01/07/80 | 12/31/88 | 35.29 | 14.50 | 67.00 | 20.31 | 6.90 | 28.56 | 2.71 | 0.92 | 4.88 |
| O3 | 12099 | 68,445 | 3,058 | 01/07/80 | 12/31/88 | 36.12 | 14.00 | 68.00 | 23.22 | 13.81 | 28.18 | 2.31 | 1.07 | 4.11 |
| O3 | 17031 | 423,928 | 3,219 | 01/07/80 | 12/31/88 | 31.91 | 10.72 | 58.29 | 9.85 | -13.48 | 27.38 | 2.59 | 0.74 | 5.35 |
| O3 | 17091 | 516 | 230 | 04/13/81 | 09/30/82 | 53.31 | 12.00 | 111.55 | 19.14 | 7.71 | 26.71 | 3.33 | 1.48 | 5.65 |
| O3 | 17097 | 3,521 | 441 | 01/07/80 | 06/08/81 | 36.49 | 7.00 | 71.33 | 8.66 | -13.30 | 26.32 | 2.30 | 0.68 | 5.07 |
| O3 | 17201 | 18,032 | 2,847 | 01/09/80 | 12/31/88 | 35.88 | 8.00 | 75.50 | 10.02 | -14.20 | 27.14 | 3.05 | 0.84 | 5.77 |
| O3 | 18089 | 36,722 | 2,842 | 05/27/80 | 10/31/88 | 37.92 | 11.76 | 80.24 | 11.06 | -12.51 | 27.35 | 2.89 | 0.80 | 5.72 |
| O3 | 19153 | 8,505 | 955 | 01/07/80 | 11/12/86 | 28.89 | 3.00 | 71.15 | 12.75 | -11.86 | 27.66 | 3.15 | 0.97 | 6.00 |
| O3 | 20133 | 179 | 318 | 02/11/88 | 12/31/88 | 49.83 | 31.00 | 75.30 | 16.35 | -1.21 | 30.28 | 3.37 | 1.16 | 6.08 |
| O3 | 21111 | 60,540 | 2,901 | 01/07/80 | 12/31/88 | 41.80 | 7.00 | 86.00 | 14.00 | -6.68 | 28.11 | 3.34 | 0.98 | 6.14 |
| O3 | 22071 | 54,064 | 2,837 | 10/06/80 | 12/30/88 | 36.68 | 7.00 | 81.00 | 20.35 | 6.31 | 28.95 | 2.04 | 0.76 | 3.79 |
| O3 | 22073 | 5,777 | 1,569 | 05/06/81 | 10/31/88 | 52.44 | 16.20 | 90.00 | 23.03 | 7.14 | 30.29 | 3.26 | 1.15 | 5.27 |
| O3 | 24003 | 5,077 | 1,023 | 01/07/80 | 11/06/88 | 27.19 | 0.00 | 73.45 | 14.92 | -5.30 | 28.35 | 2.62 | 0.88 | 4.68 |
| O3 | 24033 | 11,480 | 1,403 | 01/07/80 | 04/21/85 | 38.62 | 4.00 | 92.97 | 11.95 | -5.61 | 27.77 | 2.79 | 0.85 | 5.06 |
| O3 | 24510 | 97,248 | 3,041 | 01/07/80 | 12/31/88 | 41.09 | 5.50 | 95.50 | 12.90 | -5.58 | 27.67 | 2.87 | 0.91 | 5.20 |
| O3 | 25009 | 30,580 | 1,916 | 01/07/80 | 09/29/88 | 46.29 | 14.00 | 80.00 | 13.54 | -5.69 | 25.45 | 2.42 | 0.68 | 4.62 |
| O3 | 26049 | 3,092 | 344 | 05/04/80 | 10/31/81 | 45.34 | 14.57 | 90.85 | 17.12 | 4.60 | 25.99 | 3.10 | 1.08 | 5.47 |
| O3 | 26125 | 69,040 | 3,111 | 01/07/80 | 12/27/88 | 35.51 | 8.67 | 73.00 | 9.19 | -12.87 | 25.69 | 2.85 | 0.85 | 5.64 |
| O3 | 26163 | 68,193 | 1,091 | 01/07/80 | 09/19/86 | 26.73 | 1.00 | 67.94 | 9.08 | -13.47 | 25.21 | 2.66 | 0.86 | 5.12 |
| O3 | 29047 | 6,943 | 2,466 | 01/07/80 | 11/30/88 | 41.02 | 10.00 | 70.79 | 15.93 | -5.46 | 30.34 | 3.16 | 0.97 | 5.66 |
| O3 | 31055 | 26,760 | 2,234 | 01/07/80 | 10/31/88 | 38.47 | 12.00 | 76.00 | 14.63 | -10.25 | 29.38 | 3.33 | 0.95 | 6.08 |
| O3 | 34001 | 10,732 | 1,428 | 04/09/80 | 03/31/85 | 33.62 | 1.00 | 69.00 | 11.02 | -6.71 | 26.30 | 2.48 | 0.91 | 4.46 |
| O3 | 34013 | 78,867 | 3,075 | 01/07/80 | 12/31/88 | 41.27 | 5.50 | 105.00 | 11.29 | -8.45 | 26.63 | 3.00 | 0.92 | 5.45 |
| O3 | 36055 | 54,904 | 3,184 | 01/07/80 | 12/31/88 | 30.25 | 8.00 | 67.71 | 8.71 | -11.55 | 24.78 | 2.53 | 0.70 | 5.30 |
| O3 | 37119 | 32,211 | 2,990 | 01/07/80 | 12/31/88 | 37.58 | 5.73 | 79.00 | 15.99 | -1.28 | 28.96 | 3.37 | 0.99 | 5.84 |
| O3 | 39017 | 16,817 | 2,850 | 01/07/80 | 12/31/88 | 38.09 | 13.00 | 76.00 | 12.56 | -7.76 | 27.14 | 3.10 | 0.89 | 5.79 |
| O3 | 39023 | 5,862 | 1,539 | 06/11/80 | 11/02/88 | 36.82 | 11.00 | 74.00 | 14.95 | -5.05 | 26.51 | 3.26 | 1.08 | 5.93 |
| O3 | 39035 | 129,167 | 2,835 | 01/07/80 | 10/31/88 | 39.53 | 9.50 | 85.58 | 11.04 | -11.19 | 25.92 | 2.57 | 0.65 | 5.41 |
| O3 | 39061 | 73,840 | 2,849 | 01/07/80 | 10/31/88 | 32.54 | 5.25 | 77.92 | 13.35 | -8.61 | 27.68 | 3.17 | 0.94 | 5.86 |
| O3 | 40109 | 36,483 | 2,065 | 01/07/80 | 10/31/88 | 40.88 | 10.00 | 89.00 | 17.13 | -3.46 | 31.06 | 3.33 | 1.04 | 5.87 |
| O3 | 42003 | 133,981 | 2,796 | 01/07/80 | 10/31/88 | 27.99 | 4.00 | 57.50 | 11.39 | -9.79 | 25.18 | 3.17 | 0.95 | 6.04 |
| O3 | 42021 | 8,138 | 1,575 | 01/07/80 | 07/09/86 | 35.93 | 7.00 | 75.00 | 8.25 | -11.03 | 22.59 | 2.98 | 0.85 | 5.71 |
| O3 | 42045 | 48,103 | 3,182 | 01/07/80 | 12/31/88 | 36.02 | 6.50 | 85.58 | 11.95 | -7.13 | 27.10 | 3.01 | 1.00 | 5.35 |
| O3 | 42129 | 8,697 | 917 | 04/06/81 | 07/15/84 | 40.64 | 13.90 | 82.10 | 10.36 | -10.43 | 24.14 | 3.06 | 0.90 | 5.93 |
| O3 | 45019 | 9,400 | 1,114 | 06/16/82 | 10/31/88 | 49.08 | 17.00 | 89.00 | 24.05 | 15.02 | 29.19 | 2.10 | 0.71 | 3.76 |
| O3 | 45087 | 571 | 861 | 05/11/83 | 10/31/87 | 42.86 | 11.50 | 92.00 | 22.48 | 12.38 | 29.63 | 3.42 | 1.16 | 5.67 |
| O3 | 47065 | 26,082 | 3,134 | 01/07/80 | 12/31/88 | 43.63 | 14.00 | 83.00 | 14.76 | -2.09 | 27.79 | 3.27 | 0.97 | 5.69 |
| O3 | 47093 | 26,471 | 2,467 | 01/07/80 | 10/31/88 | 41.12 | 8.00 | 82.00 | 15.10 | -3.31 | 27.33 | 3.42 | 1.15 | 5.98 |
| O3 | 48201 | 160,672 | 3,219 | 01/07/80 | 12/31/88 | 41.88 | 17.50 | 71.56 | 20.30 | 5.51 | 29.94 | 3.07 | 1.05 | 5.50 |
| O3 | 48439 | 51,862 | 2,533 | 01/07/80 | 10/30/88 | 44.11 | 15.00 | 85.85 | 18.85 | 0.57 | 32.44 | 3.36 | 0.99 | 5.93 |
| O3 | 49035 | 36,347 | 3,027 | 01/07/80 | 12/31/88 | 41.90 | 13.50 | 80.39 | 7.36 | -9.71 | 23.62 | 3.87 | 1.06 | 6.82 |
| O3 | 55079 | 57,407 | 2,150 | 01/07/80 | 11/24/88 | 42.08 | 14.00 | 84.00 | 13.21 | -10.88 | 26.95 | 2.00 | 0.61 | 4.01 |
| O3 | 55105 | 5,630 | 1,885 | 04/06/80 | 10/31/88 | 38.83 | 12.00 | 72.00 | 16.19 | -2.09 | 27.40 | 3.17 | 0.99 | 5.70 |
| O3 | 06037 | 555,305 | 3,219 | 01/07/80 | 12/31/88 | 52.10 | 12.43 | 117.50 | 16.00 | 4.37 | 27.81 | 4.25 | 1.45 | 6.26 |
| O3 | 06047 | 450 | 215 | 09/06/81 | 10/27/82 | 17.16 | 3.00 | 40.30 | 18.12 | 7.33 | 27.49 | 4.24 | 1.63 | 6.21 |
| O3 | 06053 | 8,627 | 1,617 | 01/07/80 | 09/27/86 | 34.48 | 15.00 | 66.80 | 14.35 | 5.89 | 25.32 | 3.86 | 1.24 | 5.90 |
| O3 | 06059 | 111,807 | 3,219 | 01/07/80 | 12/31/88 | 41.70 | 11.17 | 91.83 | 16.80 | 8.90 | 25.93 | 3.37 | 1.12 | 6.11 |
| O3 | 06065 | 59,665 | 3,219 | 01/07/80 | 12/31/88 | 52.69 | 18.46 | 116.33 | 19.65 | 6.61 | 32.47 | 4.26 | 1.83 | 6.05 |
| O3 | 06095 | 14,816 | 3,175 | 01/07/80 | 12/31/88 | 31.49 | 6.18 | 62.44 | 15.41 | 5.76 | 25.93 | 4.23 | 1.25 | 7.21 |
| O3 | 06111 | 28,015 | 3,219 | 01/07/80 | 12/31/88 | 47.11 | 20.00 | 85.80 | 14.94 | 4.33 | 25.94 | 3.97 | 1.35 | 6.05 |
| O3 | 12025 | 159,490 | 3,138 | 01/07/80 | 12/31/88 | 43.56 | 16.00 | 98.50 | 23.63 | 14.32 | 28.28 | 2.40 | 1.18 | 4.47 |
| O3 | 17089 | 20,862 | 3,124 | 01/10/80 | 12/31/88 | 35.09 | 7.00 | 81.00 | 9.65 | -14.38 | 26.97 | 3.04 | 0.87 | 5.91 |
| O3 | 18003 | 24,673 | 3,150 | 01/07/80 | 12/31/88 | 34.97 | 12.50 | 69.00 | 10.13 | -12.21 | 26.28 | 3.14 | 0.90 | 6.03 |
| O3 | 21089 | 1,856 | 2,281 | 10/08/81 | 12/31/88 | 41.52 | 11.00 | 84.00 | 13.09 | -5.21 | 27.38 | 3.21 | 0.99 | 5.97 |
| O3 | 22051 | 19,525 | 2,207 | 09/06/82 | 12/31/88 | 39.76 | 7.00 | 78.92 | 20.48 | 6.55 | 28.65 | 2.01 | 0.75 | 3.83 |
| O3 | 22055 | 3,477 | 882 | 10/06/83 | 10/31/88 | 40.14 | 6.03 | 84.00 | 23.80 | 10.94 | 28.68 | 2.89 | 1.22 | 5.17 |
| O3 | 24015 | 3,659 | 2,616 | 01/07/80 | 12/31/88 | 39.45 | 6.00 | 95.00 | 12.18 | -5.78 | 27.53 | 2.76 | 0.93 | 4.94 |
| O3 | 25003 | 1,546 | 350 | 01/07/80 | 09/25/83 | 32.00 | 0.00 | 89.28 | 9.55 | -14.28 | 23.80 | 3.24 | 0.77 | 5.57 |
| O3 | 25025 | 27,275 | 987 | 06/06/80 | 10/31/87 | 34.40 | 5.00 | 77.00 | 11.09 | -8.14 | 24.95 | 2.69 | 0.77 | 4.88 |
| O3 | 25027 | 48,843 | 2,866 | 04/06/80 | 12/31/88 | 36.95 | 9.00 | 74.00 | 9.72 | -10.61 | 25.24 | 3.12 | 0.93 | 5.51 |
| O3 | 31109 | 7,494 | 1,520 | 01/07/80 | 12/31/88 | 48.45 | 11.00 | 106.03 | 10.79 | -11.78 | 28.97 | 3.45 | 0.90 | 6.33 |
| O3 | 35001 | 30,215 | 3,057 | 01/07/80 | 12/31/88 | 40.56 | 7.00 | 91.17 | 12.04 | -3.96 | 25.49 | 4.17 | 1.79 | 6.21 |
| O3 | 36029 | 86,198 | 2,918 | 01/31/80 | 12/31/88 | 43.51 | 24.00 | 66.00 | 8.48 | -12.62 | 24.08 | 2.49 | 0.67 | 5.23 |
| O3 | 36117 | 3,374 | 2,232 | 01/07/80 | 11/20/86 | 33.52 | 5.00 | 78.00 | 8.58 | -11.97 | 24.23 | 2.52 | 0.71 | 5.22 |
| O3 | 36119 | 68,146 | 2,961 | 01/07/80 | 12/31/88 | 36.31 | 6.00 | 85.50 | 11.33 | -8.11 | 26.10 | 2.80 | 0.87 | 5.11 |
| O3 | 42017 | 4,830 | 524 | 01/07/80 | 01/09/82 | 21.66 | 1.00 | 64.92 | 11.45 | -9.21 | 26.41 | 3.14 | 1.11 | 5.56 |
| O3 | 42071 | 25,228 | 2,819 | 01/07/80 | 12/31/88 | 40.19 | 4.00 | 104.00 | 11.69 | -7.62 | 26.94 | 3.01 | 0.96 | 5.43 |
| O3 | 42081 | 10,035 | 2,929 | 01/07/80 | 12/31/88 | 36.97 | 7.00 | 86.00 | 8.49 | -11.70 | 24.20 | 3.09 | 0.90 | 5.82 |
| O3 | 42125 | 6,373 | 1,068 | 01/07/80 | 11/14/88 | 33.22 | 1.00 | 86.00 | 14.90 | -4.14 | 24.94 | 3.28 | 1.10 | 5.89 |
| O3 | 45003 | 200 | 121 | 05/23/85 | 09/27/85 | 43.99 | 21.00 | 75.00 | 24.94 | 19.76 | 30.17 | 3.37 | 1.20 | 5.57 |
| O3 | 47037 | 14,058 | 874 | 01/07/80 | 12/05/83 | 45.52 | 11.91 | 82.00 | 17.26 | -1.07 | 29.37 | 3.33 | 0.98 | 5.63 |
| O3 | 47157 | 79,333 | 3,219 | 01/07/80 | 12/31/88 | 33.98 | 8.79 | 69.89 | 16.32 | -2.68 | 30.07 | 3.12 | 0.97 | 5.47 |
| O3 | 49049 | 8,045 | 2,651 | 01/07/80 | 12/11/88 | 38.88 | 13.00 | 71.00 | 6.92 | -10.47 | 23.28 | 3.90 | 1.20 | 6.57 |
| O3 | 54025 | 2,762 | 2,719 | 04/24/80 | 12/31/88 | 30.65 | 0.00 | 72.00 | 10.10 | -8.78 | 22.95 | 3.13 | 0.87 | 5.89 |
| O3 | 08031 | 51,023 | 3,030 | 01/13/80 | 12/31/88 | 53.56 | 15.00 | 120.14 | 8.18 | -11.05 | 23.70 | 4.97 | 1.42 | 7.91 |
| O3 | 09001 | 54,938 | 3,046 | 01/07/80 | 12/31/88 | 38.77 | 12.00 | 82.33 | 10.70 | -8.78 | 25.81 | 2.73 | 0.89 | 4.87 |
| O3 | 09003 | 62,770 | 3,057 | 01/07/80 | 12/31/88 | 42.75 | 7.00 | 96.30 | 9.77 | -9.52 | 25.25 | 3.16 | 0.96 | 5.56 |
| O3 | 11001 | 56,741 | 2,338 | 01/07/80 | 10/17/88 | 38.22 | 1.00 | 100.00 | 13.86 | -5.54 | 28.45 | 3.28 | 0.96 | 5.96 |
| O3 | 12011 | 101,606 | 3,033 | 01/07/80 | 12/31/88 | 40.03 | 5.00 | 85.00 | 23.47 | 13.84 | 28.20 | 2.64 | 1.28 | 4.79 |
| O3 | 12033 | 19,186 | 3,018 | 01/07/80 | 12/31/88 | 40.17 | 15.00 | 73.00 | 19.40 | 4.23 | 28.78 | 3.00 | 1.02 | 5.18 |
| O3 | 12103 | 110,752 | 3,211 | 01/07/80 | 12/31/88 | 35.51 | 16.67 | 64.33 | 22.04 | 11.12 | 28.51 | 1.64 | 0.61 | 3.17 |
| O3 | 15003 | 8,836 | 718 | 06/19/80 | 11/30/88 | 49.62 | 12.93 | 100.08 | 23.81 | 20.56 | 25.76 | 1.33 | 0.79 | 1.77 |
| O3 | 17001 | 5,007 | 2,048 | 07/29/80 | 11/30/88 | 47.17 | 11.00 | 86.00 | 16.56 | -2.26 | 29.47 | 3.19 | 0.99 | 5.71 |
| O3 | 22017 | 4,583 | 494 | 05/04/81 | 10/31/83 | 52.27 | 22.32 | 83.67 | 24.02 | 13.04 | 30.61 | 3.22 | 1.30 | 4.96 |
| O3 | 22057 | 1,083 | 767 | 11/21/83 | 10/18/88 | 48.01 | 10.00 | 98.85 | 23.17 | 8.87 | 28.43 | 2.20 | 0.87 | 4.10 |
| O3 | 26099 | 19,226 | 1,868 | 06/22/80 | 10/31/88 | 37.35 | 8.00 | 80.00 | 14.57 | -8.52 | 26.73 | 2.71 | 0.91 | 5.11 |
| O3 | 27109 | 1,004 | 238 | 01/07/80 | 09/30/80 | 33.29 | 8.00 | 62.08 | 9.49 | -18.01 | 25.91 | 3.22 | 1.05 | 6.62 |
| O3 | 28049 | 7,515 | 781 | 01/07/80 | 10/01/85 | 41.34 | 10.00 | 70.50 | 24.19 | 7.55 | 30.04 | 3.43 | 1.41 | 5.46 |
| O3 | 29095 | 2,183 | 112 | 06/14/88 | 10/03/88 | 57.16 | 29.65 | 82.22 | 24.95 | 16.84 | 30.94 | 3.37 | 1.36 | 5.28 |
| O3 | 33015 | 7,696 | 2,536 | 02/06/80 | 12/31/88 | 44.24 | 18.00 | 74.00 | 9.22 | -10.71 | 25.39 | 2.97 | 0.84 | 5.46 |
| O3 | 36063 | 4,982 | 874 | 01/07/80 | 12/31/82 | 34.63 | 9.00 | 81.00 | 9.29 | -11.57 | 23.78 | 2.23 | 0.66 | 4.41 |
| O3 | 36083 | 3,593 | 998 | 01/07/80 | 06/08/83 | 33.39 | 8.00 | 71.07 | 6.69 | -15.06 | 22.55 | 3.13 | 0.92 | 5.79 |
| O3 | 36093 | 1,629 | 331 | 01/07/80 | 12/30/80 | 31.86 | 5.00 | 81.00 | 7.66 | -13.71 | 24.03 | 3.24 | 1.11 | 6.04 |
| O3 | 37117 | 569 | 1,074 | 05/19/83 | 10/31/88 | 46.38 | 21.82 | 96.00 | 21.97 | 11.54 | 29.25 | 3.15 | 1.14 | 5.48 |
| O3 | 41039 | 16,230 | 2,809 | 01/07/80 | 12/22/88 | 41.60 | 12.60 | 77.00 | 8.52 | -2.58 | 20.97 | 3.37 | 0.89 | 6.71 |
| O3 | 42101 | 183,640 | 3,212 | 01/07/80 | 12/31/88 | 38.43 | 6.55 | 94.00 | 12.06 | -6.98 | 27.35 | 3.11 | 1.03 | 5.60 |
| O3 | 48135 | 3,757 | 1,609 | 01/07/80 | 12/28/88 | 35.82 | 10.00 | 81.00 | 17.55 | 0.61 | 29.24 | 4.29 | 1.62 | 7.08 |
| O3 | 48141 | 20,818 | 2,507 | 01/07/80 | 12/21/88 | 35.90 | 8.00 | 83.00 | 17.00 | 2.27 | 30.01 | 4.32 | 1.80 | 6.47 |
| O3 | 48355 | 20,424 | 3,178 | 01/07/80 | 12/31/88 | 40.12 | 10.67 | 86.00 | 22.20 | 8.34 | 29.93 | 2.75 | 1.01 | 4.90 |
| O3 | 51013 | 9,184 | 2,422 | 01/07/80 | 12/31/88 | 40.71 | 6.00 | 84.47 | 14.60 | -5.10 | 28.62 | 3.29 | 1.07 | 5.80 |
| O3 | 51810 | 5,539 | 1,843 | 01/07/80 | 10/31/88 | 40.58 | 5.00 | 91.00 | 19.34 | 2.36 | 28.43 | 2.27 | 0.72 | 4.41 |
| O3 | 53061 | 12,215 | 2,109 | 01/07/80 | 10/14/86 | 26.53 | 1.00 | 58.00 | 6.47 | -6.67 | 19.33 | 2.73 | 0.62 | 5.93 |
| O3 | 53063 | 25,824 | 2,719 | 01/07/80 | 10/31/88 | 23.85 | 0.50 | 56.50 | 9.39 | -10.89 | 25.53 | 3.67 | 1.23 | 6.58 |
| O3 | 01073 | 58,217 | 2,553 | 07/01/80 | 11/30/88 | 44.78 | 13.33 | 87.00 | 19.05 | 2.82 | 29.59 | 3.35 | 1.08 | 5.63 |
| O3 | 01097 | 27,427 | 2,911 | 02/27/80 | 12/31/88 | 41.58 | 12.00 | 73.50 | 19.32 | 4.42 | 28.75 | 2.83 | 0.96 | 4.83 |
| O3 | 06041 | 12,087 | 2,627 | 01/07/80 | 12/31/88 | 35.40 | 4.00 | 82.00 | 13.10 | 6.40 | 19.79 | 2.85 | 0.90 | 4.96 |
| O3 | 06071 | 76,232 | 3,219 | 01/07/80 | 12/31/88 | 53.78 | 20.00 | 113.91 | 18.62 | 4.05 | 32.41 | 4.18 | 1.93 | 5.77 |
| O3 | 08041 | 16,022 | 3,024 | 01/07/80 | 12/31/88 | 31.59 | 3.58 | 81.71 | 7.47 | -10.45 | 22.34 | 4.45 | 1.41 | 7.01 |
| O3 | 09013 | 134 | 126 | 04/13/80 | 09/15/80 | 66.68 | 20.12 | 160.62 | 18.41 | 6.49 | 26.46 | 3.61 | 1.19 | 5.77 |
| O3 | 10003 | 27,979 | 2,880 | 03/26/80 | 11/08/88 | 41.41 | 14.00 | 83.51 | 13.49 | -5.33 | 27.66 | 2.83 | 0.95 | 5.02 |
| O3 | 12117 | 12,250 | 3,120 | 01/07/80 | 12/31/88 | 39.81 | 16.00 | 75.00 | 21.56 | 9.44 | 28.42 | 2.80 | 1.11 | 4.99 |
| O3 | 18097 | 62,152 | 2,647 | 01/07/80 | 12/31/88 | 37.69 | 4.58 | 79.95 | 13.34 | -9.20 | 27.34 | 3.17 | 0.96 | 5.78 |
| O3 | 18127 | 5,078 | 2,363 | 05/03/81 | 12/31/88 | 52.86 | 25.51 | 94.97 | 11.73 | -10.58 | 27.49 | 2.84 | 0.76 | 5.59 |
| O3 | 20173 | 26,931 | 2,799 | 01/07/80 | 12/31/88 | 39.04 | 12.00 | 78.00 | 13.67 | -8.03 | 30.76 | 3.41 | 0.90 | 6.00 |
| O3 | 24005 | 40,939 | 3,067 | 01/07/80 | 12/28/88 | 42.37 | 5.67 | 102.56 | 12.27 | -6.54 | 27.22 | 2.88 | 0.92 | 5.14 |
| O3 | 26121 | 8,004 | 2,155 | 01/07/80 | 12/31/88 | 42.69 | 14.00 | 84.00 | 12.65 | -9.99 | 25.87 | 2.37 | 0.67 | 4.58 |
| O3 | 27123 | 30,918 | 2,652 | 01/07/80 | 12/31/88 | 38.10 | 10.00 | 71.00 | 8.20 | -18.79 | 26.16 | 2.92 | 0.80 | 5.84 |
| O3 | 27141 | 205 | 493 | 04/09/80 | 06/13/82 | 33.47 | 8.00 | 72.70 | 9.24 | -19.85 | 24.27 | 3.12 | 0.95 | 6.15 |
| O3 | 29189 | 73,615 | 3,219 | 01/07/80 | 12/31/88 | 36.25 | 11.33 | 72.59 | 13.15 | -8.12 | 29.26 | 3.15 | 0.90 | 5.85 |
| O3 | 29510 | 64,576 | 2,989 | 01/13/80 | 12/31/88 | 42.06 | 15.00 | 83.17 | 13.89 | -7.32 | 29.45 | 3.30 | 0.93 | 6.10 |
| O3 | 33011 | 19,362 | 2,842 | 01/07/80 | 12/31/88 | 39.52 | 16.00 | 69.50 | 7.91 | -12.69 | 24.91 | 3.16 | 0.85 | 5.77 |
| O3 | 34039 | 36,025 | 2,670 | 01/07/80 | 12/31/88 | 41.31 | 5.00 | 99.64 | 11.31 | -8.36 | 26.92 | 2.97 | 0.92 | 5.45 |
| O3 | 36067 | 37,262 | 2,917 | 03/19/80 | 12/31/88 | 35.09 | 10.45 | 77.03 | 9.20 | -11.74 | 24.11 | 2.75 | 0.80 | 5.64 |
| O3 | 39049 | 61,382 | 2,827 | 01/07/80 | 10/31/88 | 41.87 | 10.50 | 89.00 | 12.10 | -10.27 | 26.62 | 3.22 | 1.00 | 6.11 |
| O3 | 39081 | 6,597 | 2,787 | 01/07/80 | 10/31/88 | 40.47 | 8.00 | 94.00 | 11.57 | -9.76 | 25.45 | 3.16 | 0.91 | 5.98 |
| O3 | 39113 | 44,779 | 2,849 | 01/07/80 | 10/31/88 | 35.94 | 7.37 | 83.67 | 12.35 | -9.92 | 26.79 | 3.17 | 0.97 | 5.94 |
| O3 | 39151 | 29,683 | 2,879 | 07/20/80 | 12/31/88 | 38.78 | 4.00 | 99.00 | 10.09 | -10.76 | 25.48 | 3.05 | 0.85 | 6.07 |
| O3 | 44007 | 1,854 | 85 | 01/07/80 | 03/31/80 | 13.39 | 1.00 | 26.90 | -1.42 | -11.12 | 6.05 | 2.99 | 1.13 | 5.80 |
| O3 | 48113 | 111,681 | 3,189 | 01/07/80 | 12/31/88 | 38.98 | 11.00 | 67.38 | 18.96 | 1.27 | 32.19 | 3.28 | 0.98 | 5.80 |
| O3 | 48375 | 9,867 | 2,067 | 02/07/80 | 12/31/88 | 37.88 | 10.00 | 91.00 | 14.07 | -5.26 | 29.05 | 4.23 | 1.34 | 7.21 |
| O3 | 49003 | 110 | 204 | 08/06/85 | 09/09/86 | 33.71 | 11.07 | 72.00 | 18.44 | 5.57 | 26.97 | 3.36 | 1.19 | 4.79 |
| O3 | 49057 | 6,540 | 2,110 | 02/10/80 | 12/22/88 | 43.54 | 17.00 | 78.55 | 6.87 | -9.67 | 23.05 | 3.55 | 1.02 | 6.43 |
| O3 | 53033 | 93,624 | 3,169 | 01/07/80 | 12/31/88 | 38.04 | 5.50 | 86.00 | 7.44 | -4.89 | 20.11 | 2.77 | 0.72 | 6.09 |
| O3 | 53053 | 35,263 | 3,219 | 01/07/80 | 12/31/88 | 40.07 | 10.15 | 79.50 | 7.32 | -4.45 | 19.86 | 2.82 | 0.75 | 5.95 |
| O3 | 01055 | 1,658 | 584 | 01/07/80 | 10/02/81 | 36.89 | 8.00 | 93.27 | 17.02 | -0.40 | 29.70 | 3.34 | 1.02 | 5.54 |
| O3 | 06019 | 40,829 | 3,219 | 01/07/80 | 12/31/88 | 45.07 | 12.33 | 84.00 | 12.89 | 0.32 | 26.05 | 4.00 | 1.42 | 5.89 |
| O3 | 06061 | 8,027 | 2,592 | 01/07/80 | 12/31/88 | 51.23 | 17.00 | 100.00 | 11.66 | -1.27 | 24.90 | 4.02 | 0.86 | 6.59 |
| O3 | 12095 | 37,883 | 2,754 | 02/06/80 | 12/31/88 | 33.71 | 12.00 | 65.00 | 21.66 | 10.08 | 28.40 | 2.78 | 1.14 | 4.96 |
| O3 | 17163 | 20,089 | 2,852 | 01/07/80 | 12/31/88 | 31.83 | 6.00 | 72.00 | 13.39 | -7.41 | 29.42 | 3.11 | 0.89 | 5.69 |
| O3 | 20209 | 12,724 | 2,505 | 03/04/81 | 12/31/88 | 46.90 | 12.00 | 95.00 | 13.34 | -9.81 | 29.71 | 3.12 | 0.92 | 5.82 |
| O3 | 21167 | 724 | 1,878 | 06/25/80 | 10/31/88 | 48.33 | 8.00 | 100.00 | 17.46 | -1.46 | 28.05 | 3.37 | 1.09 | 5.91 |
| O3 | 21199 | 877 | 606 | 04/06/80 | 10/31/83 | 48.92 | 18.12 | 81.88 | 20.51 | 8.72 | 28.79 | 3.21 | 1.17 | 5.41 |
| O3 | 25021 | 16,203 | 1,338 | 06/06/80 | 10/31/88 | 46.08 | 13.00 | 103.58 | 16.71 | 5.14 | 26.35 | 2.86 | 0.81 | 4.91 |
| O3 | 25023 | 3,997 | 468 | 01/08/87 | 12/21/88 | 47.47 | 30.68 | 64.33 | 7.27 | -8.59 | 23.01 | 2.30 | 0.66 | 4.48 |
| O3 | 26081 | 20,293 | 1,824 | 01/07/80 | 10/31/88 | 41.59 | 14.19 | 85.42 | 13.91 | -9.83 | 26.30 | 3.09 | 1.00 | 5.62 |
| O3 | 27003 | 6,692 | 2,931 | 01/07/80 | 10/31/88 | 31.39 | 3.00 | 72.50 | 7.99 | -18.92 | 26.23 | 3.02 | 0.84 | 6.00 |
| O3 | 30013 | 2,122 | 1,090 | 02/26/80 | 10/31/88 | 36.25 | 1.00 | 77.00 | 12.67 | -5.32 | 24.65 | 4.40 | 1.27 | 7.32 |
| O3 | 34003 | 46,111 | 2,611 | 01/07/80 | 12/31/88 | 39.94 | 11.00 | 82.00 | 11.39 | -8.55 | 26.68 | 2.95 | 0.91 | 5.28 |
| O3 | 34025 | 11,168 | 840 | 06/02/80 | 09/30/83 | 31.39 | 3.00 | 97.03 | 13.34 | -5.77 | 27.26 | 2.64 | 0.81 | 4.76 |
| O3 | 36015 | 5,937 | 2,070 | 10/06/80 | 11/30/86 | 41.80 | 19.00 | 68.00 | 8.04 | -12.75 | 23.35 | 3.05 | 0.92 | 5.91 |
| O3 | 37081 | 24,540 | 2,954 | 01/13/80 | 12/31/88 | 37.23 | 10.00 | 84.17 | 14.44 | -2.84 | 28.17 | 3.32 | 0.98 | 5.79 |
| O3 | 39085 | 11,261 | 2,837 | 01/07/80 | 12/31/88 | 38.00 | 6.00 | 83.05 | 9.98 | -10.54 | 25.77 | 1.98 | 0.49 | 4.51 |
| O3 | 39093 | 14,915 | 2,842 | 01/07/80 | 10/31/88 | 37.03 | 3.00 | 88.00 | 11.28 | -11.34 | 26.19 | 2.55 | 0.70 | 5.40 |
| O3 | 41029 | 9,891 | 2,943 | 01/07/80 | 12/31/88 | 45.35 | 14.00 | 84.00 | 9.17 | -3.57 | 23.47 | 4.00 | 1.13 | 7.35 |
| O3 | 42007 | 7,252 | 1,744 | 05/04/80 | 10/31/88 | 42.97 | 1.00 | 91.33 | 15.44 | -2.73 | 25.68 | 3.29 | 1.04 | 5.94 |
| O3 | 42073 | 2,761 | 953 | 06/02/80 | 11/18/84 | 31.51 | 2.00 | 91.00 | 8.99 | -9.88 | 23.72 | 3.03 | 0.79 | 5.99 |
| O3 | 42079 | 30,540 | 2,556 | 10/06/80 | 12/31/88 | 40.52 | 11.00 | 78.00 | 9.99 | -9.29 | 24.58 | 3.03 | 0.89 | 5.61 |
| O3 | 45031 | 2,676 | 2,146 | 01/13/80 | 10/31/88 | 35.69 | 6.00 | 90.00 | 19.07 | 1.57 | 29.75 | 3.39 | 1.14 | 5.92 |
| O3 | 48061 | 6,762 | 1,651 | 02/17/80 | 10/31/88 | 39.23 | 5.00 | 79.00 | 24.68 | 12.89 | 29.70 | 2.32 | 1.07 | 3.97 |
| O3 | 55039 | 1,753 | 837 | 05/16/84 | 10/31/88 | 48.05 | 19.00 | 91.10 | 17.15 | 3.18 | 27.42 | 2.97 | 0.98 | 5.33 |
| O3 | 06099 | 22,798 | 3,176 | 01/13/80 | 12/31/88 | 51.46 | 10.69 | 114.50 | 16.32 | 4.97 | 28.87 | 4.38 | 1.43 | 6.77 |
| O3 | 13121 | 13,172 | 585 | 04/12/87 | 12/25/88 | 52.76 | 11.00 | 91.00 | 17.76 | 1.16 | 28.87 | 3.52 | 1.11 | 5.88 |
| O3 | 18163 | 20,257 | 3,063 | 01/09/80 | 12/31/88 | 42.24 | 13.00 | 79.45 | 13.88 | -6.70 | 28.92 | 3.09 | 0.94 | 5.71 |
| O3 | 25001 | 2,811 | 480 | 01/07/87 | 11/30/88 | 53.09 | 20.00 | 91.00 | 10.12 | -5.49 | 22.34 | 1.47 | 0.38 | 3.12 |
| O3 | 27075 | 700 | 2,718 | 04/06/80 | 10/31/88 | 34.35 | 6.00 | 77.50 | 4.83 | -22.68 | 22.33 | 2.60 | 0.76 | 5.20 |
| O3 | 32003 | 27,961 | 2,331 | 01/07/80 | 12/31/88 | 25.48 | 4.00 | 62.00 | 17.89 | 1.77 | 32.71 | 3.79 | 1.70 | 5.37 |
| O3 | 34007 | 31,060 | 2,676 | 02/04/80 | 10/31/88 | 33.17 | 7.00 | 80.00 | 14.01 | -5.70 | 27.61 | 3.04 | 1.01 | 5.35 |
| O3 | 36001 | 3,456 | 346 | 04/19/87 | 10/31/88 | 55.54 | 23.25 | 97.12 | 15.60 | 3.60 | 25.93 | 3.28 | 0.81 | 5.94 |
| O3 | 36103 | 82,411 | 2,908 | 01/07/80 | 12/31/88 | 30.35 | 2.00 | 94.00 | 11.56 | -5.77 | 25.47 | 1.88 | 0.64 | 3.66 |
| O3 | 37027 | 1,129 | 1,111 | 04/06/81 | 10/31/87 | 35.71 | 13.00 | 66.25 | 19.49 | 8.49 | 26.42 | 3.44 | 0.89 | 5.96 |
| O3 | 37051 | 2,002 | 493 | 05/11/81 | 05/14/84 | 35.40 | 7.00 | 75.00 | 22.52 | 11.44 | 29.79 | 3.36 | 1.23 | 5.75 |
| O3 | 37183 | 6,506 | 1,103 | 01/07/80 | 12/30/83 | 38.08 | 15.00 | 73.00 | 14.17 | -2.28 | 28.88 | 3.28 | 1.06 | 5.77 |
| O3 | 45079 | 24,159 | 2,719 | 04/09/80 | 12/18/88 | 45.52 | 15.00 | 81.00 | 18.17 | 2.19 | 29.60 | 3.35 | 1.06 | 5.85 |
| O3 | 47011 | 166 | 136 | 04/17/83 | 10/31/83 | 40.85 | 11.50 | 83.12 | 21.61 | 8.63 | 30.21 | 3.48 | 1.18 | 5.29 |
| O3 | 48029 | 67,598 | 2,813 | 01/07/80 | 10/31/88 | 40.98 | 8.00 | 80.00 | 21.04 | 4.94 | 30.41 | 3.48 | 1.14 | 5.88 |
| O3 | 51161 | 2,473 | 2,738 | 04/08/80 | 12/31/88 | 41.00 | 13.00 | 71.00 | 13.52 | -3.93 | 26.02 | 3.43 | 0.85 | 6.17 |
| O3 | 51510 | 6,791 | 2,889 | 01/07/80 | 12/31/88 | 34.97 | 3.00 | 90.80 | 13.38 | -4.67 | 28.17 | 3.13 | 0.95 | 5.65 |
| O3 | 55061 | 163 | 469 | 07/06/85 | 06/08/87 | 34.65 | 4.00 | 75.00 | 5.47 | -15.23 | 23.53 | 2.03 | 0.56 | 4.10 |
| O3 | 01099 | 171 | 375 | 05/04/80 | 10/30/82 | 37.06 | 7.35 | 70.00 | 20.78 | 5.27 | 30.03 | 3.61 | 1.48 | 5.63 |
| O3 | 01125 | 1,720 | 479 | 05/26/80 | 09/08/82 | 45.99 | 16.00 | 75.15 | 18.90 | 0.51 | 29.33 | 3.39 | 1.36 | 5.44 |
| O3 | 06077 | 27,199 | 3,034 | 01/07/80 | 12/31/88 | 32.94 | 5.00 | 76.17 | 15.74 | 5.00 | 27.20 | 4.27 | 1.31 | 6.81 |
| O3 | 06089 | 8,041 | 2,393 | 07/07/80 | 12/31/88 | 35.87 | 10.00 | 71.20 | 13.09 | -1.62 | 27.78 | 4.32 | 0.93 | 7.27 |
| O3 | 09009 | 42,249 | 1,977 | 04/22/80 | 11/02/88 | 52.77 | 16.20 | 115.50 | 14.96 | -3.33 | 25.91 | 2.77 | 0.90 | 4.78 |
| O3 | 17115 | 9,923 | 2,740 | 03/17/80 | 12/31/88 | 30.07 | 5.50 | 73.53 | 12.32 | -10.12 | 28.23 | 3.21 | 0.94 | 5.74 |
| O3 | 17167 | 19,420 | 3,129 | 01/07/80 | 12/31/88 | 35.17 | 9.00 | 73.00 | 11.90 | -11.31 | 28.38 | 3.19 | 0.92 | 5.92 |
| O3 | 22089 | 492 | 1,175 | 07/30/83 | 10/31/88 | 44.53 | 11.00 | 84.00 | 23.49 | 10.47 | 28.69 | 2.16 | 0.82 | 3.91 |
| O3 | 25005 | 35,385 | 3,040 | 01/07/80 | 12/31/88 | 35.59 | 5.50 | 77.00 | 9.81 | -8.71 | 24.94 | 2.56 | 0.81 | 4.62 |
| O3 | 39153 | 37,812 | 2,842 | 01/07/80 | 10/31/88 | 39.09 | 7.00 | 85.00 | 10.95 | -11.53 | 25.69 | 3.03 | 0.84 | 6.07 |
| O3 | 42085 | 8,466 | 2,338 | 03/15/81 | 11/27/88 | 44.99 | 21.43 | 71.00 | 10.13 | -10.40 | 24.42 | 3.05 | 0.87 | 5.97 |
| O3 | 45045 | 17,007 | 2,377 | 01/07/80 | 12/31/88 | 40.47 | 15.00 | 75.00 | 14.52 | -1.30 | 27.67 | 3.43 | 0.92 | 5.92 |
| O3 | 47047 | 154 | 365 | 04/12/87 | 10/31/88 | 48.69 | 11.10 | 98.90 | 23.19 | 11.98 | 30.19 | 3.46 | 1.27 | 5.62 |
| O3 | 48245 | 23,972 | 3,204 | 01/07/80 | 12/31/88 | 41.74 | 11.77 | 91.33 | 20.24 | 5.79 | 29.15 | 2.31 | 0.87 | 4.26 |
| O3 | 48453 | 23,621 | 2,818 | 02/29/80 | 12/22/88 | 45.15 | 13.00 | 93.00 | 19.78 | 3.84 | 30.93 | 3.43 | 1.05 | 5.88 |
| O3 | 48457 | 208 | 486 | 03/06/87 | 11/30/88 | 44.35 | 14.12 | 75.00 | 22.38 | 9.55 | 29.94 | 3.38 | 1.31 | 5.57 |
| O3 | 06085 | 76,444 | 3,219 | 01/07/80 | 12/31/88 | 34.38 | 6.86 | 77.71 | 14.19 | 5.28 | 24.71 | 3.82 | 1.28 | 6.24 |
| O3 | 06107 | 11,837 | 2,195 | 01/07/80 | 12/31/88 | 37.32 | 8.00 | 78.00 | 12.17 | -1.70 | 25.19 | 3.99 | 1.42 | 5.84 |
| O3 | 13089 | 26,302 | 2,989 | 01/22/80 | 12/31/88 | 43.99 | 18.00 | 80.00 | 16.14 | -0.48 | 28.75 | 3.39 | 0.96 | 6.00 |
| O3 | 17043 | 22,058 | 1,988 | 01/07/80 | 12/31/88 | 51.84 | 10.00 | 101.00 | 9.65 | -14.05 | 27.11 | 2.94 | 0.85 | 5.93 |
| O3 | 17049 | 1,247 | 1,332 | 05/06/81 | 11/07/88 | 55.52 | 18.00 | 125.00 | 20.98 | 8.49 | 29.15 | 3.28 | 1.10 | 5.48 |
| O3 | 18141 | 21,430 | 3,139 | 01/07/80 | 12/31/88 | 42.90 | 10.72 | 85.00 | 9.77 | -13.13 | 26.47 | 3.03 | 0.81 | 5.99 |
| O3 | 23011 | 10,185 | 2,698 | 01/07/80 | 12/31/88 | 39.33 | 10.00 | 83.00 | 7.13 | -14.52 | 23.33 | 2.96 | 0.85 | 5.56 |
| O3 | 24001 | 5,617 | 1,967 | 05/08/80 | 10/31/88 | 41.96 | 11.00 | 77.00 | 15.13 | -3.84 | 25.86 | 3.45 | 0.97 | 6.03 |
| O3 | 25017 | 36,362 | 1,225 | 01/07/80 | 12/05/88 | 41.58 | 11.30 | 83.00 | 12.94 | -9.94 | 26.73 | 3.20 | 0.99 | 5.50 |
| O3 | 27013 | 3,569 | 2,776 | 01/07/80 | 10/31/88 | 28.29 | 3.00 | 73.00 | 7.68 | -19.27 | 26.14 | 3.10 | 0.77 | 6.25 |
| O3 | 27137 | 5,223 | 787 | 09/22/81 | 03/20/85 | 32.51 | 0.00 | 81.35 | 2.24 | -24.93 | 22.72 | 2.62 | 0.75 | 5.20 |
| O3 | 34005 | 18,825 | 2,609 | 01/07/80 | 11/08/88 | 38.41 | 13.00 | 72.00 | 12.97 | -5.59 | 27.32 | 2.95 | 0.98 | 5.30 |
| O3 | 34029 | 10,024 | 744 | 04/06/85 | 10/31/88 | 53.91 | 23.00 | 113.42 | 19.09 | 7.81 | 27.58 | 2.80 | 0.93 | 5.00 |
| O3 | 36059 | 100,361 | 3,167 | 01/07/80 | 12/31/88 | 40.16 | 17.00 | 82.92 | 11.52 | -6.72 | 26.12 | 2.23 | 0.74 | 4.16 |
| O3 | 37065 | 485 | 493 | 04/06/83 | 10/31/85 | 43.97 | 15.00 | 86.00 | 22.45 | 13.03 | 28.92 | 3.17 | 1.04 | 5.54 |
| O3 | 39095 | 13,830 | 915 | 01/07/80 | 12/29/82 | 33.62 | 5.42 | 76.50 | 10.38 | -13.47 | 25.19 | 2.92 | 0.86 | 5.69 |
| O3 | 39165 | 2,241 | 2,471 | 01/07/80 | 10/31/88 | 42.00 | 10.00 | 88.00 | 12.81 | -8.58 | 26.98 | 3.19 | 0.97 | 5.95 |
| O3 | 42091 | 59,982 | 3,163 | 01/07/80 | 12/31/88 | 34.92 | 7.00 | 80.47 | 11.45 | -8.14 | 26.95 | 3.08 | 0.97 | 5.59 |
| O3 | 45091 | 5,586 | 2,719 | 01/14/80 | 12/31/88 | 43.71 | 9.00 | 83.00 | 16.61 | -0.36 | 28.83 | 3.29 | 0.98 | 5.62 |
| O3 | 49011 | 532 | 425 | 01/07/80 | 06/14/81 | 45.08 | 10.00 | 90.40 | 8.53 | -5.78 | 25.33 | 2.60 | 0.77 | 4.82 |
| O3 | 49019 | 58 | 493 | 03/08/87 | 11/30/88 | 43.49 | 17.00 | 74.00 | 14.81 | -2.81 | 26.22 | 4.46 | 1.86 | 6.33 |
| O3 | 50027 | 2,920 | 1,976 | 04/24/81 | 12/31/88 | 48.83 | 18.00 | 103.00 | 5.44 | -15.86 | 22.81 | 3.17 | 0.82 | 6.15 |
| O3 | 55059 | 6,168 | 2,413 | 04/06/80 | 10/31/88 | 36.08 | 8.00 | 73.70 | 11.38 | -11.31 | 27.16 | 2.35 | 0.70 | 4.81 |
| O3 | 55109 | 2,726 | 2,752 | 05/27/80 | 12/25/88 | 34.87 | 8.50 | 83.00 | 7.60 | -19.22 | 25.64 | 2.95 | 0.83 | 5.81 |
| O3 | 55131 | 1,675 | 1,376 | 04/03/80 | 10/31/88 | 41.31 | 11.00 | 78.00 | 16.58 | 3.31 | 27.21 | 2.87 | 0.95 | 5.26 |
| O3 | 01101 | 7,549 | 1,152 | 07/23/85 | 12/31/88 | 38.44 | 5.00 | 102.00 | 18.54 | 3.02 | 29.46 | 3.42 | 0.93 | 5.98 |
| O3 | 10001 | 3,348 | 1,739 | 04/07/80 | 11/01/88 | 42.00 | 9.00 | 111.55 | 18.77 | 0.91 | 28.51 | 2.73 | 1.03 | 4.77 |
| O3 | 12009 | 2,277 | 269 | 02/03/88 | 12/31/88 | 59.62 | 19.00 | 98.00 | 22.24 | 11.11 | 27.22 | 2.00 | 0.96 | 3.78 |
| O3 | 17199 | 5,342 | 2,570 | 07/28/80 | 12/31/88 | 38.28 | 9.00 | 78.00 | 14.26 | -5.21 | 29.06 | 3.00 | 0.94 | 5.34 |
| O3 | 18057 | 1,827 | 1,518 | 07/02/84 | 12/31/88 | 45.26 | 16.00 | 102.50 | 11.59 | -9.92 | 27.21 | 3.20 | 0.88 | 5.95 |
| O3 | 24025 | 7,199 | 3,023 | 01/07/80 | 12/31/88 | 38.64 | 4.00 | 96.50 | 12.06 | -6.71 | 27.35 | 2.71 | 0.92 | 4.76 |
| O3 | 34019 | 3,969 | 2,786 | 01/07/80 | 12/31/88 | 45.07 | 8.00 | 105.38 | 11.04 | -8.93 | 26.65 | 3.15 | 0.98 | 5.73 |
| O3 | 40143 | 36,460 | 2,795 | 01/07/80 | 10/31/88 | 41.49 | 8.00 | 92.00 | 17.33 | -2.89 | 31.52 | 3.15 | 0.92 | 5.63 |
| O3 | 41013 | 819 | 2,815 | 03/27/80 | 12/31/88 | 41.28 | 11.00 | 87.00 | 6.84 | -10.36 | 23.35 | 3.89 | 1.28 | 6.82 |
| O3 | 42077 | 30,381 | 3,013 | 01/07/80 | 12/31/88 | 37.27 | 9.50 | 84.00 | 9.87 | -9.93 | 25.79 | 3.26 | 0.96 | 6.03 |
| O3 | 45015 | 1,544 | 2,726 | 01/07/80 | 12/31/88 | 37.98 | 6.00 | 91.88 | 17.86 | 2.29 | 28.91 | 2.81 | 0.94 | 4.95 |
| O3 | 45023 | 1,736 | 2,901 | 01/07/80 | 12/31/88 | 37.68 | 5.00 | 93.50 | 16.17 | -0.29 | 28.63 | 3.26 | 0.99 | 5.59 |
| O3 | 51041 | 942 | 890 | 01/07/80 | 09/06/82 | 39.62 | 4.00 | 72.00 | 14.34 | -4.27 | 28.55 | 3.29 | 1.01 | 5.79 |
| O3 | 01117 | 979 | 928 | 11/14/85 | 12/31/88 | 44.65 | 15.00 | 84.00 | 17.00 | 1.44 | 29.56 | 3.32 | 0.99 | 5.74 |
| O3 | 06097 | 20,797 | 2,597 | 01/07/80 | 12/31/88 | 27.38 | 1.00 | 71.05 | 14.18 | 5.37 | 23.69 | 3.83 | 0.96 | 6.25 |
| O3 | 22033 | 24,501 | 2,951 | 03/10/80 | 12/31/88 | 40.30 | 9.00 | 78.00 | 19.64 | 4.34 | 28.69 | 3.12 | 1.06 | 5.38 |
| O3 | 23019 | 1,856 | 583 | 05/04/80 | 10/31/83 | 37.28 | 15.32 | 72.45 | 15.65 | 4.63 | 23.27 | 3.12 | 0.78 | 5.44 |
| O3 | 25013 | 35,910 | 2,752 | 06/16/80 | 12/31/88 | 38.77 | 14.00 | 72.37 | 8.96 | -10.72 | 24.65 | 3.14 | 0.86 | 5.64 |
| O3 | 26161 | 4,289 | 557 | 01/07/80 | 12/31/88 | 34.27 | 10.00 | 70.00 | 10.25 | -11.80 | 27.77 | 3.02 | 0.94 | 5.94 |
| O3 | 28075 | 1,621 | 533 | 01/07/87 | 12/31/88 | 27.75 | 5.00 | 46.00 | 17.20 | 1.33 | 29.07 | 3.29 | 0.89 | 5.58 |
| O3 | 34023 | 29,668 | 2,542 | 01/07/80 | 11/13/88 | 29.07 | 4.00 | 66.00 | 12.60 | -7.26 | 27.11 | 2.98 | 0.90 | 5.44 |
| O3 | 36027 | 13,435 | 2,249 | 02/06/80 | 12/31/88 | 40.29 | 20.00 | 61.00 | 9.34 | -11.08 | 25.16 | 3.13 | 0.96 | 5.69 |
| O3 | 37067 | 14,608 | 1,578 | 07/03/80 | 12/31/88 | 33.51 | 5.42 | 76.29 | 15.41 | -1.60 | 28.41 | 3.43 | 0.94 | 6.06 |
| O3 | 37101 | 578 | 484 | 04/06/83 | 10/31/85 | 40.96 | 6.00 | 97.85 | 21.65 | 11.11 | 29.38 | 3.16 | 1.02 | 5.48 |
| O3 | 40087 | 865 | 2,294 | 03/24/81 | 12/31/88 | 40.68 | 3.00 | 106.00 | 17.76 | -1.94 | 31.02 | 3.23 | 0.98 | 5.71 |
| O3 | 48039 | 3,478 | 1,781 | 01/22/80 | 10/31/86 | 37.39 | 4.00 | 79.00 | 21.46 | 6.88 | 29.45 | 2.50 | 0.96 | 4.51 |
| O3 | 51153 | 2,507 | 2,641 | 07/16/80 | 12/27/88 | 40.76 | 4.00 | 100.00 | 13.50 | -4.62 | 28.33 | 3.13 | 1.02 | 5.59 |
| O3 | 55009 | 5,227 | 1,274 | 04/24/80 | 10/06/86 | 31.72 | 10.00 | 57.59 | 16.61 | 3.44 | 25.79 | 2.90 | 0.91 | 5.16 |
| O3 | 55017 | 840 | 776 | 05/06/80 | 09/30/85 | 35.36 | 17.00 | 53.00 | 16.95 | 5.01 | 25.53 | 3.12 | 0.99 | 5.58 |
| O3 | 06007 | 8,356 | 1,891 | 04/24/80 | 11/30/88 | 25.92 | 6.00 | 55.00 | 18.10 | 4.44 | 29.44 | 4.60 | 1.08 | 7.28 |
| O3 | 06021 | 355 | 978 | 01/07/80 | 10/30/83 | 46.80 | 20.00 | 83.00 | 16.61 | 3.63 | 30.51 | 4.21 | 1.13 | 6.60 |
| O3 | 17111 | 7,017 | 2,810 | 01/07/80 | 12/31/88 | 33.09 | 7.00 | 82.00 | 9.22 | -15.46 | 26.90 | 2.89 | 0.85 | 5.80 |
| O3 | 19113 | 7,424 | 2,066 | 02/06/80 | 10/31/88 | 39.02 | 0.00 | 85.00 | 11.82 | -12.84 | 27.38 | 3.18 | 0.96 | 5.83 |
| O3 | 37159 | 8,575 | 2,840 | 01/07/80 | 12/31/88 | 34.28 | 6.00 | 93.00 | 15.67 | -1.67 | 28.63 | 3.31 | 0.97 | 5.71 |
| O3 | 39099 | 25,079 | 3,210 | 01/07/80 | 12/31/88 | 35.07 | 8.00 | 77.00 | 9.63 | -10.96 | 24.96 | 3.10 | 0.87 | 6.09 |
| O3 | 41005 | 15,331 | 3,164 | 01/09/80 | 12/31/88 | 35.48 | 9.00 | 72.00 | 8.13 | -4.06 | 21.68 | 3.19 | 0.82 | 6.67 |
| O3 | 42043 | 24,656 | 2,993 | 06/06/80 | 12/31/88 | 40.21 | 15.00 | 75.00 | 10.85 | -8.69 | 26.43 | 3.06 | 0.92 | 5.63 |
| O3 | 42095 | 7,093 | 1,998 | 01/07/80 | 12/09/85 | 34.87 | 4.67 | 84.04 | 9.98 | -10.50 | 25.29 | 3.24 | 0.95 | 5.91 |
| O3 | 47055 | 701 | 1,393 | 05/04/80 | 12/31/88 | 52.48 | 18.80 | 89.00 | 19.68 | 2.67 | 29.26 | 3.37 | 1.11 | 5.48 |
| O3 | 54011 | 8,272 | 1,722 | 01/07/82 | 10/31/88 | 39.75 | 9.00 | 79.00 | 15.07 | -4.53 | 27.67 | 3.30 | 0.96 | 6.09 |
| O3 | 05119 | 29,148 | 2,307 | 01/14/80 | 12/31/88 | 42.92 | 15.00 | 74.00 | 19.83 | 0.49 | 31.15 | 3.29 | 1.08 | 5.53 |
| O3 | 06029 | 29,400 | 3,172 | 01/07/80 | 12/31/88 | 45.11 | 8.00 | 82.00 | 16.25 | 3.09 | 29.67 | 4.13 | 1.67 | 5.94 |
| O3 | 21067 | 18,467 | 2,102 | 01/07/80 | 10/31/88 | 45.97 | 6.00 | 90.00 | 16.87 | -3.08 | 27.77 | 3.39 | 1.12 | 5.88 |
| O3 | 23005 | 21,691 | 3,108 | 01/07/80 | 12/31/88 | 37.26 | 9.00 | 81.00 | 7.62 | -12.69 | 23.44 | 2.62 | 0.70 | 4.99 |
| O3 | 23013 | 814 | 716 | 12/28/86 | 12/31/88 | 51.35 | 25.50 | 80.56 | 7.19 | -12.24 | 22.48 | 2.29 | 0.69 | 4.45 |
| O3 | 23027 | 441 | 793 | 04/24/84 | 10/15/88 | 43.37 | 18.00 | 75.00 | 15.25 | 5.68 | 23.95 | 2.72 | 0.72 | 4.81 |
| O3 | 35043 | 490 | 1,352 | 01/25/81 | 12/30/88 | 44.90 | 10.78 | 81.00 | 9.49 | -6.82 | 22.69 | 4.07 | 1.70 | 6.11 |
| O3 | 39003 | 4,534 | 1,384 | 05/04/80 | 10/31/88 | 38.01 | 5.00 | 72.00 | 14.92 | -8.19 | 27.20 | 3.35 | 1.07 | 5.99 |
| O3 | 39143 | 226 | 185 | 04/13/88 | 11/13/88 | 54.47 | 19.20 | 88.00 | 18.91 | 4.40 | 29.98 | 3.41 | 1.20 | 6.22 |
| O3 | 41047 | 16,324 | 3,154 | 01/14/80 | 12/31/88 | 36.97 | 14.00 | 65.33 | 9.35 | -2.21 | 22.29 | 3.33 | 0.89 | 6.68 |
| O3 | 42019 | 9,123 | 2,991 | 01/07/80 | 12/20/88 | 34.15 | 5.00 | 83.00 | 9.31 | -11.30 | 24.32 | 3.06 | 0.88 | 6.02 |
| O3 | 51179 | 352 | 1,468 | 04/07/80 | 10/31/88 | 57.25 | 26.00 | 95.33 | 21.32 | 9.62 | 29.28 | 3.26 | 1.04 | 5.52 |
| O3 | 55025 | 23,591 | 3,113 | 01/07/80 | 12/31/88 | 32.08 | 6.00 | 68.00 | 8.60 | -16.33 | 26.45 | 2.99 | 0.86 | 5.71 |
| O3 | 55117 | 2,856 | 1,183 | 05/06/80 | 10/31/88 | 54.85 | 22.00 | 90.45 | 16.76 | 3.57 | 26.79 | 2.48 | 0.82 | 4.60 |
| O3 | 01043 | 2,578 | 1,792 | 07/08/82 | 10/31/88 | 35.63 | 7.00 | 71.00 | 17.79 | -0.27 | 28.90 | 3.24 | 1.09 | 5.48 |
| O3 | 04019 | 47,664 | 3,282 | 01/07/80 | 12/31/88 | 40.36 | 17.00 | 65.75 | 20.14 | 6.22 | 32.54 | 4.36 | 1.79 | 6.39 |
| O3 | 06013 | 42,381 | 3,121 | 04/14/80 | 12/31/88 | 30.94 | 4.00 | 59.67 | 14.61 | 5.47 | 24.20 | 4.03 | 1.33 | 6.91 |
| O3 | 06017 | 5,327 | 3,205 | 01/07/80 | 12/31/88 | 38.99 | 17.50 | 63.33 | 10.63 | -1.77 | 24.15 | 3.98 | 0.89 | 6.58 |
| O3 | 12001 | 18,052 | 3,071 | 01/07/80 | 12/31/88 | 38.24 | 7.00 | 78.12 | 20.23 | 6.66 | 28.22 | 3.19 | 1.18 | 5.48 |
| O3 | 19193 | 11,262 | 3,168 | 01/07/80 | 12/31/88 | 31.30 | 8.59 | 60.00 | 9.47 | -14.89 | 28.00 | 3.31 | 0.86 | 6.31 |
| O3 | 22019 | 9,828 | 2,491 | 01/07/80 | 12/31/88 | 39.58 | 10.00 | 80.00 | 20.65 | 5.08 | 28.79 | 2.82 | 0.99 | 4.96 |
| O3 | 24021 | 5,249 | 2,402 | 07/22/80 | 11/18/88 | 44.48 | 7.00 | 102.97 | 11.97 | -6.98 | 27.08 | 3.19 | 0.98 | 5.76 |
| O3 | 34009 | 7,406 | 2,824 | 01/07/80 | 12/31/88 | 45.99 | 12.00 | 106.00 | 13.04 | -4.27 | 26.78 | 1.95 | 0.73 | 3.65 |
| O3 | 34021 | 5,972 | 702 | 03/02/80 | 09/30/82 | 48.06 | 15.53 | 89.43 | 12.85 | -8.49 | 26.49 | 3.21 | 0.98 | 5.65 |
| O3 | 35013 | 3,006 | 2,224 | 01/07/80 | 07/31/86 | 38.54 | 13.00 | 66.00 | 16.59 | 1.59 | 29.77 | 4.28 | 1.87 | 6.29 |
| O3 | 42049 | 23,071 | 2,932 | 01/07/80 | 12/31/88 | 38.51 | 4.00 | 103.00 | 8.13 | -11.91 | 24.03 | 2.38 | 0.61 | 5.19 |
| O3 | 42069 | 23,841 | 2,925 | 01/07/80 | 12/31/88 | 36.84 | 5.00 | 88.90 | 7.99 | -12.52 | 23.59 | 3.04 | 0.91 | 5.71 |
| O3 | 45051 | 6,319 | 2,479 | 01/19/80 | 12/31/88 | 37.42 | 10.00 | 74.00 | 17.59 | 1.75 | 28.76 | 2.90 | 1.05 | 5.19 |
| O3 | 47149 | 219 | 105 | 04/28/88 | 09/26/88 | 44.24 | 17.20 | 94.00 | 23.81 | 14.21 | 30.15 | 3.95 | 1.52 | 5.97 |
| O3 | 06025 | 4,327 | 2,394 | 01/07/80 | 10/31/87 | 45.37 | 18.00 | 76.00 | 24.47 | 11.26 | 36.64 | 4.40 | 2.03 | 6.13 |
| O3 | 13215 | 15,131 | 3,171 | 01/07/80 | 12/31/88 | 40.15 | 12.50 | 76.62 | 17.76 | 1.74 | 29.34 | 3.52 | 1.03 | 5.99 |
| O3 | 21037 | 4,627 | 1,996 | 01/07/80 | 10/31/88 | 44.86 | 8.00 | 92.12 | 16.50 | -4.68 | 28.21 | 3.33 | 1.02 | 5.83 |
| O3 | 47189 | 242 | 233 | 03/20/88 | 11/30/88 | 47.43 | 18.80 | 78.20 | 19.76 | 7.18 | 30.05 | 3.66 | 1.42 | 5.93 |
| O3 | 48085 | 2,466 | 1,042 | 01/07/86 | 12/31/88 | 39.30 | 23.00 | 63.49 | 18.72 | 2.00 | 31.97 | 3.23 | 0.96 | 6.00 |
| O3 | 50003 | 266 | 232 | 02/06/86 | 12/31/86 | 45.97 | 31.77 | 66.00 | 3.83 | -12.28 | 18.89 | 3.16 | 0.98 | 6.05 |
| O3 | 51059 | 5,237 | 1,009 | 01/07/80 | 11/16/82 | 39.18 | 17.00 | 67.27 | 12.90 | -6.13 | 27.82 | 3.10 | 0.98 | 5.55 |
| O3 | 53011 | 1,562 | 455 | 04/24/80 | 12/29/88 | 49.82 | 17.35 | 92.00 | 14.03 | 1.86 | 24.30 | 3.46 | 0.75 | 7.03 |
| O3 | 54039 | 9,057 | 1,272 | 05/14/80 | 10/20/88 | 34.35 | 10.00 | 75.00 | 20.27 | 9.22 | 27.12 | 3.27 | 1.11 | 5.65 |
| O3 | 55101 | 4,921 | 1,453 | 06/30/80 | 10/15/88 | 40.55 | 12.00 | 84.00 | 18.26 | 5.88 | 27.74 | 2.25 | 0.73 | 4.10 |
| O3 | 06031 | 1,456 | 1,089 | 07/15/80 | 09/30/83 | 48.40 | 0.00 | 102.00 | 18.92 | 6.98 | 31.86 | 4.30 | 1.71 | 6.14 |
| O3 | 06093 | 2,216 | 2,291 | 01/19/81 | 12/31/88 | 56.02 | 10.00 | 112.75 | 9.17 | -4.94 | 23.62 | 4.01 | 0.97 | 6.88 |
| O3 | 17099 | 7,943 | 2,651 | 01/07/80 | 12/31/88 | 32.74 | 6.00 | 79.00 | 10.63 | -13.49 | 27.59 | 3.19 | 0.95 | 6.03 |
| O3 | 23029 | 108 | 126 | 04/06/84 | 10/31/84 | 35.40 | 18.00 | 56.50 | 12.81 | 2.14 | 21.77 | 2.52 | 0.67 | 4.46 |
| O3 | 26145 | 3,108 | 586 | 01/07/80 | 10/18/81 | 38.11 | 17.62 | 69.38 | 8.79 | -14.55 | 24.50 | 3.07 | 0.96 | 6.22 |
| O3 | 42011 | 10,816 | 1,356 | 06/06/80 | 10/31/88 | 45.05 | 8.88 | 93.12 | 18.36 | 5.68 | 27.24 | 3.41 | 1.05 | 5.75 |
| O3 | 06081 | 37,494 | 3,065 | 01/07/80 | 12/31/88 | 35.58 | 12.00 | 67.00 | 13.25 | 7.21 | 19.56 | 2.51 | 0.81 | 4.77 |
| O3 | 08005 | 11,466 | 3,211 | 01/07/80 | 12/31/88 | 40.59 | 14.00 | 72.50 | 9.11 | -10.64 | 24.97 | 4.47 | 1.41 | 7.06 |
| O3 | 12071 | 24,225 | 3,005 | 02/04/80 | 12/31/88 | 37.40 | 13.00 | 73.00 | 22.84 | 12.36 | 28.19 | 2.67 | 1.12 | 4.71 |
| O3 | 17119 | 17,933 | 3,114 | 01/07/80 | 12/31/88 | 36.63 | 9.00 | 76.00 | 13.17 | -8.27 | 29.44 | 3.10 | 0.89 | 5.69 |
| O3 | 18019 | 5,048 | 2,646 | 01/07/80 | 12/31/88 | 36.50 | 10.00 | 75.00 | 13.27 | -7.89 | 28.06 | 3.14 | 0.96 | 5.74 |
| O3 | 39025 | 4,320 | 2,982 | 01/07/80 | 12/31/88 | 38.51 | 20.00 | 64.00 | 12.44 | -8.31 | 27.44 | 3.10 | 0.95 | 5.72 |
| O3 | 51087 | 9,199 | 1,826 | 05/06/80 | 10/31/88 | 50.84 | 14.00 | 102.00 | 18.68 | -0.13 | 29.15 | 3.37 | 1.02 | 5.73 |
| O3 | 04025 | 1,625 | 740 | 05/08/80 | 10/15/84 | 46.42 | 16.48 | 90.05 | 22.05 | 10.34 | 29.19 | 4.57 | 2.31 | 6.55 |
| O3 | 12057 | 61,304 | 3,208 | 01/07/80 | 12/31/88 | 41.76 | 18.06 | 72.00 | 21.86 | 9.92 | 28.26 | 2.88 | 1.19 | 5.09 |
| O3 | 18081 | 809 | 484 | 07/06/87 | 12/31/88 | 44.69 | 26.00 | 73.00 | 13.27 | -7.13 | 29.29 | 3.45 | 1.00 | 6.39 |
| O3 | 19163 | 7,381 | 2,198 | 09/09/80 | 12/31/88 | 35.19 | 2.00 | 76.07 | 9.20 | -15.14 | 27.81 | 3.14 | 0.86 | 5.97 |
| O3 | 29021 | 929 | 335 | 06/11/87 | 10/31/88 | 52.86 | 17.35 | 97.00 | 21.17 | 7.81 | 31.49 | 3.54 | 1.44 | 5.91 |
| O3 | 34011 | 5,387 | 1,560 | 03/30/80 | 09/30/88 | 45.75 | 9.00 | 105.03 | 17.98 | -1.68 | 27.91 | 2.61 | 1.00 | 4.52 |
| O3 | 42133 | 19,472 | 2,689 | 01/07/80 | 06/22/88 | 39.57 | 2.00 | 103.00 | 10.74 | -8.05 | 26.30 | 3.04 | 0.92 | 5.48 |
| O3 | 55011 | 41 | 227 | 02/17/81 | 08/06/82 | 39.51 | 10.00 | 74.75 | 9.44 | -15.12 | 23.62 | 2.87 | 0.79 | 5.24 |
| O3 | 55073 | 5,375 | 2,481 | 05/21/80 | 12/31/88 | 40.47 | 18.00 | 74.50 | 8.58 | -17.46 | 24.77 | 2.98 | 0.91 | 5.57 |
| O3 | 01103 | 1,179 | 599 | 01/07/80 | 04/03/83 | 37.26 | 8.00 | 71.05 | 15.41 | -0.62 | 27.45 | 3.06 | 0.86 | 5.31 |
| O3 | 06113 | 6,765 | 2,952 | 01/14/80 | 12/31/88 | 57.36 | 22.00 | 116.00 | 16.55 | 5.14 | 29.10 | 4.39 | 1.20 | 7.14 |
| O3 | 18085 | 2,035 | 2,196 | 01/07/80 | 10/31/88 | 43.18 | 8.00 | 90.00 | 11.84 | -12.32 | 26.32 | 3.19 | 0.92 | 5.97 |
| O3 | 22101 | 375 | 369 | 05/06/83 | 12/31/88 | 48.05 | 23.20 | 79.80 | 23.53 | 10.40 | 28.68 | 2.30 | 0.94 | 4.02 |
| O3 | 36007 | 1,555 | 253 | 01/07/80 | 09/30/80 | 42.85 | 19.30 | 66.70 | 9.11 | -12.15 | 23.38 | 3.23 | 1.16 | 5.95 |
| O3 | 37129 | 5,229 | 1,591 | 04/03/80 | 10/31/88 | 54.57 | 15.00 | 93.00 | 22.61 | 13.00 | 28.55 | 2.29 | 0.85 | 4.13 |
| O3 | 45037 | 77 | 263 | 09/22/80 | 10/30/82 | 35.26 | 5.00 | 75.00 | 16.80 | 0.21 | 29.32 | 3.35 | 0.97 | 5.87 |
| O3 | 08059 | 4,558 | 991 | 01/07/80 | 10/15/88 | 44.74 | 8.00 | 86.00 | 7.16 | -11.31 | 20.43 | 4.90 | 1.49 | 7.56 |
| O3 | 21029 | 678 | 3,077 | 01/07/80 | 12/31/88 | 35.95 | 10.00 | 73.00 | 13.16 | -6.77 | 28.25 | 3.20 | 0.96 | 5.91 |
| O3 | 23017 | 1,830 | 1,520 | 09/07/82 | 10/31/88 | 35.44 | 6.00 | 83.00 | 9.22 | -13.63 | 22.51 | 3.04 | 0.78 | 5.69 |
| O3 | 24013 | 133 | 81 | 06/08/83 | 10/19/83 | 30.70 | 7.00 | 62.00 | 21.74 | 12.28 | 27.07 | 3.43 | 1.44 | 5.25 |
| O3 | 28149 | 543 | 397 | 04/12/87 | 10/31/88 | 55.21 | 18.90 | 98.00 | 23.92 | 14.76 | 29.80 | 3.26 | 1.26 | 5.40 |
| O3 | 34015 | 432 | 123 | 04/23/81 | 09/30/81 | 47.29 | 27.00 | 79.85 | 20.66 | 11.92 | 27.31 | 3.08 | 1.01 | 5.14 |
| O3 | 37155 | 3,807 | 1,634 | 10/06/82 | 10/31/88 | 34.09 | 0.00 | 85.35 | 17.82 | 1.32 | 29.08 | 3.30 | 1.21 | 5.84 |
| O3 | 39027 | 2,608 | 2,950 | 01/07/80 | 12/31/88 | 38.20 | 20.00 | 62.00 | 11.28 | -9.15 | 26.61 | 3.19 | 0.96 | 6.00 |
| O3 | 39107 | 1,623 | 2,338 | 12/28/81 | 12/31/88 | 43.57 | 5.00 | 107.00 | 10.69 | -11.54 | 26.62 | 3.17 | 0.93 | 6.00 |
| O3 | 39133 | 99 | 69 | 07/15/80 | 09/30/80 | 52.14 | 21.40 | 84.10 | 21.17 | 13.43 | 26.15 | 2.85 | 1.01 | 4.63 |
| O3 | 51550 | 4,261 | 2,483 | 09/16/80 | 10/31/88 | 46.99 | 11.00 | 94.00 | 16.16 | -1.18 | 28.14 | 2.77 | 0.92 | 5.24 |
| O3 | 17019 | 924 | 247 | 01/22/80 | 11/14/80 | 21.25 | 7.00 | 35.00 | 13.91 | -9.71 | 28.00 | 3.42 | 1.13 | 6.02 |
| O3 | 17161 | 11,538 | 2,750 | 01/07/80 | 12/31/88 | 39.45 | 9.00 | 77.00 | 10.37 | -14.23 | 28.13 | 2.99 | 0.84 | 5.74 |
| O3 | 26147 | 8,910 | 2,886 | 02/07/80 | 12/31/88 | 38.86 | 11.00 | 77.50 | 9.74 | -12.13 | 25.94 | 2.50 | 0.77 | 5.10 |
| O3 | 29077 | 16,945 | 2,341 | 01/07/80 | 12/31/88 | 42.72 | 12.00 | 76.00 | 16.91 | -1.43 | 29.00 | 3.30 | 0.94 | 5.56 |
| O3 | 34027 | 4,639 | 502 | 01/30/80 | 09/30/83 | 32.22 | 3.00 | 94.47 | 9.78 | -11.52 | 26.42 | 3.20 | 1.07 | 5.38 |
| O3 | 36045 | 2,742 | 1,009 | 01/07/86 | 12/31/88 | 46.67 | 29.00 | 76.60 | 8.27 | -13.27 | 24.45 | 2.36 | 0.75 | 4.99 |
| O3 | 51800 | 536 | 464 | 03/06/87 | 11/19/88 | 46.01 | 16.57 | 76.42 | 19.09 | 4.03 | 28.90 | 3.27 | 1.06 | 5.60 |
| O3 | 55071 | 576 | 305 | 04/24/84 | 09/30/85 | 42.17 | 21.60 | 83.00 | 17.48 | 7.35 | 24.98 | 2.49 | 0.84 | 4.25 |
| O3 | 22095 | 1,366 | 2,684 | 11/05/80 | 12/31/88 | 40.79 | 11.00 | 84.92 | 20.01 | 5.44 | 28.51 | 2.47 | 0.86 | 4.40 |
| O3 | 29183 | 6,897 | 3,125 | 01/07/80 | 12/31/88 | 34.32 | 5.00 | 75.00 | 13.17 | -8.23 | 29.27 | 3.16 | 0.87 | 5.79 |
| O3 | 33005 | 77 | 78 | 05/13/81 | 09/11/81 | 33.01 | 12.00 | 62.08 | 17.92 | 9.49 | 23.41 | 3.54 | 1.22 | 5.88 |
| O3 | 37157 | 2,885 | 1,622 | 10/07/82 | 12/31/88 | 41.08 | 10.00 | 75.00 | 13.27 | -3.21 | 27.95 | 3.34 | 0.98 | 5.95 |
| O3 | 42131 | 1,464 | 2,988 | 01/14/80 | 12/25/88 | 35.08 | 4.00 | 85.32 | 8.29 | -12.39 | 23.86 | 3.04 | 0.89 | 5.76 |
| O3 | 47155 | 97 | 117 | 05/22/88 | 09/15/88 | 48.86 | 23.00 | 93.00 | 21.49 | 14.28 | 26.35 | 3.70 | 1.00 | 6.23 |
| O3 | 47163 | 14,941 | 3,105 | 01/07/80 | 12/31/88 | 39.93 | 9.33 | 77.50 | 12.31 | -5.41 | 24.75 | 3.43 | 1.00 | 6.30 |
| O3 | 47165 | 288 | 157 | 05/05/88 | 10/31/88 | 52.92 | 23.00 | 115.10 | 22.06 | 9.77 | 30.53 | 3.49 | 1.23 | 5.71 |
| O3 | 54069 | 6,650 | 2,204 | 01/07/80 | 10/30/88 | 27.58 | 0.00 | 65.00 | 12.34 | -8.37 | 25.39 | 3.14 | 0.92 | 5.80 |
| O3 | 17197 | 14,320 | 2,880 | 01/07/80 | 12/31/88 | 33.26 | 5.00 | 79.03 | 10.56 | -12.41 | 27.46 | 3.11 | 0.90 | 6.09 |
| O3 | 21071 | 2,814 | 2,524 | 01/07/81 | 12/31/88 | 32.52 | 6.08 | 74.00 | 13.34 | -4.93 | 26.73 | 3.27 | 0.89 | 6.24 |
| O3 | 24031 | 32,640 | 2,771 | 04/06/80 | 12/31/88 | 40.17 | 4.50 | 100.00 | 12.59 | -6.00 | 27.92 | 3.15 | 0.95 | 5.64 |
| O3 | 51173 | 2,786 | 2,764 | 01/07/80 | 12/31/88 | 36.94 | 3.00 | 100.00 | 10.08 | -8.32 | 23.33 | 3.34 | 0.93 | 6.26 |
| O3 | 54107 | 5,893 | 1,936 | 02/06/83 | 12/31/88 | 35.48 | 2.00 | 100.00 | 12.24 | -7.19 | 26.80 | 3.18 | 0.88 | 6.03 |
| O3 | 02020 | 4,505 | 1,997 | 02/12/82 | 12/31/88 | 36.68 | 8.00 | 81.00 | -1.81 | -18.12 | 11.40 | 1.45 | 0.37 | 3.35 |
| O3 | 04027 | 5,794 | 2,524 | 01/21/80 | 12/20/88 | 42.38 | 13.07 | 72.00 | 22.68 | 9.22 | 36.20 | 4.40 | 2.09 | 6.19 |
| O3 | 12073 | 10,428 | 2,773 | 01/07/80 | 12/31/88 | 38.89 | 15.00 | 75.00 | 19.61 | 5.07 | 28.52 | 3.23 | 1.06 | 5.48 |
| O3 | 21059 | 2,329 | 1,029 | 10/06/80 | 10/31/88 | 40.10 | 8.00 | 79.00 | 20.01 | 2.49 | 28.79 | 3.23 | 0.95 | 5.71 |
| O3 | 29099 | 2,277 | 1,401 | 04/06/81 | 10/31/88 | 38.86 | 9.00 | 88.00 | 20.80 | 8.67 | 29.71 | 3.33 | 1.13 | 5.54 |
| O3 | 37025 | 3,375 | 1,526 | 04/01/80 | 10/31/88 | 57.09 | 20.00 | 100.00 | 21.48 | 10.46 | 29.49 | 3.41 | 1.15 | 5.67 |
| O3 | 38065 | 29 | 2,725 | 01/07/80 | 11/02/88 | 46.32 | 10.00 | 94.45 | 7.05 | -21.78 | 26.15 | 3.50 | 0.90 | 6.65 |
| O3 | 45021 | 51 | 68 | 10/06/88 | 12/21/88 | 31.19 | 12.68 | 55.30 | 9.85 | -0.20 | 17.39 | 3.30 | 1.03 | 5.10 |
| O3 | 01089 | 6,662 | 1,421 | 01/07/80 | 11/30/88 | 45.22 | 12.00 | 86.00 | 15.91 | -1.32 | 28.91 | 3.24 | 0.97 | 5.55 |
| O3 | 10005 | 8,503 | 2,772 | 01/10/80 | 12/31/88 | 40.97 | 8.00 | 83.86 | 13.86 | -3.51 | 27.94 | 2.48 | 0.87 | 4.54 |
| O3 | 17133 | 781 | 2,910 | 01/07/80 | 12/31/88 | 41.83 | 10.00 | 90.00 | 14.04 | -7.11 | 29.32 | 3.14 | 0.87 | 5.70 |
| O3 | 34035 | 3,178 | 854 | 01/08/80 | 01/25/83 | 34.37 | 2.00 | 90.67 | 10.78 | -9.37 | 25.67 | 3.09 | 0.95 | 5.54 |
| O3 | 45013 | 150 | 118 | 01/07/80 | 12/31/88 | 18.64 | 0.00 | 40.08 | 11.59 | 0.51 | 18.73 | 2.64 | 0.80 | 4.78 |
| O3 | 06027 | 94 | 171 | 05/14/80 | 10/31/80 | 57.88 | 33.75 | 92.25 | 23.30 | 10.07 | 31.94 | 4.00 | 2.44 | 5.14 |
| O3 | 23007 | 579 | 955 | 01/22/86 | 12/31/88 | 42.46 | 28.00 | 66.00 | 4.36 | -17.04 | 22.53 | 2.94 | 0.81 | 5.70 |
| O3 | 55089 | 642 | 806 | 04/13/80 | 10/15/84 | 44.99 | 13.00 | 99.88 | 16.46 | 4.59 | 26.16 | 1.91 | 0.62 | 3.47 |
| O3 | 21051 | 861 | 1,910 | 10/08/81 | 11/01/88 | 43.67 | 11.72 | 83.27 | 14.11 | -5.08 | 26.85 | 3.28 | 1.00 | 6.06 |
| O3 | 29037 | 520 | 777 | 05/16/83 | 12/31/86 | 30.97 | 6.00 | 74.00 | 17.06 | -5.66 | 30.11 | 3.14 | 0.78 | 5.70 |
| O3 | 08123 | 5,648 | 2,871 | 07/17/80 | 12/31/88 | 31.86 | 14.62 | 50.50 | 9.41 | -10.59 | 25.07 | 4.36 | 1.33 | 7.05 |
| O3 | 27071 | 153 | 450 | 03/09/87 | 10/31/88 | 56.29 | 27.23 | 88.78 | 11.43 | -7.72 | 24.72 | 3.17 | 0.96 | 6.33 |
| O3 | 28033 | 320 | 948 | 01/07/80 | 10/06/88 | 35.84 | 16.00 | 60.00 | 19.20 | -0.82 | 30.38 | 3.10 | 1.05 | 5.31 |
| O3 | 36091 | 1,993 | 976 | 07/29/85 | 12/31/88 | 29.72 | 6.00 | 86.00 | 7.80 | -11.81 | 24.47 | 3.01 | 0.84 | 5.91 |
| O3 | 48167 | 13,249 | 2,554 | 01/07/80 | 12/31/88 | 43.92 | 10.00 | 96.00 | 20.67 | 6.81 | 29.22 | 2.20 | 0.86 | 4.33 |
| O3 | 13097 | 472 | 470 | 04/12/87 | 12/31/88 | 57.70 | 15.00 | 115.10 | 18.03 | -0.02 | 28.67 | 3.45 | 1.12 | 5.72 |
| O3 | 17143 | 18,278 | 2,819 | 01/07/80 | 12/31/88 | 38.48 | 8.00 | 86.00 | 10.99 | -13.70 | 28.21 | 3.15 | 0.96 | 5.89 |
| O3 | 26103 | 4,018 | 2,514 | 05/04/80 | 10/31/88 | 35.75 | 6.00 | 91.00 | 7.54 | -17.57 | 23.24 | 2.66 | 0.76 | 5.36 |
| O3 | 28073 | 193 | 225 | 03/17/87 | 11/30/87 | 46.90 | 20.00 | 76.00 | 21.74 | 8.36 | 28.89 | 3.51 | 1.11 | 6.10 |
| O3 | 28087 | 614 | 473 | 04/12/87 | 10/28/88 | 39.59 | 4.80 | 97.60 | 19.81 | 1.84 | 30.22 | 3.63 | 1.31 | 6.02 |
| O3 | 48409 | 1,857 | 2,232 | 07/23/81 | 12/31/88 | 39.25 | 12.00 | 77.00 | 22.06 | 7.90 | 29.71 | 2.74 | 1.04 | 4.92 |
| O3 | 28059 | 889 | 517 | 05/06/84 | 05/06/87 | 49.44 | 22.00 | 81.00 | 24.61 | 16.64 | 28.66 | 2.52 | 0.97 | 4.55 |
| O3 | 42013 | 11,145 | 2,218 | 07/10/80 | 12/31/88 | 35.46 | 2.00 | 96.00 | 10.30 | -9.58 | 24.50 | 3.11 | 0.87 | 5.90 |
| O3 | 50007 | 7,885 | 2,698 | 10/06/80 | 12/31/88 | 40.51 | 8.00 | 80.00 | 6.25 | -16.29 | 23.21 | 2.78 | 0.78 | 5.75 |
| O3 | 55021 | 3,447 | 3,137 | 01/07/80 | 12/31/88 | 36.77 | 8.00 | 70.30 | 8.20 | -16.99 | 26.20 | 2.98 | 0.86 | 5.80 |
| O3 | 09007 | 873 | 280 | 01/07/80 | 11/13/80 | 44.10 | 9.97 | 117.05 | 12.50 | -8.29 | 26.10 | 2.86 | 1.11 | 4.84 |
| O3 | 26027 | 708 | 1,040 | 06/06/80 | 03/08/84 | 24.39 | 1.00 | 73.03 | 9.92 | -13.04 | 26.10 | 2.79 | 0.72 | 5.46 |
| O3 | 28001 | 527 | 434 | 06/06/87 | 11/30/88 | 53.68 | 13.82 | 105.00 | 22.55 | 9.57 | 29.39 | 3.14 | 1.13 | 5.34 |
| O3 | 40027 | 723 | 432 | 01/07/80 | 12/31/88 | 32.37 | 6.00 | 76.00 | 15.86 | -2.08 | 33.61 | 3.44 | 0.86 | 6.13 |
| O3 | 20091 | 9,503 | 2,475 | 01/10/80 | 12/31/88 | 27.05 | 0.00 | 77.00 | 13.36 | -8.93 | 29.78 | 3.06 | 0.87 | 5.81 |
| O3 | 25011 | 1,979 | 1,426 | 06/12/80 | 10/31/88 | 45.64 | 16.00 | 92.38 | 15.04 | 3.20 | 24.68 | 3.37 | 0.83 | 5.79 |
| O3 | 28089 | 1,324 | 2,258 | 01/07/80 | 11/30/88 | 28.48 | 5.00 | 57.00 | 18.20 | 0.74 | 29.41 | 3.35 | 1.10 | 5.70 |
| O3 | 32031 | 17,178 | 3,058 | 01/07/80 | 12/31/88 | 35.98 | 10.71 | 72.00 | 9.45 | -7.89 | 25.84 | 3.41 | 1.31 | 5.30 |
| O3 | 39103 | 90 | 48 | 01/07/80 | 03/06/80 | 12.69 | 1.18 | 22.00 | -5.90 | -15.70 | 5.20 | 2.81 | 1.05 | 7.16 |
| O3 | 06039 | 1,754 | 1,484 | 10/06/81 | 12/31/88 | 37.96 | 7.00 | 75.92 | 12.92 | -0.84 | 25.93 | 4.25 | 1.34 | 6.44 |
| O3 | 19121 | 684 | 2,363 | 01/07/80 | 10/31/88 | 40.52 | 6.00 | 89.00 | 12.63 | -13.70 | 28.61 | 3.25 | 0.93 | 5.95 |
| O3 | 18059 | 2,619 | 2,938 | 01/07/80 | 12/31/88 | 44.58 | 12.00 | 91.52 | 11.21 | -10.20 | 26.61 | 3.22 | 0.92 | 6.03 |
| O3 | 29123 | 143 | 580 | 01/07/80 | 11/30/82 | 41.77 | 11.47 | 80.00 | 14.98 | -5.55 | 27.68 | 3.20 | 1.02 | 5.48 |
| O3 | 44003 | 242 | 85 | 01/07/80 | 03/31/80 | 11.69 | 0.00 | 25.00 | -0.97 | -10.53 | 6.23 | 2.86 | 1.00 | 5.47 |
| O3 | 04003 | 1,035 | 763 | 01/07/80 | 10/31/88 | 29.11 | 2.00 | 60.95 | 17.60 | 4.59 | 29.03 | 4.42 | 1.94 | 6.26 |
| O3 | 18157 | 1,618 | 486 | 07/22/87 | 12/31/88 | 46.26 | 30.00 | 72.88 | 11.55 | -9.17 | 29.16 | 3.37 | 0.83 | 6.13 |
| O3 | 36031 | 2,357 | 3,051 | 01/07/80 | 12/31/88 | 42.17 | 11.00 | 76.00 | 5.37 | -17.20 | 22.07 | 2.98 | 0.89 | 5.78 |
| O3 | 18167 | 1,477 | 397 | 05/29/83 | 09/19/84 | 32.22 | 12.00 | 60.10 | 15.15 | -10.07 | 28.37 | 3.17 | 0.82 | 5.51 |
| O3 | 21227 | 261 | 126 | 10/06/82 | 09/30/83 | 30.63 | 5.62 | 52.88 | 18.22 | 1.00 | 30.60 | 3.34 | 1.08 | 5.10 |
| O3 | 33013 | 2,026 | 740 | 04/07/85 | 10/31/88 | 50.13 | 18.00 | 90.53 | 14.16 | -1.23 | 25.73 | 3.21 | 0.78 | 5.89 |
| O3 | 04001 | 230 | 298 | 06/30/86 | 09/26/88 | 46.09 | 19.00 | 109.00 | 19.39 | 10.13 | 24.59 | 4.34 | 2.28 | 5.96 |
| O3 | 26017 | 146 | 59 | 07/31/80 | 10/20/80 | 38.27 | 12.45 | 78.00 | 17.63 | 5.35 | 25.49 | 2.47 | 0.91 | 4.46 |
| O3 | 37037 | 197 | 319 | 04/12/87 | 10/31/88 | 61.23 | 25.00 | 109.05 | 21.12 | 10.39 | 29.52 | 3.55 | 1.10 | 5.80 |
| O3 | 06045 | 5,913 | 3,194 | 01/07/80 | 12/31/88 | 24.23 | 3.00 | 47.50 | 12.52 | 2.86 | 23.83 | 3.80 | 0.89 | 6.50 |
| O3 | 29165 | 953 | 3,030 | 01/07/80 | 12/31/88 | 37.94 | 7.00 | 83.00 | 12.54 | -10.60 | 29.84 | 3.17 | 0.90 | 5.94 |
| O3 | 36065 | 23,159 | 3,152 | 01/07/80 | 12/31/88 | 31.45 | 6.00 | 79.61 | 7.03 | -14.76 | 22.97 | 2.91 | 0.81 | 5.85 |
| O3 | 39007 | 3,518 | 1,580 | 03/27/80 | 10/31/88 | 55.69 | 19.48 | 95.00 | 16.69 | 3.14 | 25.77 | 2.59 | 0.88 | 5.04 |
| O3 | 21107 | 2,924 | 1,650 | 01/07/80 | 10/31/88 | 42.42 | 9.00 | 88.77 | 17.40 | -4.81 | 29.88 | 3.33 | 1.05 | 5.77 |
| O3 | 06055 | 8,397 | 2,724 | 01/07/80 | 12/31/87 | 29.93 | 13.00 | 52.00 | 15.11 | 4.97 | 26.73 | 4.24 | 1.09 | 7.04 |
| O3 | 12115 | 11,245 | 1,129 | 07/06/82 | 12/31/88 | 49.81 | 12.20 | 102.00 | 24.01 | 13.85 | 28.39 | 2.33 | 0.92 | 3.89 |
| O3 | 26065 | 12,297 | 2,210 | 01/07/80 | 10/31/88 | 35.20 | 7.00 | 75.50 | 13.34 | -10.09 | 26.29 | 3.12 | 0.97 | 5.72 |
| O3 | 42099 | 373 | 999 | 04/06/80 | 12/31/88 | 33.59 | 5.00 | 76.05 | 11.40 | -6.81 | 26.94 | 3.13 | 0.91 | 5.91 |
| O3 | 06087 | 13,958 | 3,098 | 01/07/80 | 12/27/88 | 36.36 | 11.00 | 66.43 | 13.26 | 6.92 | 19.96 | 2.55 | 0.86 | 4.45 |
| O3 | 23009 | 2,238 | 1,896 | 01/07/82 | 12/31/88 | 32.01 | 3.00 | 79.50 | 8.84 | -12.78 | 21.52 | 2.46 | 0.72 | 4.80 |
| O3 | 27163 | 2,949 | 2,752 | 01/07/80 | 12/31/88 | 36.92 | 13.00 | 69.00 | 6.84 | -19.59 | 25.85 | 2.95 | 0.80 | 5.95 |
| O3 | 45011 | 522 | 1,347 | 01/07/85 | 12/31/88 | 37.04 | 17.65 | 66.35 | 17.91 | 2.01 | 29.41 | 3.45 | 1.06 | 6.24 |
| O3 | 48233 | 1,013 | 2,214 | 01/09/80 | 12/31/88 | 38.19 | 8.00 | 95.00 | 14.44 | -5.94 | 29.16 | 4.17 | 1.26 | 7.04 |
| O3 | 22011 | 687 | 1,101 | 02/23/83 | 11/01/88 | 37.37 | 5.50 | 87.00 | 23.17 | 9.21 | 29.18 | 3.16 | 1.19 | 5.17 |
| O3 | 30111 | 1,038 | 394 | 01/13/80 | 12/31/88 | 31.30 | 2.83 | 69.00 | 7.50 | -14.56 | 24.66 | 4.00 | 1.26 | 7.10 |
| O3 | 47145 | 1,910 | 1,886 | 07/27/81 | 12/31/88 | 41.54 | 14.00 | 75.00 | 14.98 | -2.40 | 28.09 | 3.17 | 0.98 | 5.56 |
| O3 | 54029 | 50 | 172 | 07/06/87 | 12/31/87 | 37.28 | 16.27 | 63.45 | 13.36 | -2.59 | 26.28 | 2.82 | 1.02 | 4.92 |
| O3 | 39083 | 232 | 207 | 04/11/88 | 11/10/88 | 58.17 | 23.30 | 107.55 | 17.41 | 3.32 | 28.98 | 3.45 | 0.95 | 6.01 |
| O3 | 48183 | 6,057 | 2,075 | 07/06/80 | 12/31/87 | 37.56 | 0.00 | 85.00 | 18.72 | 2.33 | 30.95 | 3.37 | 0.95 | 5.83 |
| O3 | 22043 | 34 | 219 | 01/07/80 | 10/08/80 | 35.75 | 5.45 | 79.00 | 21.51 | 1.97 | 31.89 | 3.46 | 1.49 | 5.53 |
| O3 | 23015 | 802 | 1,316 | 09/17/84 | 12/31/88 | 33.01 | 12.00 | 60.00 | 7.62 | -12.45 | 22.16 | 2.52 | 0.80 | 4.69 |
| O3 | 45063 | 5,689 | 2,716 | 03/07/80 | 12/31/88 | 46.24 | 12.00 | 85.12 | 17.21 | 1.42 | 29.41 | 3.32 | 0.98 | 5.93 |
| O3 | 23003 | 678 | 336 | 01/07/80 | 10/03/82 | 41.79 | 15.38 | 75.00 | 2.59 | -18.69 | 20.86 | 3.09 | 0.97 | 5.72 |
| O3 | 51187 | 1,042 | 1,936 | 02/01/83 | 12/28/88 | 44.11 | 10.00 | 102.00 | 11.88 | -6.58 | 26.82 | 3.41 | 0.91 | 6.15 |
| O3 | 51600 | 317 | 485 | 01/07/80 | 05/26/81 | 38.86 | 7.10 | 86.00 | 11.12 | -6.81 | 27.77 | 3.14 | 1.04 | 5.57 |
| O3 | 21075 | 409 | 1,299 | 06/05/81 | 02/19/86 | 32.24 | 7.00 | 67.00 | 15.34 | -4.71 | 29.13 | 2.98 | 0.92 | 5.24 |
| O3 | 23031 | 1,170 | 434 | 06/21/83 | 12/31/88 | 39.33 | 6.00 | 76.17 | 13.32 | -5.49 | 24.95 | 2.76 | 0.95 | 4.87 |
| O3 | 24043 | 8,479 | 2,706 | 08/12/80 | 12/27/88 | 38.82 | 5.00 | 87.00 | 12.22 | -7.58 | 26.98 | 3.25 | 0.96 | 5.88 |
| O3 | 55139 | 3,181 | 1,032 | 01/07/80 | 10/15/84 | 36.11 | 0.00 | 84.00 | 11.39 | -14.85 | 25.92 | 2.85 | 0.90 | 5.45 |
| O3 | 13223 | 758 | 2,020 | 08/10/80 | 11/30/88 | 43.59 | 14.50 | 77.00 | 18.89 | 3.95 | 28.25 | 3.25 | 0.96 | 5.50 |
| O3 | 21093 | 3,083 | 2,068 | 01/07/80 | 10/31/88 | 45.65 | 9.00 | 90.00 | 17.22 | -4.30 | 28.29 | 3.33 | 1.07 | 5.85 |
| O3 | 51131 | 1,320 | 1,389 | 04/25/80 | 10/20/88 | 57.46 | 23.00 | 97.00 | 21.28 | 10.69 | 27.64 | 1.04 | 0.42 | 2.18 |
| O3 | 21049 | 1,209 | 1,889 | 03/06/80 | 12/31/88 | 35.10 | 8.00 | 72.00 | 13.75 | -5.99 | 27.51 | 3.26 | 0.92 | 5.95 |
| O3 | 21103 | 78 | 617 | 05/18/81 | 10/31/85 | 46.27 | 14.00 | 88.00 | 19.05 | 4.16 | 26.73 | 3.25 | 1.05 | 5.32 |
| O3 | 41009 | 1,221 | 2,490 | 04/08/80 | 12/31/88 | 40.13 | 5.00 | 81.78 | 9.71 | -0.53 | 21.32 | 2.99 | 0.87 | 6.34 |
| O3 | 47119 | 3,919 | 2,728 | 01/07/80 | 12/31/88 | 31.15 | 4.00 | 76.00 | 14.89 | -4.07 | 28.51 | 3.25 | 0.99 | 5.74 |
| O3 | 48361 | 83 | 79 | 07/06/80 | 10/30/80 | 46.89 | 27.90 | 66.35 | 26.45 | 18.09 | 31.43 | 2.94 | 1.27 | 4.78 |
| O3 | 12081 | 3,843 | 628 | 08/20/80 | 09/30/83 | 30.95 | 11.00 | 58.00 | 22.45 | 11.54 | 28.65 | 2.95 | 1.25 | 5.11 |
| O3 | 39073 | 1,288 | 2,300 | 01/13/80 | 10/31/88 | 33.08 | 4.00 | 80.00 | 12.60 | -9.48 | 26.97 | 3.23 | 1.03 | 5.89 |
| O3 | 51775 | 1,901 | 782 | 05/18/81 | 05/28/84 | 40.59 | 16.00 | 77.00 | 11.53 | -5.05 | 25.89 | 3.47 | 0.88 | 6.52 |
| O3 | 55133 | 3,343 | 741 | 01/07/80 | 06/30/82 | 29.45 | 0.00 | 72.00 | 8.89 | -15.58 | 24.96 | 2.75 | 0.85 | 5.48 |
| O3 | 21015 | 107 | 130 | 05/06/80 | 09/30/80 | 43.18 | 14.68 | 75.65 | 21.69 | 13.69 | 27.47 | 3.28 | 0.96 | 5.80 |
| O3 | 55127 | 2,528 | 1,785 | 01/16/80 | 10/31/88 | 43.87 | 11.00 | 102.80 | 15.61 | -7.14 | 27.43 | 3.08 | 1.05 | 5.67 |
| O3 | 55033 | 1,671 | 3,099 | 01/07/80 | 12/31/88 | 38.53 | 15.00 | 76.00 | 6.90 | -19.26 | 25.24 | 3.03 | 0.88 | 6.02 |
| O3 | 45029 | 1,758 | 2,161 | 01/07/80 | 10/26/88 | 47.73 | 18.00 | 108.00 | 19.83 | 3.12 | 29.35 | 3.04 | 1.03 | 5.23 |
| O3 | 51085 | 575 | 1,419 | 07/21/80 | 10/31/88 | 40.04 | 15.00 | 74.55 | 21.19 | 9.49 | 29.22 | 3.43 | 1.12 | 5.73 |
| O3 | 22093 | 528 | 1,909 | 07/19/83 | 12/31/88 | 35.31 | 12.00 | 64.00 | 19.99 | 5.43 | 28.31 | 2.71 | 0.90 | 4.81 |
| O3 | 48291 | 267 | 261 | 02/06/88 | 12/31/88 | 44.04 | 21.00 | 77.50 | 21.15 | 7.99 | 29.12 | 3.18 | 1.21 | 5.08 |
| O3 | 47009 | 190 | 106 | 05/08/84 | 09/23/84 | 61.25 | 29.25 | 93.12 | 20.33 | 12.30 | 24.29 | 3.66 | 1.42 | 5.91 |
| O3 | 06079 | 12,583 | 3,219 | 01/07/80 | 12/31/88 | 37.51 | 19.25 | 61.00 | 15.53 | 5.38 | 26.92 | 3.85 | 1.44 | 5.68 |
| O3 | 27037 | 4,771 | 2,568 | 01/09/80 | 10/31/88 | 31.00 | 3.00 | 83.00 | 6.99 | -19.35 | 25.78 | 2.96 | 0.81 | 6.04 |
| O3 | 37103 | 48 | 1,021 | 01/07/80 | 01/16/83 | 18.86 | 0.00 | 53.00 | 16.25 | -0.69 | 28.23 | 3.06 | 1.02 | 5.39 |
| O3 | 18173 | 348 | 557 | 03/22/87 | 12/31/88 | 34.63 | 20.00 | 56.10 | 14.95 | -4.75 | 28.88 | 3.20 | 0.88 | 5.85 |
| O3 | 51191 | 324 | 324 | 06/06/85 | 11/04/86 | 52.86 | 13.07 | 105.85 | 17.85 | 7.87 | 23.91 | 3.20 | 0.93 | 5.51 |
| O3 | 36113 | 127 | 51 | 06/12/80 | 11/02/80 | 51.39 | 8.50 | 110.50 | 17.07 | 0.66 | 23.86 | 3.15 | 1.18 | 5.65 |
| O3 | 21145 | 9,125 | 2,895 | 01/20/80 | 12/31/88 | 35.76 | 2.00 | 93.00 | 15.16 | -5.22 | 29.54 | 3.15 | 0.95 | 5.54 |
| O3 | 23001 | 776 | 257 | 01/17/88 | 12/12/88 | 53.63 | 20.00 | 96.00 | 10.06 | -9.91 | 26.22 | 2.85 | 0.79 | 5.64 |
| O3 | 55141 | 1,068 | 425 | 04/17/80 | 10/03/83 | 38.24 | 7.60 | 69.00 | 15.71 | 1.92 | 25.25 | 3.06 | 0.97 | 5.59 |
| O3 | 19103 | 1,738 | 429 | 06/18/80 | 10/31/88 | 31.29 | 6.00 | 96.30 | 12.80 | -10.74 | 29.77 | 3.39 | 1.20 | 5.96 |
| O3 | 21101 | 3,290 | 2,978 | 01/07/80 | 12/31/88 | 40.24 | 7.00 | 77.79 | 14.02 | -6.39 | 28.81 | 3.08 | 0.93 | 5.75 |
| O3 | 37123 | 61 | 139 | 02/21/82 | 09/13/82 | 28.38 | 12.45 | 49.55 | 19.92 | 3.41 | 26.81 | 3.02 | 1.17 | 5.62 |
| O3 | 55063 | 5,712 | 1,789 | 04/16/80 | 10/05/88 | 45.14 | 11.00 | 75.15 | 12.90 | -14.81 | 26.41 | 2.99 | 0.86 | 5.48 |
| O3 | 55125 | 1,002 | 2,495 | 07/06/81 | 12/31/88 | 41.51 | 6.00 | 90.00 | 5.16 | -19.71 | 23.45 | 2.71 | 0.79 | 5.26 |
| O3 | 51061 | 1,659 | 2,027 | 06/25/81 | 12/31/88 | 44.11 | 6.65 | 110.00 | 12.64 | -5.54 | 27.62 | 3.32 | 1.00 | 5.95 |
| O3 | 37077 | 1,303 | 1,681 | 01/07/80 | 04/17/85 | 31.89 | 8.00 | 68.00 | 14.01 | -3.07 | 28.34 | 3.17 | 0.96 | 5.54 |
| O3 | 55055 | 958 | 810 | 08/20/85 | 10/31/88 | 47.51 | 11.00 | 96.78 | 13.76 | -11.45 | 28.13 | 3.00 | 0.91 | 5.72 |
| O3 | 37147 | 3,060 | 919 | 10/06/82 | 10/31/88 | 35.41 | 4.00 | 76.00 | 21.95 | 11.42 | 29.11 | 3.14 | 1.09 | 5.59 |
| O3 | 22047 | 1,827 | 2,987 | 01/07/80 | 12/31/88 | 39.04 | 7.00 | 90.00 | 19.59 | 4.59 | 28.60 | 2.91 | 1.03 | 5.00 |
| O3 | 48497 | 259 | 452 | 03/06/87 | 11/30/88 | 50.58 | 22.00 | 86.00 | 22.33 | 7.34 | 31.94 | 3.51 | 1.40 | 6.04 |
| O3 | 22015 | 1,687 | 1,527 | 01/07/80 | 05/08/88 | 43.66 | 12.15 | 80.00 | 19.67 | 1.49 | 30.36 | 3.20 | 0.95 | 5.30 |
| O3 | 22121 | 31 | 171 | 05/06/83 | 10/31/83 | 58.63 | 15.25 | 97.75 | 24.12 | 16.23 | 28.48 | 2.91 | 1.25 | 4.72 |
| O3 | 24017 | 1,642 | 1,612 | 07/08/80 | 12/31/88 | 55.56 | 23.00 | 101.00 | 14.55 | -3.83 | 28.75 | 2.73 | 0.80 | 4.96 |
| O3 | 21021 | 2,293 | 2,942 | 01/07/80 | 12/31/88 | 29.89 | 8.00 | 61.00 | 13.49 | -6.25 | 27.56 | 3.21 | 0.98 | 5.87 |
| O3 | 01001 | 207 | 625 | 04/10/80 | 09/30/88 | 45.96 | 16.00 | 80.00 | 20.34 | 2.04 | 29.35 | 3.48 | 1.21 | 5.72 |
| O3 | 21177 | 238 | 276 | 01/07/80 | 04/25/81 | 30.26 | 0.00 | 67.38 | 12.96 | -7.37 | 30.44 | 3.39 | 1.20 | 6.26 |
| O3 | 37109 | 811 | 1,207 | 04/19/82 | 10/31/88 | 44.89 | 12.15 | 96.85 | 21.32 | 10.55 | 29.04 | 3.30 | 0.96 | 5.54 |
| O3 | 06069 | 306 | 784 | 01/07/80 | 09/16/86 | 36.92 | 10.00 | 85.00 | 14.25 | 4.35 | 26.14 | 4.30 | 1.44 | 6.51 |
| O3 | 37059 | 417 | 1,129 | 05/16/82 | 10/31/88 | 49.92 | 18.00 | 106.00 | 21.30 | 10.11 | 29.16 | 3.48 | 1.02 | 5.84 |
| O3 | 26037 | 63 | 150 | 04/07/80 | 10/30/80 | 36.96 | 8.72 | 76.83 | 14.63 | 2.26 | 25.49 | 3.19 | 1.12 | 6.07 |
| O3 | 06101 | 2,970 | 2,937 | 04/17/80 | 12/31/88 | 42.82 | 7.40 | 98.50 | 17.52 | 5.25 | 30.27 | 4.39 | 1.17 | 6.93 |
| O3 | 17117 | 1,771 | 1,840 | 01/07/80 | 11/01/88 | 44.76 | 12.00 | 98.51 | 17.84 | -5.17 | 29.37 | 3.26 | 1.03 | 5.63 |
| O3 | 21141 | 924 | 1,730 | 03/22/81 | 10/31/88 | 49.41 | 11.00 | 93.00 | 18.58 | -1.52 | 29.00 | 3.32 | 1.01 | 5.72 |
| O3 | 45077 | 1,568 | 1,366 | 05/04/80 | 10/31/87 | 43.52 | 16.00 | 78.00 | 18.24 | 1.50 | 27.73 | 3.38 | 0.96 | 5.75 |
| O3 | 48121 | 5,250 | 2,231 | 01/24/81 | 12/31/88 | 36.00 | 7.00 | 88.25 | 17.25 | -0.17 | 31.79 | 3.21 | 0.94 | 5.91 |
| O3 | 55087 | 766 | 273 | 05/04/80 | 04/20/81 | 29.66 | 6.80 | 62.20 | 7.85 | -19.05 | 25.07 | 3.11 | 0.96 | 6.35 |
| O3 | 55043 | 2,715 | 2,436 | 04/06/80 | 10/31/88 | 36.10 | 5.50 | 81.12 | 10.87 | -15.05 | 26.79 | 2.97 | 0.85 | 5.54 |
| O3 | 26063 | 1,092 | 1,320 | 01/07/80 | 10/31/85 | 39.34 | 12.00 | 76.00 | 11.71 | -11.73 | 24.68 | 2.14 | 0.68 | 4.60 |
| O3 | 21117 | 183 | 51 | 01/07/80 | 03/31/80 | 27.80 | 5.25 | 50.00 | 2.87 | -6.94 | 10.27 | 2.97 | 1.07 | 6.20 |
| O3 | 21191 | 35 | 475 | 05/17/81 | 10/31/83 | 44.40 | 16.00 | 74.00 | 20.27 | 9.22 | 28.38 | 3.34 | 1.21 | 5.49 |
| O3 | 22077 | 339 | 736 | 05/06/83 | 12/31/88 | 44.82 | 8.75 | 92.00 | 22.12 | 8.31 | 28.69 | 2.98 | 1.10 | 5.19 |
| O3 | 39135 | 968 | 2,667 | 01/07/80 | 10/31/88 | 38.12 | 8.00 | 81.00 | 12.20 | -10.52 | 26.52 | 3.23 | 0.94 | 6.06 |
| O3 | 18083 | 2,542 | 1,656 | 04/20/84 | 12/31/88 | 48.83 | 17.00 | 95.00 | 13.80 | -7.29 | 28.22 | 3.15 | 0.84 | 5.95 |
| O3 | 51015 | 314 | 860 | 01/07/85 | 12/31/88 | 31.39 | 1.00 | 98.00 | 12.01 | -7.00 | 25.93 | 3.53 | 0.90 | 6.38 |
| O3 | 33007 | 2,396 | 2,335 | 05/06/81 | 12/31/88 | 39.21 | 6.00 | 84.00 | 5.12 | -17.73 | 20.96 | 2.98 | 0.82 | 5.82 |
| O3 | 48071 | 570 | 2,064 | 09/07/81 | 12/31/88 | 36.79 | 8.00 | 76.42 | 20.25 | 6.19 | 29.22 | 2.52 | 0.93 | 4.68 |
| O3 | 55027 | 3,365 | 1,704 | 05/06/82 | 10/31/88 | 38.95 | 6.00 | 87.42 | 11.30 | -15.77 | 26.96 | 3.01 | 0.92 | 5.70 |
| O3 | 08069 | 8,118 | 3,205 | 01/07/80 | 12/31/88 | 38.14 | 13.50 | 66.50 | 3.67 | -13.11 | 18.08 | 4.23 | 1.47 | 6.80 |
| O3 | 24045 | 5,802 | 1,662 | 03/06/84 | 12/23/88 | 48.48 | 22.00 | 83.00 | 14.46 | -3.44 | 28.20 | 2.61 | 0.97 | 4.75 |
| O3 | 06109 | 346 | 358 | 04/20/87 | 10/15/88 | 48.54 | 16.00 | 96.08 | 15.18 | 1.60 | 22.69 | 4.96 | 2.12 | 6.74 |
| O3 | 30093 | 256 | 210 | 01/13/80 | 12/22/80 | 32.84 | 4.00 | 77.78 | 1.49 | -10.88 | 14.54 | 3.92 | 1.41 | 6.75 |
| O3 | 56039 | 99 | 597 | 04/12/87 | 12/31/88 | 35.78 | 6.00 | 85.20 | 3.23 | -15.24 | 16.97 | 3.99 | 1.29 | 6.81 |
| O3 | 48367 | 298 | 321 | 02/01/88 | 12/31/88 | 33.65 | 13.00 | 53.00 | 20.21 | 4.73 | 31.97 | 3.77 | 1.53 | 6.59 |
| O3 | 30023 | 121 | 318 | 01/13/80 | 12/30/80 | 16.24 | 0.00 | 40.00 | 1.01 | -16.18 | 14.63 | 4.00 | 1.50 | 6.93 |
| O3 | 18169 | 137 | 179 | 07/06/88 | 12/31/88 | 34.75 | 13.90 | 65.75 | 13.34 | -7.72 | 29.17 | 3.15 | 0.86 | 5.66 |
| O3 | 53009 | 719 | 508 | 03/06/87 | 11/12/88 | 49.23 | 22.00 | 82.00 | 10.44 | 3.12 | 18.72 | 2.36 | 0.83 | 4.47 |
| O3 | 55037 | 23 | 354 | 10/06/87 | 12/31/88 | 41.18 | 2.48 | 86.17 | 2.21 | -20.91 | 24.50 | 3.15 | 0.93 | 6.40 |
| O3 | 49047 | 399 | 1,648 | 01/07/83 | 11/07/88 | 34.86 | 10.00 | 68.83 | 7.50 | -11.09 | 24.06 | 4.02 | 1.66 | 6.21 |
| O3 | 37145 | 874 | 1,584 | 10/06/82 | 12/12/87 | 38.03 | 9.57 | 71.00 | 14.22 | -1.89 | 27.84 | 3.22 | 0.94 | 5.62 |
| O3 | 21019 | 473 | 290 | 05/05/80 | 03/31/81 | 46.10 | 11.45 | 82.78 | 12.16 | -8.16 | 27.32 | 3.21 | 0.95 | 6.17 |
| O3 | 08077 | 2,129 | 901 | 01/23/86 | 12/31/88 | 54.54 | 20.00 | 102.50 | 7.85 | -11.31 | 22.03 | 4.51 | 1.73 | 6.59 |
| O3 | 47187 | 178 | 181 | 04/10/88 | 10/31/88 | 47.93 | 16.00 | 109.00 | 21.34 | 9.76 | 30.01 | 3.68 | 1.54 | 5.90 |
| O3 | 25015 | 6,270 | 2,676 | 01/29/81 | 12/31/88 | 40.44 | 12.50 | 73.56 | 8.48 | -11.31 | 24.48 | 3.18 | 0.85 | 5.79 |
| O3 | 47041 | 36 | 87 | 10/06/88 | 12/31/88 | 31.94 | 14.15 | 54.85 | 8.76 | -1.84 | 16.05 | 3.03 | 1.03 | 5.43 |
| O3 | 22005 | 631 | 869 | 04/13/83 | 10/28/88 | 41.83 | 8.00 | 83.00 | 23.80 | 14.02 | 28.73 | 2.80 | 1.10 | 4.75 |
| O3 | 24027 | 42 | 43 | 06/15/80 | 09/16/80 | 68.65 | 33.05 | 116.75 | 24.06 | 18.11 | 28.25 | 3.26 | 1.57 | 5.16 |
| O3 | 48339 | 226 | 103 | 09/05/88 | 12/31/88 | 27.32 | 12.55 | 54.90 | 19.43 | 7.68 | 28.20 | 3.29 | 0.97 | 5.35 |
| O3 | 55031 | 454 | 468 | 01/07/80 | 04/25/81 | 37.96 | 10.68 | 73.49 | 2.69 | -20.92 | 22.66 | 3.07 | 0.81 | 6.23 |
| O3 | 21185 | 40 | 181 | 04/22/81 | 11/16/81 | 46.40 | 19.50 | 76.00 | 18.54 | 6.32 | 26.86 | 3.07 | 1.06 | 5.32 |
| O3 | 32510 | 1,507 | 1,713 | 03/05/81 | 10/31/88 | 40.72 | 12.00 | 78.00 | 14.96 | -0.88 | 25.63 | 4.11 | 1.61 | 6.24 |
| O3 | 28045 | 306 | 521 | 03/11/87 | 11/30/88 | 48.01 | 9.00 | 111.00 | 22.46 | 9.97 | 28.75 | 2.68 | 0.93 | 4.70 |
| O3 | 15001 | 680 | 331 | 06/11/87 | 10/03/88 | 54.73 | 22.00 | 99.75 | 17.86 | 15.11 | 19.74 | 1.99 | 0.91 | 2.76 |
| O3 | 17083 | 181 | 337 | 01/09/88 | 12/31/88 | 68.86 | 31.00 | 128.00 | 13.18 | -7.61 | 30.32 | 3.34 | 1.04 | 5.83 |
| O3 | 31041 | 210 | 504 | 06/08/80 | 10/31/81 | 43.57 | 25.00 | 71.00 | 13.48 | -7.58 | 28.74 | 3.84 | 1.10 | 6.56 |
| O3 | 17033 | 324 | 646 | 01/07/80 | 01/21/82 | 33.04 | 5.00 | 81.00 | 12.42 | -9.57 | 28.57 | 3.14 | 1.01 | 6.08 |
| O3 | 51113 | 308 | 1,892 | 01/27/83 | 12/31/88 | 32.90 | 2.00 | 99.72 | 12.70 | -5.33 | 27.25 | 3.43 | 0.95 | 6.11 |
| O3 | 08003 | 130 | 447 | 06/28/87 | 12/31/88 | 42.78 | 28.00 | 58.00 | 7.68 | -12.54 | 18.97 | 4.94 | 2.22 | 6.98 |
| O3 | 21061 | 133 | 1,681 | 01/07/84 | 12/31/88 | 52.59 | 26.00 | 96.00 | 14.14 | -5.08 | 28.16 | 3.21 | 0.95 | 5.87 |
| O3 | 06011 | 883 | 3,046 | 01/07/80 | 12/31/88 | 35.74 | 8.00 | 71.00 | 16.92 | 4.42 | 30.68 | 4.31 | 1.10 | 6.82 |
| O3 | 13191 | 22 | 311 | 01/11/87 | 01/31/88 | 35.57 | 17.00 | 61.00 | 18.94 | 4.83 | 29.09 | 2.42 | 0.80 | 4.31 |
| O3 | 21035 | 1,480 | 1,917 | 05/18/80 | 10/31/88 | 49.88 | 14.00 | 90.00 | 19.73 | 1.28 | 29.68 | 3.10 | 1.03 | 5.22 |
| O3 | 18109 | 897 | 1,127 | 04/13/84 | 10/12/87 | 50.35 | 10.00 | 105.85 | 13.86 | -8.68 | 26.82 | 3.10 | 0.89 | 5.71 |
| O3 | 04011 | 105 | 912 | 05/11/80 | 09/30/85 | 53.11 | 35.77 | 75.00 | 19.88 | 9.11 | 26.00 | 4.92 | 2.41 | 7.16 |
| O3 | 21183 | 836 | 1,716 | 10/06/80 | 10/31/88 | 46.65 | 14.00 | 84.00 | 18.74 | -0.60 | 28.94 | 3.28 | 1.05 | 5.68 |
| O3 | 21169 | 132 | 1,705 | 06/07/81 | 12/31/88 | 14.92 | 0.00 | 52.00 | 14.28 | -4.62 | 27.87 | 3.13 | 0.96 | 5.70 |
| O3 | 48397 | 81 | 179 | 07/06/88 | 12/31/88 | 44.07 | 18.00 | 76.55 | 21.18 | 5.66 | 32.55 | 3.34 | 0.97 | 5.31 |
| O3 | 50025 | 1,396 | 1,426 | 11/11/82 | 08/26/88 | 36.44 | 19.00 | 58.00 | 6.42 | -14.25 | 22.43 | 3.16 | 0.74 | 5.98 |
| O3 | 50001 | 1,255 | 2,785 | 01/07/80 | 12/31/88 | 42.17 | 7.00 | 82.00 | 6.28 | -15.88 | 22.91 | 3.03 | 0.89 | 6.01 |
| O3 | 26043 | 1,482 | 1,300 | 01/07/85 | 12/31/88 | 49.74 | 10.00 | 95.00 | 6.13 | -17.20 | 24.03 | 3.17 | 0.94 | 6.30 |
| O3 | 26133 | 110 | 226 | 06/15/83 | 09/16/84 | 36.92 | 9.62 | 80.75 | 19.12 | 8.26 | 25.84 | 3.26 | 1.25 | 5.17 |
| O3 | 48109 | 33 | 503 | 04/12/87 | 11/15/88 | 33.78 | 16.00 | 59.90 | 18.02 | 1.24 | 28.63 | 4.27 | 1.86 | 6.54 |
| O3 | 13247 | 74 | 147 | 01/07/80 | 06/08/80 | 47.16 | 9.95 | 86.00 | 12.48 | -1.22 | 25.12 | 3.51 | 1.00 | 6.06 |
| O3 | 38057 | 391 | 3,001 | 06/12/80 | 10/31/88 | 42.84 | 8.00 | 90.50 | 6.69 | -22.02 | 26.37 | 3.42 | 0.86 | 6.62 |
| O3 | 06043 | 34 | 81 | 07/09/86 | 10/06/86 | 48.33 | 22.00 | 93.00 | 19.27 | 8.39 | 25.16 | 4.71 | 2.18 | 6.22 |
| O3 | 06033 | 2,890 | 2,431 | 01/07/80 | 12/31/88 | 45.57 | 12.00 | 88.00 | 13.73 | 2.14 | 27.88 | 4.27 | 0.96 | 6.86 |
| O3 | 55083 | 477 | 719 | 04/15/80 | 10/11/84 | 40.64 | 14.00 | 72.05 | 16.16 | 5.03 | 25.61 | 3.05 | 1.04 | 5.47 |
| O3 | 21043 | 309 | 1,425 | 05/01/83 | 11/30/88 | 44.55 | 14.00 | 82.00 | 17.73 | 2.57 | 28.11 | 3.37 | 0.99 | 5.96 |
| O3 | 55099 | 1,170 | 2,676 | 01/09/80 | 10/31/88 | 36.49 | 8.00 | 89.00 | 7.23 | -19.36 | 23.92 | 3.01 | 0.86 | 5.81 |
| O3 | 20087 | 106 | 385 | 06/22/82 | 09/25/86 | 81.16 | 24.20 | 115.40 | 23.17 | 13.03 | 30.39 | 3.10 | 1.24 | 4.99 |
| O3 | 21221 | 104 | 459 | 05/18/81 | 10/30/83 | 44.54 | 12.00 | 77.55 | 21.84 | 11.24 | 30.05 | 3.06 | 0.98 | 4.78 |
| O3 | 32005 | 32 | 286 | 07/01/82 | 10/31/83 | 43.97 | 15.25 | 91.50 | 15.06 | 2.77 | 22.60 | 4.47 | 1.77 | 6.56 |
| O3 | 30075 | 49 | 1,157 | 02/26/80 | 10/31/88 | 35.62 | 0.00 | 88.10 | 15.56 | -1.91 | 28.05 | 4.37 | 1.45 | 7.25 |
| O3 | 21139 | 80 | 260 | 05/06/81 | 10/31/82 | 45.18 | 12.00 | 83.05 | 21.01 | 8.92 | 28.19 | 2.98 | 1.09 | 4.55 |
| O3 | 06051 | 263 | 1,999 | 08/06/81 | 12/31/88 | 39.51 | 9.00 | 96.00 | 6.12 | -10.66 | 20.56 | 3.91 | 1.50 | 5.80 |

**Note:** Geographic units are identified by NCHS county-level codes. Total deaths and observation days are counts. Exposure concentration, daily mean temperature (tave), and temperature variability (tsd) are summarized by the mean, 2.5th percentile, and 97.5th percentile. PM_2.5_ concentrations are reported in µg/m³; the erroneous 1000-fold scaling in the source PM_2.5_ Table 1 export has been reversed. O_3_ concentrations are reported in ppb.
