# Supplementary material for: Modelling beyond additive risks: A framework for estimating the cascading burden of environmental exposure
Source: Eco Environ Health. 2026 Sep 1;5(4):100281. doi: 10.1016/j.eehl.2026.100281 (PMC13587907; doi:10.1016/j.eehl.2026.100281)
Supplement: Supplementary file 3 [file mmc3.docx]

**Table S2.** Geographic-unit-specific attributable fractions for all-cause mortality in the PM_2.5_ and O_3_ analyses

| **Pollutant** | **Geographic unit** | **Median annual deaths** | **iDLM attributable fraction (%)** | | | **Standard DLM AF (%)** | **95% CI: iDLM total AF** | | **95% CI: standard DLM AF** | |
| --- | --- | --- | --- | --- | --- | --- | --- | --- | --- | --- |
|  |  |  | **Main** | **Total** | **Cascading** | **Estimate** | **Lower** | **Upper** | **Lower** | **Upper** |
| PM2.5 | 42129 | 3,564 | -0.0884 | -0.0552 | 0.0332 | -0.1035 | -0.1832 | 0.0751 | -0.0023 | 0.0003 |
| PM2.5 | 13115 | 958 | -0.0823 | -0.0365 | 0.0457 | -0.1001 | -0.1747 | 0.1015 | -0.0023 | 0.0004 |
| PM2.5 | 42051 | 1,464 | -0.0812 | -0.0662 | 0.0149 | -0.0921 | -0.1632 | 0.0322 | -0.0019 | 0.0002 |
| PM2.5 | 29037 | 272 | -0.0759 | -0.0584 | 0.0175 | -0.0876 | -0.1546 | 0.0395 | -0.0019 | 0.0003 |
| PM2.5 | 42007 | 1,638 | -0.0757 | -0.0317 | 0.0440 | -0.0874 | -0.1517 | 0.0894 | -0.0019 | 0.0003 |
| PM2.5 | 49011 | 483 | 0.0046 | -0.0606 | -0.0653 | 0.0194 | -0.1463 | 0.0313 | -0.0006 | 0.0009 |
| PM2.5 | 42005 | 663 | -0.0479 | -0.0402 | 0.0077 | -0.0622 | -0.1210 | 0.0432 | -0.0016 | 0.0004 |
| PM2.5 | 02090 | 216 | -0.0445 | 0.1959 | 0.2404 | -0.0569 | -0.0903 | 0.4971 | -0.0014 | 0.0004 |
| PM2.5 | 47065 | 2,916 | -0.0353 | -0.0278 | 0.0075 | -0.0444 | -0.0874 | 0.0333 | -0.0011 | 0.0003 |
| PM2.5 | 53035 | 991 | -0.0461 | -0.0312 | 0.0149 | -0.0503 | -0.0836 | 0.0240 | -0.0010 | 0.0001 |
| PM2.5 | 39099 | 2,911 | -0.0207 | -0.0040 | 0.0167 | -0.0353 | -0.0828 | 0.0698 | -0.0011 | 0.0005 |
| PM2.5 | 13077 | 367 | -0.0185 | 0.3553 | 0.3742 | -0.0208 | -0.0788 | 0.8130 | -0.0004 | 0.0000 |
| PM2.5 | 01119 | 116 | -0.0449 | 0.0028 | 0.0476 | -0.0509 | -0.0710 | 0.0789 | -0.0011 | 0.0001 |
| PM2.5 | 42125 | 2,242 | -0.0343 | -0.0306 | 0.0038 | -0.0405 | -0.0690 | 0.0109 | -0.0009 | 0.0001 |
| PM2.5 | 41059 | 448 | -0.0452 | 0.0839 | 0.1301 | -0.0512 | -0.0668 | 0.2437 | -0.0011 | 0.0001 |
| PM2.5 | 06019 | 4,308 | -0.0148 | 0.4192 | 0.4360 | -0.0170 | -0.0667 | 0.9129 | -0.0004 | 0.0000 |
| PM2.5 | 13223 | 152 | 0.0013 | 0.2910 | 0.2901 | 0.0005 | -0.0517 | 0.6493 | -0.0001 | 0.0001 |
| PM2.5 | 53065 | 182 | -0.0195 | 0.1512 | 0.1711 | -0.0213 | -0.0471 | 0.3470 | -0.0004 | 0.0000 |
| PM2.5 | 06039 | 438 | -0.0093 | 0.2801 | 0.2906 | -0.0102 | -0.0456 | 0.6176 | -0.0002 | 0.0000 |
| PM2.5 | 28075 | 987 | -0.0238 | 0.0213 | 0.0451 | -0.0265 | -0.0398 | 0.0832 | -0.0005 | 0.0000 |
| PM2.5 | 48203 | 463 | -0.0014 | 0.2126 | 0.2139 | -0.0026 | -0.0388 | 0.4783 | -0.0001 | 0.0000 |
| PM2.5 | 53033 | 10,252 | -0.0246 | 0.0366 | 0.0613 | -0.0276 | -0.0371 | 0.1127 | -0.0006 | 0.0001 |
| PM2.5 | 06031 | 490 | -0.0132 | 0.3732 | 0.3882 | -0.0162 | -0.0371 | 0.7765 | -0.0004 | 0.0001 |
| PM2.5 | 48323 | 128 | -0.0068 | 0.0698 | 0.0768 | -0.0076 | -0.0316 | 0.1720 | -0.0002 | 0.0000 |
| PM2.5 | 16009 | 62 | -0.0185 | 0.0217 | 0.0402 | -0.0216 | -0.0314 | 0.0778 | -0.0005 | 0.0001 |
| PM2.5 | 01073 | 8,091 | 0.0004 | 0.2125 | 0.2122 | -0.0001 | -0.0304 | 0.4695 | 0.0000 | 0.0000 |
| PM2.5 | 41061 | 186 | -0.0352 | 0.1252 | 0.1607 | -0.0394 | -0.0282 | 0.2744 | -0.0008 | 0.0001 |
| PM2.5 | 41025 | 64 | -0.0149 | 0.0281 | 0.0430 | -0.0166 | -0.0276 | 0.0861 | -0.0003 | 0.0000 |
| PM2.5 | 51087 | 1,685 | -0.0106 | 0.0863 | 0.0971 | -0.0135 | -0.0260 | 0.2033 | -0.0003 | 0.0001 |
| PM2.5 | 22103 | 698 | -0.0052 | 0.1586 | 0.1643 | -0.0047 | -0.0248 | 0.3481 | -0.0001 | 0.0000 |
| PM2.5 | 53071 | 542 | -0.0172 | 0.0310 | 0.0483 | -0.0196 | -0.0234 | 0.0875 | -0.0004 | 0.0000 |
| PM2.5 | 13151 | 229 | -0.0081 | 0.2964 | 0.3051 | -0.0069 | -0.0225 | 0.6194 | -0.0001 | 0.0000 |
| PM2.5 | 37173 | 127 | 0.0025 | 0.1329 | 0.1306 | 0.0037 | -0.0215 | 0.2911 | 0.0000 | 0.0001 |
| PM2.5 | 42095 | 1,364 | -0.0224 | 0.1291 | 0.1516 | -0.0262 | -0.0209 | 0.2894 | -0.0006 | 0.0001 |
| PM2.5 | 39061 | 9,320 | -0.0131 | -0.0051 | 0.0081 | -0.0151 | -0.0209 | 0.0120 | -0.0003 | 0.0000 |
| PM2.5 | 31157 | 380 | -0.0101 | -0.0032 | 0.0069 | -0.0124 | -0.0207 | 0.0143 | -0.0003 | 0.0001 |
| PM2.5 | 16083 | 478 | -0.0038 | 0.1102 | 0.1139 | -0.0048 | -0.0200 | 0.2475 | -0.0001 | 0.0000 |
| PM2.5 | 34039 | 4,868 | -0.0132 | 0.0705 | 0.0838 | -0.0156 | -0.0196 | 0.1656 | -0.0003 | 0.0001 |
| PM2.5 | 18141 | 2,371 | 0.0036 | 0.2425 | 0.2394 | 0.0040 | -0.0193 | 0.5172 | 0.0000 | 0.0001 |
| PM2.5 | 42081 | 1,231 | -0.0131 | 0.0178 | 0.0309 | -0.0156 | -0.0186 | 0.0553 | -0.0004 | 0.0001 |
| PM2.5 | 13135 | 634 | -0.0098 | 0.1246 | 0.1345 | -0.0126 | -0.0186 | 0.2710 | -0.0003 | 0.0001 |
| PM2.5 | 42011 | 3,069 | -0.0125 | 0.0258 | 0.0383 | -0.0144 | -0.0185 | 0.0714 | -0.0003 | 0.0000 |
| PM2.5 | 39103 | 659 | 0.0120 | 0.1212 | 0.1093 | 0.0125 | -0.0184 | 0.2647 | 0.0000 | 0.0002 |
| PM2.5 | 53011 | 1,216 | -0.0098 | 0.0241 | 0.0339 | -0.0111 | -0.0182 | 0.0676 | -0.0002 | 0.0000 |
| PM2.5 | 34017 | 5,366 | -0.0107 | 0.1049 | 0.1158 | -0.0127 | -0.0181 | 0.2309 | -0.0003 | 0.0000 |
| PM2.5 | 34041 | 737 | -0.0153 | 0.0426 | 0.0579 | -0.0183 | -0.0178 | 0.1057 | -0.0004 | 0.0001 |
| PM2.5 | 06067 | 6,282 | 0.0043 | 0.1447 | 0.1408 | 0.0051 | -0.0174 | 0.3116 | 0.0000 | 0.0001 |
| PM2.5 | 13297 | 204 | -0.0084 | 0.1631 | 0.1718 | -0.0092 | -0.0173 | 0.3435 | -0.0002 | 0.0000 |
| PM2.5 | 49049 | 960 | 0.2691 | 1.2379 | 0.9723 | 0.3556 | -0.0165 | 2.4775 | -0.0027 | 0.0091 |
| PM2.5 | 53009 | 459 | -0.0089 | -0.0061 | 0.0028 | -0.0101 | -0.0163 | 0.0047 | -0.0002 | 0.0000 |
| PM2.5 | 36029 | 10,786 | -0.0023 | 0.0763 | 0.0786 | -0.0028 | -0.0161 | 0.1732 | -0.0001 | 0.0000 |
| PM2.5 | 41051 | 6,179 | -0.0113 | 0.0359 | 0.0473 | -0.0136 | -0.0161 | 0.0910 | -0.0003 | 0.0001 |
| PM2.5 | 06043 | 90 | 0.0067 | 0.1849 | 0.1783 | 0.0091 | -0.0157 | 0.3900 | -0.0001 | 0.0002 |
| PM2.5 | 16069 | 328 | -0.0101 | 0.0127 | 0.0229 | -0.0116 | -0.0146 | 0.0417 | -0.0002 | 0.0000 |
| PM2.5 | 26099 | 3,952 | -0.0049 | 0.0360 | 0.0409 | -0.0058 | -0.0128 | 0.0870 | -0.0001 | 0.0000 |
| PM2.5 | 48485 | 1,110 | -0.0037 | 0.0542 | 0.0580 | -0.0042 | -0.0126 | 0.1246 | -0.0001 | 0.0000 |
| PM2.5 | 41037 | 60 | -0.0105 | -0.0049 | 0.0055 | -0.0097 | -0.0120 | 0.0023 | -0.0002 | 0.0000 |
| PM2.5 | 42089 | 639 | -0.0111 | 0.0327 | 0.0438 | -0.0123 | -0.0117 | 0.0798 | -0.0003 | 0.0000 |
| PM2.5 | 24033 | 3,301 | -0.0039 | 0.1480 | 0.1522 | -0.0050 | -0.0114 | 0.3082 | -0.0001 | 0.0000 |
| PM2.5 | 53047 | 266 | -0.0015 | 0.0297 | 0.0312 | -0.0014 | -0.0112 | 0.0719 | 0.0000 | 0.0000 |
| PM2.5 | 26017 | 911 | -0.0054 | 0.0159 | 0.0213 | -0.0069 | -0.0105 | 0.0442 | -0.0002 | 0.0000 |
| PM2.5 | 06113 | 782 | 0.0077 | 0.3967 | 0.3907 | 0.0078 | -0.0100 | 0.8060 | 0.0000 | 0.0001 |
| PM2.5 | 01089 | 1,593 | -0.0006 | 0.0522 | 0.0527 | -0.0007 | -0.0094 | 0.1165 | 0.0000 | 0.0000 |
| PM2.5 | 48141 | 2,730 | -0.0063 | 0.0139 | 0.0201 | -0.0079 | -0.0094 | 0.0394 | -0.0002 | 0.0000 |
| PM2.5 | 47157 | 8,640 | -0.0004 | 0.0771 | 0.0775 | -0.0006 | -0.0087 | 0.1668 | 0.0000 | 0.0000 |
| PM2.5 | 01033 | 468 | -0.0028 | 0.0293 | 0.0322 | -0.0032 | -0.0086 | 0.0684 | -0.0001 | 0.0000 |
| PM2.5 | 53075 | 177 | -0.0062 | 0.0003 | 0.0065 | -0.0068 | -0.0084 | 0.0094 | -0.0001 | 0.0000 |
| PM2.5 | 06101 | 367 | -0.0025 | 0.1565 | 0.1592 | -0.0023 | -0.0084 | 0.3246 | 0.0000 | 0.0000 |
| PM2.5 | 48439 | 6,788 | -0.0023 | 0.0237 | 0.0260 | -0.0027 | -0.0083 | 0.0568 | -0.0001 | 0.0000 |
| PM2.5 | 53021 | 198 | -0.0054 | 0.0091 | 0.0145 | -0.0066 | -0.0082 | 0.0269 | -0.0002 | 0.0000 |
| PM2.5 | 12009 | 2,135 | -0.0068 | 0.0178 | 0.0246 | -0.0078 | -0.0082 | 0.0454 | -0.0002 | 0.0000 |
| PM2.5 | 46103 | 567 | -0.0030 | 0.0123 | 0.0153 | -0.0026 | -0.0076 | 0.0322 | -0.0001 | 0.0000 |
| PM2.5 | 53003 | 148 | -0.0073 | 0.0719 | 0.0793 | -0.0080 | -0.0075 | 0.1536 | -0.0002 | 0.0000 |
| PM2.5 | 41019 | 709 | -0.0026 | 0.0137 | 0.0164 | -0.0039 | -0.0070 | 0.0343 | -0.0001 | 0.0001 |
| PM2.5 | 16019 | 478 | -0.0020 | 0.0417 | 0.0438 | -0.0021 | -0.0070 | 0.0930 | 0.0000 | 0.0000 |
| PM2.5 | 08103 | 36 | -0.0034 | 0.0174 | 0.0208 | -0.0043 | -0.0069 | 0.0429 | -0.0001 | 0.0000 |
| PM2.5 | 16055 | 448 | 0.0042 | 0.0711 | 0.0670 | 0.0055 | -0.0069 | 0.1519 | 0.0000 | 0.0001 |
| PM2.5 | 12111 | 841 | -0.0083 | 0.0316 | 0.0399 | -0.0093 | -0.0069 | 0.0724 | -0.0002 | 0.0000 |
| PM2.5 | 53027 | 591 | -0.0038 | 0.0094 | 0.0133 | -0.0047 | -0.0066 | 0.0261 | -0.0001 | 0.0000 |
| PM2.5 | 39095 | 5,127 | 0.0023 | 0.1043 | 0.1021 | 0.0019 | -0.0065 | 0.2185 | 0.0000 | 0.0000 |
| PM2.5 | 51760 | 4,044 | -0.0071 | 0.0561 | 0.0632 | -0.0093 | -0.0061 | 0.1212 | -0.0002 | 0.0001 |
| PM2.5 | 17201 | 2,220 | -0.0057 | 0.0261 | 0.0319 | -0.0075 | -0.0058 | 0.0595 | -0.0002 | 0.0001 |
| PM2.5 | 53061 | 2,015 | -0.0077 | 0.0700 | 0.0776 | -0.0086 | -0.0058 | 0.1480 | -0.0002 | 0.0000 |
| PM2.5 | 42115 | 277 | 0.0004 | 0.0392 | 0.0388 | 0.0000 | -0.0057 | 0.0851 | 0.0000 | 0.0000 |
| PM2.5 | 23017 | 460 | 0.0076 | 0.0883 | 0.0808 | 0.0088 | -0.0057 | 0.1852 | 0.0000 | 0.0002 |
| PM2.5 | 34001 | 2,685 | 0.0009 | 0.0892 | 0.0885 | 0.0009 | -0.0057 | 0.1882 | 0.0000 | 0.0000 |
| PM2.5 | 48375 | 1,650 | -0.0025 | 0.0172 | 0.0198 | -0.0032 | -0.0056 | 0.0411 | -0.0001 | 0.0000 |
| PM2.5 | 30087 | 54 | -0.0055 | 0.0290 | 0.0345 | -0.0060 | -0.0054 | 0.0648 | -0.0001 | 0.0000 |
| PM2.5 | 35013 | 444 | -0.0047 | 0.0202 | 0.0249 | -0.0053 | -0.0054 | 0.0459 | -0.0001 | 0.0000 |
| PM2.5 | 06009 | 176 | -0.0047 | 0.0178 | 0.0226 | -0.0064 | -0.0053 | 0.0421 | -0.0002 | 0.0000 |
| PM2.5 | 48453 | 2,723 | -0.0008 | 0.0402 | 0.0411 | -0.0013 | -0.0052 | 0.0869 | 0.0000 | 0.0000 |
| PM2.5 | 51069 | 71 | -0.0085 | 0.0184 | 0.0269 | -0.0083 | -0.0050 | 0.0425 | -0.0001 | 0.0000 |
| PM2.5 | 51770 | 1,581 | -0.0009 | 0.0650 | 0.0660 | -0.0015 | -0.0049 | 0.1357 | 0.0000 | 0.0000 |
| PM2.5 | 39003 | 1,124 | -0.0214 | 0.2119 | 0.2331 | -0.0246 | -0.0047 | 0.4304 | -0.0005 | 0.0001 |
| PM2.5 | 32003 | 3,480 | -0.0018 | 0.0020 | 0.0037 | -0.0024 | -0.0047 | 0.0085 | -0.0001 | 0.0000 |
| PM2.5 | 16077 | 39 | -0.0222 | 0.6838 | 0.7031 | -0.0220 | -0.0046 | 1.3529 | -0.0005 | 0.0001 |
| PM2.5 | 46033 | 49 | -0.0020 | 0.0195 | 0.0216 | -0.0024 | -0.0046 | 0.0447 | -0.0001 | 0.0000 |
| PM2.5 | 08029 | 203 | -0.0020 | -0.0003 | 0.0017 | -0.0025 | -0.0043 | 0.0038 | -0.0001 | 0.0000 |
| PM2.5 | 06037 | 60,870 | 0.0035 | 0.2275 | 0.2242 | 0.0037 | -0.0042 | 0.4662 | 0.0000 | 0.0001 |
| PM2.5 | 42117 | 352 | -0.0035 | 0.0127 | 0.0162 | -0.0039 | -0.0042 | 0.0298 | -0.0001 | 0.0000 |
| PM2.5 | 48243 | 8 | 0.0012 | 0.0191 | 0.0178 | 0.0031 | -0.0041 | 0.0437 | -0.0001 | 0.0001 |
| PM2.5 | 40135 | 197 | -0.0081 | 0.0245 | 0.0327 | -0.0068 | -0.0039 | 0.0540 | -0.0001 | 0.0000 |
| PM2.5 | 35025 | 353 | 0.0000 | 0.0209 | 0.0209 | -0.0001 | -0.0037 | 0.0471 | 0.0000 | 0.0000 |
| PM2.5 | 51059 | 1,979 | -0.0010 | 0.0320 | 0.0330 | -0.0011 | -0.0035 | 0.0690 | 0.0000 | 0.0000 |
| PM2.5 | 41011 | 553 | -0.0023 | 0.0020 | 0.0043 | -0.0027 | -0.0034 | 0.0077 | -0.0001 | 0.0000 |
| PM2.5 | 15003 | 4,020 | 0.0007 | 0.0165 | 0.0157 | 0.0009 | -0.0032 | 0.0365 | 0.0000 | 0.0000 |
| PM2.5 | 51161 | 360 | 0.0013 | 0.0203 | 0.0190 | 0.0005 | -0.0030 | 0.0449 | 0.0000 | 0.0000 |
| PM2.5 | 53001 | 89 | -0.0136 | 0.0284 | 0.0418 | -0.0129 | -0.0029 | 0.0581 | -0.0002 | 0.0000 |
| PM2.5 | 24027 | 424 | -0.0005 | 0.0080 | 0.0085 | 0.0001 | -0.0029 | 0.0196 | 0.0000 | 0.0000 |
| PM2.5 | 48303 | 1,784 | -0.0015 | 0.0107 | 0.0122 | -0.0016 | -0.0026 | 0.0245 | 0.0000 | 0.0000 |
| PM2.5 | 44007 | 6,737 | 0.0002 | 0.0347 | 0.0345 | -0.0002 | -0.0024 | 0.0726 | 0.0000 | 0.0000 |
| PM2.5 | 23003 | 730 | 0.0007 | 0.0182 | 0.0175 | 0.0001 | -0.0023 | 0.0396 | 0.0000 | 0.0000 |
| PM2.5 | 06001 | 9,448 | 0.0051 | 0.1910 | 0.1872 | 0.0055 | -0.0022 | 0.3756 | 0.0000 | 0.0001 |
| PM2.5 | 53005 | 534 | 0.0017 | 0.0250 | 0.0233 | 0.0014 | -0.0021 | 0.0529 | 0.0000 | 0.0000 |
| PM2.5 | 53045 | 211 | 0.0018 | 0.0182 | 0.0163 | 0.0009 | -0.0021 | 0.0387 | -0.0001 | 0.0001 |
| PM2.5 | 46127 | 54 | -0.0084 | 0.0771 | 0.0856 | -0.0106 | -0.0020 | 0.1596 | -0.0003 | 0.0001 |
| PM2.5 | 53051 | 70 | -0.0012 | 0.0016 | 0.0029 | -0.0012 | -0.0020 | 0.0051 | 0.0000 | 0.0000 |
| PM2.5 | 08041 | 1,766 | -0.0015 | 0.0074 | 0.0089 | -0.0020 | -0.0019 | 0.0174 | -0.0001 | 0.0000 |
| PM2.5 | 09005 | 1,287 | -0.0015 | 0.0183 | 0.0199 | -0.0020 | -0.0019 | 0.0394 | -0.0001 | 0.0000 |
| PM2.5 | 48355 | 2,252 | 0.0011 | 0.0239 | 0.0228 | 0.0016 | -0.0018 | 0.0504 | 0.0000 | 0.0000 |
| PM2.5 | 53007 | 551 | -0.0008 | 0.0631 | 0.0645 | -0.0031 | -0.0017 | 0.1292 | -0.0001 | 0.0001 |
| PM2.5 | 56025 | 421 | -0.0012 | 0.0009 | 0.0021 | -0.0011 | -0.0017 | 0.0035 | 0.0000 | 0.0000 |
| PM2.5 | 45045 | 2,415 | -0.0001 | 0.1199 | 0.1203 | -0.0002 | -0.0013 | 0.2416 | 0.0000 | 0.0000 |
| PM2.5 | 24005 | 4,971 | -0.0004 | 0.0045 | 0.0050 | -0.0005 | -0.0012 | 0.0102 | 0.0000 | 0.0000 |
| PM2.5 | 22015 | 438 | 0.0016 | 0.0309 | 0.0293 | 0.0014 | -0.0011 | 0.0644 | 0.0000 | 0.0000 |
| PM2.5 | 06111 | 2,900 | -0.0012 | 0.0252 | 0.0264 | -0.0014 | -0.0011 | 0.0518 | 0.0000 | 0.0000 |
| PM2.5 | 26005 | 471 | -0.0038 | 0.0186 | 0.0224 | -0.0047 | -0.0009 | 0.0383 | -0.0001 | 0.0000 |
| PM2.5 | 30083 | 92 | -0.0026 | 0.0195 | 0.0221 | -0.0011 | -0.0008 | 0.0409 | -0.0001 | 0.0000 |
| PM2.5 | 40081 | 206 | -0.0018 | 0.0203 | 0.0222 | -0.0021 | -0.0008 | 0.0417 | 0.0000 | 0.0000 |
| PM2.5 | 47037 | 5,736 | 0.0740 | 0.0934 | 0.0194 | 0.0870 | -0.0008 | 0.1875 | -0.0004 | 0.0020 |
| PM2.5 | 42069 | 2,967 | -0.0032 | 0.0914 | 0.0948 | -0.0044 | -0.0007 | 0.1858 | -0.0001 | 0.0001 |
| PM2.5 | 42077 | 3,498 | -0.0005 | 0.0116 | 0.0122 | -0.0005 | -0.0007 | 0.0240 | 0.0000 | 0.0000 |
| PM2.5 | 25003 | 1,562 | 0.0006 | 0.0075 | 0.0069 | 0.0006 | -0.0006 | 0.0160 | 0.0000 | 0.0000 |
| PM2.5 | 42131 | 184 | -0.0004 | 0.0019 | 0.0022 | -0.0004 | -0.0006 | 0.0045 | 0.0000 | 0.0000 |
| PM2.5 | 28059 | 640 | 0.0000 | 0.0461 | 0.0461 | 0.0007 | -0.0005 | 0.0934 | 0.0000 | 0.0000 |
| PM2.5 | 06047 | 828 | -0.0037 | 0.0257 | 0.0295 | -0.0039 | -0.0004 | 0.0517 | -0.0001 | 0.0000 |
| PM2.5 | 34013 | 9,141 | -0.0011 | 0.0193 | 0.0204 | -0.0008 | -0.0003 | 0.0385 | 0.0000 | 0.0000 |
| PM2.5 | 47009 | 612 | -0.0014 | 0.0121 | 0.0136 | -0.0014 | -0.0002 | 0.0250 | 0.0000 | 0.0000 |
| PM2.5 | 15009 | 444 | 0.0003 | 0.0012 | 0.0009 | 0.0001 | -0.0002 | 0.0026 | 0.0000 | 0.0000 |
| PM2.5 | 42085 | 1,304 | -0.0007 | 0.0027 | 0.0034 | -0.0005 | -0.0002 | 0.0056 | 0.0000 | 0.0000 |
| PM2.5 | 41003 | 394 | -0.0032 | 0.0155 | 0.0187 | -0.0030 | -0.0001 | 0.0317 | -0.0001 | 0.0000 |
| PM2.5 | 35049 | 502 | 0.0037 | 0.0039 | 0.0002 | 0.0037 | -0.0001 | 0.0077 | 0.0000 | 0.0001 |
| PM2.5 | 06073 | 14,045 | -0.0016 | 0.0293 | 0.0309 | -0.0019 | 0.0002 | 0.0591 | 0.0000 | 0.0000 |
| PM2.5 | 06079 | 1,309 | -0.0056 | 0.0649 | 0.0706 | -0.0057 | 0.0003 | 0.1298 | -0.0001 | 0.0000 |
| PM2.5 | 53067 | 884 | 0.0109 | 0.1217 | 0.1111 | 0.0100 | 0.0003 | 0.2477 | 0.0000 | 0.0002 |
| PM2.5 | 30111 | 917 | 0.0004 | 0.0130 | 0.0126 | 0.0008 | 0.0004 | 0.0257 | 0.0000 | 0.0000 |
| PM2.5 | 09009 | 7,683 | 0.0002 | 0.0356 | 0.0355 | 0.0002 | 0.0004 | 0.0714 | 0.0000 | 0.0000 |
| PM2.5 | 09001 | 6,596 | 0.0021 | 0.0371 | 0.0351 | 0.0021 | 0.0010 | 0.0742 | 0.0000 | 0.0000 |
| PM2.5 | 41023 | 69 | 0.0040 | 0.0302 | 0.0262 | 0.0031 | 0.0011 | 0.0593 | 0.0000 | 0.0001 |
| PM2.5 | 22071 | 6,661 | -0.0007 | 0.0399 | 0.0407 | -0.0008 | 0.0011 | 0.0804 | 0.0000 | 0.0000 |
| PM2.5 | 15007 | 235 | -0.0016 | 0.0181 | 0.0197 | -0.0020 | 0.0011 | 0.0356 | 0.0000 | 0.0000 |
| PM2.5 | 06083 | 2,356 | 0.0008 | 0.0053 | 0.0045 | 0.0009 | 0.0011 | 0.0096 | 0.0000 | 0.0000 |
| PM2.5 | 48201 | 17,232 | 0.0016 | 0.0273 | 0.0257 | 0.0018 | 0.0012 | 0.0548 | 0.0000 | 0.0000 |
| PM2.5 | 53041 | 492 | 0.0014 | 0.0128 | 0.0115 | 0.0011 | 0.0012 | 0.0245 | 0.0000 | 0.0000 |
| PM2.5 | 04019 | 4,816 | 0.0013 | 0.0033 | 0.0020 | 0.0015 | 0.0013 | 0.0054 | 0.0000 | 0.0000 |
| PM2.5 | 27137 | 2,368 | 0.0008 | 0.0040 | 0.0032 | 0.0010 | 0.0013 | 0.0067 | 0.0000 | 0.0000 |
| PM2.5 | 24025 | 822 | -0.0017 | 0.0283 | 0.0299 | -0.0028 | 0.0015 | 0.0562 | -0.0001 | 0.0000 |
| PM2.5 | 22087 | 405 | -0.0026 | 0.0381 | 0.0407 | -0.0035 | 0.0015 | 0.0763 | -0.0001 | 0.0000 |
| PM2.5 | 30093 | 471 | 0.0043 | 0.0399 | 0.0356 | 0.0047 | 0.0017 | 0.0797 | 0.0000 | 0.0001 |
| PM2.5 | 15001 | 558 | 0.0020 | 0.0050 | 0.0030 | 0.0018 | 0.0017 | 0.0083 | 0.0000 | 0.0000 |
| PM2.5 | 18089 | 4,610 | 0.0008 | 0.0712 | 0.0705 | 0.0012 | 0.0017 | 0.1427 | 0.0000 | 0.0000 |
| PM2.5 | 02110 | 84 | -0.0076 | 0.0937 | 0.1014 | -0.0074 | 0.0018 | 0.1847 | -0.0002 | 0.0000 |
| PM2.5 | 40031 | 691 | -0.0031 | 0.0322 | 0.0352 | -0.0036 | 0.0019 | 0.0635 | -0.0001 | 0.0000 |
| PM2.5 | 38053 | 49 | 0.0115 | 0.0220 | 0.0105 | 0.0121 | 0.0019 | 0.0402 | 0.0000 | 0.0003 |
| PM2.5 | 06061 | 983 | 0.0014 | 0.0074 | 0.0060 | 0.0016 | 0.0021 | 0.0129 | 0.0000 | 0.0000 |
| PM2.5 | 08045 | 142 | 0.0029 | 0.0080 | 0.0051 | 0.0031 | 0.0021 | 0.0138 | 0.0000 | 0.0001 |
| PM2.5 | 38105 | 176 | 0.0027 | 0.0184 | 0.0158 | 0.0013 | 0.0022 | 0.0344 | 0.0000 | 0.0001 |
| PM2.5 | 36119 | 8,283 | 0.0057 | 0.0369 | 0.0313 | 0.0059 | 0.0022 | 0.0724 | 0.0000 | 0.0001 |
| PM2.5 | 16057 | 174 | 0.0035 | 0.0077 | 0.0042 | 0.0032 | 0.0023 | 0.0128 | 0.0000 | 0.0001 |
| PM2.5 | 36001 | 3,671 | 0.0005 | 0.0763 | 0.0760 | -0.0002 | 0.0023 | 0.1509 | 0.0000 | 0.0000 |
| PM2.5 | 36103 | 9,869 | 0.0007 | 0.0210 | 0.0203 | 0.0005 | 0.0023 | 0.0401 | 0.0000 | 0.0000 |
| PM2.5 | 12085 | 872 | 0.0021 | 0.0101 | 0.0080 | 0.0019 | 0.0024 | 0.0180 | 0.0000 | 0.0000 |
| PM2.5 | 48361 | 394 | 0.0020 | 0.0123 | 0.0103 | 0.0012 | 0.0024 | 0.0222 | 0.0000 | 0.0001 |
| PM2.5 | 53053 | 3,726 | -0.0002 | 0.0385 | 0.0387 | 0.0000 | 0.0026 | 0.0753 | 0.0000 | 0.0000 |
| PM2.5 | 47105 | 209 | 0.0052 | 0.0235 | 0.0183 | 0.0060 | 0.0026 | 0.0444 | 0.0000 | 0.0001 |
| PM2.5 | 12011 | 11,336 | 0.0065 | 0.0101 | 0.0036 | 0.0076 | 0.0026 | 0.0177 | 0.0000 | 0.0002 |
| PM2.5 | 01103 | 704 | 0.0039 | 0.0447 | 0.0409 | 0.0041 | 0.0027 | 0.0883 | 0.0000 | 0.0001 |
| PM2.5 | 44003 | 1,275 | 0.0037 | 0.0379 | 0.0342 | 0.0037 | 0.0027 | 0.0734 | 0.0000 | 0.0001 |
| PM2.5 | 25013 | 4,670 | 0.0024 | 0.0184 | 0.0160 | 0.0025 | 0.0027 | 0.0350 | 0.0000 | 0.0000 |
| PM2.5 | 31079 | 531 | 0.0023 | 0.0317 | 0.0295 | 0.0009 | 0.0028 | 0.0626 | 0.0000 | 0.0001 |
| PM2.5 | 56021 | 508 | 0.0049 | 0.0184 | 0.0135 | 0.0058 | 0.0033 | 0.0334 | 0.0000 | 0.0001 |
| PM2.5 | 26091 | 644 | 0.0000 | 0.0318 | 0.0319 | -0.0007 | 0.0034 | 0.0599 | 0.0000 | 0.0000 |
| PM2.5 | 47145 | 366 | 0.0045 | 0.0406 | 0.0362 | 0.0047 | 0.0034 | 0.0792 | 0.0000 | 0.0001 |
| PM2.5 | 53073 | 805 | -0.0007 | 0.0568 | 0.0578 | -0.0012 | 0.0035 | 0.1089 | 0.0000 | 0.0000 |
| PM2.5 | 09003 | 7,067 | -0.0001 | 0.0232 | 0.0233 | 0.0000 | 0.0036 | 0.0436 | 0.0000 | 0.0000 |
| PM2.5 | 12103 | 12,062 | 0.0001 | 0.0432 | 0.0432 | 0.0005 | 0.0038 | 0.0842 | 0.0000 | 0.0000 |
| PM2.5 | 54039 | 2,627 | -0.0002 | 0.0177 | 0.0179 | -0.0003 | 0.0038 | 0.0318 | 0.0000 | 0.0000 |
| PM2.5 | 42015 | 761 | 0.0036 | 0.0090 | 0.0054 | 0.0031 | 0.0040 | 0.0142 | 0.0000 | 0.0001 |
| PM2.5 | 26163 | 22,832 | 0.0036 | 0.0218 | 0.0182 | 0.0041 | 0.0044 | 0.0390 | 0.0000 | 0.0001 |
| PM2.5 | 40109 | 6,168 | -0.0008 | 0.0299 | 0.0308 | -0.0010 | 0.0045 | 0.0550 | 0.0000 | 0.0000 |
| PM2.5 | 00036 | 75,624 | 0.0027 | 0.0561 | 0.0535 | 0.0029 | 0.0046 | 0.1089 | 0.0000 | 0.0001 |
| PM2.5 | 33001 | 404 | 0.0049 | 0.0301 | 0.0252 | 0.0044 | 0.0048 | 0.0560 | 0.0000 | 0.0001 |
| PM2.5 | 27031 | 36 | 0.0057 | 0.0103 | 0.0046 | 0.0061 | 0.0049 | 0.0161 | 0.0000 | 0.0001 |
| PM2.5 | 30049 | 358 | 0.0013 | 0.0311 | 0.0299 | 0.0008 | 0.0053 | 0.0579 | 0.0000 | 0.0000 |
| PM2.5 | 49057 | 989 | 0.1350 | 0.4843 | 0.3502 | 0.1996 | 0.0055 | 0.9794 | -0.0021 | 0.0056 |
| PM2.5 | 48029 | 8,394 | 0.0010 | 0.0217 | 0.0206 | 0.0012 | 0.0056 | 0.0378 | 0.0000 | 0.0000 |
| PM2.5 | 26081 | 3,963 | -0.0001 | 0.0358 | 0.0359 | -0.0004 | 0.0057 | 0.0667 | 0.0000 | 0.0000 |
| PM2.5 | 06081 | 4,202 | 0.0105 | 0.8554 | 0.8508 | 0.0123 | 0.0058 | 1.6595 | 0.0000 | 0.0002 |
| PM2.5 | 55043 | 417 | 0.0016 | 0.0367 | 0.0352 | 0.0011 | 0.0058 | 0.0691 | 0.0000 | 0.0000 |
| PM2.5 | 48013 | 190 | -0.0255 | 0.1410 | 0.1665 | -0.0242 | 0.0061 | 0.2735 | -0.0004 | -0.0001 |
| PM2.5 | 23005 | 2,426 | 0.0043 | 0.0425 | 0.0382 | 0.0049 | 0.0061 | 0.0801 | 0.0000 | 0.0001 |
| PM2.5 | 36065 | 2,609 | -0.0001 | 0.0541 | 0.0543 | 0.0002 | 0.0061 | 0.1037 | 0.0000 | 0.0000 |
| PM2.5 | 26065 | 2,026 | 0.0058 | 0.0342 | 0.0284 | 0.0067 | 0.0062 | 0.0616 | 0.0000 | 0.0001 |
| PM2.5 | 02020 | 678 | 0.0091 | 0.0407 | 0.0316 | 0.0081 | 0.0062 | 0.0757 | 0.0000 | 0.0002 |
| PM2.5 | 39155 | 2,146 | 0.0073 | 0.0684 | 0.0611 | 0.0083 | 0.0064 | 0.1323 | 0.0000 | 0.0002 |
| PM2.5 | 36063 | 2,139 | 0.0011 | 0.0433 | 0.0422 | 0.0010 | 0.0064 | 0.0811 | 0.0000 | 0.0000 |
| PM2.5 | 06085 | 8,294 | 0.0062 | 0.2925 | 0.2878 | 0.0072 | 0.0065 | 0.5731 | 0.0000 | 0.0002 |
| PM2.5 | 17031 | 48,955 | -0.0019 | 0.0629 | 0.0649 | -0.0023 | 0.0067 | 0.1211 | -0.0001 | 0.0000 |
| PM2.5 | 25011 | 576 | 0.0073 | 0.0458 | 0.0385 | 0.0079 | 0.0071 | 0.0851 | 0.0000 | 0.0002 |
| PM2.5 | 55087 | 958 | 0.0021 | 0.0244 | 0.0223 | 0.0019 | 0.0072 | 0.0419 | 0.0000 | 0.0000 |
| PM2.5 | 42049 | 2,767 | 0.0040 | 0.0407 | 0.0367 | 0.0053 | 0.0072 | 0.0742 | 0.0000 | 0.0001 |
| PM2.5 | 53025 | 309 | -0.0012 | 0.0494 | 0.0508 | -0.0015 | 0.0072 | 0.0920 | 0.0000 | 0.0000 |
| PM2.5 | 41063 | 58 | 0.0037 | 0.0566 | 0.0529 | 0.0054 | 0.0076 | 0.1075 | -0.0001 | 0.0001 |
| PM2.5 | 26033 | 252 | 0.0079 | 0.0205 | 0.0126 | 0.0092 | 0.0076 | 0.0336 | -0.0001 | 0.0002 |
| PM2.5 | 06099 | 2,323 | -0.0102 | 0.1569 | 0.1675 | -0.0118 | 0.0079 | 0.3118 | -0.0003 | 0.0000 |
| PM2.5 | 16005 | 435 | 0.0020 | 0.0561 | 0.0542 | 0.0014 | 0.0082 | 0.1050 | 0.0000 | 0.0000 |
| PM2.5 | 36087 | 1,942 | 0.0025 | 0.0473 | 0.0449 | 0.0021 | 0.0082 | 0.0891 | 0.0000 | 0.0000 |
| PM2.5 | 08069 | 785 | 0.0025 | 0.0511 | 0.0486 | 0.0024 | 0.0082 | 0.0953 | 0.0000 | 0.0001 |
| PM2.5 | 48273 | 181 | 0.0012 | 0.0428 | 0.0416 | 0.0007 | 0.0083 | 0.0776 | 0.0000 | 0.0000 |
| PM2.5 | 39049 | 7,742 | 0.0035 | 0.0451 | 0.0416 | 0.0043 | 0.0087 | 0.0839 | 0.0000 | 0.0001 |
| PM2.5 | 13089 | 3,025 | 0.0038 | 0.0425 | 0.0387 | 0.0037 | 0.0088 | 0.0775 | 0.0000 | 0.0001 |
| PM2.5 | 25005 | 4,283 | 0.0000 | 0.1224 | 0.1226 | 0.0003 | 0.0089 | 0.2371 | 0.0000 | 0.0000 |
| PM2.5 | 30063 | 598 | 0.0047 | 0.0309 | 0.0263 | 0.0046 | 0.0089 | 0.0529 | 0.0000 | 0.0001 |
| PM2.5 | 27017 | 264 | -0.0006 | 0.0649 | 0.0655 | -0.0014 | 0.0093 | 0.1179 | -0.0001 | 0.0001 |
| PM2.5 | 46013 | 359 | 0.0058 | 0.0317 | 0.0259 | 0.0056 | 0.0097 | 0.0545 | 0.0000 | 0.0001 |
| PM2.5 | 34023 | 4,168 | 0.0075 | 0.0840 | 0.0768 | 0.0078 | 0.0099 | 0.1599 | 0.0000 | 0.0001 |
| PM2.5 | 34003 | 6,526 | 0.0091 | 0.0968 | 0.0879 | 0.0099 | 0.0099 | 0.1875 | 0.0000 | 0.0002 |
| PM2.5 | 22063 | 271 | 0.0017 | 0.0412 | 0.0395 | 0.0044 | 0.0099 | 0.0719 | -0.0001 | 0.0002 |
| PM2.5 | 36055 | 6,192 | 0.0029 | 0.0674 | 0.0646 | 0.0030 | 0.0102 | 0.1271 | 0.0000 | 0.0001 |
| PM2.5 | 47093 | 3,662 | 0.0015 | 0.0785 | 0.0771 | 0.0011 | 0.0102 | 0.1511 | 0.0000 | 0.0000 |
| PM2.5 | 12099 | 7,017 | 0.0027 | 0.0530 | 0.0503 | 0.0033 | 0.0109 | 0.0963 | 0.0000 | 0.0001 |
| PM2.5 | 25015 | 873 | -0.0002 | 0.0484 | 0.0487 | 0.0001 | 0.0112 | 0.0842 | 0.0000 | 0.0000 |
| PM2.5 | 13051 | 2,129 | 0.0054 | 0.0780 | 0.0727 | 0.0064 | 0.0120 | 0.1474 | 0.0000 | 0.0001 |
| PM2.5 | 06063 | 134 | 0.0052 | 0.2189 | 0.2220 | 0.0009 | 0.0134 | 0.4058 | -0.0002 | 0.0002 |
| PM2.5 | 36059 | 11,536 | 0.0061 | 0.0582 | 0.0522 | 0.0070 | 0.0134 | 0.1043 | 0.0000 | 0.0001 |
| PM2.5 | 06103 | 333 | 0.0004 | 0.0773 | 0.0772 | -0.0007 | 0.0135 | 0.1388 | -0.0001 | 0.0000 |
| PM2.5 | 37021 | 2,176 | -0.0031 | 0.0733 | 0.0765 | -0.0025 | 0.0137 | 0.1341 | 0.0000 | 0.0000 |
| PM2.5 | 23001 | 978 | 0.0044 | 0.0585 | 0.0543 | 0.0040 | 0.0139 | 0.1050 | 0.0000 | 0.0001 |
| PM2.5 | 24510 | 11,455 | 0.0201 | 0.4267 | 0.4080 | 0.0230 | 0.0140 | 0.8562 | -0.0001 | 0.0005 |
| PM2.5 | 19163 | 1,204 | 0.0042 | 0.0684 | 0.0643 | 0.0043 | 0.0142 | 0.1238 | 0.0000 | 0.0001 |
| PM2.5 | 41039 | 1,838 | 0.0058 | 0.1299 | 0.1249 | 0.0052 | 0.0142 | 0.2472 | 0.0000 | 0.0001 |
| PM2.5 | 48245 | 2,590 | 0.0177 | 0.0834 | 0.0657 | 0.0212 | 0.0145 | 0.1489 | -0.0001 | 0.0005 |
| PM2.5 | 53063 | 3,337 | -0.0001 | 0.0720 | 0.0728 | -0.0011 | 0.0147 | 0.1268 | -0.0001 | 0.0000 |
| PM2.5 | 06027 | 199 | 0.0003 | 0.0959 | 0.0955 | 0.0003 | 0.0148 | 0.1743 | 0.0000 | 0.0001 |
| PM2.5 | 19045 | 551 | 0.0000 | 0.0744 | 0.0744 | -0.0005 | 0.0148 | 0.1337 | 0.0000 | 0.0000 |
| PM2.5 | 40143 | 4,549 | 0.0182 | 0.0880 | 0.0699 | 0.0216 | 0.0148 | 0.1584 | -0.0001 | 0.0005 |
| PM2.5 | 32031 | 1,825 | 0.0025 | 0.1150 | 0.1128 | 0.0028 | 0.0151 | 0.2143 | 0.0000 | 0.0001 |
| PM2.5 | 06075 | 8,203 | -0.0015 | 0.1344 | 0.1359 | -0.0014 | 0.0168 | 0.2563 | 0.0000 | 0.0000 |
| PM2.5 | 53077 | 1,512 | -0.0024 | 0.1326 | 0.1384 | -0.0049 | 0.0171 | 0.2450 | -0.0002 | 0.0001 |
| PM2.5 | 12057 | 6,358 | 0.0155 | 0.0496 | 0.0341 | 0.0174 | 0.0182 | 0.0803 | 0.0000 | 0.0004 |
| PM2.5 | 11001 | 8,499 | 0.0021 | 0.1342 | 0.1323 | 0.0021 | 0.0184 | 0.2561 | 0.0000 | 0.0000 |
| PM2.5 | 28047 | 1,485 | 0.0167 | 0.0894 | 0.0728 | 0.0191 | 0.0185 | 0.1592 | 0.0000 | 0.0004 |
| PM2.5 | 06059 | 11,360 | 0.0001 | 0.1206 | 0.1207 | -0.0001 | 0.0200 | 0.2237 | 0.0000 | 0.0000 |
| PM2.5 | 45019 | 2,859 | 0.0029 | 0.0886 | 0.0858 | 0.0027 | 0.0207 | 0.1585 | 0.0000 | 0.0001 |
| PM2.5 | 22033 | 2,710 | 0.0096 | 0.0743 | 0.0647 | 0.0109 | 0.0214 | 0.1265 | 0.0000 | 0.0002 |
| PM2.5 | 36067 | 4,528 | 0.0052 | 0.1213 | 0.1165 | 0.0048 | 0.0223 | 0.2228 | 0.0000 | 0.0001 |
| PM2.5 | 40147 | 453 | 0.0216 | 0.0870 | 0.0654 | 0.0233 | 0.0260 | 0.1489 | 0.0000 | 0.0005 |
| PM2.5 | 06087 | 1,610 | -0.0007 | 0.2817 | 0.2842 | -0.0001 | 0.0281 | 0.5312 | 0.0000 | 0.0000 |
| PM2.5 | 06105 | 85 | 0.0152 | 0.7530 | 0.7397 | 0.0126 | 0.0283 | 1.4757 | 0.0000 | 0.0002 |
| PM2.5 | 41047 | 1,776 | -0.0040 | 0.1281 | 0.1330 | -0.0040 | 0.0288 | 0.2049 | -0.0001 | 0.0000 |
| PM2.5 | 06107 | 1,807 | 0.0610 | 0.3056 | 0.2452 | 0.0697 | 0.0289 | 0.5763 | -0.0002 | 0.0015 |
| PM2.5 | 12033 | 2,160 | 0.0084 | 0.1504 | 0.1423 | 0.0088 | 0.0292 | 0.2765 | 0.0000 | 0.0002 |
| PM2.5 | 53013 | 47 | 0.0385 | 0.1450 | 0.1066 | 0.0453 | 0.0352 | 0.2481 | -0.0001 | 0.0010 |
| PM2.5 | 39151 | 3,598 | 0.0251 | 0.1324 | 0.1074 | 0.0276 | 0.0362 | 0.2331 | 0.0000 | 0.0005 |
| PM2.5 | 06029 | 3,140 | 0.0085 | 0.2683 | 0.2603 | 0.0110 | 0.0375 | 0.5070 | -0.0001 | 0.0003 |
| PM2.5 | 39153 | 4,832 | 0.0171 | 0.1916 | 0.1746 | 0.0189 | 0.0407 | 0.3496 | 0.0000 | 0.0004 |
| PM2.5 | 06021 | 143 | 0.0090 | 0.3798 | 0.3720 | 0.0060 | 0.0423 | 0.7213 | -0.0001 | 0.0002 |
| PM2.5 | 18003 | 2,684 | 0.0346 | 0.1535 | 0.1190 | 0.0368 | 0.0441 | 0.2632 | -0.0001 | 0.0008 |
| PM2.5 | 06007 | 1,452 | 0.0035 | 0.2945 | 0.2927 | 0.0037 | 0.0522 | 0.5435 | 0.0000 | 0.0001 |
| PM2.5 | 30081 | 177 | -0.0034 | 0.2592 | 0.2652 | -0.0021 | 0.0677 | 0.4167 | -0.0001 | 0.0000 |
| PM2.5 | 41067 | 1,415 | 0.0205 | 0.4140 | 0.3989 | 0.0204 | 0.0719 | 0.7022 | 0.0000 | 0.0003 |
| PM2.5 | 49035 | 4,220 | 0.3858 | 1.3561 | 0.9753 | 0.4557 | 0.0833 | 2.6375 | -0.0015 | 0.0100 |
| PM2.5 | 41043 | 627 | -0.0058 | 0.6338 | 0.6423 | -0.0025 | 0.1026 | 1.0437 | -0.0002 | 0.0001 |
| PM2.5 | 20091 | 1,333 | 0.1578 | 0.3308 | 0.1733 | 0.1832 | 0.1230 | 0.5416 | -0.0004 | 0.0040 |
| PM2.5 | 20209 | 1,833 | 0.2761 | 0.7265 | 0.4513 | 0.3251 | 0.2449 | 1.2036 | -0.0011 | 0.0071 |
| O3 | 24027 | 498 | -0.1867 | -0.1572 | 0.0295 | -0.1754 | -0.2566 | -0.0535 | -0.2374 | -0.1125 |
| O3 | 26037 | 171 | -0.0973 | -0.0313 | 0.0658 | -0.1156 | -0.1154 | 0.0536 | -0.1551 | -0.0734 |
| O3 | 09013 | 486 | 0.0242 | 0.2049 | 0.1817 | 0.0409 | -0.0899 | 0.5215 | 0.0126 | 0.0685 |
| O3 | 26133 | 159 | -0.0270 | 0.0344 | 0.0615 | -0.0455 | -0.0809 | 0.1619 | -0.0712 | -0.0185 |
| O3 | 45021 | 283 | -0.0792 | -0.0344 | 0.0448 | -0.0876 | -0.0766 | 0.0091 | -0.1113 | -0.0616 |
| O3 | 39133 | 706 | -0.0295 | 0.0541 | 0.0836 | 0.0066 | -0.0596 | 0.1738 | -0.0456 | 0.0604 |
| O3 | 49003 | 197 | -0.0133 | 0.0183 | 0.0317 | -0.0140 | -0.0534 | 0.0930 | -0.0279 | 0.0001 |
| O3 | 55011 | 78 | -0.0159 | 0.0450 | 0.0607 | -0.0629 | -0.0531 | 0.1479 | -0.1243 | -0.0008 |
| O3 | 30075 | 15 | -0.0952 | 0.0800 | 0.1753 | -0.0946 | -0.0503 | 0.2111 | -0.1375 | -0.0522 |
| O3 | 47155 | 268 | -0.0066 | 0.0507 | 0.0574 | 0.0054 | -0.0483 | 0.1619 | -0.0110 | 0.0221 |
| O3 | 22121 | 60 | -0.0734 | 0.1158 | 0.1891 | -0.1084 | -0.0466 | 0.2866 | -0.1560 | -0.0564 |
| O3 | 06027 | 202 | -0.0257 | 0.0261 | 0.0518 | -0.0373 | -0.0450 | 0.0967 | -0.0531 | -0.0192 |
| O3 | 48339 | 642 | -0.0365 | -0.0057 | 0.0308 | -0.0446 | -0.0424 | 0.0341 | -0.0612 | -0.0272 |
| O3 | 21061 | 32 | -0.0408 | 0.0447 | 0.0857 | -0.0502 | -0.0405 | 0.1372 | -0.0661 | -0.0322 |
| O3 | 37065 | 384 | -0.0103 | 0.0385 | 0.0488 | -0.0175 | -0.0377 | 0.1187 | -0.0273 | -0.0073 |
| O3 | 55037 | 29 | -0.1072 | 0.1771 | 0.2841 | -0.1798 | -0.0376 | 0.3953 | -0.2793 | -0.0747 |
| O3 | 37123 | 180 | -0.0305 | 0.0067 | 0.0373 | -0.0538 | -0.0358 | 0.0501 | -0.0829 | -0.0225 |
| O3 | 04001 | 238 | -0.0177 | 0.1013 | 0.1194 | -0.0374 | -0.0355 | 0.2519 | -0.0623 | -0.0109 |
| O3 | 45003 | 602 | -0.0124 | 0.0129 | 0.0254 | -0.0151 | -0.0344 | 0.0657 | -0.0211 | -0.0090 |
| O3 | 47009 | 649 | 0.0481 | 0.0677 | 0.0197 | 0.0766 | -0.0315 | 0.1676 | 0.0332 | 0.1174 |
| O3 | 13191 | 26 | -0.0377 | 0.0140 | 0.0517 | -0.0487 | -0.0300 | 0.0581 | -0.0646 | -0.0304 |
| O3 | 27071 | 129 | -0.0026 | 0.0506 | 0.0532 | 0.0022 | -0.0289 | 0.1380 | -0.0088 | 0.0124 |
| O3 | 24013 | 620 | -0.0023 | 0.0444 | 0.0467 | -0.0092 | -0.0275 | 0.1239 | -0.0199 | 0.0016 |
| O3 | 51800 | 432 | -0.0202 | 0.0304 | 0.0506 | -0.0156 | -0.0270 | 0.0931 | -0.0287 | -0.0021 |
| O3 | 34029 | 4,593 | 0.0026 | 0.0885 | 0.0859 | 0.0115 | -0.0260 | 0.2193 | -0.0008 | 0.0233 |
| O3 | 21015 | 367 | -0.0436 | 0.0337 | 0.0772 | -0.0346 | -0.0252 | 0.0965 | -0.0535 | -0.0142 |
| O3 | 18167 | 1,338 | 0.0001 | 0.0361 | 0.0360 | 0.0003 | -0.0231 | 0.1034 | 0.0000 | 0.0006 |
| O3 | 26049 | 3,523 | 0.0011 | 0.0846 | 0.0837 | 0.0028 | -0.0139 | 0.1942 | 0.0005 | 0.0050 |
| O3 | 53009 | 491 | -0.0010 | 0.0593 | 0.0604 | 0.0002 | -0.0123 | 0.1377 | -0.0061 | 0.0067 |
| O3 | 28059 | 671 | -0.0199 | 0.0442 | 0.0640 | -0.0174 | -0.0109 | 0.1016 | -0.0249 | -0.0100 |
| O3 | 18173 | 200 | -0.0049 | 0.0138 | 0.0187 | -0.0085 | -0.0094 | 0.0394 | -0.0133 | -0.0034 |
| O3 | 47149 | 650 | -0.0106 | 0.0931 | 0.1038 | -0.0106 | -0.0083 | 0.1992 | -0.0330 | 0.0128 |
| O3 | 54039 | 2,770 | 0.0009 | 0.0570 | 0.0562 | 0.0019 | -0.0081 | 0.1300 | 0.0006 | 0.0031 |
| O3 | 55017 | 427 | -0.0195 | 0.0218 | 0.0412 | -0.0187 | -0.0062 | 0.0490 | -0.0270 | -0.0109 |
| O3 | 49011 | 501 | -0.0322 | 0.1101 | 0.1422 | -0.0463 | -0.0055 | 0.2331 | -0.0651 | -0.0251 |
| O3 | 30023 | 117 | -0.0133 | 0.0270 | 0.0403 | -0.0262 | -0.0042 | 0.0585 | -0.0425 | -0.0085 |
| O3 | 37027 | 369 | -0.0015 | 0.0326 | 0.0340 | 0.0036 | -0.0040 | 0.0737 | -0.0036 | 0.0106 |
| O3 | 25011 | 518 | -0.0036 | 0.0825 | 0.0862 | -0.0073 | -0.0037 | 0.1780 | -0.0130 | -0.0015 |
| O3 | 20133 | 207 | -0.0138 | 0.0502 | 0.0640 | -0.0242 | -0.0034 | 0.1035 | -0.0425 | -0.0057 |
| O3 | 49019 | 50 | 0.0113 | 0.0928 | 0.0815 | -0.0087 | -0.0013 | 0.1870 | -0.0480 | 0.0280 |
| O3 | 06043 | 90 | -0.0392 | 0.1170 | 0.1562 | -0.0506 | -0.0011 | 0.2401 | -0.0709 | -0.0287 |
| O3 | 48497 | 194 | -0.0298 | 0.0947 | 0.1245 | -0.0213 | -0.0011 | 0.1940 | -0.0403 | -0.0030 |
| O3 | 06109 | 319 | 0.0012 | 0.1506 | 0.1494 | 0.0131 | -0.0010 | 0.3170 | -0.0070 | 0.0327 |
| O3 | 51179 | 97 | 0.0090 | 0.0924 | 0.0833 | 0.0182 | -0.0007 | 0.1941 | 0.0059 | 0.0295 |
| O3 | 42125 | 2,180 | -0.0075 | 0.1251 | 0.1326 | -0.0171 | -0.0006 | 0.2594 | -0.0293 | -0.0042 |
| O3 | 37051 | 1,698 | -0.0063 | 0.0662 | 0.0725 | -0.0052 | -0.0004 | 0.1410 | -0.0104 | 0.0002 |
| O3 | 29099 | 617 | -0.0174 | 0.0929 | 0.1103 | -0.0145 | -0.0001 | 0.1925 | -0.0228 | -0.0063 |
| O3 | 54029 | 82 | -0.0249 | 0.0430 | 0.0678 | -0.0223 | 0.0003 | 0.0849 | -0.0326 | -0.0124 |
| O3 | 34015 | 1,488 | 0.0049 | 0.0423 | 0.0373 | 0.0072 | 0.0010 | 0.0878 | 0.0025 | 0.0114 |
| O3 | 51775 | 927 | -0.0073 | 0.0766 | 0.0840 | -0.0051 | 0.0014 | 0.1593 | -0.0103 | 0.0002 |
| O3 | 21185 | 72 | -0.0216 | 0.0964 | 0.1176 | -0.0380 | 0.0015 | 0.1948 | -0.0743 | -0.0010 |
| O3 | 29095 | 7,473 | 0.0107 | 0.0364 | 0.0256 | 0.0217 | 0.0015 | 0.0725 | 0.0066 | 0.0361 |
| O3 | 48085 | 813 | -0.0071 | 0.0307 | 0.0378 | -0.0093 | 0.0015 | 0.0620 | -0.0125 | -0.0056 |
| O3 | 18081 | 520 | -0.0029 | 0.0544 | 0.0574 | -0.0037 | 0.0015 | 0.1116 | -0.0052 | -0.0022 |
| O3 | 20087 | 122 | -0.0927 | 0.2100 | 0.3025 | -0.1223 | 0.0021 | 0.4169 | -0.1647 | -0.0745 |
| O3 | 51131 | 359 | 0.0121 | 0.0860 | 0.0739 | 0.0168 | 0.0027 | 0.1808 | 0.0097 | 0.0228 |
| O3 | 08003 | 91 | -0.0121 | 0.0222 | 0.0343 | -0.0131 | 0.0027 | 0.0419 | -0.0168 | -0.0090 |
| O3 | 37109 | 267 | -0.0283 | 0.1053 | 0.1341 | -0.0314 | 0.0031 | 0.2168 | -0.0410 | -0.0213 |
| O3 | 36045 | 980 | -0.0051 | 0.0455 | 0.0506 | -0.0059 | 0.0031 | 0.0913 | -0.0078 | -0.0039 |
| O3 | 22101 | 373 | -0.0120 | 0.0638 | 0.0757 | -0.0152 | 0.0038 | 0.1267 | -0.0208 | -0.0093 |
| O3 | 01125 | 1,339 | -0.0049 | 0.0751 | 0.0800 | -0.0138 | 0.0040 | 0.1500 | -0.0277 | 0.0003 |
| O3 | 26145 | 1,978 | -0.0027 | 0.0462 | 0.0488 | -0.0033 | 0.0044 | 0.0911 | -0.0047 | -0.0019 |
| O3 | 30093 | 468 | -0.0471 | 0.1547 | 0.2018 | -0.0937 | 0.0049 | 0.3067 | -0.1528 | -0.0301 |
| O3 | 06047 | 837 | -0.0064 | 0.0425 | 0.0489 | -0.0089 | 0.0049 | 0.0795 | -0.0151 | -0.0027 |
| O3 | 21075 | 136 | -0.0341 | 0.0881 | 0.1221 | -0.0549 | 0.0050 | 0.1741 | -0.0818 | -0.0260 |
| O3 | 17019 | 1,296 | -0.0002 | 0.0154 | 0.0156 | 0.0018 | 0.0050 | 0.0272 | -0.0016 | 0.0053 |
| O3 | 18157 | 1,194 | -0.0075 | 0.0465 | 0.0540 | -0.0103 | 0.0052 | 0.0891 | -0.0141 | -0.0059 |
| O3 | 37059 | 140 | 0.0220 | 0.1365 | 0.1148 | 0.0279 | 0.0058 | 0.2851 | 0.0178 | 0.0364 |
| O3 | 26017 | 901 | -0.0099 | 0.0898 | 0.0999 | 0.0129 | 0.0061 | 0.1780 | -0.0196 | 0.0446 |
| O3 | 08123 | 726 | -0.0051 | 0.0362 | 0.0412 | -0.0103 | 0.0072 | 0.0660 | -0.0171 | -0.0031 |
| O3 | 27109 | 1,521 | -0.0167 | 0.0511 | 0.0677 | -0.0171 | 0.0075 | 0.0948 | -0.0222 | -0.0121 |
| O3 | 29123 | 116 | -0.0303 | 0.1029 | 0.1332 | -0.0541 | 0.0080 | 0.2030 | -0.0847 | -0.0225 |
| O3 | 50025 | 357 | -0.0103 | 0.0487 | 0.0591 | -0.0220 | 0.0084 | 0.0904 | -0.0366 | -0.0063 |
| O3 | 55071 | 701 | 0.0275 | 0.0705 | 0.0430 | 0.0275 | 0.0087 | 0.1370 | 0.0188 | 0.0360 |
| O3 | 23027 | 196 | 0.0084 | 0.0624 | 0.0541 | 0.0144 | 0.0087 | 0.1232 | 0.0057 | 0.0226 |
| O3 | 48367 | 290 | -0.0202 | 0.0559 | 0.0761 | -0.0287 | 0.0087 | 0.1065 | -0.0401 | -0.0159 |
| O3 | 55131 | 486 | -0.0027 | 0.0855 | 0.0882 | -0.0038 | 0.0090 | 0.1677 | -0.0056 | -0.0020 |
| O3 | 45037 | 110 | 0.0084 | 0.0983 | 0.0899 | 0.0165 | 0.0097 | 0.1976 | 0.0038 | 0.0288 |
| O3 | 45011 | 133 | 0.0044 | 0.0524 | 0.0480 | -0.0039 | 0.0097 | 0.0982 | -0.0156 | 0.0075 |
| O3 | 24045 | 1,236 | -0.0113 | 0.0843 | 0.0957 | -0.0159 | 0.0098 | 0.1622 | -0.0226 | -0.0086 |
| O3 | 23007 | 217 | -0.0042 | 0.0395 | 0.0437 | 0.0040 | 0.0104 | 0.0709 | -0.0072 | 0.0154 |
| O3 | 45077 | 437 | -0.0106 | 0.0926 | 0.1032 | -0.0137 | 0.0107 | 0.1782 | -0.0185 | -0.0084 |
| O3 | 55141 | 1,010 | -0.0293 | 0.1010 | 0.1302 | -0.0425 | 0.0108 | 0.1919 | -0.0615 | -0.0220 |
| O3 | 26027 | 252 | -0.0175 | 0.1022 | 0.1199 | -0.0162 | 0.0116 | 0.1919 | -0.0253 | -0.0075 |
| O3 | 47187 | 365 | -0.0736 | 0.1428 | 0.2164 | -0.0741 | 0.0118 | 0.2650 | -0.1066 | -0.0430 |
| O3 | 22017 | 3,513 | 0.0037 | 0.0790 | 0.0753 | 0.0040 | 0.0119 | 0.1519 | 0.0028 | 0.0052 |
| O3 | 36113 | 867 | 0.0233 | 0.2040 | 0.1811 | -0.0186 | 0.0120 | 0.4211 | -0.0778 | 0.0379 |
| O3 | 18169 | 282 | -0.0164 | 0.0784 | 0.0949 | -0.0316 | 0.0124 | 0.1497 | -0.0506 | -0.0111 |
| O3 | 44007 | 7,027 | 0.0103 | 0.0244 | 0.0141 | 0.0061 | 0.0126 | 0.0367 | -0.0006 | 0.0128 |
| O3 | 55089 | 304 | 0.0081 | 0.1410 | 0.1330 | 0.0092 | 0.0131 | 0.2787 | 0.0055 | 0.0129 |
| O3 | 28033 | 128 | -0.0064 | 0.0531 | 0.0595 | -0.0111 | 0.0132 | 0.0933 | -0.0172 | -0.0046 |
| O3 | 06051 | 43 | 0.0142 | 0.1413 | 0.1271 | 0.0161 | 0.0135 | 0.2803 | 0.0072 | 0.0253 |
| O3 | 55117 | 909 | 0.0126 | 0.0979 | 0.0854 | 0.0212 | 0.0135 | 0.1919 | 0.0089 | 0.0325 |
| O3 | 21191 | 34 | 0.0121 | 0.1064 | 0.0943 | -0.0236 | 0.0135 | 0.2025 | -0.0721 | 0.0243 |
| O3 | 37129 | 1,211 | 0.0021 | 0.1154 | 0.1134 | 0.0097 | 0.0136 | 0.2279 | -0.0006 | 0.0197 |
| O3 | 06007 | 1,657 | -0.0053 | 0.0602 | 0.0655 | -0.0068 | 0.0136 | 0.1080 | -0.0094 | -0.0039 |
| O3 | 55009 | 1,555 | -0.0031 | 0.0540 | 0.0571 | 0.0005 | 0.0137 | 0.0951 | -0.0046 | 0.0054 |
| O3 | 48409 | 306 | -0.0005 | 0.0906 | 0.0912 | -0.0007 | 0.0137 | 0.1731 | -0.0018 | 0.0003 |
| O3 | 25015 | 863 | -0.0080 | 0.0860 | 0.0940 | -0.0129 | 0.0138 | 0.1615 | -0.0194 | -0.0060 |
| O3 | 13247 | 265 | -0.0050 | 0.1103 | 0.1153 | -0.0242 | 0.0143 | 0.2134 | -0.0507 | 0.0031 |
| O3 | 48375 | 1,704 | -0.0175 | 0.1403 | 0.1578 | -0.0262 | 0.0145 | 0.2731 | -0.0386 | -0.0129 |
| O3 | 39027 | 325 | -0.0098 | 0.0526 | 0.0624 | -0.0130 | 0.0158 | 0.0903 | -0.0177 | -0.0078 |
| O3 | 32005 | 51 | 0.1105 | 0.1538 | 0.0433 | 0.1278 | 0.0159 | 0.2890 | 0.0876 | 0.1634 |
| O3 | 50003 | 396 | -0.0055 | 0.0421 | 0.0476 | -0.0064 | 0.0163 | 0.0682 | -0.0090 | -0.0037 |
| O3 | 13089 | 3,150 | -0.0028 | 0.0850 | 0.0878 | -0.0058 | 0.0164 | 0.1568 | -0.0097 | -0.0018 |
| O3 | 04005 | 437 | -0.0290 | 0.1007 | 0.1297 | -0.0442 | 0.0166 | 0.1865 | -0.0657 | -0.0218 |
| O3 | 36029 | 10,810 | -0.0053 | 0.0556 | 0.0609 | -0.0103 | 0.0170 | 0.0944 | -0.0166 | -0.0034 |
| O3 | 23019 | 1,266 | 0.0148 | 0.0693 | 0.0545 | 0.0147 | 0.0170 | 0.1277 | 0.0098 | 0.0196 |
| O3 | 53063 | 3,475 | -0.0051 | 0.0763 | 0.0815 | -0.0091 | 0.0172 | 0.1359 | -0.0144 | -0.0036 |
| O3 | 06055 | 1,122 | 0.0040 | 0.0463 | 0.0424 | 0.0036 | 0.0172 | 0.0755 | 0.0020 | 0.0051 |
| O3 | 21221 | 81 | -0.0093 | 0.1215 | 0.1310 | -0.0036 | 0.0173 | 0.2261 | -0.0126 | 0.0051 |
| O3 | 28049 | 3,687 | -0.0033 | 0.0807 | 0.0840 | 0.0023 | 0.0173 | 0.1466 | -0.0058 | 0.0105 |
| O3 | 12117 | 1,397 | -0.0022 | 0.0837 | 0.0860 | -0.0070 | 0.0175 | 0.1546 | -0.0136 | -0.0003 |
| O3 | 37117 | 203 | 0.0068 | 0.0908 | 0.0843 | 0.0103 | 0.0175 | 0.1719 | 0.0027 | 0.0177 |
| O3 | 25017 | 11,352 | -0.0082 | 0.1156 | 0.1237 | -0.0144 | 0.0176 | 0.2173 | -0.0231 | -0.0054 |
| O3 | 48135 | 848 | -0.0092 | 0.1294 | 0.1386 | -0.0108 | 0.0179 | 0.2486 | -0.0146 | -0.0069 |
| O3 | 31041 | 165 | -0.0054 | 0.0579 | 0.0634 | -0.0074 | 0.0182 | 0.0964 | -0.0103 | -0.0042 |
| O3 | 36027 | 2,161 | -0.0080 | 0.0585 | 0.0665 | -0.0125 | 0.0185 | 0.0997 | -0.0187 | -0.0059 |
| O3 | 18127 | 756 | -0.0125 | 0.0956 | 0.1081 | -0.0141 | 0.0186 | 0.1755 | -0.0184 | -0.0097 |
| O3 | 06001 | 9,673 | -0.0029 | 0.0599 | 0.0628 | -0.0077 | 0.0186 | 0.1014 | -0.0140 | -0.0012 |
| O3 | 12031 | 5,501 | -0.0047 | 0.0756 | 0.0804 | -0.0080 | 0.0186 | 0.1344 | -0.0125 | -0.0033 |
| O3 | 12103 | 12,642 | -0.0005 | 0.0647 | 0.0652 | -0.0020 | 0.0187 | 0.1120 | -0.0040 | 0.0000 |
| O3 | 45045 | 2,572 | -0.0039 | 0.0925 | 0.0964 | -0.0094 | 0.0188 | 0.1692 | -0.0164 | -0.0020 |
| O3 | 39103 | 713 | 0.0422 | 0.0495 | 0.0073 | 0.0305 | 0.0188 | 0.0838 | 0.0083 | 0.0530 |
| O3 | 51085 | 151 | 0.0296 | 0.0721 | 0.0425 | 0.0376 | 0.0189 | 0.1289 | 0.0233 | 0.0505 |
| O3 | 12073 | 1,386 | -0.0094 | 0.0907 | 0.1002 | -0.0127 | 0.0191 | 0.1638 | -0.0176 | -0.0073 |
| O3 | 49049 | 1,112 | -0.0055 | 0.0751 | 0.0806 | -0.0077 | 0.0192 | 0.1343 | -0.0114 | -0.0037 |
| O3 | 37025 | 829 | -0.0090 | 0.1454 | 0.1544 | -0.0110 | 0.0197 | 0.2787 | -0.0151 | -0.0067 |
| O3 | 29077 | 2,699 | -0.0022 | 0.0977 | 0.0998 | -0.0018 | 0.0203 | 0.1799 | -0.0035 | -0.0002 |
| O3 | 17091 | 913 | 0.0063 | 0.1223 | 0.1161 | 0.0042 | 0.0203 | 0.2325 | -0.0023 | 0.0102 |
| O3 | 45013 | 432 | -0.0036 | 0.0564 | 0.0600 | 0.0180 | 0.0206 | 0.0941 | -0.0109 | 0.0478 |
| O3 | 21227 | 805 | 0.0198 | 0.0565 | 0.0366 | 0.0391 | 0.0207 | 0.0937 | 0.0132 | 0.0630 |
| O3 | 45087 | 256 | 0.0138 | 0.1029 | 0.0892 | 0.0141 | 0.0207 | 0.1885 | 0.0103 | 0.0179 |
| O3 | 42003 | 17,725 | -0.0034 | 0.0721 | 0.0756 | -0.0072 | 0.0208 | 0.1248 | -0.0121 | -0.0020 |
| O3 | 25023 | 3,073 | 0.0025 | 0.0480 | 0.0456 | 0.0007 | 0.0209 | 0.0757 | -0.0020 | 0.0033 |
| O3 | 23015 | 217 | -0.0001 | 0.0587 | 0.0587 | 0.0029 | 0.0211 | 0.0975 | -0.0012 | 0.0069 |
| O3 | 06075 | 8,203 | 0.0065 | 0.1594 | 0.1531 | 0.0063 | 0.0212 | 0.3091 | 0.0044 | 0.0080 |
| O3 | 04025 | 874 | 0.0031 | 0.1002 | 0.0970 | 0.0119 | 0.0216 | 0.1891 | -0.0019 | 0.0254 |
| O3 | 42099 | 138 | -0.0232 | 0.1248 | 0.1480 | -0.0410 | 0.0217 | 0.2296 | -0.0642 | -0.0168 |
| O3 | 48109 | 24 | 0.0039 | 0.0601 | 0.0561 | 0.0239 | 0.0218 | 0.0991 | -0.0024 | 0.0489 |
| O3 | 06073 | 15,314 | -0.0054 | 0.1136 | 0.1190 | -0.0127 | 0.0219 | 0.2094 | -0.0224 | -0.0026 |
| O3 | 12071 | 2,863 | -0.0028 | 0.0897 | 0.0925 | -0.0067 | 0.0222 | 0.1603 | -0.0117 | -0.0015 |
| O3 | 12057 | 6,887 | -0.0046 | 0.0843 | 0.0889 | -0.0099 | 0.0224 | 0.1474 | -0.0169 | -0.0025 |
| O3 | 23011 | 1,373 | -0.0027 | 0.1182 | 0.1210 | -0.0057 | 0.0225 | 0.2202 | -0.0097 | -0.0016 |
| O3 | 37077 | 289 | -0.0047 | 0.0746 | 0.0793 | -0.0126 | 0.0229 | 0.1285 | -0.0227 | -0.0020 |
| O3 | 06053 | 2,043 | -0.0045 | 0.0719 | 0.0765 | -0.0093 | 0.0230 | 0.1217 | -0.0157 | -0.0025 |
| O3 | 12099 | 7,915 | -0.0019 | 0.0791 | 0.0810 | -0.0023 | 0.0231 | 0.1358 | -0.0031 | -0.0014 |
| O3 | 48457 | 144 | -0.0086 | 0.1018 | 0.1104 | -0.0037 | 0.0232 | 0.1821 | -0.0118 | 0.0041 |
| O3 | 39007 | 840 | -0.0043 | 0.1341 | 0.1385 | 0.0018 | 0.0232 | 0.2520 | -0.0066 | 0.0100 |
| O3 | 12033 | 2,278 | -0.0015 | 0.0913 | 0.0929 | -0.0058 | 0.0234 | 0.1628 | -0.0114 | -0.0001 |
| O3 | 45051 | 914 | -0.0054 | 0.1042 | 0.1097 | -0.0080 | 0.0236 | 0.1875 | -0.0117 | -0.0041 |
| O3 | 06079 | 1,432 | -0.0003 | 0.0681 | 0.0684 | -0.0033 | 0.0238 | 0.1121 | -0.0075 | 0.0008 |
| O3 | 53061 | 2,206 | -0.0022 | 0.0805 | 0.0827 | -0.0033 | 0.0239 | 0.1363 | -0.0049 | -0.0017 |
| O3 | 53011 | 1,334 | -0.0300 | 0.1456 | 0.1755 | -0.0479 | 0.0239 | 0.2737 | -0.0740 | -0.0209 |
| O3 | 39025 | 521 | -0.0005 | 0.0649 | 0.0654 | -0.0041 | 0.0239 | 0.1057 | -0.0095 | 0.0013 |
| O3 | 45019 | 2,995 | -0.0071 | 0.1339 | 0.1412 | -0.0022 | 0.0245 | 0.2489 | -0.0095 | 0.0047 |
| O3 | 48361 | 378 | 0.0530 | 0.0616 | 0.0087 | 0.0664 | 0.0247 | 0.0991 | 0.0427 | 0.0871 |
| O3 | 54069 | 1,119 | -0.0079 | 0.0960 | 0.1040 | -0.0112 | 0.0248 | 0.1671 | -0.0157 | -0.0062 |
| O3 | 48291 | 382 | -0.0121 | 0.1179 | 0.1301 | -0.0221 | 0.0249 | 0.2175 | -0.0389 | -0.0052 |
| O3 | 13223 | 143 | 0.0007 | 0.1088 | 0.1081 | 0.0059 | 0.0253 | 0.1961 | -0.0040 | 0.0158 |
| O3 | 34007 | 4,290 | -0.0041 | 0.1047 | 0.1090 | -0.0074 | 0.0253 | 0.1845 | -0.0118 | -0.0028 |
| O3 | 48439 | 7,778 | 0.0016 | 0.1229 | 0.1214 | -0.0005 | 0.0255 | 0.2247 | -0.0036 | 0.0024 |
| O3 | 33011 | 2,519 | -0.0050 | 0.0860 | 0.0910 | -0.0096 | 0.0259 | 0.1450 | -0.0160 | -0.0031 |
| O3 | 23029 | 310 | 0.0138 | 0.0577 | 0.0439 | 0.0092 | 0.0259 | 0.0890 | -0.0007 | 0.0190 |
| O3 | 26081 | 4,076 | 0.0047 | 0.1158 | 0.1112 | 0.0060 | 0.0266 | 0.2089 | 0.0037 | 0.0079 |
| O3 | 39023 | 1,400 | -0.0012 | 0.0910 | 0.0923 | -0.0044 | 0.0266 | 0.1544 | -0.0086 | -0.0002 |
| O3 | 51161 | 341 | 0.0041 | 0.0927 | 0.0886 | -0.0031 | 0.0268 | 0.1594 | -0.0137 | 0.0072 |
| O3 | 01099 | 184 | -0.0074 | 0.1262 | 0.1333 | -0.0132 | 0.0276 | 0.2270 | -0.0272 | 0.0009 |
| O3 | 21043 | 82 | -0.0102 | 0.1235 | 0.1336 | -0.0032 | 0.0277 | 0.2234 | -0.0139 | 0.0076 |
| O3 | 50027 | 530 | -0.0082 | 0.1435 | 0.1517 | -0.0091 | 0.0278 | 0.2678 | -0.0128 | -0.0055 |
| O3 | 25013 | 4,690 | -0.0095 | 0.1081 | 0.1176 | -0.0188 | 0.0278 | 0.1885 | -0.0312 | -0.0057 |
| O3 | 23003 | 699 | 0.0096 | 0.0836 | 0.0739 | 0.0115 | 0.0279 | 0.1429 | 0.0067 | 0.0162 |
| O3 | 06083 | 2,533 | -0.0028 | 0.0883 | 0.0912 | -0.0081 | 0.0282 | 0.1474 | -0.0149 | -0.0011 |
| O3 | 04019 | 5,235 | -0.0044 | 0.0865 | 0.0909 | -0.0110 | 0.0284 | 0.1453 | -0.0196 | -0.0020 |
| O3 | 34003 | 6,435 | -0.0019 | 0.1232 | 0.1251 | -0.0049 | 0.0289 | 0.2179 | -0.0089 | -0.0009 |
| O3 | 51191 | 382 | -0.0054 | 0.1602 | 0.1657 | 0.0010 | 0.0290 | 0.3084 | -0.0082 | 0.0101 |
| O3 | 26147 | 1,117 | -0.0030 | 0.1036 | 0.1066 | -0.0030 | 0.0291 | 0.1780 | -0.0041 | -0.0019 |
| O3 | 06045 | 669 | 0.0059 | 0.0676 | 0.0617 | 0.0072 | 0.0292 | 0.1065 | 0.0047 | 0.0094 |
| O3 | 48201 | 18,185 | -0.0041 | 0.0987 | 0.1028 | -0.0087 | 0.0294 | 0.1688 | -0.0150 | -0.0022 |
| O3 | 45031 | 479 | -0.0180 | 0.1425 | 0.1606 | -0.0310 | 0.0294 | 0.2603 | -0.0482 | -0.0128 |
| O3 | 32510 | 336 | 0.0079 | 0.1049 | 0.0970 | 0.0116 | 0.0295 | 0.1823 | 0.0053 | 0.0174 |
| O3 | 06089 | 1,160 | -0.0067 | 0.0992 | 0.1059 | -0.0126 | 0.0296 | 0.1699 | -0.0203 | -0.0044 |
| O3 | 55039 | 809 | 0.0114 | 0.1232 | 0.1119 | 0.0177 | 0.0301 | 0.2245 | 0.0088 | 0.0256 |
| O3 | 44003 | 1,281 | 0.0271 | 0.0449 | 0.0178 | 0.0350 | 0.0301 | 0.0602 | 0.0211 | 0.0472 |
| O3 | 06081 | 4,479 | -0.0045 | 0.0990 | 0.1035 | -0.0099 | 0.0304 | 0.1683 | -0.0170 | -0.0024 |
| O3 | 29021 | 1,126 | -0.0122 | 0.1626 | 0.1748 | 0.0050 | 0.0304 | 0.3024 | -0.0184 | 0.0286 |
| O3 | 55101 | 1,339 | 0.0062 | 0.1132 | 0.1071 | 0.0133 | 0.0305 | 0.2008 | 0.0036 | 0.0223 |
| O3 | 36083 | 1,324 | 0.0001 | 0.0942 | 0.0941 | -0.0062 | 0.0306 | 0.1568 | -0.0147 | 0.0020 |
| O3 | 06017 | 569 | -0.0059 | 0.0813 | 0.0872 | -0.0079 | 0.0306 | 0.1326 | -0.0108 | -0.0048 |
| O3 | 28073 | 294 | 0.0160 | 0.0969 | 0.0809 | 0.0247 | 0.0310 | 0.1695 | 0.0116 | 0.0368 |
| O3 | 17115 | 1,334 | -0.0127 | 0.1125 | 0.1253 | -0.0205 | 0.0310 | 0.1952 | -0.0313 | -0.0092 |
| O3 | 31055 | 4,478 | 0.0002 | 0.1052 | 0.1050 | -0.0009 | 0.0311 | 0.1773 | -0.0026 | 0.0006 |
| O3 | 37101 | 463 | 0.0089 | 0.1488 | 0.1400 | 0.0142 | 0.0314 | 0.2758 | 0.0069 | 0.0206 |
| O3 | 25027 | 6,371 | -0.0027 | 0.1125 | 0.1152 | -0.0049 | 0.0314 | 0.1910 | -0.0081 | -0.0018 |
| O3 | 48233 | 181 | -0.0187 | 0.1719 | 0.1907 | -0.0235 | 0.0316 | 0.3145 | -0.0316 | -0.0143 |
| O3 | 21199 | 550 | 0.0005 | 0.1033 | 0.1028 | 0.0123 | 0.0322 | 0.1775 | -0.0102 | 0.0350 |
| O3 | 04027 | 794 | -0.0121 | 0.1134 | 0.1255 | -0.0267 | 0.0324 | 0.1960 | -0.0455 | -0.0066 |
| O3 | 12095 | 4,888 | 0.0046 | 0.0874 | 0.0828 | 0.0054 | 0.0325 | 0.1419 | 0.0036 | 0.0068 |
| O3 | 01097 | 3,381 | -0.0066 | 0.1173 | 0.1239 | -0.0118 | 0.0326 | 0.2008 | -0.0186 | -0.0046 |
| O3 | 06087 | 1,666 | -0.0016 | 0.0954 | 0.0970 | -0.0068 | 0.0327 | 0.1585 | -0.0136 | 0.0001 |
| O3 | 06095 | 1,669 | -0.0009 | 0.1013 | 0.1022 | -0.0085 | 0.0327 | 0.1705 | -0.0188 | 0.0020 |
| O3 | 22095 | 199 | 0.0098 | 0.1433 | 0.1336 | -0.0015 | 0.0329 | 0.2580 | -0.0180 | 0.0141 |
| O3 | 17031 | 48,007 | -0.0028 | 0.0891 | 0.0919 | -0.0077 | 0.0330 | 0.1467 | -0.0141 | -0.0011 |
| O3 | 55033 | 196 | -0.0133 | 0.1227 | 0.1362 | -0.0202 | 0.0332 | 0.2101 | -0.0294 | -0.0100 |
| O3 | 21139 | 82 | 0.0030 | 0.1170 | 0.1139 | 0.0122 | 0.0334 | 0.1937 | -0.0175 | 0.0426 |
| O3 | 34011 | 1,339 | -0.0025 | 0.1656 | 0.1682 | 0.0070 | 0.0334 | 0.3017 | -0.0061 | 0.0202 |
| O3 | 55105 | 1,136 | -0.0011 | 0.1073 | 0.1083 | 0.0027 | 0.0334 | 0.1789 | -0.0024 | 0.0077 |
| O3 | 25009 | 6,054 | -0.0014 | 0.1210 | 0.1224 | 0.0013 | 0.0335 | 0.2110 | -0.0024 | 0.0050 |
| O3 | 37183 | 2,400 | 0.0019 | 0.1037 | 0.1019 | -0.0065 | 0.0335 | 0.1746 | -0.0178 | 0.0046 |
| O3 | 21183 | 189 | -0.0088 | 0.1289 | 0.1376 | -0.0074 | 0.0335 | 0.2233 | -0.0114 | -0.0036 |
| O3 | 36059 | 11,619 | -0.0028 | 0.1142 | 0.1171 | -0.0078 | 0.0336 | 0.1956 | -0.0142 | -0.0011 |
| O3 | 36015 | 1,046 | -0.0058 | 0.0908 | 0.0966 | -0.0108 | 0.0340 | 0.1488 | -0.0173 | -0.0039 |
| O3 | 41047 | 1,911 | -0.0022 | 0.0896 | 0.0918 | -0.0062 | 0.0342 | 0.1466 | -0.0114 | -0.0008 |
| O3 | 37145 | 202 | -0.0013 | 0.1149 | 0.1162 | -0.0029 | 0.0343 | 0.1953 | -0.0051 | -0.0008 |
| O3 | 51087 | 1,932 | 0.0050 | 0.1722 | 0.1673 | 0.0107 | 0.0344 | 0.3159 | 0.0032 | 0.0174 |
| O3 | 28089 | 213 | -0.0039 | 0.1058 | 0.1097 | -0.0163 | 0.0349 | 0.1784 | -0.0329 | 0.0004 |
| O3 | 19193 | 1,298 | -0.0012 | 0.0924 | 0.0937 | -0.0039 | 0.0351 | 0.1498 | -0.0076 | -0.0003 |
| O3 | 26063 | 294 | 0.0049 | 0.1129 | 0.1080 | -0.0053 | 0.0351 | 0.1945 | -0.0202 | 0.0087 |
| O3 | 42085 | 1,331 | -0.0035 | 0.0864 | 0.0900 | -0.0052 | 0.0352 | 0.1386 | -0.0077 | -0.0026 |
| O3 | 22071 | 6,966 | -0.0047 | 0.1370 | 0.1417 | -0.0094 | 0.0353 | 0.2386 | -0.0155 | -0.0030 |
| O3 | 37147 | 1,081 | -0.0052 | 0.1324 | 0.1376 | -0.0030 | 0.0357 | 0.2260 | -0.0067 | 0.0008 |
| O3 | 25021 | 4,730 | 0.0045 | 0.1515 | 0.1471 | 0.0084 | 0.0358 | 0.2729 | 0.0032 | 0.0128 |
| O3 | 34023 | 4,273 | -0.0111 | 0.1110 | 0.1222 | -0.0147 | 0.0361 | 0.1859 | -0.0201 | -0.0087 |
| O3 | 21169 | 31 | -0.0307 | 0.1285 | 0.1593 | -0.0406 | 0.0363 | 0.2269 | -0.0554 | -0.0244 |
| O3 | 36007 | 2,241 | -0.0046 | 0.0993 | 0.1038 | -0.0153 | 0.0363 | 0.1649 | -0.0288 | -0.0011 |
| O3 | 12081 | 2,360 | 0.0116 | 0.0755 | 0.0639 | 0.0210 | 0.0364 | 0.1151 | 0.0078 | 0.0331 |
| O3 | 22047 | 225 | -0.0014 | 0.1679 | 0.1694 | -0.0073 | 0.0365 | 0.3029 | -0.0164 | 0.0017 |
| O3 | 29047 | 1,090 | 0.0019 | 0.1062 | 0.1043 | 0.0055 | 0.0367 | 0.1749 | 0.0007 | 0.0100 |
| O3 | 51059 | 2,104 | -0.0041 | 0.0967 | 0.1008 | -0.0092 | 0.0369 | 0.1583 | -0.0161 | -0.0020 |
| O3 | 26121 | 1,382 | 0.0034 | 0.1279 | 0.1245 | 0.0043 | 0.0370 | 0.2163 | 0.0027 | 0.0054 |
| O3 | 01043 | 540 | -0.0082 | 0.1221 | 0.1303 | -0.0127 | 0.0374 | 0.2044 | -0.0189 | -0.0060 |
| O3 | 47145 | 355 | -0.0035 | 0.1196 | 0.1231 | -0.0114 | 0.0374 | 0.2002 | -0.0218 | -0.0010 |
| O3 | 06013 | 5,067 | -0.0045 | 0.1105 | 0.1150 | -0.0117 | 0.0375 | 0.1850 | -0.0209 | -0.0019 |
| O3 | 15003 | 4,586 | -0.0173 | 0.2011 | 0.2185 | -0.0155 | 0.0378 | 0.3723 | -0.0219 | -0.0093 |
| O3 | 23005 | 2,560 | -0.0034 | 0.1424 | 0.1458 | -0.0072 | 0.0378 | 0.2512 | -0.0122 | -0.0020 |
| O3 | 04011 | 39 | 0.0130 | 0.0685 | 0.0555 | 0.0236 | 0.0381 | 0.1006 | 0.0073 | 0.0391 |
| O3 | 22015 | 432 | 0.0089 | 0.1349 | 0.1260 | 0.0007 | 0.0381 | 0.2365 | -0.0112 | 0.0125 |
| O3 | 55087 | 1,021 | -0.0013 | 0.0995 | 0.1008 | -0.0026 | 0.0382 | 0.1597 | -0.0051 | 0.0000 |
| O3 | 42017 | 3,635 | 0.0018 | 0.1229 | 0.1212 | -0.0023 | 0.0383 | 0.2100 | -0.0082 | 0.0033 |
| O3 | 26161 | 2,765 | 0.0042 | 0.1005 | 0.0964 | 0.0124 | 0.0383 | 0.1675 | 0.0015 | 0.0228 |
| O3 | 01055 | 1,073 | 0.0033 | 0.1344 | 0.1311 | 0.0041 | 0.0385 | 0.2298 | 0.0023 | 0.0057 |
| O3 | 17049 | 372 | 0.0068 | 0.2106 | 0.2043 | 0.0166 | 0.0385 | 0.3932 | 0.0028 | 0.0294 |
| O3 | 36055 | 6,303 | 0.0008 | 0.1063 | 0.1055 | -0.0021 | 0.0386 | 0.1743 | -0.0061 | 0.0018 |
| O3 | 35013 | 534 | -0.0084 | 0.1105 | 0.1189 | -0.0193 | 0.0389 | 0.1843 | -0.0330 | -0.0045 |
| O3 | 39003 | 1,236 | -0.0245 | 0.1516 | 0.1760 | -0.0307 | 0.0390 | 0.2622 | -0.0408 | -0.0189 |
| O3 | 10001 | 713 | 0.0056 | 0.1969 | 0.1917 | 0.0112 | 0.0392 | 0.3643 | 0.0039 | 0.0175 |
| O3 | 40027 | 674 | 0.0030 | 0.1313 | 0.1285 | -0.0011 | 0.0393 | 0.2213 | -0.0071 | 0.0046 |
| O3 | 04013 | 14,615 | -0.0075 | 0.1159 | 0.1234 | -0.0176 | 0.0394 | 0.1939 | -0.0306 | -0.0038 |
| O3 | 00036 | 74,752 | -0.0038 | 0.1247 | 0.1286 | -0.0100 | 0.0396 | 0.2104 | -0.0180 | -0.0018 |
| O3 | 22011 | 235 | -0.0297 | 0.1506 | 0.1803 | -0.0342 | 0.0402 | 0.2608 | -0.0454 | -0.0224 |
| O3 | 55073 | 778 | 0.0080 | 0.1056 | 0.0976 | 0.0102 | 0.0402 | 0.1677 | 0.0063 | 0.0133 |
| O3 | 51015 | 148 | -0.0437 | 0.2112 | 0.2549 | -0.0513 | 0.0403 | 0.3859 | -0.0664 | -0.0346 |
| O3 | 17097 | 3,059 | 0.0058 | 0.1058 | 0.1000 | 0.0076 | 0.0403 | 0.1698 | 0.0038 | 0.0113 |
| O3 | 47011 | 502 | 0.0299 | 0.1100 | 0.0802 | 0.0398 | 0.0404 | 0.1839 | 0.0226 | 0.0546 |
| O3 | 21117 | 1,178 | 0.0096 | 0.1047 | 0.0951 | 0.0083 | 0.0405 | 0.1691 | 0.0025 | 0.0140 |
| O3 | 28045 | 193 | -0.0091 | 0.2410 | 0.2506 | -0.0228 | 0.0407 | 0.4455 | -0.0477 | 0.0039 |
| O3 | 01073 | 8,403 | -0.0040 | 0.1524 | 0.1565 | -0.0052 | 0.0409 | 0.2634 | -0.0075 | -0.0029 |
| O3 | 26065 | 2,118 | 0.0038 | 0.1300 | 0.1263 | 0.0053 | 0.0411 | 0.2179 | 0.0030 | 0.0072 |
| O3 | 48355 | 2,312 | -0.0066 | 0.1510 | 0.1577 | -0.0100 | 0.0417 | 0.2645 | -0.0149 | -0.0048 |
| O3 | 33013 | 959 | -0.0050 | 0.1533 | 0.1582 | -0.0044 | 0.0417 | 0.2662 | -0.0084 | -0.0005 |
| O3 | 36067 | 4,697 | 0.0053 | 0.1219 | 0.1167 | 0.0048 | 0.0417 | 0.2009 | 0.0026 | 0.0068 |
| O3 | 21059 | 870 | 0.0003 | 0.1502 | 0.1499 | 0.0004 | 0.0422 | 0.2603 | 0.0000 | 0.0006 |
| O3 | 49057 | 1,129 | -0.0012 | 0.1131 | 0.1143 | -0.0020 | 0.0422 | 0.1808 | -0.0057 | 0.0015 |
| O3 | 05119 | 4,710 | 0.0043 | 0.1086 | 0.1043 | 0.0114 | 0.0428 | 0.1712 | 0.0020 | 0.0200 |
| O3 | 55083 | 241 | 0.0148 | 0.1020 | 0.0872 | 0.0253 | 0.0430 | 0.1606 | 0.0106 | 0.0385 |
| O3 | 36063 | 2,106 | -0.0001 | 0.1262 | 0.1265 | 0.0044 | 0.0431 | 0.2084 | -0.0015 | 0.0100 |
| O3 | 55079 | 9,836 | -0.0004 | 0.1494 | 0.1499 | -0.0005 | 0.0433 | 0.2531 | -0.0011 | 0.0000 |
| O3 | 29189 | 8,461 | -0.0050 | 0.1340 | 0.1390 | -0.0132 | 0.0434 | 0.2239 | -0.0239 | -0.0021 |
| O3 | 27013 | 475 | -0.0192 | 0.1613 | 0.1806 | -0.0361 | 0.0435 | 0.2819 | -0.0578 | -0.0127 |
| O3 | 21021 | 272 | 0.0071 | 0.1010 | 0.0939 | 0.0049 | 0.0436 | 0.1591 | 0.0005 | 0.0088 |
| O3 | 47165 | 523 | -0.0213 | 0.1895 | 0.2110 | -0.0189 | 0.0440 | 0.3347 | -0.0266 | -0.0112 |
| O3 | 45029 | 303 | -0.0083 | 0.1712 | 0.1797 | -0.0087 | 0.0440 | 0.2977 | -0.0120 | -0.0056 |
| O3 | 18003 | 2,826 | -0.0017 | 0.1171 | 0.1188 | -0.0062 | 0.0442 | 0.1915 | -0.0123 | -0.0002 |
| O3 | 51061 | 294 | 0.0012 | 0.2158 | 0.2148 | 0.0051 | 0.0443 | 0.4007 | -0.0006 | 0.0105 |
| O3 | 26163 | 22,672 | 0.0043 | 0.1248 | 0.1206 | 0.0027 | 0.0444 | 0.2039 | -0.0002 | 0.0053 |
| O3 | 28001 | 462 | -0.0154 | 0.2199 | 0.2356 | -0.0072 | 0.0446 | 0.4020 | -0.0203 | 0.0061 |
| O3 | 06025 | 697 | 0.0016 | 0.1235 | 0.1219 | -0.0007 | 0.0447 | 0.2020 | -0.0041 | 0.0024 |
| O3 | 08013 | 924 | -0.0042 | 0.1126 | 0.1169 | -0.0082 | 0.0448 | 0.1822 | -0.0134 | -0.0027 |
| O3 | 17111 | 890 | -0.0094 | 0.1575 | 0.1670 | -0.0159 | 0.0449 | 0.2690 | -0.0251 | -0.0065 |
| O3 | 48113 | 12,904 | -0.0027 | 0.1212 | 0.1238 | -0.0068 | 0.0450 | 0.1987 | -0.0123 | -0.0011 |
| O3 | 26099 | 3,780 | 0.0082 | 0.1408 | 0.1326 | 0.0113 | 0.0450 | 0.2364 | 0.0066 | 0.0151 |
| O3 | 26125 | 8,172 | 0.0051 | 0.1271 | 0.1220 | 0.0058 | 0.0452 | 0.2063 | 0.0040 | 0.0073 |
| O3 | 04003 | 557 | -0.0099 | 0.1178 | 0.1277 | -0.0076 | 0.0453 | 0.1899 | -0.0137 | -0.0013 |
| O3 | 55061 | 129 | 0.0097 | 0.1410 | 0.1313 | -0.0081 | 0.0455 | 0.2389 | -0.0371 | 0.0195 |
| O3 | 23013 | 402 | -0.0126 | 0.1407 | 0.1532 | -0.0188 | 0.0455 | 0.2341 | -0.0275 | -0.0096 |
| O3 | 47055 | 206 | -0.0028 | 0.1574 | 0.1602 | -0.0027 | 0.0458 | 0.2642 | -0.0055 | -0.0001 |
| O3 | 27163 | 374 | 0.0005 | 0.1154 | 0.1149 | -0.0013 | 0.0459 | 0.1836 | -0.0042 | 0.0015 |
| O3 | 47041 | 139 | 0.0614 | 0.0999 | 0.0386 | 0.0612 | 0.0460 | 0.1518 | 0.0402 | 0.0826 |
| O3 | 21051 | 162 | -0.0193 | 0.1611 | 0.1804 | -0.0317 | 0.0462 | 0.2777 | -0.0493 | -0.0135 |
| O3 | 30013 | 718 | -0.0053 | 0.1460 | 0.1513 | -0.0021 | 0.0463 | 0.2432 | -0.0071 | 0.0029 |
| O3 | 39017 | 2,159 | -0.0054 | 0.1293 | 0.1347 | -0.0101 | 0.0469 | 0.2138 | -0.0168 | -0.0034 |
| O3 | 08005 | 1,301 | -0.0152 | 0.1377 | 0.1529 | -0.0283 | 0.0469 | 0.2318 | -0.0449 | -0.0102 |
| O3 | 17001 | 932 | -0.0028 | 0.1561 | 0.1589 | -0.0045 | 0.0470 | 0.2626 | -0.0077 | -0.0014 |
| O3 | 55025 | 2,801 | -0.0011 | 0.1315 | 0.1326 | -0.0042 | 0.0470 | 0.2152 | -0.0083 | -0.0001 |
| O3 | 36091 | 693 | -0.0130 | 0.1673 | 0.1805 | -0.0225 | 0.0472 | 0.2862 | -0.0350 | -0.0093 |
| O3 | 01117 | 352 | -0.0113 | 0.1504 | 0.1617 | -0.0125 | 0.0473 | 0.2515 | -0.0169 | -0.0081 |
| O3 | 13215 | 1,760 | -0.0029 | 0.1416 | 0.1445 | -0.0086 | 0.0473 | 0.2360 | -0.0160 | -0.0009 |
| O3 | 06085 | 8,601 | -0.0023 | 0.1535 | 0.1557 | -0.0083 | 0.0479 | 0.2574 | -0.0166 | -0.0002 |
| O3 | 17033 | 186 | -0.0217 | 0.1596 | 0.1814 | -0.0297 | 0.0481 | 0.2699 | -0.0424 | -0.0157 |
| O3 | 22073 | 1,403 | 0.0075 | 0.1511 | 0.1436 | 0.0097 | 0.0481 | 0.2528 | 0.0061 | 0.0127 |
| O3 | 19153 | 3,379 | -0.0123 | 0.1342 | 0.1465 | -0.0131 | 0.0485 | 0.2210 | -0.0170 | -0.0090 |
| O3 | 08069 | 945 | -0.0013 | 0.1161 | 0.1174 | -0.0050 | 0.0487 | 0.1836 | -0.0099 | -0.0001 |
| O3 | 39135 | 131 | -0.0135 | 0.1694 | 0.1829 | -0.0221 | 0.0488 | 0.2914 | -0.0336 | -0.0098 |
| O3 | 47037 | 6,140 | -0.0025 | 0.1603 | 0.1627 | -0.0056 | 0.0489 | 0.2689 | -0.0101 | -0.0012 |
| O3 | 09005 | 1,259 | 0.0138 | 0.3009 | 0.2879 | 0.0253 | 0.0489 | 0.5692 | 0.0100 | 0.0388 |
| O3 | 48061 | 1,574 | -0.0177 | 0.1834 | 0.2011 | -0.0334 | 0.0491 | 0.3198 | -0.0534 | -0.0118 |
| O3 | 41005 | 1,831 | -0.0048 | 0.1352 | 0.1401 | -0.0094 | 0.0491 | 0.2223 | -0.0153 | -0.0031 |
| O3 | 37081 | 3,009 | -0.0009 | 0.1446 | 0.1456 | -0.0050 | 0.0494 | 0.2405 | -0.0106 | 0.0005 |
| O3 | 47189 | 375 | 0.0005 | 0.1304 | 0.1301 | -0.0016 | 0.0495 | 0.2149 | -0.0065 | 0.0034 |
| O3 | 48245 | 2,658 | -0.0071 | 0.1707 | 0.1779 | -0.0141 | 0.0495 | 0.2937 | -0.0232 | -0.0046 |
| O3 | 18019 | 673 | -0.0044 | 0.1457 | 0.1502 | -0.0095 | 0.0498 | 0.2420 | -0.0164 | -0.0023 |
| O3 | 49047 | 89 | -0.0075 | 0.1372 | 0.1448 | -0.0178 | 0.0502 | 0.2275 | -0.0321 | -0.0031 |
| O3 | 41039 | 2,079 | -0.0015 | 0.1439 | 0.1455 | -0.0056 | 0.0502 | 0.2385 | -0.0112 | -0.0001 |
| O3 | 27075 | 88 | -0.0110 | 0.1536 | 0.1647 | -0.0145 | 0.0502 | 0.2566 | -0.0208 | -0.0076 |
| O3 | 22033 | 3,058 | -0.0059 | 0.1503 | 0.1562 | -0.0075 | 0.0503 | 0.2487 | -0.0102 | -0.0045 |
| O3 | 45079 | 3,220 | 0.0006 | 0.1431 | 0.1425 | 0.0009 | 0.0506 | 0.2342 | 0.0002 | 0.0016 |
| O3 | 18163 | 2,433 | -0.0026 | 0.1485 | 0.1511 | -0.0086 | 0.0507 | 0.2442 | -0.0169 | -0.0002 |
| O3 | 29510 | 7,827 | -0.0034 | 0.1461 | 0.1496 | -0.0077 | 0.0507 | 0.2400 | -0.0133 | -0.0019 |
| O3 | 28149 | 525 | 0.0000 | 0.1599 | 0.1598 | 0.0214 | 0.0514 | 0.2722 | -0.0068 | 0.0490 |
| O3 | 42021 | 1,905 | -0.0045 | 0.1566 | 0.1612 | -0.0108 | 0.0517 | 0.2609 | -0.0192 | -0.0021 |
| O3 | 33015 | 1,069 | -0.0016 | 0.1259 | 0.1275 | -0.0059 | 0.0518 | 0.2010 | -0.0115 | -0.0003 |
| O3 | 49035 | 4,394 | -0.0003 | 0.1456 | 0.1459 | -0.0041 | 0.0520 | 0.2385 | -0.0096 | 0.0012 |
| O3 | 21071 | 407 | -0.0024 | 0.1506 | 0.1530 | -0.0073 | 0.0520 | 0.2493 | -0.0138 | -0.0009 |
| O3 | 01001 | 148 | 0.0545 | 0.1470 | 0.0925 | 0.0441 | 0.0521 | 0.2457 | 0.0185 | 0.0678 |
| O3 | 55109 | 351 | -0.0011 | 0.1567 | 0.1579 | -0.0073 | 0.0523 | 0.2614 | -0.0159 | 0.0013 |
| O3 | 55043 | 408 | -0.0047 | 0.1752 | 0.1800 | -0.0086 | 0.0523 | 0.2946 | -0.0141 | -0.0034 |
| O3 | 47157 | 8,928 | -0.0024 | 0.1493 | 0.1518 | -0.0058 | 0.0528 | 0.2454 | -0.0103 | -0.0013 |
| O3 | 48141 | 3,095 | -0.0084 | 0.1634 | 0.1719 | -0.0143 | 0.0528 | 0.2706 | -0.0227 | -0.0057 |
| O3 | 36065 | 2,683 | -0.0028 | 0.1530 | 0.1560 | -0.0082 | 0.0529 | 0.2534 | -0.0151 | -0.0011 |
| O3 | 06059 | 12,628 | -0.0063 | 0.1960 | 0.2023 | -0.0150 | 0.0530 | 0.3418 | -0.0266 | -0.0030 |
| O3 | 09007 | 1,119 | 0.0466 | 0.2645 | 0.2184 | 0.0444 | 0.0532 | 0.4848 | 0.0282 | 0.0613 |
| O3 | 42129 | 3,504 | -0.0030 | 0.1500 | 0.1530 | -0.0116 | 0.0536 | 0.2495 | -0.0232 | 0.0003 |
| O3 | 45063 | 736 | -0.0091 | 0.1853 | 0.1944 | -0.0224 | 0.0544 | 0.3162 | -0.0400 | -0.0041 |
| O3 | 47065 | 3,045 | -0.0027 | 0.1654 | 0.1681 | -0.0110 | 0.0545 | 0.2763 | -0.0215 | 0.0000 |
| O3 | 36117 | 551 | 0.0128 | 0.1451 | 0.1325 | 0.0106 | 0.0545 | 0.2343 | 0.0055 | 0.0153 |
| O3 | 21037 | 858 | -0.0093 | 0.1956 | 0.2048 | -0.0126 | 0.0547 | 0.3320 | -0.0177 | -0.0072 |
| O3 | 42043 | 3,039 | -0.0031 | 0.1516 | 0.1548 | -0.0117 | 0.0548 | 0.2494 | -0.0235 | 0.0001 |
| O3 | 36031 | 281 | 0.0009 | 0.1331 | 0.1323 | -0.0021 | 0.0549 | 0.2101 | -0.0068 | 0.0026 |
| O3 | 17163 | 2,591 | -0.0079 | 0.1575 | 0.1655 | -0.0180 | 0.0549 | 0.2620 | -0.0312 | -0.0041 |
| O3 | 22093 | 104 | 0.0104 | 0.1248 | 0.1145 | 0.0063 | 0.0550 | 0.1948 | -0.0006 | 0.0129 |
| O3 | 17117 | 365 | 0.0048 | 0.1783 | 0.1736 | 0.0094 | 0.0551 | 0.3029 | 0.0009 | 0.0175 |
| O3 | 55127 | 543 | 0.0209 | 0.1876 | 0.1669 | 0.0329 | 0.0554 | 0.3265 | 0.0160 | 0.0480 |
| O3 | 22005 | 263 | 0.0209 | 0.1514 | 0.1305 | 0.0311 | 0.0556 | 0.2534 | 0.0160 | 0.0439 |
| O3 | 27123 | 4,276 | -0.0011 | 0.1315 | 0.1327 | -0.0030 | 0.0556 | 0.2089 | -0.0057 | -0.0004 |
| O3 | 55021 | 403 | -0.0030 | 0.1499 | 0.1528 | -0.0102 | 0.0556 | 0.2464 | -0.0197 | -0.0006 |
| O3 | 09001 | 6,596 | 0.0002 | 0.1494 | 0.1493 | -0.0017 | 0.0560 | 0.2440 | -0.0045 | 0.0009 |
| O3 | 51113 | 55 | -0.0452 | 0.2557 | 0.3011 | -0.0533 | 0.0562 | 0.4516 | -0.0725 | -0.0322 |
| O3 | 54025 | 372 | -0.0038 | 0.1496 | 0.1535 | -0.0038 | 0.0563 | 0.2428 | -0.0052 | -0.0025 |
| O3 | 47119 | 522 | -0.0114 | 0.1817 | 0.1932 | -0.0222 | 0.0564 | 0.3049 | -0.0359 | -0.0075 |
| O3 | 55063 | 1,201 | -0.0073 | 0.1476 | 0.1547 | -0.0133 | 0.0565 | 0.2407 | -0.0210 | -0.0050 |
| O3 | 20173 | 3,551 | -0.0002 | 0.1505 | 0.1508 | -0.0052 | 0.0568 | 0.2436 | -0.0120 | 0.0015 |
| O3 | 36001 | 3,723 | 0.0230 | 0.1708 | 0.1480 | 0.0383 | 0.0568 | 0.2926 | 0.0163 | 0.0587 |
| O3 | 29037 | 230 | -0.0122 | 0.1758 | 0.1881 | -0.0211 | 0.0572 | 0.2940 | -0.0335 | -0.0082 |
| O3 | 22019 | 1,464 | -0.0125 | 0.1776 | 0.1901 | -0.0211 | 0.0572 | 0.3009 | -0.0330 | -0.0086 |
| O3 | 48183 | 1,057 | 0.0004 | 0.1890 | 0.1887 | -0.0041 | 0.0574 | 0.3247 | -0.0110 | 0.0025 |
| O3 | 02020 | 832 | 0.0035 | 0.1498 | 0.1463 | 0.0073 | 0.0576 | 0.2407 | 0.0022 | 0.0119 |
| O3 | 36093 | 1,763 | -0.0002 | 0.1828 | 0.1832 | -0.0057 | 0.0576 | 0.3127 | -0.0134 | 0.0017 |
| O3 | 24001 | 1,104 | 0.0116 | 0.1497 | 0.1381 | 0.0155 | 0.0584 | 0.2391 | 0.0094 | 0.0205 |
| O3 | 06041 | 1,675 | -0.0037 | 0.1807 | 0.1845 | -0.0087 | 0.0584 | 0.3028 | -0.0161 | -0.0015 |
| O3 | 28075 | 1,096 | 0.0098 | 0.1121 | 0.1022 | 0.0129 | 0.0584 | 0.1682 | 0.0074 | 0.0178 |
| O3 | 42073 | 1,080 | -0.0311 | 0.2132 | 0.2445 | -0.0506 | 0.0586 | 0.3719 | -0.0775 | -0.0224 |
| O3 | 37037 | 228 | 0.0364 | 0.2209 | 0.1845 | 0.0233 | 0.0587 | 0.3798 | -0.0062 | 0.0511 |
| O3 | 23031 | 1,006 | -0.0013 | 0.1784 | 0.1798 | -0.0036 | 0.0589 | 0.2970 | -0.0082 | 0.0012 |
| O3 | 10003 | 3,577 | -0.0036 | 0.1616 | 0.1652 | -0.0075 | 0.0591 | 0.2636 | -0.0127 | -0.0022 |
| O3 | 17099 | 1,079 | 0.0020 | 0.1608 | 0.1590 | -0.0028 | 0.0593 | 0.2609 | -0.0098 | 0.0037 |
| O3 | 42077 | 3,613 | -0.0103 | 0.1813 | 0.1917 | -0.0196 | 0.0593 | 0.3067 | -0.0318 | -0.0066 |
| O3 | 22089 | 154 | -0.0014 | 0.1774 | 0.1787 | -0.0127 | 0.0595 | 0.2967 | -0.0289 | 0.0035 |
| O3 | 27037 | 717 | -0.0112 | 0.2025 | 0.2138 | -0.0307 | 0.0598 | 0.3464 | -0.0556 | -0.0044 |
| O3 | 42011 | 3,039 | 0.0189 | 0.1907 | 0.1719 | 0.0239 | 0.0600 | 0.3259 | 0.0154 | 0.0310 |
| O3 | 32031 | 2,023 | 0.0007 | 0.1448 | 0.1441 | -0.0003 | 0.0601 | 0.2290 | -0.0020 | 0.0012 |
| O3 | 54011 | 1,764 | 0.0031 | 0.1609 | 0.1577 | 0.0021 | 0.0602 | 0.2616 | -0.0001 | 0.0039 |
| O3 | 17161 | 1,542 | -0.0060 | 0.1693 | 0.1754 | -0.0150 | 0.0606 | 0.2829 | -0.0266 | -0.0027 |
| O3 | 23017 | 452 | -0.0059 | 0.1781 | 0.1840 | -0.0042 | 0.0607 | 0.2968 | -0.0076 | -0.0010 |
| O3 | 06107 | 1,938 | -0.0041 | 0.1633 | 0.1674 | -0.0114 | 0.0608 | 0.2668 | -0.0210 | -0.0015 |
| O3 | 32003 | 4,145 | 0.0042 | 0.1295 | 0.1254 | 0.0090 | 0.0609 | 0.1999 | 0.0024 | 0.0147 |
| O3 | 21049 | 234 | 0.0036 | 0.1481 | 0.1445 | 0.0089 | 0.0613 | 0.2332 | 0.0019 | 0.0152 |
| O3 | 18089 | 4,734 | -0.0033 | 0.1682 | 0.1717 | -0.0115 | 0.0613 | 0.2792 | -0.0226 | -0.0002 |
| O3 | 22055 | 1,461 | 0.0095 | 0.1684 | 0.1590 | 0.0185 | 0.0614 | 0.2788 | 0.0064 | 0.0294 |
| O3 | 39073 | 208 | -0.0096 | 0.1900 | 0.1996 | -0.0189 | 0.0618 | 0.3202 | -0.0317 | -0.0058 |
| O3 | 17167 | 2,267 | -0.0011 | 0.1606 | 0.1617 | -0.0043 | 0.0622 | 0.2581 | -0.0086 | 0.0000 |
| O3 | 34005 | 2,695 | 0.0010 | 0.1415 | 0.1406 | 0.0016 | 0.0623 | 0.2218 | 0.0007 | 0.0023 |
| O3 | 21035 | 299 | 0.0106 | 0.1788 | 0.1681 | 0.0085 | 0.0623 | 0.2962 | 0.0038 | 0.0127 |
| O3 | 53053 | 4,009 | -0.0066 | 0.1746 | 0.1812 | -0.0156 | 0.0626 | 0.2891 | -0.0273 | -0.0033 |
| O3 | 42095 | 1,361 | -0.0044 | 0.1849 | 0.1893 | -0.0133 | 0.0628 | 0.3062 | -0.0252 | -0.0009 |
| O3 | 22043 | 72 | 0.0682 | 0.1675 | 0.0995 | 0.0779 | 0.0634 | 0.2864 | 0.0521 | 0.1031 |
| O3 | 18083 | 592 | -0.0059 | 0.1960 | 0.2020 | -0.0149 | 0.0634 | 0.3292 | -0.0274 | -0.0022 |
| O3 | 36119 | 8,523 | -0.0036 | 0.1910 | 0.1946 | -0.0098 | 0.0634 | 0.3184 | -0.0181 | -0.0013 |
| O3 | 18141 | 2,514 | -0.0038 | 0.1776 | 0.1814 | -0.0065 | 0.0634 | 0.2914 | -0.0104 | -0.0026 |
| O3 | 42091 | 6,911 | -0.0077 | 0.1911 | 0.1989 | -0.0167 | 0.0635 | 0.3205 | -0.0285 | -0.0042 |
| O3 | 48397 | 54 | 0.0290 | 0.1322 | 0.1033 | 0.0426 | 0.0635 | 0.1981 | 0.0196 | 0.0642 |
| O3 | 55031 | 351 | 0.0073 | 0.1383 | 0.1311 | 0.0058 | 0.0637 | 0.2154 | 0.0017 | 0.0097 |
| O3 | 19163 | 1,209 | -0.0135 | 0.1962 | 0.2097 | -0.0276 | 0.0638 | 0.3290 | -0.0461 | -0.0081 |
| O3 | 41029 | 1,206 | -0.0023 | 0.1753 | 0.1776 | -0.0053 | 0.0639 | 0.2862 | -0.0094 | -0.0013 |
| O3 | 01103 | 720 | 0.0029 | 0.1650 | 0.1621 | 0.0033 | 0.0641 | 0.2623 | -0.0001 | 0.0064 |
| O3 | 37159 | 1,094 | -0.0002 | 0.1956 | 0.1961 | -0.0071 | 0.0642 | 0.3291 | -0.0166 | 0.0022 |
| O3 | 06111 | 3,190 | -0.0077 | 0.1709 | 0.1787 | -0.0182 | 0.0642 | 0.2798 | -0.0316 | -0.0039 |
| O3 | 42081 | 1,261 | 0.0049 | 0.1799 | 0.1751 | -0.0008 | 0.0644 | 0.2952 | -0.0098 | 0.0076 |
| O3 | 23009 | 434 | -0.0128 | 0.1906 | 0.2035 | -0.0056 | 0.0645 | 0.3132 | -0.0175 | 0.0065 |
| O3 | 17119 | 2,110 | -0.0055 | 0.1720 | 0.1776 | -0.0122 | 0.0647 | 0.2815 | -0.0208 | -0.0031 |
| O3 | 37103 | 19 | 0.0734 | 0.1227 | 0.0494 | 0.0699 | 0.0651 | 0.1824 | 0.0466 | 0.0931 |
| O3 | 17201 | 2,306 | 0.0010 | 0.1673 | 0.1664 | -0.0015 | 0.0653 | 0.2677 | -0.0053 | 0.0019 |
| O3 | 42079 | 4,318 | 0.0079 | 0.1530 | 0.1451 | 0.0175 | 0.0654 | 0.2394 | 0.0046 | 0.0291 |
| O3 | 39099 | 2,874 | -0.0011 | 0.1738 | 0.1749 | -0.0064 | 0.0655 | 0.2836 | -0.0136 | 0.0009 |
| O3 | 41013 | 104 | -0.0166 | 0.1960 | 0.2127 | -0.0190 | 0.0656 | 0.3244 | -0.0246 | -0.0129 |
| O3 | 01089 | 1,794 | -0.0011 | 0.1956 | 0.1968 | 0.0058 | 0.0665 | 0.3215 | -0.0035 | 0.0150 |
| O3 | 45091 | 730 | -0.0109 | 0.1998 | 0.2107 | -0.0168 | 0.0665 | 0.3305 | -0.0251 | -0.0082 |
| O3 | 34035 | 1,377 | -0.0157 | 0.2252 | 0.2411 | -0.0346 | 0.0670 | 0.3896 | -0.0587 | -0.0086 |
| O3 | 12001 | 2,203 | -0.0029 | 0.1778 | 0.1806 | -0.0045 | 0.0674 | 0.2881 | -0.0076 | -0.0017 |
| O3 | 39143 | 473 | 0.0176 | 0.1530 | 0.1353 | 0.0404 | 0.0676 | 0.2439 | 0.0066 | 0.0725 |
| O3 | 47163 | 1,690 | -0.0028 | 0.1868 | 0.1897 | -0.0047 | 0.0689 | 0.3024 | -0.0076 | -0.0020 |
| O3 | 48453 | 3,061 | -0.0026 | 0.2087 | 0.2113 | -0.0095 | 0.0693 | 0.3500 | -0.0191 | -0.0002 |
| O3 | 21019 | 521 | -0.0351 | 0.2141 | 0.2491 | -0.0415 | 0.0697 | 0.3608 | -0.0540 | -0.0272 |
| O3 | 26103 | 594 | 0.0081 | 0.2118 | 0.2039 | 0.0096 | 0.0707 | 0.3534 | 0.0040 | 0.0149 |
| O3 | 17197 | 1,803 | -0.0085 | 0.1967 | 0.2053 | -0.0143 | 0.0710 | 0.3232 | -0.0222 | -0.0061 |
| O3 | 42045 | 5,548 | -0.0052 | 0.2055 | 0.2107 | -0.0155 | 0.0714 | 0.3422 | -0.0289 | -0.0016 |
| O3 | 51041 | 469 | -0.0097 | 0.1915 | 0.2010 | -0.0256 | 0.0714 | 0.3146 | -0.0460 | -0.0040 |
| O3 | 21093 | 548 | -0.0042 | 0.2196 | 0.2238 | -0.0009 | 0.0715 | 0.3669 | -0.0063 | 0.0045 |
| O3 | 39061 | 9,557 | -0.0009 | 0.1987 | 0.1997 | -0.0067 | 0.0716 | 0.3276 | -0.0148 | 0.0014 |
| O3 | 55099 | 159 | 0.0095 | 0.1865 | 0.1772 | 0.0159 | 0.0719 | 0.3000 | 0.0074 | 0.0236 |
| O3 | 48167 | 1,884 | -0.0037 | 0.2222 | 0.2260 | -0.0067 | 0.0720 | 0.3692 | -0.0120 | -0.0017 |
| O3 | 23001 | 1,024 | 0.0042 | 0.1972 | 0.1930 | 0.0046 | 0.0723 | 0.3241 | 0.0015 | 0.0075 |
| O3 | 39083 | 445 | 0.0252 | 0.2171 | 0.1920 | 0.0525 | 0.0726 | 0.3635 | 0.0122 | 0.0907 |
| O3 | 48121 | 791 | -0.0092 | 0.2125 | 0.2219 | -0.0211 | 0.0727 | 0.3580 | -0.0362 | -0.0050 |
| O3 | 37157 | 601 | -0.0024 | 0.1750 | 0.1774 | -0.0062 | 0.0731 | 0.2776 | -0.0118 | -0.0008 |
| O3 | 17133 | 97 | -0.0073 | 0.2024 | 0.2098 | -0.0119 | 0.0734 | 0.3397 | -0.0190 | -0.0047 |
| O3 | 37067 | 3,141 | -0.0011 | 0.1890 | 0.1903 | -0.0029 | 0.0737 | 0.3072 | -0.0056 | -0.0004 |
| O3 | 06077 | 3,194 | -0.0016 | 0.1885 | 0.1903 | -0.0047 | 0.0739 | 0.3028 | -0.0089 | -0.0006 |
| O3 | 39035 | 16,678 | 0.0003 | 0.2053 | 0.2051 | -0.0040 | 0.0740 | 0.3358 | -0.0103 | 0.0020 |
| O3 | 37119 | 3,935 | -0.0090 | 0.1956 | 0.2047 | -0.0164 | 0.0741 | 0.3193 | -0.0262 | -0.0061 |
| O3 | 51810 | 1,103 | 0.0143 | 0.2306 | 0.2164 | 0.0259 | 0.0742 | 0.3853 | 0.0102 | 0.0398 |
| O3 | 34001 | 2,729 | -0.0092 | 0.1945 | 0.2036 | -0.0216 | 0.0742 | 0.3153 | -0.0372 | -0.0051 |
| O3 | 17089 | 2,471 | -0.0051 | 0.1949 | 0.2001 | -0.0124 | 0.0744 | 0.3175 | -0.0226 | -0.0022 |
| O3 | 08001 | 1,146 | -0.0266 | 0.2635 | 0.2901 | -0.0411 | 0.0747 | 0.4527 | -0.0611 | -0.0198 |
| O3 | 42019 | 1,131 | -0.0049 | 0.2017 | 0.2067 | -0.0102 | 0.0752 | 0.3283 | -0.0174 | -0.0029 |
| O3 | 48071 | 98 | 0.0169 | 0.1693 | 0.1524 | 0.0191 | 0.0754 | 0.2617 | 0.0131 | 0.0250 |
| O3 | 17199 | 772 | 0.0042 | 0.1839 | 0.1798 | 0.0028 | 0.0756 | 0.2900 | 0.0002 | 0.0052 |
| O3 | 55059 | 941 | 0.0023 | 0.1680 | 0.1658 | 0.0071 | 0.0761 | 0.2631 | 0.0007 | 0.0130 |
| O3 | 09009 | 8,135 | 0.0067 | 0.2542 | 0.2479 | 0.0115 | 0.0762 | 0.4313 | 0.0048 | 0.0173 |
| O3 | 45023 | 224 | -0.0103 | 0.2362 | 0.2467 | -0.0258 | 0.0762 | 0.3973 | -0.0451 | -0.0055 |
| O3 | 48029 | 9,023 | -0.0060 | 0.2008 | 0.2068 | -0.0149 | 0.0763 | 0.3290 | -0.0266 | -0.0027 |
| O3 | 48039 | 749 | -0.0123 | 0.2179 | 0.2303 | -0.0183 | 0.0764 | 0.3591 | -0.0266 | -0.0093 |
| O3 | 42069 | 2,983 | -0.0041 | 0.2249 | 0.2291 | -0.0132 | 0.0765 | 0.3734 | -0.0254 | -0.0009 |
| O3 | 51550 | 636 | -0.0033 | 0.2310 | 0.2343 | -0.0055 | 0.0769 | 0.3814 | -0.0097 | -0.0014 |
| O3 | 39113 | 5,765 | 0.0002 | 0.2108 | 0.2107 | -0.0039 | 0.0770 | 0.3463 | -0.0100 | 0.0019 |
| O3 | 53033 | 10,769 | -0.0065 | 0.2214 | 0.2280 | -0.0125 | 0.0773 | 0.3686 | -0.0203 | -0.0043 |
| O3 | 25003 | 1,585 | -0.0014 | 0.2445 | 0.2461 | 0.0027 | 0.0784 | 0.4031 | -0.0029 | 0.0081 |
| O3 | 27003 | 861 | -0.0016 | 0.1983 | 0.2000 | -0.0067 | 0.0785 | 0.3186 | -0.0139 | 0.0003 |
| O3 | 06039 | 414 | 0.0096 | 0.1668 | 0.1573 | 0.0227 | 0.0791 | 0.2562 | 0.0056 | 0.0386 |
| O3 | 06097 | 2,845 | 0.0045 | 0.1874 | 0.1830 | 0.0024 | 0.0795 | 0.2938 | -0.0014 | 0.0058 |
| O3 | 19121 | 106 | -0.0044 | 0.2209 | 0.2254 | -0.0137 | 0.0797 | 0.3590 | -0.0254 | -0.0015 |
| O3 | 24017 | 341 | -0.0050 | 0.2242 | 0.2293 | -0.0113 | 0.0797 | 0.3698 | -0.0202 | -0.0025 |
| O3 | 06069 | 166 | -0.0024 | 0.2103 | 0.2128 | -0.0042 | 0.0798 | 0.3391 | -0.0095 | 0.0009 |
| O3 | 25005 | 4,250 | -0.0022 | 0.1980 | 0.2003 | -0.0067 | 0.0800 | 0.3170 | -0.0127 | -0.0006 |
| O3 | 41009 | 176 | -0.0019 | 0.2026 | 0.2045 | -0.0097 | 0.0801 | 0.3264 | -0.0201 | 0.0006 |
| O3 | 28087 | 464 | -0.0001 | 0.2576 | 0.2576 | -0.0074 | 0.0801 | 0.4345 | -0.0175 | 0.0025 |
| O3 | 21141 | 200 | 0.0049 | 0.2115 | 0.2067 | 0.0054 | 0.0803 | 0.3412 | -0.0036 | 0.0140 |
| O3 | 50007 | 1,098 | -0.0005 | 0.1909 | 0.1914 | -0.0015 | 0.0804 | 0.3026 | -0.0032 | -0.0001 |
| O3 | 29183 | 809 | 0.0023 | 0.1935 | 0.1913 | 0.0029 | 0.0807 | 0.3053 | 0.0009 | 0.0048 |
| O3 | 45015 | 202 | -0.0022 | 0.2389 | 0.2413 | -0.0228 | 0.0812 | 0.3966 | -0.0507 | 0.0048 |
| O3 | 21167 | 139 | -0.0278 | 0.2773 | 0.3050 | -0.0398 | 0.0813 | 0.4751 | -0.0565 | -0.0214 |
| O3 | 10005 | 1,107 | -0.0014 | 0.1983 | 0.1998 | 0.0005 | 0.0821 | 0.3182 | -0.0021 | 0.0029 |
| O3 | 30111 | 948 | 0.0202 | 0.1587 | 0.1387 | 0.0356 | 0.0823 | 0.2384 | 0.0139 | 0.0555 |
| O3 | 55055 | 394 | 0.0080 | 0.2325 | 0.2244 | 0.0075 | 0.0843 | 0.3772 | 0.0015 | 0.0133 |
| O3 | 18085 | 341 | -0.0028 | 0.2365 | 0.2393 | -0.0099 | 0.0843 | 0.3885 | -0.0206 | 0.0007 |
| O3 | 34021 | 3,255 | 0.0058 | 0.1872 | 0.1814 | 0.0089 | 0.0844 | 0.2897 | 0.0045 | 0.0126 |
| O3 | 40109 | 6,609 | -0.0091 | 0.2373 | 0.2466 | -0.0166 | 0.0844 | 0.3926 | -0.0270 | -0.0061 |
| O3 | 29165 | 112 | 0.0026 | 0.1903 | 0.1879 | 0.0114 | 0.0846 | 0.2983 | 0.0000 | 0.0226 |
| O3 | 50001 | 170 | -0.0074 | 0.2016 | 0.2090 | -0.0051 | 0.0850 | 0.3185 | -0.0091 | -0.0010 |
| O3 | 39095 | 5,486 | -0.0058 | 0.2195 | 0.2254 | -0.0098 | 0.0854 | 0.3528 | -0.0156 | -0.0040 |
| O3 | 51013 | 1,436 | -0.0143 | 0.2310 | 0.2453 | -0.0275 | 0.0859 | 0.3780 | -0.0450 | -0.0091 |
| O3 | 21029 | 79 | 0.0091 | 0.1863 | 0.1773 | 0.0121 | 0.0862 | 0.2868 | 0.0052 | 0.0187 |
| O3 | 39153 | 4,879 | -0.0024 | 0.2411 | 0.2436 | -0.0097 | 0.0863 | 0.3957 | -0.0200 | 0.0004 |
| O3 | 21101 | 402 | -0.0019 | 0.2082 | 0.2101 | -0.0087 | 0.0863 | 0.3312 | -0.0180 | 0.0004 |
| O3 | 55027 | 739 | 0.0012 | 0.2396 | 0.2385 | -0.0041 | 0.0864 | 0.3948 | -0.0118 | 0.0029 |
| O3 | 47093 | 3,840 | 0.0025 | 0.2178 | 0.2153 | 0.0025 | 0.0868 | 0.3491 | 0.0017 | 0.0032 |
| O3 | 39165 | 318 | -0.0053 | 0.2303 | 0.2356 | -0.0166 | 0.0872 | 0.3770 | -0.0309 | -0.0018 |
| O3 | 24021 | 823 | -0.0040 | 0.2692 | 0.2734 | -0.0197 | 0.0873 | 0.4476 | -0.0408 | 0.0010 |
| O3 | 56039 | 61 | 0.0572 | 0.2215 | 0.1648 | 0.0931 | 0.0873 | 0.3677 | 0.0346 | 0.1494 |
| O3 | 42007 | 1,608 | -0.0059 | 0.2627 | 0.2685 | -0.0036 | 0.0877 | 0.4336 | -0.0074 | 0.0001 |
| O3 | 39085 | 1,420 | -0.0021 | 0.2221 | 0.2243 | -0.0091 | 0.0879 | 0.3585 | -0.0188 | 0.0005 |
| O3 | 42131 | 177 | -0.0011 | 0.2269 | 0.2281 | -0.0064 | 0.0879 | 0.3699 | -0.0145 | 0.0016 |
| O3 | 08041 | 1,889 | -0.0042 | 0.2195 | 0.2241 | -0.0104 | 0.0883 | 0.3537 | -0.0187 | -0.0019 |
| O3 | 35001 | 3,600 | 0.0037 | 0.2193 | 0.2157 | 0.0045 | 0.0888 | 0.3506 | 0.0031 | 0.0054 |
| O3 | 51510 | 852 | -0.0083 | 0.2484 | 0.2570 | -0.0155 | 0.0890 | 0.4092 | -0.0249 | -0.0057 |
| O3 | 55125 | 153 | -0.0126 | 0.2453 | 0.2579 | -0.0160 | 0.0894 | 0.4010 | -0.0220 | -0.0097 |
| O3 | 42101 | 20,838 | -0.0052 | 0.2500 | 0.2554 | -0.0137 | 0.0899 | 0.4120 | -0.0247 | -0.0023 |
| O3 | 06011 | 104 | 0.0023 | 0.2009 | 0.1987 | 0.0073 | 0.0900 | 0.3100 | 0.0007 | 0.0133 |
| O3 | 33005 | 453 | 0.0409 | 0.1603 | 0.1194 | 0.0533 | 0.0910 | 0.2300 | 0.0333 | 0.0705 |
| O3 | 17143 | 2,373 | -0.0021 | 0.2315 | 0.2337 | -0.0060 | 0.0912 | 0.3712 | -0.0113 | -0.0008 |
| O3 | 31109 | 1,769 | -0.0161 | 0.2802 | 0.2963 | -0.0206 | 0.0921 | 0.4696 | -0.0279 | -0.0126 |
| O3 | 51187 | 192 | -0.0004 | 0.2739 | 0.2744 | -0.0118 | 0.0926 | 0.4595 | -0.0290 | 0.0049 |
| O3 | 12009 | 2,688 | -0.0095 | 0.2668 | 0.2761 | -0.0161 | 0.0927 | 0.4391 | -0.0252 | -0.0068 |
| O3 | 12011 | 11,762 | -0.0085 | 0.2313 | 0.2398 | -0.0166 | 0.0927 | 0.3719 | -0.0271 | -0.0055 |
| O3 | 19113 | 1,341 | -0.0034 | 0.2278 | 0.2311 | -0.0007 | 0.0933 | 0.3643 | -0.0054 | 0.0036 |
| O3 | 13097 | 341 | -0.0338 | 0.3428 | 0.3762 | -0.0616 | 0.0938 | 0.5848 | -0.0972 | -0.0239 |
| O3 | 54107 | 1,100 | -0.0140 | 0.2929 | 0.3073 | -0.0262 | 0.0939 | 0.4920 | -0.0430 | -0.0090 |
| O3 | 18109 | 294 | -0.0237 | 0.3222 | 0.3460 | -0.0384 | 0.0942 | 0.5528 | -0.0588 | -0.0172 |
| O3 | 21111 | 7,652 | -0.0036 | 0.2545 | 0.2581 | -0.0118 | 0.0943 | 0.4152 | -0.0230 | -0.0006 |
| O3 | 06019 | 4,622 | -0.0033 | 0.2303 | 0.2335 | -0.0119 | 0.0943 | 0.3686 | -0.0233 | -0.0005 |
| O3 | 39081 | 867 | -0.0006 | 0.2599 | 0.2606 | -0.0044 | 0.0944 | 0.4281 | -0.0099 | 0.0007 |
| O3 | 47047 | 153 | 0.0421 | 0.2333 | 0.1914 | 0.0624 | 0.0944 | 0.3753 | 0.0322 | 0.0885 |
| O3 | 33007 | 361 | 0.0036 | 0.2144 | 0.2109 | 0.0068 | 0.0950 | 0.3337 | 0.0025 | 0.0105 |
| O3 | 21067 | 3,263 | 0.0036 | 0.2618 | 0.2582 | 0.0073 | 0.0950 | 0.4253 | 0.0022 | 0.0116 |
| O3 | 01101 | 2,329 | -0.0225 | 0.3063 | 0.3290 | -0.0377 | 0.0951 | 0.5206 | -0.0591 | -0.0156 |
| O3 | 21089 | 266 | 0.0147 | 0.2099 | 0.1953 | 0.0212 | 0.0951 | 0.3243 | 0.0112 | 0.0300 |
| O3 | 39049 | 7,840 | -0.0008 | 0.2478 | 0.2487 | -0.0053 | 0.0954 | 0.3994 | -0.0117 | 0.0010 |
| O3 | 37155 | 842 | -0.0126 | 0.2623 | 0.2749 | -0.0216 | 0.0959 | 0.4325 | -0.0345 | -0.0086 |
| O3 | 21177 | 300 | 0.0081 | 0.2267 | 0.2184 | -0.0008 | 0.0961 | 0.3570 | -0.0138 | 0.0117 |
| O3 | 06021 | 134 | 0.0217 | 0.2194 | 0.1977 | 0.0127 | 0.0974 | 0.3418 | -0.0033 | 0.0277 |
| O3 | 24015 | 478 | -0.0031 | 0.2618 | 0.2652 | -0.0101 | 0.0979 | 0.4281 | -0.0193 | -0.0007 |
| O3 | 27141 | 157 | 0.0175 | 0.2141 | 0.1967 | 0.0135 | 0.0982 | 0.3286 | 0.0036 | 0.0226 |
| O3 | 24003 | 1,942 | 0.0020 | 0.2203 | 0.2184 | 0.0074 | 0.0987 | 0.3463 | 0.0004 | 0.0141 |
| O3 | 22077 | 159 | 0.0102 | 0.2541 | 0.2439 | 0.0000 | 0.0990 | 0.4099 | -0.0168 | 0.0159 |
| O3 | 36103 | 10,403 | 0.0018 | 0.2690 | 0.2676 | -0.0025 | 0.0995 | 0.4371 | -0.0090 | 0.0034 |
| O3 | 25025 | 10,090 | -0.0008 | 0.2335 | 0.2344 | 0.0031 | 0.0997 | 0.3682 | -0.0021 | 0.0080 |
| O3 | 08077 | 812 | 0.0018 | 0.2361 | 0.2344 | 0.0065 | 0.0998 | 0.3749 | -0.0003 | 0.0129 |
| O3 | 21107 | 651 | 0.0089 | 0.2568 | 0.2479 | 0.0031 | 0.1005 | 0.4144 | -0.0065 | 0.0118 |
| O3 | 20209 | 1,860 | -0.0036 | 0.2674 | 0.2710 | -0.0066 | 0.1011 | 0.4306 | -0.0113 | -0.0022 |
| O3 | 12025 | 18,404 | -0.0017 | 0.2543 | 0.2566 | -0.0042 | 0.1013 | 0.4044 | -0.0077 | -0.0009 |
| O3 | 18097 | 8,594 | -0.0009 | 0.2409 | 0.2417 | -0.0036 | 0.1020 | 0.3830 | -0.0076 | 0.0002 |
| O3 | 42049 | 2,844 | -0.0086 | 0.2951 | 0.3041 | -0.0255 | 0.1020 | 0.4924 | -0.0477 | -0.0027 |
| O3 | 40143 | 4,849 | -0.0026 | 0.2760 | 0.2787 | -0.0077 | 0.1033 | 0.4514 | -0.0148 | -0.0007 |
| O3 | 24033 | 3,108 | 0.0049 | 0.2571 | 0.2525 | -0.0030 | 0.1040 | 0.4119 | -0.0145 | 0.0078 |
| O3 | 22051 | 3,137 | 0.0005 | 0.2404 | 0.2400 | -0.0015 | 0.1047 | 0.3766 | -0.0045 | 0.0012 |
| O3 | 40087 | 134 | -0.0068 | 0.3012 | 0.3082 | -0.0202 | 0.1053 | 0.4938 | -0.0409 | 0.0004 |
| O3 | 15001 | 636 | -0.0296 | 0.3095 | 0.3389 | -0.0500 | 0.1060 | 0.5154 | -0.0765 | -0.0216 |
| O3 | 42013 | 1,840 | -0.0057 | 0.2824 | 0.2883 | -0.0108 | 0.1069 | 0.4608 | -0.0185 | -0.0034 |
| O3 | 55133 | 1,770 | -0.0044 | 0.2781 | 0.2824 | -0.0118 | 0.1082 | 0.4460 | -0.0212 | -0.0022 |
| O3 | 34039 | 4,980 | -0.0045 | 0.2896 | 0.2943 | -0.0197 | 0.1082 | 0.4750 | -0.0404 | 0.0013 |
| O3 | 20091 | 1,411 | -0.0129 | 0.2718 | 0.2849 | -0.0215 | 0.1089 | 0.4341 | -0.0332 | -0.0094 |
| O3 | 22057 | 521 | 0.0292 | 0.2592 | 0.2304 | 0.0328 | 0.1092 | 0.4029 | 0.0233 | 0.0413 |
| O3 | 24043 | 1,167 | 0.0024 | 0.2682 | 0.2660 | 0.0019 | 0.1110 | 0.4239 | -0.0001 | 0.0038 |
| O3 | 08059 | 1,881 | 0.0162 | 0.2283 | 0.2121 | 0.0223 | 0.1118 | 0.3485 | 0.0129 | 0.0301 |
| O3 | 18059 | 324 | 0.0032 | 0.2547 | 0.2518 | -0.0026 | 0.1119 | 0.4001 | -0.0112 | 0.0052 |
| O3 | 06029 | 3,357 | -0.0013 | 0.2612 | 0.2624 | -0.0056 | 0.1123 | 0.4098 | -0.0117 | 0.0002 |
| O3 | 39093 | 1,938 | -0.0022 | 0.2897 | 0.2921 | -0.0104 | 0.1126 | 0.4694 | -0.0219 | 0.0010 |
| O3 | 24025 | 882 | -0.0047 | 0.3018 | 0.3068 | -0.0135 | 0.1127 | 0.4942 | -0.0252 | -0.0016 |
| O3 | 21145 | 1,145 | -0.0013 | 0.3151 | 0.3166 | -0.0110 | 0.1132 | 0.5178 | -0.0239 | 0.0019 |
| O3 | 34027 | 3,362 | -0.0005 | 0.2640 | 0.2647 | -0.0026 | 0.1134 | 0.4163 | -0.0072 | 0.0017 |
| O3 | 06061 | 1,105 | 0.0017 | 0.2835 | 0.2819 | -0.0039 | 0.1137 | 0.4518 | -0.0119 | 0.0037 |
| O3 | 42133 | 2,650 | -0.0126 | 0.3283 | 0.3412 | -0.0294 | 0.1150 | 0.5447 | -0.0505 | -0.0070 |
| O3 | 11001 | 8,817 | 0.0020 | 0.3090 | 0.3074 | 0.0009 | 0.1157 | 0.5006 | -0.0017 | 0.0035 |
| O3 | 34019 | 514 | 0.0009 | 0.3115 | 0.3108 | -0.0007 | 0.1157 | 0.5069 | -0.0034 | 0.0015 |
| O3 | 34025 | 4,981 | 0.0021 | 0.3124 | 0.3107 | 0.0075 | 0.1183 | 0.5049 | 0.0005 | 0.0141 |
| O3 | 51600 | 248 | 0.0107 | 0.2736 | 0.2631 | -0.0111 | 0.1183 | 0.4339 | -0.0440 | 0.0205 |
| O3 | 06033 | 414 | 0.0066 | 0.2600 | 0.2535 | 0.0117 | 0.1183 | 0.4006 | 0.0014 | 0.0215 |
| O3 | 13121 | 7,563 | -0.0069 | 0.2915 | 0.2981 | -0.0144 | 0.1187 | 0.4653 | -0.0254 | -0.0035 |
| O3 | 24031 | 4,186 | -0.0108 | 0.3346 | 0.3458 | -0.0255 | 0.1224 | 0.5542 | -0.0440 | -0.0061 |
| O3 | 51173 | 352 | -0.0015 | 0.3322 | 0.3341 | -0.0107 | 0.1250 | 0.5429 | -0.0235 | 0.0018 |
| O3 | 39151 | 3,791 | 0.0010 | 0.3236 | 0.3229 | -0.0080 | 0.1259 | 0.5199 | -0.0205 | 0.0040 |
| O3 | 24510 | 11,767 | 0.0055 | 0.3116 | 0.3065 | 0.0038 | 0.1262 | 0.4987 | 0.0006 | 0.0066 |
| O3 | 38065 | 4 | 0.0029 | 0.2716 | 0.2686 | 0.0590 | 0.1265 | 0.4189 | -0.0167 | 0.1351 |
| O3 | 39107 | 254 | -0.0039 | 0.3549 | 0.3591 | -0.0107 | 0.1278 | 0.5828 | -0.0210 | -0.0006 |
| O3 | 24005 | 4,751 | -0.0023 | 0.3412 | 0.3437 | -0.0097 | 0.1288 | 0.5546 | -0.0203 | 0.0005 |
| O3 | 34009 | 966 | 0.0108 | 0.3191 | 0.3086 | 0.0294 | 0.1291 | 0.5007 | 0.0037 | 0.0533 |
| O3 | 12115 | 3,560 | 0.0142 | 0.2755 | 0.2617 | 0.0196 | 0.1292 | 0.4263 | 0.0113 | 0.0267 |
| O3 | 06113 | 839 | 0.0033 | 0.3372 | 0.3343 | -0.0031 | 0.1303 | 0.5410 | -0.0123 | 0.0056 |
| O3 | 09003 | 7,558 | -0.0104 | 0.3453 | 0.3558 | -0.0216 | 0.1309 | 0.5630 | -0.0367 | -0.0059 |
| O3 | 18057 | 391 | 0.0082 | 0.3238 | 0.3159 | 0.0121 | 0.1314 | 0.5191 | 0.0064 | 0.0168 |
| O3 | 06101 | 366 | -0.0045 | 0.3361 | 0.3408 | -0.0055 | 0.1318 | 0.5334 | -0.0080 | -0.0032 |
| O3 | 38057 | 48 | 0.0356 | 0.2912 | 0.2557 | 0.0497 | 0.1322 | 0.4472 | 0.0264 | 0.0699 |
| O3 | 06067 | 6,979 | -0.0032 | 0.3260 | 0.3292 | -0.0104 | 0.1327 | 0.5193 | -0.0205 | -0.0004 |
| O3 | 51153 | 330 | 0.0067 | 0.3520 | 0.3457 | -0.0020 | 0.1328 | 0.5713 | -0.0158 | 0.0112 |
| O3 | 21103 | 52 | 0.1053 | 0.2506 | 0.1455 | 0.1397 | 0.1352 | 0.3644 | 0.0847 | 0.1871 |
| O3 | 26043 | 411 | -0.0078 | 0.3502 | 0.3579 | -0.0147 | 0.1357 | 0.5650 | -0.0249 | -0.0045 |
| O3 | 42071 | 3,268 | -0.0032 | 0.3576 | 0.3612 | -0.0141 | 0.1367 | 0.5803 | -0.0282 | 0.0002 |
| O3 | 34013 | 9,460 | -0.0052 | 0.3693 | 0.3749 | -0.0120 | 0.1432 | 0.5944 | -0.0216 | -0.0025 |
| O3 | 06065 | 6,586 | -0.0192 | 0.4024 | 0.4219 | -0.0423 | 0.1447 | 0.6679 | -0.0724 | -0.0103 |
| O3 | 55139 | 1,152 | 0.0044 | 0.3580 | 0.3536 | 0.0034 | 0.1483 | 0.5624 | 0.0010 | 0.0053 |
| O3 | 17083 | 180 | -0.0572 | 0.4493 | 0.5065 | -0.0748 | 0.1509 | 0.7549 | -0.1016 | -0.0448 |
| O3 | 27137 | 2,447 | 0.0185 | 0.3341 | 0.3158 | 0.0140 | 0.1559 | 0.5136 | 0.0047 | 0.0236 |
| O3 | 25001 | 1,880 | 0.0280 | 0.2962 | 0.2682 | 0.0434 | 0.1569 | 0.4426 | 0.0211 | 0.0640 |
| O3 | 19103 | 1,470 | -0.0222 | 0.4202 | 0.4430 | -0.0323 | 0.1600 | 0.6891 | -0.0465 | -0.0169 |
| O3 | 35043 | 145 | 0.0270 | 0.3075 | 0.2805 | 0.0442 | 0.1658 | 0.4560 | 0.0199 | 0.0664 |
| O3 | 06099 | 2,539 | -0.0008 | 0.4465 | 0.4476 | -0.0048 | 0.1762 | 0.7147 | -0.0106 | 0.0005 |
| O3 | 06071 | 8,449 | -0.0104 | 0.4334 | 0.4441 | -0.0232 | 0.1781 | 0.6940 | -0.0404 | -0.0054 |
| O3 | 17043 | 4,072 | 0.0001 | 0.4417 | 0.4416 | 0.0001 | 0.1947 | 0.6908 | -0.0015 | 0.0013 |
| O3 | 06037 | 62,601 | -0.0101 | 0.5215 | 0.5319 | -0.0246 | 0.2037 | 0.8458 | -0.0438 | -0.0047 |
| O3 | 08031 | 6,208 | -0.0106 | 0.5143 | 0.5256 | -0.0197 | 0.2098 | 0.8240 | -0.0329 | -0.0068 |
| O3 | 06093 | 336 | -0.0013 | 0.5450 | 0.5464 | 0.0075 | 0.2396 | 0.8551 | -0.0042 | 0.0187 |
| O3 | 06031 | 490 | 0.0196 | 0.5439 | 0.5244 | 0.0154 | 0.2474 | 0.8356 | 0.0059 | 0.0236 |

**Note:** AF, attributable fraction; CI, confidence interval; DLM, distributed lag model; iDLM, interactive distributed lag model. The iDLM columns report the AF attributed to main effects, the total AF, and the AF attributed to cascading interactions. Median annual deaths corresponds to the Total field in the source CSV files. All AF estimates and confidence limits are percentages.
